# Climate and demography drive 7000 years of dietary change in the Central Andes

**DOI:** 10.1038/s41598-022-05774-y

**Published:** 2022-02-07

**Authors:** Kurt M. Wilson, Weston C. McCool, Simon C. Brewer, Nicole Zamora-Wilson, Percy J. Schryver, Roxanne Lois F. Lamson, Ashlyn M. Huggard, Joan Brenner Coltrain, Daniel A. Contreras, Brian F. Codding

**Affiliations:** 1 Department of Anthropology, University of Utah, 260 S. Central Campus Drive, Room 4625, Salt Lake City, UT 84112 USA; 2 Global Change and Sustainability Center, University of Utah, Salt Lake City, UT 84112 USA; 3 University of Utah Archaeological Center, University of Utah, Salt Lake City, UT 84112 USA; 4 Department of Geography, University of Utah, Salt Lake City, UT 84112 USA; 5 Department of Anthropology, University of Florida, Gainesville, FL 32603 USA

**Keywords:** Climate-change impacts, Archaeology, Palaeoclimate, Socioeconomic scenarios

## Abstract

Explaining the factors that influence past dietary variation is critically important for understanding changes in subsistence, health, and status in past societies; yet systematic studies comparing possible driving factors remain scarce. Here we compile the largest dataset of past diet derived from stable isotope δ^13^C‰ and δ^15^N‰ values in the Americas to quantitatively evaluate the impact of 7000 years of climatic and demographic change on dietary variation in the Central Andes. Specifically, we couple paleoclimatic data from a general circulation model with estimates of relative past population inferred from archaeologically derived radiocarbon dates to assess the influence of climate and population on spatiotemporal dietary variation using an ensemble machine learning model capable of accounting for interactions among predictors. Results reveal that climate and population strongly predict diet (80% of δ15N‰ and 66% of δ13C‰) and that Central Andean diets correlate much more strongly with local climatic conditions than regional population size, indicating that the past 7000 years of dietary change was influenced more by climatic than socio-demographic processes. Visually, the temporal pattern suggests decreasing dietary variation across elevation zones during the Late Horizon, raising the possibility that sociopolitical factors overrode the influence of local climatic conditions on diet during that time. The overall findings and approach establish a general framework for understanding the influence of local climate and demography on dietary change across human history.

## Introduction

Identifying the long-term drivers of past spatiotemporal variation in individuals’ diets remains one of the grand challenges in archaeology^1^, yet systematic studies that quantify the relative influence of competing factors remain limited. Two factors, climate and population, may be particularly influential in structuring dietary variation. Local climatic conditions, proxies for environments, structure resource availability and land use strategies, resulting in patterned adaptations across similar environments^2–5^. Changes in those local climatic conditions may constrain viable subsistence adaptations by limiting agricultural viability^6–8^ or altering resource distributions, leading to dietary change. Demographically-driven resource competition may also drive major subsistence transitions^9,10^, including domestication^11,12^ and intensification^13^, whose proximate causes are innovation or crises^11,14^. Additionally, increasing population can cause changes such as altering mobility patterns^15^ and/or influence rising sociopolitical complexity^16^. Critically, the influence of both climatic^17^ and demographic^18^ factors goes beyond diet itself to impact health and longevity, as well as status and political complexity^19^. However, the interaction between climate and demography further complicates these dynamics: climatic change may structure population growth rates and carrying capacity^20–22^ leading to changes in diet and/or dietary variation^23^ or population change may drive people to alter their environments^24–26^, also potentially causing changes in diets and/or dietary variation. As a result, untangling the interactive drivers of climate and demography on spatiotemporal dietary variation and, therefore, its consequences for factors like health and status, remains difficult.

As a case study to systematically untangle and evaluate the influences of climatic and demographic change on diet we investigate the predictive power of both climate and population on individual diets over the past 7000 years in the Central Andes. Given the scarcity of long-term quantitative studies on diet in the region, first, we generate a database of 1965 archaeological individuals with stable isotope δ^15^N‰ or δ^13^C‰ bone collagen values from which we reconstruct both the patterns of dietary change over space and time and the variation between individuals’ diets. Due to the dramatic influence of elevation on environment^27^ and subsistence strategy^28^ in the Andes, we divide the study area into three elevational zones and classify each individual by location (see methods). The data reveal that Central Andean diets exhibit broad changes and vary significantly over time and space. To evaluate whether the observed variation results more from climatic factors or from population change, we analyze this spatially-explicit time series of individual diets using simulated paleoclimate derived from a general circulation model (GCM) and a population proxy generated from a dataset of 3957 archaeological radiocarbon dates. To address the analytic challenge posed by the likelihood that climatic and demographic change are linked^22,29^, we conduct the first quantitative inter-regional analysis of trans-Holocene diets in the Central Andes employing an ensemble machine learning model capable of accounting for interactions among predictors to assess whether the observed variation between individuals’ diets results more from changing local climates or from population. Our findings suggest that climate change is the dominant driver of Central Andean dietary variation.

### Regional background

The Central Andes are characterized by extreme heterogeneity in geography^27,30^ and spatially and temporally diverse cultures^31^. Prehispanic subsistence ranged from marine and high-elevation foragers^32–34^ to semi-sedentary pastoralists and agro-pastoralists^35–37^ to semi- and mostly sedentary agriculturalists occupying multiple elevational niches^38^. Prior research has documented the potential for environment^39^, demography^16,40^, climate^41^, and sociocultural factors^42,43^ to influence sociopolitical, economic, and dietary changes.

Climatically, the Central Andes experienced significantly changing conditions over the Holocene^44^. Variations in El Niño Southern Oscillation (ENSO) frequency^45,46^ and resultant effects such as sediment deposition^47^ and precipitation and drought^48,49^, have all heavily impacted the area. Spatial variation in the timing and intensity of ENSO appears to have existed between north and south Peru^46^, with staggered transitions in both regions from low intensity and dampened ENSO frequencies to modern ENSO frequencies over the past ~ 7000 years. Largely independent of ENSO, average temperatures in the Central Andes have also undergone changes and significant variation^44,50^, with sub-regions and localities exhibiting at times divergent patterns. Climatic variation in the Central Andes has been linked to socioeconomic changes^47,51,52^, intensification in production or infrastructure^41^, settlement shifts^53^, and population history^29,54^.

Few demographic reconstructions of population for the Central Andes exist, but those that do suggest populations were relatively low and stable early in the settlement of the region, with rapid increases in size later in time, though the rates and scales of population change differ by elevation^29,40,55^. Rapid population expansion^56^ and larger population sizes^57^ in the region appear to correlate with the incorporation of domesticates and transition to reliance on intensive agriculture as well as decreasing climatic volatility^29^. Here we generate a proxy of the past population sizes for each individual as a quantitative measure of the relative changes over time.

The sociopolitical trajectory, in general, of the Central Andes from the terminal Pleistocene until the arrival of the Spanish is one of increasing political interconnectivity. Particularly after ~ 4000 yBP, periods of greater and lesser political centralization occur, culminating in the region-wide Inca Empire (~ 480–418 yBP) ^31,58,59^. Initial domestication of resources began before 6000 yBP^38,60^, predating the emergence of more complex sociopolitical entities and integration which began by 3000 yBP^61,62^. Socio-cultural practices around ritual/religion, sociopolitical complexity, trade, elite manipulation, and cultural continuity and hegemony have been well documented in the Central Andes, with significant differences in practices, beliefs, and effects over space and time^63–66^. The changes in sociopolitical complexity in particular may be linked with population dynamics, as increasing population size may increase sociopolitical complexity and drive the rise of the state^16^ or vice-versa. Increasing sociopolitical complexity in the Andes produced varying patterns of influence and manipulation of resources and people by polities, including resettlement of individuals^67,68^, interconnectivity through trade^69,70^, and altering social dimensions of food^43,71^, all of which could influence individual diets.

## Results

**Figure 1:**
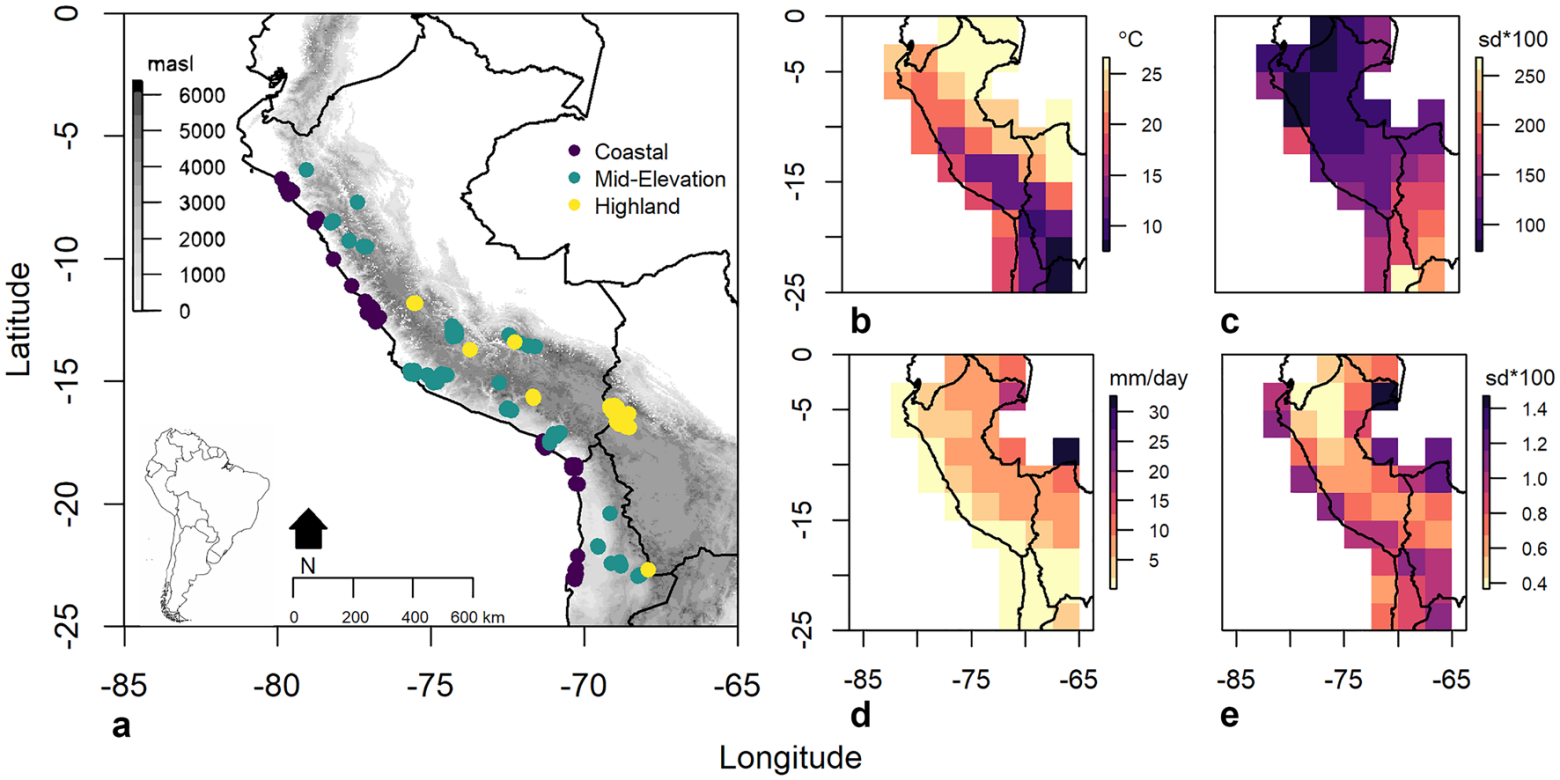
Study area and spatial climate variation. Left: (**a**) Map of locations from which individuals are derived. Colors correspond to the elevation zone. Right: Averaged climate value for each grid cell from the past 7000 years for the study area. Climate variables are (**b**) temperature (°C), (**c**) temperature seasonality (standard deviation in °C * 100), (**d**) precipitation (mm/day), and **e)** precipitation seasonality (standard deviation in mm/day * 100) generated from TraCE21ka simulations^119,120^ through the PaleoView Paleoenvironmental Reconstruction Tool^122^. Maps were produced in the R statistical environment^136^ using the **mapdata**^150^ and **raster**^151^ packages with shapefiles obtained from the NOAA Global self-consistent, hierarchical, high-resolution geography database^152^.

**Figure 2:**
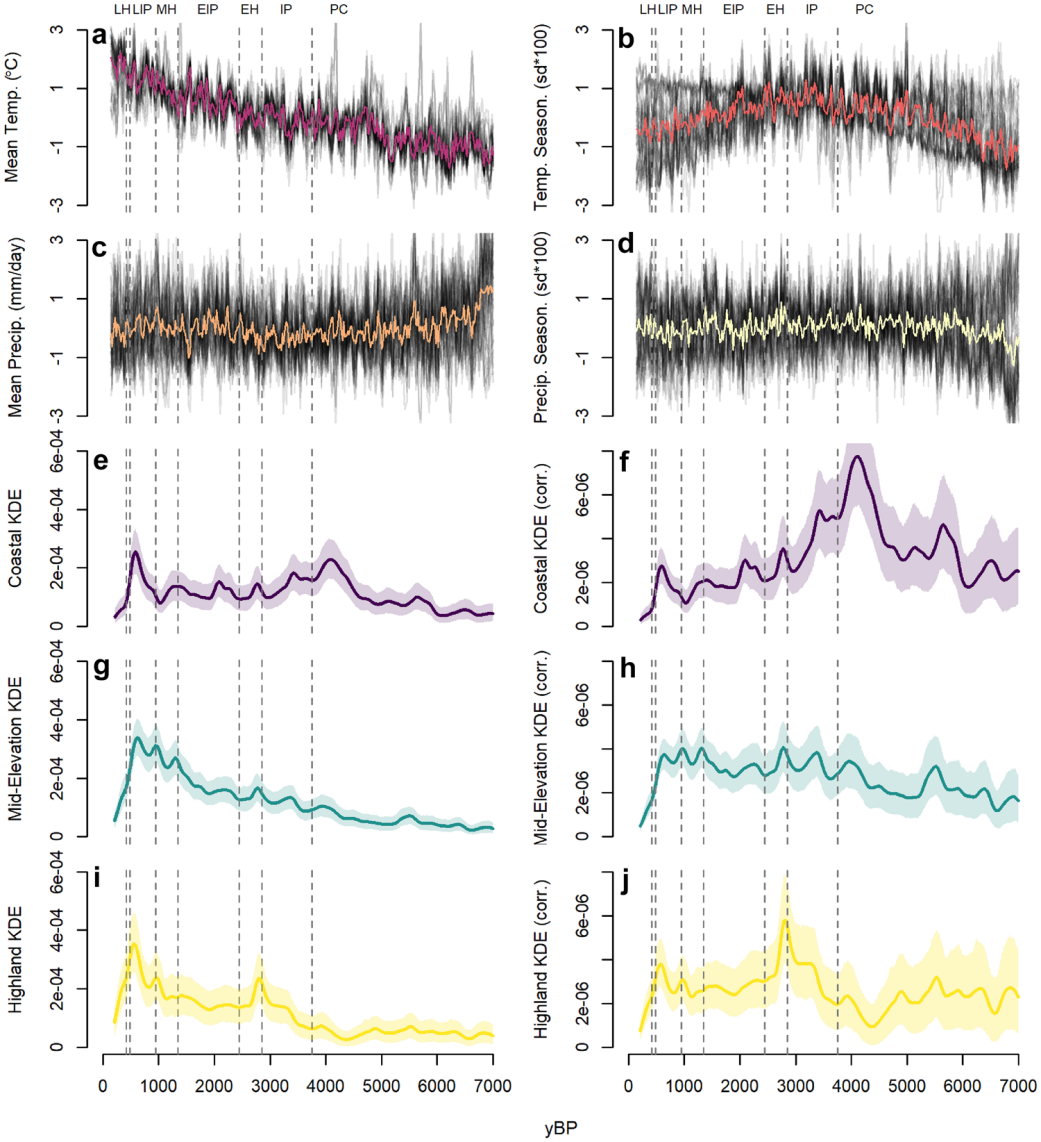
Time series of climate and demographic variables. (**a–d**) z-scores of temporal climate deviation from the mean for the past 7000 years. Each grey line represents the z-score variation of an individual grid cell (see Fig. 1). Colored lines represent the moving average z-score for all raster grid cells combined. In order, the variables are (**a**) mean temperature (°C), (**b)** temperature seasonality (sd °C * 100), (**c**) mean precipitation (mm/day), and (**d**) precipitation seasonality (sd mm/day * 100). (**e–j**) Coastal, mid-elevation, and highland KDE estimates documenting relative variation in population size over time for each elevation category employing uncorrected (left) and taphonomically corrected (right) KDEs. For analytical purposes, individuals receive climate value estimates from the time series of the grid cell at their spatial coordinates, not the moving average central tendency line.

Examining individual diets represented by δ^15^N‰ or δ^13^C‰ measured on bone collagen as a function of paleoclimate reconstructions from a GCM (Figs. 1, 2) and paleo-demographic reconstructions using Kernel Density Estimates (KDEs) (Fig. 2) depicts how the long-term dietary patterns in the Central Andes are influenced by climate and population.

To control for the elevation induced effects on climate^27^ and subsistence strategies^28^, here individual diets and broader dietary change are analyzed across three elevation zones: coastal, mid-elevations, and highlands (see methods for details on assignation of individuals). These zones approximate elevation-influenced subsistence patterns^28,31,35^ and allow for: a) reconstruction of general dietary change within similar strategies, and b) evaluation of if the effects of climate and population vary by subsistence strategy.

### Visualizing temporal variation in Central Andean diets by elevation zone

**Figure 3:**
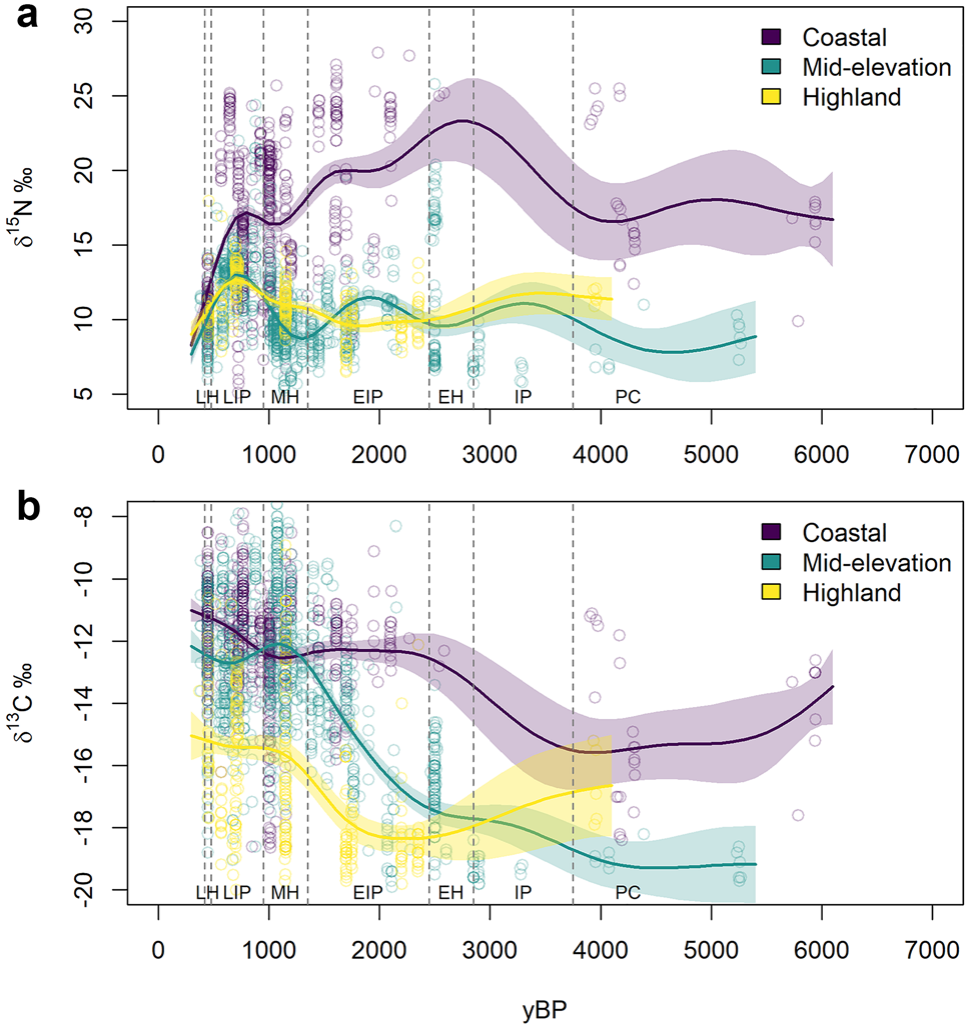
Generalized additive model time series. GAM regression results for both (**a**) δ^15^N‰ and (**b**) δ^13^C‰ across the three elevation categories. Dots represent the observed individuals plotted at their median date determined via resampling of their date range 10,000 times (weighted by calibrated radiocarbon probability for directly dated individuals). Solid lines are the central tendencies for each elevation category with shaded 95% confidence intervals. Lines were generated by creating 10,000 GAMs per elevation zone and isotope (60,000 total) where, for each GAM, each individual received a single year as their date, sampled via weighted sampling of their date range. The central tendency line is the mean fit line of the 10,000 GAMs for each sample, with 95% CIs created from the mean standard error and standard deviation of the 10,000 GAMs for each sample. Time periods follow the chronological periods from Moseley^58^: Preceramic (PC), Initial Period (IP), Early Horizon (EH), Early Intermediate Period (EIP), Middle Horizon (MH), Late Intermediate Period (LIP), Late Horizon (LH). Note: This is a time series only and does not incorporate space. Some of the variation within elevation zones relates to spatial variation which is addressed in the RF analyses.

To visualize temporal variation in diet, we collapse spatial variation into the three elevation categories and fit the trend using bootstrapped generalized additive model (GAM) regression. This reveals several patterns in broad dietary change over time across elevation zones (Fig. 3). (1) Unsurprisingly, diets differed significantly depending upon the elevation zone in which an individual lived. Until the Late Horizon period, coastal individuals generally have δ^15^N values 5 to 10‰ higher than all others. This is combined with 2 to 6‰ higher δ^13^C until about 1300 yBP. Mid-elevation individuals mostly have diets around 10‰ δ^15^N over the entire period, though the typical δ^13^C values change drastically between ~ 2500 yBP and 1350 yBP. Highland individuals have similar δ^15^N values as those from mid-elevations but generally have δ^13^C values that are 2 to 4‰ lower than mid-elevation individuals and up to 6‰ lower than those on the coast. (2) An increase in δ^13^C values over time is noticeable across each elevation zone, with the largest change in the mid-elevations. This increase is smallest on the coasts and likely occurs sometime between the Preceramic and Early Horizon. In the mid-elevations and highlands, the increase occurs later in time, appearing to happen during the Early Intermediate Period, with mid-elevation values generally experiencing a ~ 6‰ increase with highland values increasing by about 3‰. (3) The central tendency in δ^15^N for mid-elevation individuals appears to possess some variation dependent upon period, in particular showing elevated values 2 to 4‰ higher during the Early and Late Intermediate Periods relative to the Middle and Late Horizons. (4) The amount of differentiation between individuals’ diets diminishes during the Late Horizon. During this period, δ^15^N values across each elevation overlap with each other around 10 to 11‰. At the same time, δ^13^C values for mid-elevation and coastal individuals are quite similar, around − 11 to − 12‰, though highland values continue to remain 3 to 4‰ lower. These patterns are also visible in 95% confidence interval ellipses plots (Supplementary Fig. 1, Supplementary Material 2).

### Climate and population predict past diets

The cross-validated random forest (RF) regression models evaluating the predictive power of climatic and population change on diet reveal that combined, our climate and population variables explain 79.82 and 66.02% of the deviance in δ^15^N‰ and δ^13^C‰ respectively when using uncorrected KDEs, or 80.04% and 66.77% when correcting for taphonomic loss. Root mean square error values for δ^15^N‰ models are 2.05 (uncorrected KDE) or 2.04 (corrected KDE) and are 1.62 or 1.60 for δ^13^C‰. As such, combined climate and population estimates explain the majority of dietary variation, regardless of demographic estimate employed. Model diagnostics reveal residuals are normally distributed (Supplementary Figs. S6, S8, S10, S12) around zero, possess limited, unstructured temporal autocorrelation to about 200 years (Supplementary Figs. S7, S9, S11, S13), and do not possess spatial autocorrelation, indicating that the models perform well in predicting the variation in δ^15^N‰ and δ^13^C‰ across time and space.

### Climate has a stronger influence on diets than population

**Figure 4:**
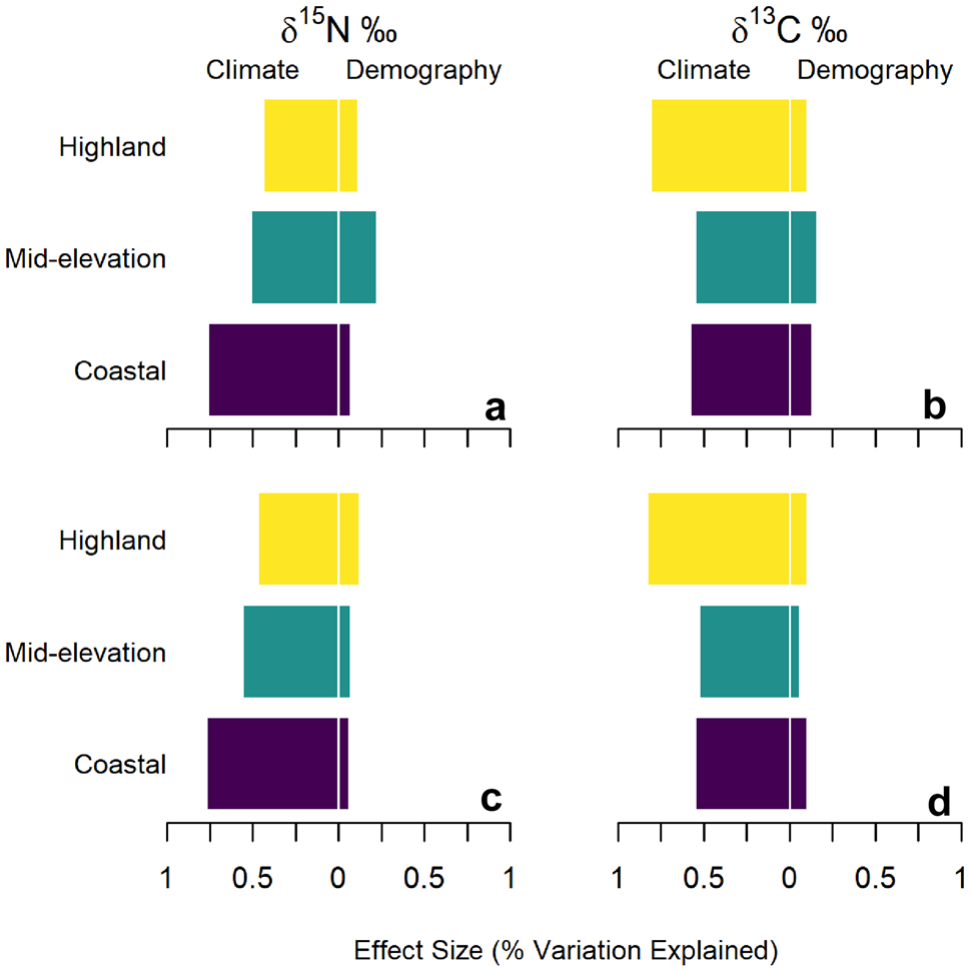
Cumulative climate and demographic effect sizes. Cumulative climate and demographic effect sizes (percent of the per mil amount of variation explained) within each elevation category for δ^15^N‰ (left column) and δ^13^C‰ (right column). (**a,** **b**) The cumulative effect sizes employing the uncorrected KDEs. (**c, d**) The cumulative effect sizes employing the taphonomically corrected KDEs. Regardless of KDE used for demographic estimates, climate change consistently has a larger effect than demography.

As the models perform well in predicting diet, we employ effect size calculations for each predictor variable within each elevation zone to evaluate whether climate or population change has a greater impact. Effect sizes, controlled for the interactions of all predictor variables, reveal climate has a much larger effect on δ^15^N‰ and δ^13^C‰ than population (Tables 1, 2, Fig. 4). Climate, cumulatively, has an effect size ranging from two to fourteen times that of demography across both isotopes.

**Table 1:** Random Forest Model Effect Sizes with uncorrected KDEs.

Isotope	Elevation category	Temp. EF‰ (%)	Temp. seasonality EF‰ (%)	Precip. EF‰ (%)	Precip. seasonality EF‰ (%)	Cumulative climate EF‰ (%)	Demography EF‰ (%)
δ^15^N‰	*Coastal*	1.4 (5.6%)	6.5 (25.7%)	10.2 (40.0%)	1.2 (4.8%)	19.4 (76.2%)	1.6 (6.6%)
	*Mid-elevation*	0.7 (4.1%)	2.0 (11.8%)	5.3 (29.9%)	0.9 (5.5%)	9.0 (51.2%)	3.8 (22.1%)
	*Highland*	0.4 (8.5%)	0.4 (9.3%)	0.5 (11.0%)	0.7 (15.4%)	2.1 (44.1%)	0.5 (11.2%)
δ^13^C‰	*Coastal*	0.7 (10.3%)	0.7 (10.2%)	1.5 (21.0%)	1.2 (16.7%)	4.1 (58.1%)	0.9 (13.3%)
	*Mid-elevation*	1.0 (6.4%)	2.6 (15.9%)	1.8 (11.0%)	3.5 (21.2%)	8.9 (54.5%)	2.7 (16.4%)
	*Highland*	2.2 (35.4%)	0.6 (9.6%)	1.1 (18.7%)	1.1 (17.1%)	4.9 (80.7%)	0.6 (10.4%)

Model sum of squares effect sizes (amount of variation in δ^15^N‰ or δ^13^C‰ explained) for each variable within the elevation categories. These values are in per mil (‰). Percentages are the model sum of squares effect sizes as a percent of the total amount of variation. Effect sizes are directly comparable within each isotope, but not between them, given that there are different models for each stable isotope. Values here have been deducted by Friedman's *H*-statistic to remove the proportion of the effects resulting from interaction between variables. Here demography is generated via uncorrected KDEs. Note: the effect sizes are calculated through iterated simulation employing representative subsets of each key predictor variable, as they do not include every possible combination the percentages will not sum to 100.

**Table 2:** Random forest model effect sizes with corrected KDEs.

Isotope	Elevation category	Temp. EF‰ (%)	Temp. seasonality EF‰ (%)	Precip. EF‰ (%)	Precip. seasonality EF‰ (%)	Cumulative climate EF‰ (%)	Demography EF‰ (%)
δ^15^N‰	*Coastal*	1.7 (6.7%)	6.7 (26.4%)	10.1 (39.4%)	1.3 (5.0%)	19.7 (77.4%)	1.4 (5.7%)
	*Mid-elevation*	1.2 (6.9%)	2.0 (11.5%)	5.0 (28.7%)	1.5 (8.8%)	9.7 (55.9%)	1.3 (7.4%)
	*Highland*	0.5 (10.8%)	0.6 (12.5%)	0.4 (9.4%)	0.7 (14.2%)	2.2 (47.0%)	0.6 (11.6%)
δ^13^C‰	*Coastal*	0.8 (10.9%)	0.7 (10.6%)	1.2 (17.7%)	1.1 (15.9%)	3.9 (55.1%)	0.7 (10.1%)
	*Mid-elevation*	1.0 (6.5%)	2.8 (17.0%)	1.5 (9.0%)	3.3 (20.3%)	8.6 (52.7%)	1.0 (5.9%)
	*Highland*	2.1 (34.9%)	0.6 (10.6%)	1.1 (18.8%)	1.1 (18.4%)	5.0 (82.7%)	0.6 (10.4%)

Model sum of squares effect sizes (amount of variation in δ^15^N‰ or δ^13^C‰ explained) for each variable within the elevation categories. These values are in per mil (‰). Percentages are the model sum of squares effect sizes as a percent of the total amount of variation. Effect sizes are directly comparable within each isotope, but not between them, given that there are different models for each stable isotope. Values here have been deducted by Friedman's *H*-statistic to remove the proportion of the effects resulting from interaction between variables. Here demography is generated via taphonomically corrected KDEs. Note: the effect sizes are calculated through iterated simulation employing representative subsets of each key predictor variable, as they do not include every possible combination the percentages will not sum to 100*.*

Regardless of demographic proxy employed (taphonomically uncorrected or corrected KDE), climate explains the majority of the variation in the isotopic values between individuals across space and time. Even considering climatic variables independently suggests that individual climatic factors are more influential than demography in every elevation zone for both isotopes (Tables 1, 2). The one analytical location where the influence of demography comes close to that of climate is mid-elevation δ^15^N‰, where, across the temporal range of observations, the cumulative effect of climate is a little over two times (Table 1) that of demography, measured as uncorrected KDEs, and the effect size of demography is ~ 3.8‰.

Individually, precipitation is the most influential climate variable for coastal and mid-elevation δ^15^N‰ regardless of KDE employed, whereas in the highlands, precipitation seasonality has the largest effect on δ^15^N‰ (Tables 1, 2). For δ^13^C‰, each of the climate variables tend to be similarly correlated in the coastal and mid-elevations, with mean temperature having a larger relative effect in the highlands (Tables 1, 2).

When using uncorrected KDEs, population size fairly consistently correlates with around 13% of the variability, across isotope and elevation category, with the noted exception of mid-elevation δ^15^N‰ where demography corresponds with 22.1% (~ 3.8‰) of the variability. When using corrected KDEs, population size generally accounts for around 10% of the variability, maxing out at 11.61% (0.60‰) for highland δ^15^N‰.

In terms of per mil variability, the demographic effect corresponds with potentially meaningful variation (> 1.0‰) in mid-elevation δ^15^N (3.8‰) and δ^13^C (2.7‰) as well as coastal δ^15^N (1.6‰) when using uncorrected KDEs. When using corrected KDEs, the most meaningful effects of demography are on coastal and mid-elevation δ^15^N (1.4 and 1.3‰ respectively). However, these values are smaller than most of the individual climate variable effects and much smaller than the respective cumulative effects of climate. See Supplementary Tables S1 to S4 for the complete set of Friedman’s *H* statistics and both initial and deducted effect size values for each variable within each elevation category.

## Discussion

Reconstruction of the long-term dietary trends in the Central Andes reveals significant dietary variation existed, particularly between, though also within, elevation zones. Given the isotopic differences between marine, terrestrial mammal, and terrestrial plant resources (see methods for overview), variation between elevation zones is enhanced by what seems to be multi-trophic level difference from coastal to all other individuals. Based on δ^15^N‰, this difference is likely driven by differential marine resource consumption. Ready access to and reliance on marine resources is well-documented^72–75^, and should increase relative δ^15^N‰. Until around 1350 yBP, δ^13^C‰ shows significant differences between coastal and other individuals as well. While marine resources certainly contribute to this pattern given their typical values^76,77^, the δ^13^C‰ differences may also suggest different levels of reliance on C3 vs C4 plants, with mid-elevation individuals more heavily C3 reliant up until around 1350 yBP. By ~ 1350 yBP, increasing mid-elevation and highland δ^13^C‰ may suggest an increase in maize consumption (as food or as *chicha*), which has been suggested to have taken longer to establish in the mid- and high elevations and southern latitudes than on the more northerly coasts^38,78,79^. Alternately, it could be evidence of greater inclusion of low-trophic level marine resources farther inland^70^ or of animal husbandry practices employing greater amounts of C4 fodder^80^. Consistently however, highland individuals possess lower δ^13^C‰ values than all others, suggestive of continual use of more C3 resources than in the other zones. This may be expected, particularly as the ceiling for maize cultivation today is generally ~ 3400 masl, though specific microenvironments enable cultivation up to ~ 3800 masl^7,38,81^.

Our results suggest the observed differences in individuals’ diets are driven by local climatic conditions: climate has a much stronger correlation than relative population size with stable isotope measured individual diets in the Central Andes. Crucially, for the analysis, our results are derived from local spatiotemporal measures. This means that while the central tendency in dietary change (Fig. 3) captures the general temporal trends, our results (Fig. 4) reveal the predictive power of climate and population when considering spatial as well as temporal variation. The pattern of climate having more influence than population holds whether considering climate variables individually or their cumulative effect and regardless of whether corrected or uncorrected demographic estimates are employed (Fig. 4, Tables 1, 2). Given that the RF models explain ~ 80% of the variance in δ^15^N‰, and ~ 66% in δ^13^C‰, and that climate is the significantly stronger correlate, our results suggest that local climate strongly constrains viable subsistence adaptations in the Central Andes. As the local climatic conditions experienced by individuals changed through time, individuals adapted by modifying their diets or subsistence strategies. Given our inability to control for foddering or fertilization for each of our individuals, and our reliance exclusively on δ^13^C‰ and δ^15^N‰, we cannot yet determine if this correlation is due to changing food items in diets or changes in subsistence practices that maintain the same food items but alter their isotopic signature. Likely both occur and both may produce large changes in the isotopic signature of resources. For instance, some of the dietary changes over time may represent greater incorporation of new resources, such as maize^60,82–84^. However other dietary changes may result from the introduction of new practices like fertilization or increased animal foddering^80,85–87^. If these new subsistence practices were responses to climate change, as has been suggested for multiple changes in practices^41,47,51,52^, this would be captured in our analysis as climatic effect on diet even though the foods being consumed did not change.

The significantly stronger impact of climate than population on isotope value, and the fact that the majority of our samples come from the past 2000 years, may suggest that vertical migration and horizontal exchange between groups in the Central Andes^54,88^, intensification in response to heightened levels of violence and warfare^89^, and innovations in subsistence economy techniques^41,51^ relate to local climate more strongly than demographically induced competition or sociopolitical change. However, demography, regardless of corrected or uncorrected population proxy, does consistently account for around 10–13% of the effect, indicating population changes exert some influence. Our results suggest this effect may be largest for mid-elevation δ^15^N‰. A larger mid-elevation effect may relate to the heavier dependence on agriculture as a key subsistence strategy as competition over arable land may lead to violence^89,90^, state level formation^30^, co-option of land^91^, or extensification; all of which could interact with population size to have a bigger influence on mid-elevation diets. Further, it is plausible that the incised drainages of the mid-elevations allowed changes in population size to have more dramatic influences on population density than in other areas. Intensification in response to density changes that produces greater reliance on agriculture through fertilization^92^ or terrace building^93^, as well as trade^69^, or other behaviors such as water engineering may be more necessary for Central Andean mid-elevation agriculturalists than for those individuals with access to more marine or pastoral resources. It is also plausible that demographically driven competition caused intensification when and where climatic constraints allowed, such as in the middle elevations, but caused scarcity in others. Future work evaluating if this 10%, or larger in the case of mid-elevation δ^15^N‰, effect is consistent over time and space or driven by stronger population influences during a particular period(s) may unveil new insights into the temporal dynamics of population influence in the Andes. For instance, it is plausible that alteration in subsistence techniques could occur coincident with climate change, but not in response to it (i.e., Boserupian intensification resultant from increasing population separated from climatic effects), and future studies may be able to elucidate if this occurs at various points in time in the Central Andes.

Divergent from the rest of the time periods however, during the Late Horizon isotope values from most mid-elevation and coastal, as well as some highland, individuals overlap. Individuals living on the coast experienced a sharp drop in δ^15^N‰, with a similar albeit smaller decrease experienced by mid-elevation and highland individuals. Particularly on the coast this is a surprise as it appears to be a decrease in either the amount or trophic level of marine resources consumed, despite a near 7000-year history of coastal individuals making heavy use of high δ^15^N‰ marine resources. At the same time, mid-elevation and coastal δ^13^C‰ continued to be nearly indistinguishable. This stark decrease in dietary variation between individuals occurs temporally rapidly over broad elevation gradients. These findings may result from a warming less seasonal environment, which could plausibly decrease variability in resources while increasing the economic payoffs from agriculture, or from sociopolitical change.

Overall, earlier in time, climate appears to have been more locally disparate (Fig. 2), with somewhat greater deviations from the mean in temperature, temperature seasonality, mean precipitation, and precipitation seasonality than later in time. In the context of marked spatial variation in climates, local resources would have been varied in type, presence and abundance. Over the past 1300 years, however, temperatures in the region continued to rise significantly above the mean, temperature seasonality generally decreased from the preceding 1000 years, and precipitation and precipitation seasonality became slightly more spatially varied from preceding periods, with some locations experiencing decreased precipitation and seasonality while others saw increases (Fig. 2). Taken together, a warmer and potentially less seasonal environment may have decreased some of the variability in resources, either latitudinally or longitudinally, while increasing the viability of agriculture as a subsistence strategy, which could cause the differences between individuals’ diets to diminish. Diminishing differentiation was potentially occurring between ~ 1300 and 500 yBP and appears to have strongly occurred during the Late Horizon.

Alternatively however, dietary convergence may support earlier work by Burger, et al. ^94^ suggesting Inca administration may have led to heavy reliance on maize across the Central Andes, though their conclusions were based dominantly on individuals from Machu Picchu. Given the spatial spread and political influence of the Inca, such convergence in the isotope data likely reflects the power of the Inca Empire, realized through state level redistribution of resources^69,95,96^ and people^67^, as well as emphasis on the social importance of maize^71^. These activities may have collapsed dietary variation across elevation zones and may reflect increasing homogenization in subsistence practices. The overall pattern of dietary convergence coinciding with increasing sociopolitical integration and potential genetic homogenization^97^ suggests the possibility of strong interplay between social processes and consumption late in time.

During the Middle Horizon (~ 1350–950 yBP), both the Tiwanaku and Wari Empires engaged in regional integration, trade, and resettlement^98,99^ as well, and they contributed to changing social dimensions of maize^43,65^, yet individuals’ diets remained quite distinct both between and within elevation zones during this period. Mid-elevation and coastal δ^13^C‰ does overlap, but δ^15^N‰ stays about 5 to 8‰ higher on the coasts even as inland transport of marine resources was possible and occurring, at least in southern Peru^70^. Additionally, during the Late Intermediate Period (~ 950–480 yBP), vertical migration between groups^54^ and horizontal exchange^88^ suggest economic and/or political as well as social interconnectivity between individuals in different elevation zones existed. This may be expected to significantly decrease dietary variation and, yet, large dietary differentiation between and within elevation zones remained. Thus, our results may suggest that either integration between the coast and mid-elevations was not widespread or may have been limited in its scope during the Middle Horizon and Late Intermediate Period. Taken together with the pattern during the Late Horizon, our results may suggest that, in the Late Horizon, the influence exerted by the Inca Empire overrode local climatic influences on diet in ways the Wari and Tiwanaku Empires and the increased Balkanization of the LIP could not. Alternately, given the strength of climate in our analysis, it is possible that continued climate change drove optimal socioeconomic decisions causing subsistence behaviors to fixate on similar resources. Finally, there is also potential for unique interactions between climate change and sociopolitical processes in the Late Horizon.

The sharp convergence of diets during the Late Horizon and the ~ 20–35% of the variation remaining unexplained by our models suggests that though climate change is the dominant influence it is not the sole explanatory factor. The remaining variation may be representative, in part, of the effect that sociopolitical complexity and other social factors had on dietary variation. There are several possible explanations for how sociopolitical complexity could account for remaining variation even though demographic influence within the models is small. First, it may be that the effects of sociopolitical complexity on dietary variation are divorced from population effects, meaning the number of people involved is less important a factor than the influences exerted by state-level decisions, organizational complexity, changing ritual practices, market integrations, etc., particularly if those factors are not driven by population change. This could be especially true if sociopolitical institutions were adapting to climate change by promoting behaviors such as exchange, redistribution, or increased forays into multiple elevation zones. Such adaptations in response to climate change would result in decreasing dietary variation that correlates strongly with climate but not population size. Second, it is possible that population does have a larger effect and correlate with sociopolitical complexity but that our climate proxies more accurately estimate past climate than our demographic proxies estimate relative past population sizes. Finally, it is possible, maybe likely, that our population reconstructions underestimate relative population size during the Late Horizon in particular, which could decrease the influence of demography if it were having a significantly stronger impact on individuals during that period than over the rest of the study period. Each of these possibilities warrants future investigation.

To further evaluate whether Central Andean dietary change may be driven by changing foods or subsistence practices and what may be causing the Late Horizon dietary overlap, significant future work is required. Estimates of sociopolitical influence at the site level both within and between temporal periods along with increased sample sizes of multiple different light isotopes, greater temporal control for most individuals within the region, improved climate change proxies, expanded datasets on non-isotope proxies for diet (i.e., zooarchaeology, paleobotany), and increased multi-site investigations will all be necessary. Combining these various datatypes will prove useful in generating unique insights into the long-term human–environment interactions in the Central Andes.

Here we show that in a region defined by its unique cultural, elevation, subsistence, and climatic heterogeneity, the local climatic conditions individuals experienced during their lives were the strongest factors influencing their dietary patterns. Our models employing simulated climate with relative population size explain ~ 80% of the variation in δ^15^N‰ and ~ 66% in δ^13^C‰ across the past 7000 years. While we anticipate more detailed reconstructions of local climate change, more nuanced demographic reconstructions, and future incorporation of social factors may increase the amount of the dietary variation explained, these current variables perform well in constraining isotopic values of diet. Our results suggest that climatic changes are a stronger correlate with dietary differentiation between individuals than changing population sizes. Though we suspect sociopolitical complexity impacted dietary variation as well, its influence appears constrained within a pattern of climatic change and environmental limitation, at least until the Late Horizon. Such a result implies that past and future climatic change did and will highly influence subsistence decisions and dietary outcomes. Given the rapid climate changes occurring in the world today, our analysis of dietary change over the past 7000 years in the Central Andes implies that generating projected climatic changes will be highly productive in predicting health^17^ and complexity^19^ changes in the future as these aspects of life are intimately impacted by subsistence.

The approach presented here offers a systematic framework for exploring the relative role of climate and other socio-demographic factors on dietary change through time. We suspect that climate will have a similarly outsized influence on long-term dietary change in other regions where systematic studies have yet to be undertaken.

## Materials and methods

### Data

We compiled a database of 1965 individuals (Supplementary Data File 1) from published literature in Peruvian, northern Chilean, and Lake Titicaca archaeological contexts (Fig. 1) with δ^13^C‰ or δ^15^N‰ measured on bone collagen. Nearly 40 years of isotopic studies have documented that carbon and nitrogen isotope ratios from human bone collagen in the Central Andes are responsive to the proportion of C3/C4 plants, marine/terrestrial animal input, and other dietary behaviors, effectively capturing variation in diets^100–104^. For our analysis, we exclude individuals under the age of five to control for weaning effects but include other subadults noting that although subadult isotope data represent a shorter window of life compared to adults, these data are independent of nursing induced trophic level enrichment^105,106^. Individuals are further evaluated using reported C:N ratios; those with C:N ratios between 2.9 and 3.6^107^ are accepted as reliable data. Each individual outside that ratio is checked for the reporting author’s decision on whether to accept the isotope values as valid. If the reporting author accepts a value outside the 2.9–3.6 ratio we include that individual in the dataset. In effect, this process expands the acceptable C:N ratio to 2.6–3.6, as many authors rely upon the 2.6–3.4 C:N ratio proposed by Schoeninger, et al^108^. Some individuals do not have a direct C:N ratio, but the reporting author confirmed in text that all individuals were within the acceptable C:N range, resulting in inclusion in our analysis. If authors did not report a C:N ratio, specifically or study-wide, but accept their data as valid, we include the data (n = 122, 7% of the sample). As a final step, we drop Colonial Period individuals (n = 25) to constrain the analysis to pre-Spanish arrival. This process results in a sample of 1767 individuals, 1727 with bone collagen δ^15^N values and 1761 with bone collagen δ^13^C (Supplementary Data File 2). These isotope values, which represent each individuals’ average adult diet^105^, span the past ~ 7000 years.

Elevation in the Andes affects climate^27^ and subsistence modes/opportunities^28^. Therefore, we group individuals into three elevation categories (coastal, mid-elevation, and highland) which roughly correspond to elevation-influenced subsistence patterns: agro-marine with heavier marine influence on the coast, agro-marine-pastoral in the mid-elevations with greater emphasis on agriculture, and agro-pastoral with heavier influence of pastoralism in the highlands^28,31,35^. This allows us to i) evaluate the long-term trends in dietary variation across different subsistence strategies, ii) more directly examine whether climate or population have greater effects on dietary variation by examining influences within elevation categories, and iii) evaluate if the effects of climate and population size vary between the categories, implying differential influences based on primary subsistence pattern.

We establish the elevation category (coastal, mid-elevation, highland) for each individual by extracting elevation (meters above sea level, masl) from a digital elevation map (DEM) and employing an adapted version of the natural zones from Pulgar Vidal^27^. Any individual recovered from less than 350 masl and a distance to coast of less than 15 km is categorized as coastal. Individuals recovered from less than 3500 masl, but above 350 masl, or less than 350 masl but greater than 15 km from the coast are considered mid-elevation. Individuals recovered from above 3500 masl are categorized as highland. Defining elevation zones places the measure of elevation on a level of precision comparable to our climatic and demographic data and allows us to control for some of the spatial variation to enhance analyzing variation over time.

### Stable isotopes

Isotope values of bone accurately record isotopic composition of dietary inputs with known fractionation offsets and thus can be used to examine dietary variation between groups and individuals^109–111^. Here we rely solely on bone collagen δ^13^C and δ^15^N per mil values as our inferred dietary measure to make all individuals directly comparable and maximize sample size. Based primarily on the differences in photosynthetic discrimination against metabolism of ^13^CO_2_ in C3 versus C4 plants^109,112^, with limited variation due to local soil chemistry and aridity^113^, δ^13^C values track reliance on C3 versus C4 plant foods and animal protein sources reliant on such foods. In secondary consumers like humans, δ^15^N values are useful for revealing trophic level and protein intake^110,114,115^. With each step up a food web, δ^15^N fractionation increases by ~ 2–4‰^115^. Though overlapping δ^13^C values may exist between marine mammals, terrestrial plants, and terrestrial mammals, the combination of δ^13^C and δ^15^N typically enables discrimination between dietary sources^116^.

In the Central Andes, distinct isotopic signatures of key resources have proven useful in identifying variation in dietary reliance on maize (*Zea mays*) or other C4 plants^60,82,103^, marine mammals and fish^76^, camelids^104^, and potatoes (*Solanum sp.*) and quinoa (*Chenopodium quinoa*)^117^ among other resources. In general, maize in the Central Andes has δ^13^C signatures of ~ − 11 to − 12‰ and δ^15^N of ~ 4 to 8‰^85,117^. The high δ^13^C values enable maize to be distinguished from C3 plants which have values such as ~ − 26‰ for potatoes, ~ − 25‰ for quinoa, and ~ − 25‰ for beans (*Phaseolus sp.*)^85,117^. Similarly, the isotopic signatures of Central Andean camelids differ from those of marine resources in that marine resources possess both elevated δ^13^C (~ − 11 to − 16‰) and δ^15^N (~ 16 to 23‰) values ^76,77^, whereas camelids tend to be less elevated in δ^15^N (~ 5 to 8‰), though δ^13^C may vary^77,118^. For this analysis, we do not explore the specific inputs contributing to observed δ^13^C‰ and δ^15^N‰ values. Rather, we use the observed values as composite measures of variation in diet.

### Climatic Variables

We capture variation in local climates in space and time through a general circulation model simulation of mean annual precipitation (mm/day), mean annual temperature (°C), mean annual temperature seasonality (sd °C * 100), and mean annual precipitation seasonality (sd mm/day * 100) (Fig. 1). Pre-historic climatic variables are generated from the TRaCE-21 ka experiments^119–121^ via the PaleoView Paleoenvironmental Reconstruction Tool^122^. Through this tool, we obtain estimates for precipitation, temperature, and seasonality at 20-year intervals from 11,000 to 140 yBP over a 2.5 × 2.5 degree latitude/longitude grid, providing broad resolution variation in spatial and temporal estimates (Figs. 1, 2).

Climate variables are related to each individual through iterated, weighted sampling of the climatic values within an individual’s date range, using site coordinates to get the data from the individual’s spatial location. Individual date ranges are based upon reporting author assignations, converted into years before present (yBP), or, for individuals directly radiocarbon dated (n = 155, ~ 9% of the sample), the calibrated radiocarbon date range BP. To help account for potential bias driven by uncertainty in the age estimate of each individual, we conduct a Monte Carlo sampling routine to obtain climate estimates. Each climate variable’s values are extracted from each year within the maximum and minimum possible date for each individual. Each individual’s temporal range is then randomly sampled, with replacement, 10,000 times. For individuals who are radiocarbon dated, the probability of selecting a given year’s climate observation is weighted by the probability density of that year from the calibrated date. For individuals who are not radiocarbon dated, each year in their time window has equal probability of being selected at each sampling. We then average the 10,000 samples for each climatic variable per individual, providing an estimate of the most likely climatic conditions from their lifetime.

### Demography

Evaluation of archaeological population size/demography is difficult. Here we attempt to capture relative changes in population size by proxy through recreating demographic trends as the relative change in population estimate between points in time. To evaluate this variation, we employ the “dates as data” approach^29,40,123–125^. To generate the data for this approach we collate radiocarbon dates from existing compilations generated by Ziólkowski^126^, Gayo, et al.^55^, Rademaker, et al.^127^, Goldberg, et al.^128^, Riris^40^, and Roscoe, et al.^16^. From the compilation, we select all dates from Peru, northern Chile, and the Bolivian highlands, to align with regions from which we have isotope data. Dates are checked for duplication using the laboratory ID code and all duplicated dates are reduced to one observation. We employ several strategies for date quality control. 1) We remove dates older than 15,000 years as unlikely to be human generated (though our analysis relies on dates from the past 7000 years and is not influenced by this removal). 2) Following Riris^40^ and Robinson, et al.^129^, dates with errors greater than 200 years are also removed as such dates may be unreliable. 3) We drop any dates for which coordinates could not be identified. Dates from Roscoe, et al.^16^ are an exception and are kept even if missing coordinates as the compilation relied exclusively on coastal dates. The quality control process results in a set of 3957 dates from which the demographic data are generated.

In order to account for uncertainty in both population size estimates derived from radiocarbon dates and uncertainty in the date in which each individual died, we employ composite kernel density estimates (KDEs) of radiocarbon date demographic reconstruction^130–133^ and Monte Carlo iterated sampling. We generate population size estimates via KDEs within each of our elevation zones, allowing us to estimate population size trends separately for the coast, mid-elevations, and highlands. To classify dates by elevation, we take the coordinates for each date, extract elevation from the DEM, and then categorize the dates using the same elevation criterion as for individuals with isotope values. The resultant samples are 1304 (1115 < 7000 yBP) coastal dates, 1843 (1610 < 7000 yBP) mid-elevation dates, and 810 (657 < 7000 yBP) highland dates from which we generate the KDEs. KDE generation requires calibrated dates; all non-marine dates are calibrated using SHCal20. Marine-derived dates are calibrated using the Marine20 curve with reservoir effect offsets for each date calculated using calib.org^134^. Though implementation of the SHCal20 curve for all dates may introduce some errors given the potential for mixing Northern and Southern hemisphere climate dynamics, the magnitude of any such errors will be small and distributed across our sample^135^.

Following previous researchers using dates as data in the Central Andes^16,29,40^, we employ hierarchical clustering using a defined cutoff of 200 years to control for overrepresentation of individual sites^40,125,131^. To construct KDEs, we employ the **rcarbon** package^131^ in the R statistical environment^136^ to generate 1000 unique kernel density estimates through random sampling of calendar ages from each of the calibrated radiocarbon probability distributions, providing an estimate of the envelope of possible reconstructions. Demographic estimates for each individual are then constructed through iterated resampling via the following steps. First, we randomly select 1 of the 1000 KDE fits. Second, we subset that estimate by the individual’s time window, providing the possible KDE values from the full range of time when that person may have died. Third, we randomly select one of the years from that subset and retain the KDE value from that year. Fourth, these steps are repeated 10,000 times per individual. This provides 10,000 distinct KDE values for each individual representing the potential range of the size of the population that existed when they died. Sampling in this fashion helps capture the uncertainty in the demographic reconstruction as the 1000 KDE fits provide the envelope of possibilities and it helps capture the uncertainty in when individuals most likely died by randomly selecting only one year in each iteration. For individuals who are radiocarbon dated, the same process is used as described above except that, when it comes to selecting an individual year to sample the KDE value from, the probability a year is selected is weighted by the probability density from the individual’s calibrated radiocarbon date. This weights the selection of values toward higher probability years. Central tendency population size values are then assigned to each individual by taking the average of each individual’s 10,000 samples. The result is a proxy of population size per zone during the lifespan of each individual, capturing uncertainty and weighted toward the years in which the individual most likely died.

Prior dates as data research has suggested that taphonomic loss of older radiocarbon dateable material has the potential to impact demographic reconstructions using radiocarbon dates^137^. In the Central Andes, scholars have decided to employ taphonomic correction (or not) based upon research question and sampling area^16,29,40,55^ and have suggested that while corrected and uncorrected datasets are generally in agreement, some differences can occur^55^. To address uncertainty as to whether corrected or uncorrected reconstructions are more appropriate, the complete analysis here is run twice, once each for uncorrected and corrected demographic reconstructions. To generate the corrected reconstructions, we apply the global taphonomic correction equation from Surovell et al.^137^ to each of the 1000 KDE fits and then implement the same sampling steps listed above for each individual. This provides both a corrected and uncorrected demographic estimate based on 10,000 iterated samplings for each individual. For our analysis, these relative measures of population enable us to evaluate if variation in population size is an influential factor for predicting diet.

### Statistical analyses

All analyses are conducted in the R statistical environment^136^, with complete code to replicate the analyses available in Supplementary Material 1.

We visualize δ^15^N and δ^13^C trends across each of the elevation categories over time using iterated generalized additive model (GAM) fits. GAMs estimate non-linear fits between response and predictor variables using splines, with smoothing parameters estimated using generalized cross validation^138,139^. We construct GAMs for each elevation zone for both δ^15^N and δ^13^C, predicting the isotope value with an individual’s date. To address uncertainty in when individual’s date to, we construct a series of 10,000 GAMs for each of our six elevation and isotope combinations (60,000 total). First, we take each individual and resample their date window 10,000 times, obtaining 10,000 individual year estimates per person, with each estimate representing a unique run. For radiocarbon dated individuals, we weight the probability of selecting a year from their calibrated date window based upon the probability density from the calibration. We then subset the data to obtain datasets of individuals for coastal, mid-elevation, and highland δ^15^N and do the same for δ^13^C, with each individual possessing 10,000 dates. For each combination of elevation zone and isotope, we fit one GAM per unique run (10,000 runs), so each individual has a single year estimate in each of the GAMs. Each unique GAM fit is used to predict the isotope value over the study period. We then aggregate the 10,000 GAMs per elevation zone and isotope, generating a mean predicted value, mean standard error, and mean standard deviation per temporal observation. These data are used to fit a central tendency line with 95% confidence intervals for each combination (Fig. 3).

We also visualize the trends over time using 95% confidence interval ellipses plots broken out by time period, elevation zone, and isotope (Supplementary Fig. 1 and Supplementary Material 2) using the **SIBER** package^140^ in R.

We assess the correlation of climate and relative population size with diet in the Central Andes empirically using random forest (RF)^141^ regression via the **ranger** package^142^ in R. R code for replicating the complete analysis is provided in Supplementary Material 1. RF regression is a machine learning method that employs an ensemble approach to generate mean prediction of a dependent variable when relationships may be non-linear and the number of interactions between variables may be large. RFs subsample predictor variables to prevent over-reliance on any single predictor and control for complex interaction between variables, allowing us to parse out the influence of climatic and demographic variables separately. Here, four regression models are generated, one each for δ^13^C‰ and δ^15^N‰ using taphonomically uncorrected or corrected demographic estimates. For each set of individuals with δ^15^N‰ and those with δ^13^C‰, we assess the collinearity of predictor variables by calculating the strength of correlation between pairwise comparisons. Variables with correlation strengths less than 0.70^143^ are deemed viable for inclusion in the models, but any greater than 0.70 present potential co-variance problems, although RF analysis can handle such highly correlated variables^141^. For both the δ^13^C‰ and δ^15^N‰ sets of individuals, all variables are under the 0.70 correlation threshold and we therefore include all variables in the models.

RF models are evaluated with prediction errors and the standard deviation of residuals from tenfold model cross-validation using the **spm** package^144^. We then check for temporal and spatial autocorrelation in the model residuals using acf plots and evaluating the expected vs observed Moran’s I values on an inverse distance matrix calculated using the **ape** package^145^ in R.

To estimate the influence of climate versus population size, we calculate the effect size of each covariate. As random forest fits non-linear responses, we use the centered partial dependency function to estimate effect size. In the absence of interactions between variables, the overall predicted response ($F\left( X \right)$) is the sum of individual partial dependencies for each covariate^146^. We therefore define effect size for variable $j$ as:

$${\text{Effect}}\left( {x_{j} } \right) = \sqrt {\mathop \sum \limits_{i = 0}^{n} \hat{F}\left( {x_{j} } \right)}$$

where $\hat{F}\left( {x_{j} } \right)$ is the centered partial dependency for $j$. A proportional effect size can then be calculated as the ratio of this value to the overall predicted response.

As there are interactions between our variables, we calculate Friedman’s $H$ statistic, or the proportion of each variable’s effect resulting from its interaction with the other variables^146^ within each elevation category:

$$H = \sqrt {\mathop \sum \limits_{i = 0}^{n} F\left( X \right) - \hat{F}\left( {x_{j} } \right) - \hat{F}\left( {x_{ - j} } \right)} /\sqrt {\mathop \sum \limits_{i = 0}^{n} F\left( X \right)}$$

Calculation of this statistic is done using the **iml** package in R^147^. Friedman’s *H* statistic allows us, for each variable, to deduct the proportion of the effect due to the interaction with other variables to get the independent effect size of each predictor variable.

Effect sizes and *H* statistic values are estimated through 100 iterated simulations wherein, for each iteration, we randomly sample 100 values of variable *j* and assign those values to each individual in the dataset. This results in 100 copies of each individual where the difference is the value of variable *j*. We then predict the isotopic value for each individual using the RF model. Using the resultant output, we calculate the total response of the isotope (δ^13^C‰ or δ^15^N‰), the partial dependence of the isotope to the chosen predictor variable, the combined partial dependence to all other predictor variables, Friedman’s *H statistic*, explained sum of squares, explained sum of squares deducted by Friedman’s *H statistic*, and the explained sum of squares as a percentage of the total variability in the isotope for the chosen variable. The results of each of the 100 iterations are then averaged together to provide an estimate of the effect size of each variable within each elevation category. We take the square root of the effect sizes to return the values into per mil (‰) space. We then divide the square root of the explained sum of square effect sizes by the total variability to obtain the percent of the variability explained by each of our predictor variables, enabling comparison within models. We compare cumulative climate with demographic effects to evaluate the relative influence of climate versus population size on dietary variation. This comparison removes the influence of the interactions between variables to enable evaluation of the independent effects.

### Data limitations

Our isotope dataset is the largest compiled in the Americas and we are confident in its usefulness for characterizing differentiation in individiuals’ diets over time in the Central Andes. However, as with all data, there are several limitations. First, the isotope record is not a random sample and possesses some unevenness in its spatial and temporal distribution (Supplementary Figs. S2 & S3). Numerous individuals represent the past approximately 2000 yBP (Supplementary Figs. S2, S3) on the coasts and mid-elevations, but fewer predate this point in time or are from the highlands. This makes us more confident in the coastal, mid-elevation, and post-2000 yBP patterns than the pre-2000 yBP and highland patterns. Further, we rely on broadly local estimates of climate and population, which might not represent the circumstances experienced by highly mobile individuals e.g.,^148^. Finally, δ^15^N and δ^13^C capture broad dietary differences between resources such as marine and camelid protein or maize (C4) and potatoes (C3). Changes in diet that are not shifts between C3 and C4, such as shifting from one C3 resource to another C3 resource, in response to climatic or population change, may not be reflected in this data. Future work combining additional aspects of diet such as isotopic signatures of local food items by site, primary and secondary dietary inputs from zooarchaeological and palaeobotanical analyses, adding additional highland or early individuals, and incorporating additional isotopes such as δ^18^O and δ^34^S or δ^13^C from enamel or bone apatite may reveal unseen patterns and better enable evaluation of what direct food items contribute to changes. The data also are potentially limited by a lack of local isotope baselines for all samples for generating corrections, uncontrolled impacts from possible fertilization, and by implementation of different foddering practices for camelids, even within sites^80,85–87^. Each of these limitations highlights potentially productive avenues for future research.

Additionally, our climate data are spatially coarse-grained simulations (2.5 × 2.5° lat/long) which do not capture highly localized variation in climatic patterns. However, the Trace21ka simulation provides complete coverage in time and space across the study area. Attempting to instead rely on proxy records would severely limit the spatial and temporal variation we employ as available proxy records are inconsistent in the temporal periods they cover and would provide less spatial resolution. Given the spatial distribution of the individuals included in this analysis, the Trace21ka simulations provide spatial and temporal variation (Figs. 1, 2) across the geographic and temporal spread of the entire study area. The data we use provide a conservative estimate of the influence of climate and population given the spatial variation they do average over. Future work employing downscaling of GCM variables and/or generating and using more regional paleoclimate proxy data may improve the accuracy of the analyzed relationships.

Finally, using dates as data employs a range of assumptions which may impact the accuracy of demographic trend recreations and KDEs, like summed probability distributions, are flawed proxies for past population estimates^149^. To address some of the flaws, researchers employing this approach have proposed applying taphonomic corrections^137^, binning dates to control for the influence of well-dated sites^125,131^, and separating regions and/or time periods with significantly different human behaviors for analysis^123^. As it is unclear whether taphonomically correcting KDEs is more or less accurate than leaving them uncorrected in this case, we run the analysis twice, once for each the corrected and uncorrected data. To deal with sampling biases, we ‘bin’ the dates using hierarchical clustering with a 200 year cut-off value and we separate KDEs into elevation zones to attempt to compare KDE fluctuations within contexts likely sharing similar behaviors. We attempt to deal with the assumptions in the best way possible, though violations of them will lower the correlative and interpretive power of our demographic variable. Despite these limitations, KDEs are useful in this case as they provide an estimate of the relative variation in population size and our analysis is concerned with such relative, rather than absolute, differences. While our proxies for both climate/environment and demography are imperfect, they remain useful for assessing the relative influence of these two factors on diet.

## Supplementary Information

Supplementary Information 1.

Supplementary Information 2.

Supplementary Information 3.

Supplementary Information 4.

Supplementary Legends.

## Data Availability

All data needed to evaluate the conclusions in the paper are present in the paper and/or Supplementary materials.
