# Supplementary material for: Climate and demography drive 7000 years of dietary change in the Central Andes
Source: Sci Rep. 2022 Feb 7;12:2026. doi: 10.1038/s41598-022-05774-y (PMC8821598; doi:10.1038/s41598-022-05774-y)
Supplement: Supplementary file 3 — Supplementary Information 3. [file 41598_2022_5774_MOESM3_ESM.pdf]

Supplemental Material 1

Wilson, Kurt M., McCool, Weston C., Brewer, Simon C., Zamora-Wilson, Nicole, Schryver, Percy J., Lamson, Roxanne Lois F., Huggard, Ashlyn M., Brenner Coltrain, Joan, Contreras, Daniel A., Codding, Brian F.

11/22/2021

This supplemental material is from “Climate and demogrpahy drive 7,000 years of dietary change in the Central Andes”, submitted to *Scientific Reports*. The corresponding author, Kurt M. Wilson, may be contacted at kurt.wilson@utah.edu (mailto:kurt.wilson@utah.edu).The code below enables complete rerunning of the analysis.

Note: pages 155 - 159 provide source code for running effect size calculations that will need to be saved as its own R code file in the same directory as the rest of this supplement.

Load Required Packages

Read in the R packages used in this analysis

```
#load packages

#working with the data tables and files
suppressPackageStartupMessages(library(plyr))
suppressPackageStartupMessages(library(dplyr))
suppressPackageStartupMessages(library(tidyr))
suppressPackageStartupMessages(library(stringr))
suppressPackageStartupMessages(library(stringi))
suppressPackageStartupMessages(library(tidyverse))

#exporting as an html file
suppressPackageStartupMessages(library(knitr))

#Analysis packages
suppressPackageStartupMessages(library(corrplot))#used to plot correlation matrices
suppressPackageStartupMessages(library(ranger))#random forest regression
suppressPackageStartupMessages(library(iml))#package used to calculate the effect sizes,
including Friedman's h-stat
suppressPackageStartupMessages(library(spm))#cross-validation on ranger RF
suppressPackageStartupMessages(library(phenocamr))#package for calculating optimal span
 using BIC
suppressPackageStartupMessages(library(ape))#use for calculating Moran's I of the residu
als to test for spatial autocorrelation
suppressPackageStartupMessages(library(sf))#use for setting up data for cross-validation

#Plotting
suppressPackageStartupMessages(library(DescTools)) #plotting Loess regression lines
suppressPackageStartupMessages(library(viridis))#color-blind safe plotting colors
suppressPackageStartupMessages(library(funModeling))#used in creation of correlation mat
rix and plotting
suppressPackageStartupMessages(library(SIBER))#used to generate ellipses plots

source("mg_functions.R")#Code for calculating the effect sizes - written by Simon Brewe
r.
```

Prepare the Data

First, load the analysis data:

```
dat <- read.csv("Supplemental_Data_File_2.csv", na.strings=c("","NA")) names(dat)
```

```
##  [1] "X.1"                          "X"
##  [3] "Site.ID"                      "Site"
##  [5] "Location"                     "Region"
##  [7] "Period"                       "Start.BP.site"
##  [9] "End.BP.site"                  "Start.BP"
## [11] "End.BP"                       "C14.Date"
## [13] "C14..."                       "Start.BP.14c"
## [15] "End.BP.14c"                   "Latitude"
## [17] "Longitude"                    "Study.ID"
## [19] "Individual.ID"                "Sample.ID"
## [21] "Averaged"                     "Sex"
## [23] "Age"                          "Age_Cat"
## [25] "C.b.coll."                    "N.b.coll."
## [27] "C.N..bone."                   "CN.accept"
## [29] "X..collagen.yield..bone."     "elevation"
## [31] "dist2coast"                   "d2coast_km"
## [33] "elev.factor"                  "Demography.spd"
## [35] "Demography.spd.corr"          "Temp_Mean"
## [37] "Precip_Mean"                  "Temp_Mean_Seasonality"
## [39] "Precip_Mean_Seasonality"      "Temp_Mean.sd"
## [41] "Precip_Mean.sd"               "Temp_Mean_Seasonality.sd"
## [43] "Precip_Mean_Seasonality.sd"   "Demography.kde.14c"
## [45] "Demography.kde.corr.14c"      "Demography.kde.14c.sd"
## [47] "Demography.kde.corr.14c.sd"   "Citations"
## [49] "median.year"
```

Next, create elevation zone categories and check to be sure the variable is a factor level for analytical purposes. We use an adapted version of Pulgar Vidal’s (1981) (17) natural zones for these categories. Coastal individuals are anyone recovered <15km from the coast and <350 meters above sea level (masl). Mid-elevation individuals are those recovered from <350 masl but >15km from the coast or >350 masl but <3500 masl. Highland individuals are anyone recovered from >3500 masl.

```
#Follow the code below to recreate the elevation zones used in the analysis. These are a
lready in the csv file, so this code is for replication purposes.

#dat$elev.factor <- "Coastal" #set all individuals to be coastal - individuals who are l
ower than 350 masl and less than 15km from the coast should remain coastal
#dat$elev.factor[dat$elevation < 350 & dat$d2coast_km > 15] <- "Mid-elevation" #Individu
als who are lower than 350 masl but greater than 15km from the coast should be mid-eleva
tion
#dat$elev.factor[dat$elevation > 350] <- "Mid-elevation" #Individuals above 350 masl and
below 3500 masl should also be mid-elevation
#dat$elev.factor[dat$elevation > 3500] <- "Highland" #Individuals above 3500 masl should
be highland

dat$elev.factor <- as.factor(dat$elev.factor)#set elev.factor as a factor level

is.factor(dat$elev.factor)#Check to make sure the elev.factor column is a factor level v
ariable
```

```
## [1] TRUE
```

Before breaking the data apart into individuals with bone collagen δ^{15}N‰ and those with bone collagen δ^{13}C‰, we visualize the δ^{15}N‰ and δ^{13}C‰ ellipses over time. This is purely a visual representation, but provides an overview of the level of difference over time. We plot the 95% CI ellipses for each of the elevation categories as well as broken into time periods.

```
dat.both <- dat[which(dat$N.b.coll. != "NA" & dat$C.b.coll. != "NA"),] #subset to get on
ly the individuals who have both carbon and nitrogen (1731 individuals)

#put data into format usable by the Siber package for drawing ellipses. We need to have
 isotope 1, isotope 2, a group, and a community. Columns must be named iso1, iso2, grou
p, and community. Here we want to do iso1 as carbon, iso2 as nitrogen, group as the elev
ation factor, and community as the time period.
dat.siber <- dat.both[,c("C.b.coll.", "N.b.coll.","elev.factor","Period")]#grab appropri
ate columns
colnames(dat.siber) <- c("iso1", "iso2", "group","community")#rename to fit SIBER packag
e



#Subset to periods with enough observations for SIBER plotting (these are Preceramic, Ea
rly Horizon, Early Intermediate Period, Middle Horizon, Late Intermediate Period, and La
te Horizon. We do not have enough unique individuals for each elevation category for the
initial period.)

dat.siber.periods <- dat.siber[which(dat.siber$community == "Preceramic" | dat.siber$com
munity == "Early Horizon" | dat.siber$community == "Early Intermediate Period" | dat.sib
er$community == "Middle Horizon" | dat.siber$community == "Late Intermediate Period" | d
at.siber$community == "Late Horizon"),]#takes us to 1671 individuals

#subset even further, by the time periods to enable easier plotting
siber.pre <- dat.siber.periods[which(dat.siber.periods$community == "Preceramic"),]
siber.pre$community <- as.factor(siber.pre$community)

siber.eh <- dat.siber.periods[which(dat.siber.periods$community == "Early Horizon"),]
siber.eh$community <- as.factor(siber.eh$community)

siber.eip <- dat.siber.periods[which(dat.siber.periods$community == "Early Intermediate
 Period"),]
siber.eip$community <- as.factor(siber.eip$community)

siber.mh <- dat.siber.periods[which(dat.siber.periods$community == "Middle Horizon"),]
siber.mh$community <- as.factor(siber.mh$community)

siber.lip <- dat.siber.periods[which(dat.siber.periods$community == "Late Intermediate P
eriod"),]
siber.lip$community <- as.factor(siber.lip$community)

siber.lh <- dat.siber.periods[which(dat.siber.periods$community == "Late Horizon"),]
siber.lh$community <- as.factor(siber.lh$community)
```

Now that we have the data, we want to put it into a format that the SIBER package can work with, a Siber object.

```
#create the SIBER objects
siber.pre <- createSiberObject(siber.pre)
#reorder the levels so they go Coastal, Mid-elevation, Highland
siber.pre$original.data$group <- factor(siber.pre$original.data$group, levels = c("Coastal", "Mid-elevation", "Highland"))
siber.eh <- createSiberObject(siber.eh)
#reorder the levels so they go Coastal, Mid-elevation, Highland
siber.eh$original.data$group <- factor(siber.eh$original.data$group, levels = c("Coastal", "Mid-elevation", "Highland"))
siber.eip <- createSiberObject(siber.eip)
#reorder the levels so they go Coastal, Mid-elevation, Highland
siber.eip$original.data$group <- factor(siber.eip$original.data$group, levels = c("Coastal", "Mid-elevation", "Highland"))
siber.mh <- createSiberObject(siber.mh)
#reorder the levels so they go Coastal, Mid-elevation, Highland
siber.mh$original.data$group <- factor(siber.mh$original.data$group, levels = c("Coastal", "Mid-elevation", "Highland"))
siber.lip <- createSiberObject(siber.lip)
#reorder the levels so they go Coastal, Mid-elevation, Highland
siber.lip$original.data$group <- factor(siber.lip$original.data$group, levels = c("Coastal", "Mid-elevation", "Highland"))
siber.lh <- createSiberObject(siber.lh)
#reorder the levels so they go Coastal, Mid-elevation, Highland
siber.lh$original.data$group <- factor(siber.lh$original.data$group, levels = c("Coastal", "Mid-elevation", "Highland"))
```

Make some transparent colors to use for the points.

```
coast.alpha <- rgb(68,1,84, max = 255, alpha = 60)
midel.alpha <- rgb(33,144,140, max = 255, alpha = 60)
highl.alpha <- rgb(253,231,37, max = 255, alpha = 90)
```

Plot the C and N values with 95% confidence interval ellipses around them for each elevation category by time period.

```
#png("Siber_C_N_plots.png", width = 5, height = 3.5, units = 'in', res = 300)
par(pty = "s", mfrow = c(2,3),oma = c(4,4,1,1),mar = c(1,1,1,1), xpd = NA)
#Preceramic
#par(mar=c(0.5,1.5,1.5,1.5))
plot(siber.pre$original.data$iso1, siber.pre$original.data$iso2, ylim = c(0,35), xlim =
 c(-25,-5), pch = 19, col = c(coast.alpha, midel.alpha, highl.alpha)[siber.pre$original.
data$group], xlab = "", ylab = "", xaxt = "na")
coords <- addEllipse(siber.pre$ML.mu[[1]][ , , 1],
                     siber.pre$ML.cov[[1]][ , , 1],
                     m = NULL,
                     n = 100,
                     p.interval = 0.95,
                     ci.mean = FALSE,
                     col = "#440154FF",
                     lty = 1,
                     lwd = 2)
coords <- addEllipse(siber.pre$ML.mu[[1]][ , , 2],
                     siber.pre$ML.cov[[1]][ , , 2],
                     m = NULL,
                     n = 100,
                     p.interval = 0.95,
                     ci.mean = FALSE,
                     col = "#21908CFF",
                     lty = 1,
                     lwd = 2)
coords <- addEllipse(siber.pre$ML.mu[[1]][ , , 3],
                     siber.pre$ML.cov[[1]][ , , 3],
                     m = NULL,
                     n = 100,
                     p.interval = 0.95,
                     ci.mean = FALSE,
                     col = "#FDE725FF",
                     lty = 1,
                     lwd = 2)
text(-25, 0.5, labels = "Preceramic", pos = 4, cex = 0.75)
mtext(text = expression({delta}^15*N~'\u2030'), side = 2, line = 3, at = -3, cex = 0.75)
legend("topleft", bty="n", col=c("#440154FF", "#21908CFF", "#FDE725FF"), pch = 19, legen
d=c("Coastal", "Mid-elevation", "Highland"), cex = 0.75)

#par(mar=c(0.5,1.5,1.5,1.5))
plot(siber.eh$original.data$iso1, siber.eh$original.data$iso2, ylim = c(0,35), xlim = c
(-25,-5), pch = 19, col = c(coast.alpha, midel.alpha, highl.alpha)[siber.eh$original.dat
a$group], xlab = "", ylab = "", xaxt = "na", yaxt = "na")
coords <- addEllipse(siber.eh$ML.mu[[1]][ , , 1],
                     siber.eh$ML.cov[[1]][ , , 1],
                     m = NULL,
                     n = 100,
                     p.interval = 0.95,
                     ci.mean = FALSE,
                     col = "#440154FF",
                     lty = 1,
                     lwd = 2)
```

```
coords <- addEllipse(siber.eh$ML.mu[[1]][ , , 2],
                     siber.eh$ML.cov[[1]][ , , 2],
                     m = NULL,
                     n = 100,
                     p.interval = 0.95,
                     ci.mean = FALSE,
                     col = "#21908CFF",
                     lty = 1,
                     lwd = 2)
coords <- addEllipse(siber.eh$ML.mu[[1]][ , , 3],
                     siber.eh$ML.cov[[1]][ , , 3],
                     m = NULL,
                     n = 100,
                     p.interval = 0.95,
                     ci.mean = FALSE,
                     col = "#FDE725FF",
                     lty = 1,
                     lwd = 2)
text(-25, 0.5, labels = "Early Horizon", pos = 4, cex = 0.75)

#par(mar=c(0.5,1.5,1.5,1.5))
plot(siber.eip$original.data$iso1, siber.eip$original.data$iso2, ylim = c(0,35), xlim =
 c(-25,-5), pch = 19, col = c(coast.alpha, midel.alpha, highl.alpha)[siber.eip$original.
data$group], xlab = "", ylab = "", xaxt = "na", yaxt = "na")
coords <- addEllipse(siber.eip$ML.mu[[1]][ , , 1],
                     siber.eip$ML.cov[[1]][ , , 1],
                     m = NULL,
                     n = 100,
                     p.interval = 0.95,
                     ci.mean = FALSE,
                     col = "#440154FF",
                     lty = 1,
                     lwd = 2)
coords <- addEllipse(siber.eip$ML.mu[[1]][ , , 2],
                     siber.eip$ML.cov[[1]][ , , 2],
                     m = NULL,
                     n = 100,
                     p.interval = 0.95,
                     ci.mean = FALSE,
                     col = "#21908CFF",
                     lty = 1,
                     lwd = 2)
coords <- addEllipse(siber.eip$ML.mu[[1]][ , , 3],
                     siber.eip$ML.cov[[1]][ , , 3],
                     m = NULL,
                     n = 100,
                     p.interval = 0.95,
                     ci.mean = FALSE,
                     col = "#FDE725FF",
                     lty = 1,
                     lwd = 2)
text(-25, 0.5, labels = "Early Intermeidate Period", pos = 4, cex = 0.75)
```

```
#par(mar=c(1.5,1.5,0.5,1.5))
plot(siber.mh$original.data$iso1, siber.mh$original.data$iso2, ylim = c(0,35), xlim = c
(-25,-5), pch = 19, col = c(coast.alpha, midel.alpha, highl.alpha)[siber.mh$original.dat
a$group], xlab = "", ylab = "")
coords <- addEllipse(siber.mh$ML.mu[[1]][ , , 1],
                     siber.mh$ML.cov[[1]][ , , 1],
                     m = NULL,
                     n = 100,
                     p.interval = 0.95,
                     ci.mean = FALSE,
                     col = "#440154FF",
                     lty = 1,
                     lwd = 2)
coords <- addEllipse(siber.mh$ML.mu[[1]][ , , 2],
                     siber.mh$ML.cov[[1]][ , , 2],
                     m = NULL,
                     n = 100,
                     p.interval = 0.95,
                     ci.mean = FALSE,
                     col = "#21908CFF",
                     lty = 1,
                     lwd = 2)
coords <- addEllipse(siber.mh$ML.mu[[1]][ , , 3],
                     siber.mh$ML.cov[[1]][ , , 3],
                     m = NULL,
                     n = 100,
                     p.interval = 0.95,
                     ci.mean = FALSE,
                     col = "#FDE725FF",
                     lty = 1,
                     lwd = 2)
text(-25, 0.5, labels = "Middle Horizon", pos = 4, cex = 0.75)

#par(mar=c(1.5,1.5,0.5,1.5))
plot(siber.lip$original.data$iso1, siber.lip$original.data$iso2, ylim = c(0,35), xlim =
 c(-25,-5), pch = 19, col = c(coast.alpha, midel.alpha, highl.alpha)[siber.lip$original.
data$group], xlab = expression({delta}^13*C~'\u2030'), ylab = "", yaxt = "na")
coords <- addEllipse(siber.lip$ML.mu[[1]][ , , 1],
                     siber.lip$ML.cov[[1]][ , , 1],
                     m = NULL,
                     n = 100,
                     p.interval = 0.95,
                     ci.mean = FALSE,
                     col = "#440154FF",
                     lty = 1,
                     lwd = 2)
coords <- addEllipse(siber.lip$ML.mu[[1]][ , , 2],
                     siber.lip$ML.cov[[1]][ , , 2],
                     m = NULL,
                     n = 100,
                     p.interval = 0.95,
```

```
                     ci.mean = FALSE,
                     col = "#21908CFF",
                     lty = 1,
                     lwd = 2)
coords <- addEllipse(siber.lip$ML.mu[[1]][ , , 3],
                     siber.lip$ML.cov[[1]][ , , 3],
                     m = NULL,
                     n = 100,
                     p.interval = 0.95,
                     ci.mean = FALSE,
                     col = "#FDE725FF",
                     lty = 1,
                     lwd = 2)
text(-25, 0.5, labels = "Late Intermediate Period", pos = 4, cex = 0.75)


#par(mar=c(1.5,1.5,0.5,1.5))
plot(siber.lh$original.data$iso1, siber.lh$original.data$iso2, ylim = c(0,35), xlim = c
(-25,-5), pch = 19, col = c(coast.alpha, midel.alpha, highl.alpha)[siber.lh$original.dat
a$group], xlab = "", ylab = "", yaxt = "na")

coords <- addEllipse(siber.lh$ML.mu[[1]][ , , 1],
                     siber.lh$ML.cov[[1]][ , , 1],
                     m = NULL,
                     n = 100,
                     p.interval = 0.95,
                     ci.mean = FALSE,
                     col = "#440154FF",
                     lty = 1,
                     lwd = 2)
coords <- addEllipse(siber.lh$ML.mu[[1]][ , , 2],
                     siber.lh$ML.cov[[1]][ , , 2],
                     m = NULL,
                     n = 100,
                     p.interval = 0.95,
                     ci.mean = FALSE,
                     col = "#21908CFF",
                     lty = 1,
                     lwd = 2)
coords <- addEllipse(siber.lh$ML.mu[[1]][ , , 3],
                     siber.lh$ML.cov[[1]][ , , 3],
                     m = NULL,
                     n = 100,
                     p.interval = 0.95,
                     ci.mean = FALSE,
                     col = "#FDE725FF",
                     lty = 1,
                     lwd = 2)
text(-25, 0.5, labels = "Late Horizon", pos = 4, cex = 0.75)
```

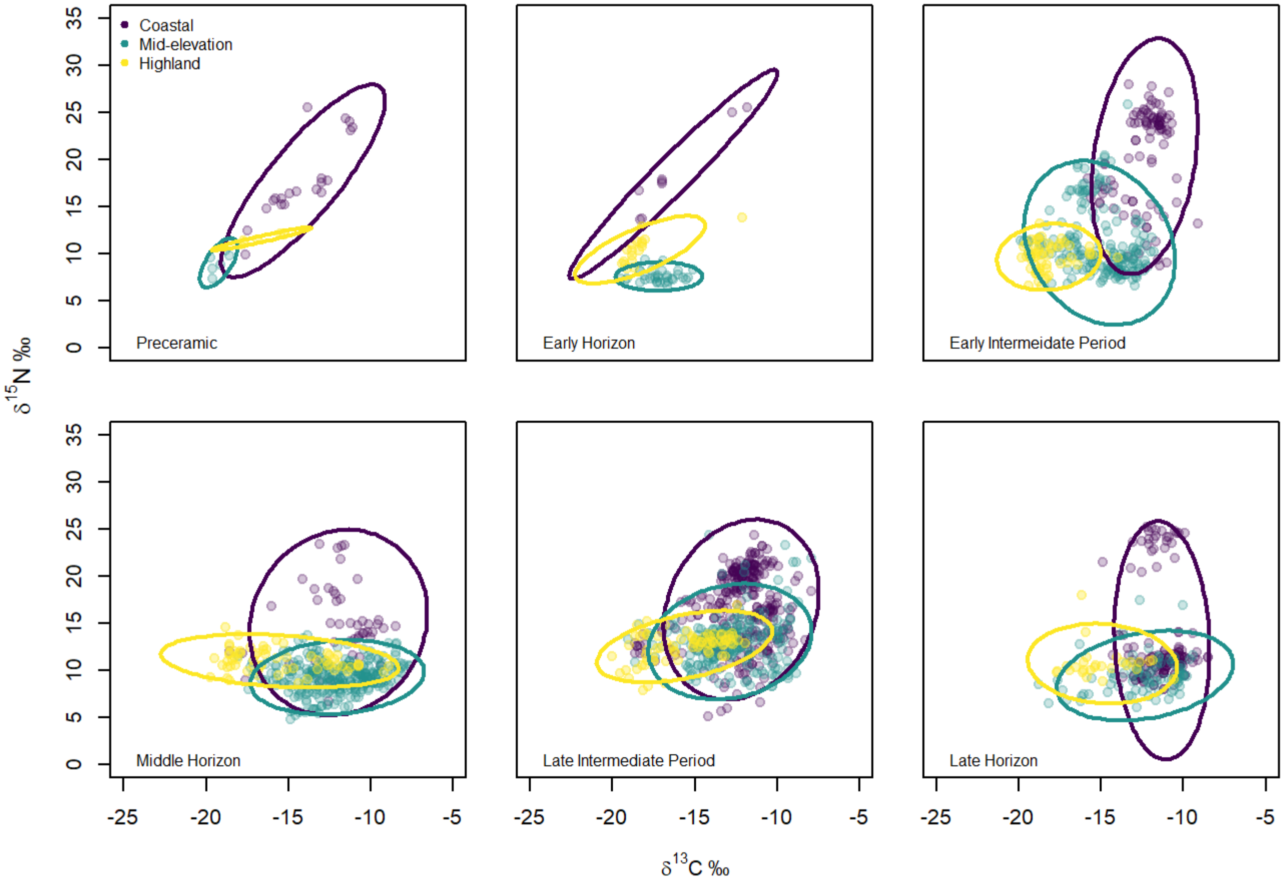


```
#dev.off()
```

Fig. S1. δ^{15}N‰ and δ^{13}C‰ 95% Ellipses Plots by Time Period. Ellipses show some general changes over time. Beginning around the Early Intermediate Period there appears to be a shift toward higher δ^{13}C‰ dietary items, particularly on the coasts. During the Middle Horizon these shifts appear to continue, particularly in the mid-elevations, and all diets remain relatively high in δ^{13}C‰ through the Late Horizon. Coastal diets are always elevated in δ^{15}N‰ relative to others, most likely reflecting heavier reliance on marine resources. There appears to be increases in mid-elevation individuals' dietary δ^{15}N‰ during Intermediate periods relative to Horizons.

For visualization of the change over time, we animate the ellipses plots as a gif as well.

```
library(animation)

#Create a list of the carbon and nitrogen isotope data for each period
iso.pts <- list(siber.pre$original.data$iso1, siber.pre$original.data$iso2, siber.eh$original.data$iso1, siber.eh$original.data$iso2, siber.eip$original.data$iso1, siber.eip$original.data$iso2, siber.mh$original.data$iso1, siber.mh$original.data$iso2, siber.lip$original.data$iso1, siber.lip$original.data$iso2, siber.lh$original.data$iso1, siber.lh$original.data$iso2)

#Create a list of the data for generating the ellipses from the Siber object
siber.data.all <- list(siber.pre$ML.mu[[1]][ , , 1], siber.pre$ML.cov[[1]][ , , 1],siber.pre$ML.mu[[1]][ , , 2], siber.pre$ML.cov[[1]][ , , 2],siber.pre$ML.mu[[1]][ , , 3], siber.pre$ML.cov[[1]][ , , 3], siber.eh$ML.mu[[1]][ , , 1], siber.eh$ML.cov[[1]][ , , 1],siber.eh$ML.mu[[1]][ , , 2], siber.eh$ML.cov[[1]][ , , 2],siber.eh$ML.mu[[1]][ , , 3], siber.eh$ML.cov[[1]][ , , 3], siber.eip$ML.mu[[1]][ , , 1], siber.eip$ML.cov[[1]][ , , 1], siber.eip$ML.mu[[1]][ , , 2], siber.eip$ML.cov[[1]][ , , 2],siber.eip$ML.mu[[1]][ , , 3], siber.eip$ML.cov[[1]][ , , 3], siber.mh$ML.mu[[1]][ , , 1], siber.mh$ML.cov[[1]][ , , 1],siber.mh$ML.mu[[1]][ , , 2], siber.mh$ML.cov[[1]][ , , 2],siber.mh$ML.mu[[1]][ , , 3], siber.mh$ML.cov[[1]][ , , 3], siber.lip$ML.mu[[1]][ , , 1], siber.lip$ML.cov[[1]][ , , 1],siber.lip$ML.mu[[1]][ , , 2], siber.lip$ML.cov[[1]][ , , 2],siber.lip$ML.mu[[1]][ , , 3], siber.lip$ML.cov[[1]][ , , 3], siber.lh$ML.mu[[1]][ , , 1], siber.lh$ML.cov[[1]][ , , 1],siber.lh$ML.mu[[1]][ , , 2], siber.lh$ML.cov[[1]][ , , 2],siber.lh$ML.mu[[1]][ , , 3], siber.lh$ML.cov[[1]][ , , 3])

#Create a list of the elevation factor for each individual
fctr.lvls.siber <- list(siber.pre$original.data$group, siber.eh$original.data$group, siber.eip$original.data$group, siber.mh$original.data$group, siber.lip$original.data$group, siber.lh$original.data$group)

#create a list of the periods
period.labels <- c("Preceramic", "Early Horizon", "Early Intermediate Period", "Middle Horizon", "Late Intermediate Period", "Late Horizon")

#Generate a for loop to plot each of the figures. By removing the # in front of saveGIF and the final } at the bottom of the code an animated gif of the changing ellipses over time will be generated

saveGIF({
for(i in 0:5) {
  par(pty = "s",oma = c(4,4,1,1),mar = c(1,1,1,1), xpd = NA)
  plot(iso.pts[[i + i + 1]], iso.pts[[i + i + 2]], ylim = c(0,35), xlim = c(-25,-5), pch = 19, col = c(coast.alpha, midel.alpha, highl.alpha)[fctr.lvls.siber[[i + 1]]], xlab = expression({delta}^13*C~'\u2030'), ylab = expression({delta}^15*N~'\u2030'))
  coords <- addEllipse(siber.data.all[[i * 6 + 1]],
                       siber.data.all[[i * 6 + 2]],
                       m = NULL,
                       n = 100,
                       p.interval = 0.95,
                       ci.mean = FALSE,
                       col = "#440154FF",
                       lty = 1,
                       lwd = 2)
```

```
 coords <- addEllipse(siber.data.all[[i * 6 + 3]],
                     siber.data.all[[i * 6 + 4]],
                     m = NULL,
                     n = 100,
                     p.interval = 0.95,
                     ci.mean = FALSE,
                     col = "#21908CFF",
                     lty = 1,
                     lwd = 2)
 coords <- addEllipse(siber.data.all[[i * 6 + 5]],
                     siber.data.all[[i * 6 + 6]],
                     m = NULL,
                     n = 100,
                     p.interval = 0.95,
                     ci.mean = FALSE,
                     col = "#FDE725FF",
                     lty = 1,
                     lwd = 2)
 text(-25, 0.5, labels = period.labels[i + 1], pos = 4)
 legend("topleft", bty="n", col=c("#440154FF", "#21908CFF", "#FDE725FF"), pch = 19,    l
egend=c("Coastal", "Mid-elevation", "Highland"))
}
}, interval = 2,movie.name = "Isotope_period_ellipses.gif")
```

To help more formally visualize the pattern in diets over time while controlling for uncertainty in individuals' dates, we also construct a series of six 10,000 Monte Carlo style simulated generalized additive models to show how the general trend in diet changes over time within each of the elevation zones.

First, we generate iterated resampling of individual's potential dates 10,000 times. This process will a) extract each individual's time window, then b) randomly sample 1 year out of that window which, c) gets stored in a dataframe. This process is repeated 10,000 times for each individual. For the subset of individuals in our database who are directly radiocarbon dated, this process is slightly different: a) calibrate their radiocarbon date using the the southern hemisphere 2020 calibration curve, b) extract the calibrated probability density for each year, c) extract the years each probability density is associated with, d) with the years and probabilities from the calibration, sample 1 year out of the time window while weighting the probability a year gets selected by its probability density, e) store the date in a dataframe, and f) repeat 10,000 times. This process will produce a dataframe that is 17,670,000 rows long and has 10,000 individual year estimates for each individual. These year estimates, given the iterated sampling, should approximate the most likely year(s) of existence for each individual. These year estimates are not used in the evaluation of the Random Forest (RF) model below, but are used for the visualization process to show how diets change (or not) over time and across elevation zones in the Andes.

Note: this code may take a very long time (~10+ hours) to run.

```
#try to do this using multiple cores to speed up processing
parallel::detectCores()#Find how many cores the computer has
n.cores <- parallel::detectCores() - 2 #use all cores except 2
my.cluster <- parallel::makeCluster( #make a cluster of cores for use
  n.cores,
  type = "PSOCK"
)

print(my.cluster)#check to make sure the number of cores is correct

#register the core cluster as the one to use
doParallel::registerDoParallel(cl = my.cluster)
foreach::getDoParRegistered()
foreach::getDoParWorkers()

#Create an empty data.frame for the results
dat.df <- data.frame(matrix(ncol = 7, nrow = 0))
colnames(dat.df) <- c("Study.ID","Ind.ID","Run", "elev.factor", "C", "N", "year")#set th
at df column names

#Run the below code in parallel to get 10,000 year estimates for each individual - weigh
ting by the 14c date if the individual is radiocarbon dated

#NOTE: The code here is set for a subsample of 30 individuals (i = 1:30). This is to spe
ed up the runtime process. If you want to rerun this code for every individual you will
 need to either do i = 1:1767 or repeat the code chunk, changing i each time (for exampl
e: i = 1:30, i = 31:60, i = 61:90, etc.).
date.indiv.df <- foreach (i = 1:30, .combine = rbind) %dopar% {#do to every individual i
n the dataframe
  focal.ind <- dat[i,]#rotate through every row (individual)
    if (!is.na(focal.ind$C14.Date)) {#if the individual is radiocarbon dated (is not NA)
       date.14 <- rcarbon::calibrate(focal.ind$C14.Date, focal.ind$C14..., calCurves =
"shcal20")#calibrate their date
       pr.dens <- date.14$grids$`1`$PrDens #extract the probability for each year
       d.range <- data.frame(date.14$grids$`1`$calBP)#extract the years
     } else {
       yr.df <- data.frame(seq(focal.ind$End.BP, focal.ind$Start.BP, 1))#make a datafram
e with every year in the individual's time window
     }

  for (j in 1:10000) {#do this 10000 times for each of the individuals
    new.row <- data.frame(matrix(ncol = 0, nrow = 1))#reset the new dataframe
    new.row$Study.ID <- focal.ind$Study.ID #grab the unique study id
    new.row$Ind.ID <- focal.ind$Individual.ID#grab the individual's ID
    new.row$Run <- j#grab the run number
    new.row$elev.factor <- focal.ind$elev.factor #grab the elevation zone for the indivi
dual
    new.row$C <- focal.ind$C.b.coll. #grab their carbon value
    new.row$N <- focal.ind$N.b.coll. #grab their nitrogen value

      if (!is.na(focal.ind$C14.Date)) { #if an individual is radiocarbon dated,
        new.row$year <- d.range[sample(nrow(d.range), 1, prob = d.range$PrDens),]#sample
```

```
from their calibrated year window, weighting by probability a given year is the correct
 one
        dat.df <- rbind(dat.df, new.row) #add the individual to c.df
      }
      else { #if they aren't radiocarbon dated, do the following
        new.row$year <- yr.df[sample(nrow(yr.df), 1),] #sample from the time window with
no weighting - all years treated equally
        dat.df <- rbind(dat.df, new.row) #add the individual to c.df
      }

  }
  return(dat.df)#return the df for each individual with 10,000 rows. The .combine = rbin
d call in the foreach setup will rowbind each individuals 10,000 rows to those already c
ompleted, giving us a complete dataset of 17,670,000 rows at the end.
}

#When done, stop the cluster
parallel::stopCluster(cl = my.cluster)

#If repeating the above code in 30 individual sets to speed up runtime, rename each of t
he code copies to produce a unique dataframe and then bind them all together.
#date.indiv.df.complete <- do.call("rbind", list(date.indiv.df, date.indiv.df.2, date.in
div.df.3, date.indiv.df.4, date.indiv.df.5, date.indiv.df.6, date.indiv.df.7, date.indi
v.df.8, date.indiv.df.9, date.indiv.df.10, date.indiv.df.11, date.indiv.df.12, date.indi
v.df.13, date.indiv.df.14, date.indiv.df.15, date.indiv.df.16, date.indiv.df.17, date.in
div.df.18, date.indiv.df.19, date.indiv.df.20, date.indiv.df.21, date.indiv.df.22, date.
indiv.df.23, date.indiv.df.24, date.indiv.df.25, date.indiv.df.26, date.indiv.df.27, dat
e.indiv.df.28, date.indiv.df.29, date.indiv.df.30, date.indiv.df.31, date.indiv.df.32, d
ate.indiv.df.33, date.indiv.df.34, date.indiv.df.35, date.indiv.df.36, date.indiv.df.37,
date.indiv.df.38, date.indiv.df.39, date.indiv.df.40, date.indiv.df.41, date.indiv.df.4
2, date.indiv.df.43, date.indiv.df.44, date.indiv.df.45, date.indiv.df.46, date.indiv.d
f.47, date.indiv.df.48, date.indiv.df.49, date.indiv.df.50, date.indiv.df.51, date.indi
v.df.52, date.indiv.df.53, date.indiv.df.54, date.indiv.df.55, date.indiv.df.56, date.in
div.df.57, date.indiv.df.58, date.indiv.df.59))

#write the data to a csv
#write.csv(date.indiv.df.complete, "GAM_Visualization_10000yrs_per_ind.csv")
```

Next, now that we have 10,000 iterated sample years for each individual, we want to generate a median year for each individual. This median year is not used within the GAMs for the generation of the predicted fit lines, but for plotting the individual points on Figure 3.

```
#dat.df <- date.indiv.df.complete or
dat.df <- read.csv("GAM_Visualization_10000yrs_per_ind.csv")#read in the 17,670,000 row
 csv created above
dat.df$elev.factor <- as.factor(dat.df$elev.factor)#Ensure elevation zone is a factor va
riable
#get median year for each individual for plotting purposes only
med.year <- aggregate(dat.df[,8], list(dat.df$Study.ID), median)#For each of the individ
uals in the study (Study.ID), take their 10,000 sampled years and find the median year.
head(med.year)#Check to make sure this workd
dat$median.year <- med.year$x #Add the median year to the primary dat dataframe.

#Also create some subset dataframes for plotting purposes
coast.dat <- dat[which(dat$elev.factor == "Coastal"),]
midel.dat <- dat[which(dat$elev.factor == "Mid-elevation"),]
highl.dat <- dat[which(dat$elev.factor == "Highland"),]
```

Now, subset the large dataframe (dat.df) data into individuals with carbon and those with nitrogen for use in the GAMs.

```
dat.carbon <- dat.df[which(dat.df$C != "NA"),]#17,610,000 rows - 10,000 per individual
dat.nitrogen <- dat.df[which(dat.df$N != "NA"),]#17,270,000 rows
```

Then we generate the various GAMs to use for visualizing. We run the 10,000 iterated samples with 6 data subsets: Coastal, Mid-Elevation, and Highland individuals all on their own each for δ^{15}N‰ and δ^{13}C‰.

Start with δ^{15}N‰, coastal individuals. *Note: this code may take a long time (~50+ mins) to run.*

```
#remember the complete set of 10,000 year samples for each individual with nitrogen is d
at.nitrogen (17,270,000 rows)

#grab the levels of the factor variable - this is used in the predict area
elev.list <- levels(dat.nitrogen$elev.factor)

#setup an empty dataframe to receive the predicted data
coast.N.pred.df <- data.frame(matrix(ncol = 5, nrow = 0))

#make a dataframe of the years we want to predict over
pred.N.dat <- data.frame(
  year = seq(7000,300,-100)
)

#Grab the subset of individuals who are coastal
dat.nitrogen.coast <- dat.nitrogen[which(dat.nitrogen$elev.factor == "Coastal"),]


for (i in 1:10000) {#number of Gams which equals number of individual sample years. 10,0
00 GAMs will be made
  #make the gam
  nitrogen.gam <- gam(N ~ s(year),
      family = gaussian,
      data = subset(dat.nitrogen.coast, Run == i)
      )


  #predict nitrogen for every year from 300 ybp back to 7000 ybp in intervals of 100 yea
rs
  pred_output <- predict(nitrogen.gam,
                         newdata = pred.N.dat,
                         se.fit = TRUE,
                         type = "response")

  #combine response with labels
  pred.df <- data.frame(
    "year" = pred.N.dat$year,#year for which data is predicted
    "Run" = i,#which run the gam is based on. Runs are established in the iterated sampl
ing to get date estimates. Each individual has 10,000 runs (1 run per sampled year). For
the GAMs we run an individual GAM for each run, meaning for each GAM we have 1 single ye
ar treated as 'the true' year for each individual.
    "Pred.N" = pred_output$fit, #get the predicted nitrogen value for each year
    "SE.Pred.N" = pred_output$se.fit #get the standard error in nitrogen value for each
 year
  )

  #add this Run's predicted GAM output to the master dataframe for use in plotting
  coast.N.pred.df <- rbind(coast.N.pred.df, pred.df)

}

#Write the predicted output to a csv to avoid rerunning the code. This output is 10,000
```

```
 individual predicted fits of how nitrogen varies over time.
write.csv(coast.N.pred.df, "Coastal_Gams_preds_N_10000.csv")

coast.N.pred.df <- read.csv("Coastal_Gams_preds_N_10000.csv")
#this gives us just the coastal individual fit

#Subset the separated output dataframe by the max and min median year +- 200 years. This
allow us to plot predicted lines beginning where we have observations at each elevation
 zone (and in 200 years either earlier or later), rather than predicting beyond where we
have any observed values
coast.n.obs.yrs <- subset(coast.N.pred.df, year <= max(coast.dat$median.year+200) & year
>= min(coast.dat$median.year-200))

#Now calculate the central tendency line and the standard errors over observed space. Wh
at this code is doing is finding the mean central tendency, mean standard error, and mea
n standard deviation using all 10,000 GAMs. This allows us to plot a central tendency li
ne and confidence intervals based on the iterated samples without having to plot all 10,
000 lines.
coast.n.obs.mean <- aggregate(coast.n.obs.yrs[,4], list(coast.n.obs.yrs$year), FUN = mea
n)
coast.n.obs.se <- aggregate(coast.n.obs.yrs[,5], list(coast.n.obs.yrs$year), FUN = mean)
coast.n.obs.sd <- aggregate(coast.n.obs.yrs[,4], list(coast.n.obs.yrs$year), FUN = sd)
```

Now δ^{15}N‰ for mid-elevation individuals. *Note: this code may take a long time (~50+ mins) to run.*

```
#subset the data to get only mid-elevation individuals
dat.nitrogen.midel <- dat.nitrogen[which(dat.nitrogen$elev.factor == "Mid-elevation"),]

#setup an empty dataframe to receive the predicted data
midel.N.pred.df <- data.frame(matrix(ncol = 5, nrow = 0))

for (i in 1:10000) {#number of Gams which equals number of Runs
  #make the gam
  nitrogen.gam <- gam(N ~ s(year),
      family = gaussian,
      data = subset(dat.nitrogen.midel, Run == i)
      )

  pred_output <- predict(nitrogen.gam,
                       newdata = pred.N.dat,
                       se.fit = TRUE,
                       type = "response")

  #combine response with labels
  pred.df <- data.frame(
    "year" = pred.N.dat$year,
    "Run" = i,
    "Pred.N" = pred_output$fit,
    "SE.Pred.N" = pred_output$se.fit
  )

  #add this Run's predicted GAM output to the master dataframe for use in plotting
  midel.N.pred.df <- rbind(midel.N.pred.df, pred.df)

}

#Write results to a csv
write.csv(midel.N.pred.df, "Midel_Gams_preds_N_10000.csv")

midel.N.pred.df <- read.csv("Midel_Gams_preds_N_10000.csv")


#Subset the separated output dataframe by the max and min median year +- 200 years. This
allow us to plot predicted lines beginning where we have observations at each elevation
 zone (and in 200 years either earlier or later), rather than predicting beyond where we
have any observed values
midel.n.obs.yrs <- subset(midel.N.pred.df, year <= max(midel.dat$median.year+200) & year
>= min(midel.dat$median.year-200))


#Now calculate the central tendency line and the standard errors over observed space. Wh
at this code is doing is finding the mean central tendency, mean standard error, and mea
n standard deviation using all 10,000 GAMs. This allows us to plot a central tendency li
ne and confidence intervals based on the iterated samples without having to plot all 10,
000 lines.
midel.n.obs.mean <- aggregate(midel.n.obs.yrs[,3], list(midel.n.obs.yrs$year), FUN = mea
n)
```

```
midel.n.obs.se <- aggregate(midel.n.obs.yrs[,4], list(midel.n.obs.yrs$year), FUN = mean)
midel.n.obs.sd <- aggregate(midel.n.obs.yrs[,3], list(midel.n.obs.yrs$year), FUN = sd)
```

Now δ^{15}N‰ for highland individuals. *Note: this code may take a long time (~50+ mins) to run.*

```
dat.nitrogen.highl <- dat.nitrogen[which(dat.nitrogen$elev.factor == "Highland"),]

#setup an empty dataframe to receive the predicted data
highl.N.pred.df <- data.frame(matrix(ncol = 5, nrow = 0))

for (i in 1:10000) {#number of Gams which equals number of Runs
  #make the gam
  nitrogen.gam <- gam(N ~ s(year),
      family = gaussian,
      data = subset(dat.nitrogen.highl, Run == i)
      )

  pred_output <- predict(nitrogen.gam,
                       newdata = pred.N.dat,
                       se.fit = TRUE,
                       type = "response")

  #combine response with labels
  pred.df <- data.frame(
    "year" = pred.N.dat$year,
    "Run" = i,
    "Pred.N" = pred_output$fit,
    "SE.Pred.N" = pred_output$se.fit
  )

  #add this Run's predicted GAM output to the master dataframe for use in plotting
  highl.N.pred.df <- rbind(highl.N.pred.df, pred.df)
}

write.csv(highl.N.pred.df, "Highl_Gams_preds_N_10000.csv")

highl.N.pred.df <- read.csv("Highl_Gams_preds_N_10000.csv")

#Subset the separated output dataframe by the max and min median year +- 200 years. This
allow us to plot predicted lines beginning where we have observations at each elevation
 zone (and in 200 years either earlier or later), rather than predicting beyond where we
have any observed values
highl.n.obs.yrs <- subset(highl.N.pred.df, year <= max(highl.dat$median.year+200) & year
>= min(highl.dat$median.year-200))


#Now calculate the central tendency line and the standard errors over observed space. Wh
at this code is doing is finding the mean central tendency, mean standard error, and mea
n standard deviation using all 10,000 GAMs. This allows us to plot a central tendency li
ne and confidence intervals based on the iterated samples without having to plot all 10,
000 lines.
highl.n.obs.mean <- aggregate(highl.n.obs.yrs[,3], list(highl.n.obs.yrs$year), FUN = mea
n)
highl.n.obs.se <- aggregate(highl.n.obs.yrs[,4], list(highl.n.obs.yrs$year), FUN = mean)
highl.n.obs.sd <- aggregate(highl.n.obs.yrs[,3], list(highl.n.obs.yrs$year), FUN = sd)
```

Repeat the above, but for δ^{13}C‰, beginning with coastal individuals. *Note: this code may take a long time (~50+ mins) to run.*

```
#remember the complete set of 10,000 year samples for each individual with carbon is da
t.carbon

#grab the levels of the factor variable - this is used in the predict area
elev.list <- levels(dat.carbon$elev.factor)

#setup an empty dataframe to receive the predicted data
coast.C.pred.df <- data.frame(matrix(ncol = 5, nrow = 0))

#make a dataframe of the years we want to predict over
pred.C.dat <- data.frame(
  year = seq(7000,300,-100)
)

#Create the subset of coastal individuals
dat.carbon.coast <- dat.carbon[which(dat.carbon$elev.factor == "Coastal"),]


for (i in 1:10000) {#number of Gams which equals number of Runs
  #make the gam
  carbon.gam <- gam(C ~ s(year),
      family = gaussian,
      data = subset(dat.carbon.coast, Run == i)
      )

  pred_output <- predict(carbon.gam,
                         newdata = pred.C.dat,
                         se.fit = TRUE,
                         type = "response")

  #combine response with labels
  pred.df <- data.frame(
    "year" = pred.C.dat$year,
    "Run" = i,
    "Pred.C" = pred_output$fit,
    "SE.Pred.C" = pred_output$se.fit
  )

  #add this Run's predicted GAM output to the master dataframe for use in plotting
  coast.C.pred.df <- rbind(coast.C.pred.df, pred.df)

}

#Write the output to a csv to avoid having to rerun the code
write.csv(coast.C.pred.df, "Coast_Gams_preds_Carbon_10000.csv")

coast.C.pred.df <- read.csv("Coast_Gams_preds_Carbon_10000.csv")

#Subset the separated output dataframe by the max and min median year +- 200 years. This
allow us to plot predicted lines beginning where we have observations at each elevation
 zone (and in 200 years either earlier or later), rather than predicting beyond where we
have any observed values
```

```
coast.c.obs.yrs <- subset(coast.C.pred.df, year <= max(coast.dat$median.year+200) & year
>= min(coast.dat$median.year-200))


#Now calculate the central tendency line and the standard errors over observed space. Wh
at this code is doing is finding the mean central tendency, mean standard error, and mea
n standard deviation using all 10,000 GAMs. This allows us to plot a central tendency li
ne and confidence intervals based on the iterated samples without having to plot all 10,
000 lines.
coast.c.obs.mean <- aggregate(coast.c.obs.yrs[,3], list(coast.c.obs.yrs$year), FUN = mea
n)
coast.c.obs.se <- aggregate(coast.c.obs.yrs[,4], list(coast.c.obs.yrs$year), FUN = mean)
coast.c.obs.sd <- aggregate(coast.c.obs.yrs[,3], list(coast.c.obs.yrs$year), FUN = sd)
```

Now δ^{13}C‰ for mid-elevation individuals. *Note: this code may take a long time (~50+ mins) to run.*

```
dat.carbon.midel <- dat.carbon[which(dat.carbon$elev.factor == "Mid-elevation"),]

#setup an empty dataframe to receive the predicted data
midel.C.pred.df <- data.frame(matrix(ncol = 5, nrow = 0))

for (i in 1:10000) {#number of Gams which equals number of Runs
  #make the gam
  carbon.gam <- gam(C ~ s(year),
      family = gaussian,
      data = subset(dat.carbon.midel, Run == i)
      )

  pred_output <- predict(carbon.gam,
                       newdata = pred.C.dat,
                       se.fit = TRUE,
                       type = "response")

  #combine response with labels
  pred.df <- data.frame(
    "year" = pred.C.dat$year,
    "Run" = i,
    "Pred.C" = pred_output$fit,
    "SE.Pred.C" = pred_output$se.fit
  )

  #add this Run's predicted GAM output to the master dataframe for use in plotting
  midel.C.pred.df <- rbind(midel.C.pred.df, pred.df)

}

write.csv(midel.C.pred.df, "Midel_Gams_preds_Carbon_10000.csv")

midel.C.pred.df <- read.csv("Midel_Gams_preds_Carbon_10000.csv")


#Subset the separated output dataframe by the max and min median year +- 200 years. This
allow us to plot predicted lines beginning where we have observations at each elevation
 zone (and in 200 years either earlier or later), rather than predicting beyond where we
have any observed values
midel.c.obs.yrs <- subset(midel.C.pred.df, year <= max(midel.dat$median.year+200) & year
>= min(midel.dat$median.year-200))


#Now calculate the central tendency line and the standard errors over observed space. Wh
at this code is doing is finding the mean central tendency, mean standard error, and mea
n standard deviation using all 10,000 GAMs. This allows us to plot a central tendency li
ne and confidence intervals based on the iterated samples without having to plot all 10,
000 lines.
midel.c.obs.mean <- aggregate(midel.c.obs.yrs[,3], list(midel.c.obs.yrs$year), FUN = mea
n)
midel.c.obs.se <- aggregate(midel.c.obs.yrs[,4], list(midel.c.obs.yrs$year), FUN = mean)
midel.c.obs.sd <- aggregate(midel.c.obs.yrs[,3], list(midel.c.obs.yrs$year), FUN = sd)
```

Now δ^{13}C‰ for highland individuals. *Note: this code may take a long time (~50+ mins) to run.*

```
dat.carbon.highl <- dat.carbon[which(dat.carbon$elev.factor == "Highland"),]

#setup an empty dataframe to receive the predicted data
highl.C.pred.df <- data.frame(matrix(ncol = 5, nrow = 0))

for (i in 1:10000) {#number of Gams which equals number of Runs
  #make the gam
  carbon.gam <- gam(C ~ s(year),
      family = gaussian,
      data = subset(dat.carbon.highl, Run == i)
      )

  pred_output <- predict(carbon.gam,
                       newdata = pred.C.dat,
                       se.fit = TRUE,
                       type = "response")

  #combine response with labels
  pred.df <- data.frame(
    "year" = pred.C.dat$year,
    "Run" = i,
    "Pred.C" = pred_output$fit,
    "SE.Pred.C" = pred_output$se.fit
  )

  #add this Run's predicted GAM output to the master dataframe for use in plotting
  highl.C.pred.df <- rbind(highl.C.pred.df, pred.df)
}

write.csv(highl.C.pred.df, "Highl_Gams_preds_Carbon_10000.csv")

highl.C.pred.df <- read.csv("Highl_Gams_preds_Carbon_10000.csv")

#Subset the separated output dataframe by the max and min median year +- 200 years. This
allow us to plot predicted lines beginning where we have observations at each elevation
 zone (and in 200 years either earlier or later), rather than predicting beyond where we
have any observed values
highl.c.obs.yrs <- subset(highl.C.pred.df, year <= max(highl.dat$median.year+200) & year
>= min(highl.dat$median.year-200))



#Now calculate the central tendency line and the standard errors over observed space. Wh
at this code is doing is finding the mean central tendency, mean standard error, and mea
n standard deviation using all 10,000 GAMs. This allows us to plot a central tendency li
ne and confidence intervals based on the iterated samples without having to plot all 10,
000 lines.
highl.c.obs.mean <- aggregate(highl.c.obs.yrs[,3], list(highl.c.obs.yrs$year), FUN = mea
n)
highl.c.obs.se <- aggregate(highl.c.obs.yrs[,4], list(highl.c.obs.yrs$year), FUN = mean)
highl.c.obs.sd <- aggregate(highl.c.obs.yrs[,3], list(highl.c.obs.yrs$year), FUN = sd)
```

Make sure all of the central tendency data is set for plotting.

```
coast.N.pred.df <- read.csv("Coastal_Gams_preds_N_10000.csv")
#Subset the separated output dataframe by the max and min median year +- 200 years. This
allow us to plot predicted lines beginning where we have observations at each elevation
 zone (and in 200 years either earlier or later), rather than predicting beyond where we
have any observed values
coast.n.obs.yrs <- subset(coast.N.pred.df, year <= max(dat[which(dat$elev.factor == "Coa
stal"),]$median.year+200) & year >= min(dat[which(dat$elev.factor == "Coastal"),]$media
n.year-200))
#Now calculate the central tendency line and the standard errors over observed space. Wh
at this code is doing is finding the mean central tendency, mean standard error, and mea
n standard deviation using all 10,000 GAMs. This allows us to plot a central tendency li
ne and confidence intervals based on the iterated samples without having to plot all 10,
000 lines.
coast.n.obs.mean <- aggregate(coast.n.obs.yrs[,4], list(coast.n.obs.yrs$year), FUN = mea
n)
coast.n.obs.se <- aggregate(coast.n.obs.yrs[,5], list(coast.n.obs.yrs$year), FUN = mean)
coast.n.obs.sd <- aggregate(coast.n.obs.yrs[,4], list(coast.n.obs.yrs$year), FUN = sd)

midel.N.pred.df <- read.csv("Midel_Gams_preds_N_10000.csv")
#Subset the separated output dataframe by the max and min median year +- 200 years. This
allow us to plot predicted lines beginning where we have observations at each elevation
 zone (and in 200 years either earlier or later), rather than predicting beyond where we
have any observed values
midel.n.obs.yrs <- subset(midel.N.pred.df, year <= max(dat[which(dat$elev.factor == "Mid
-elevation"),]$median.year+200) & year >= min(dat[which(dat$elev.factor == "Mid-elevatio
n"),]$median.year-200))
#Now calculate the central tendency line and the standard errors over observed space. Wh
at this code is doing is finding the mean central tendency, mean standard error, and mea
n standard deviation using all 10,000 GAMs. This allows us to plot a central tendency li
ne and confidence intervals based on the iterated samples without having to plot all 10,
000 lines.
midel.n.obs.mean <- aggregate(midel.n.obs.yrs[,4], list(midel.n.obs.yrs$year), FUN = mea
n)
midel.n.obs.se <- aggregate(midel.n.obs.yrs[,5], list(midel.n.obs.yrs$year), FUN = mean)
midel.n.obs.sd <- aggregate(midel.n.obs.yrs[,4], list(midel.n.obs.yrs$year), FUN = sd)

highl.N.pred.df <- read.csv("Highl_Gams_preds_N_10000.csv")
#Subset the separated output dataframe by the max and min median year +- 200 years. This
allow us to plot predicted lines beginning where we have observations at each elevation
 zone (and in 200 years either earlier or later), rather than predicting beyond where we
have any observed values
highl.n.obs.yrs <- subset(highl.N.pred.df, year <= max(dat[which(dat$elev.factor == "Hig
hland"),]$median.year+200) & year >= min(dat[which(dat$elev.factor == "Highland"),]$medi
an.year-200))
#Now calculate the central tendency line and the standard errors over observed space. Wh
at this code is doing is finding the mean central tendency, mean standard error, and mea
n standard deviation using all 10,000 GAMs. This allows us to plot a central tendency li
ne and confidence intervals based on the iterated samples without having to plot all 10,
000 lines.
highl.n.obs.mean <- aggregate(highl.n.obs.yrs[,4], list(highl.n.obs.yrs$year), FUN = mea
n)
highl.n.obs.se <- aggregate(highl.n.obs.yrs[,5], list(highl.n.obs.yrs$year), FUN = mean)
```

```
highl.n.obs.sd <- aggregate(highl.n.obs.yrs[,4], list(highl.n.obs.yrs$year), FUN = sd)

coast.C.pred.df <- read.csv("Coast_Gams_preds_Carbon_10000.csv")
#Subset the separated output dataframe by the max and min median year +- 200 years. This
allow us to plot predicted lines beginning where we have observations at each elevation
 zone (and in 200 years either earlier or later), rather than predicting beyond where we
have any observed values
coast.c.obs.yrs <- subset(coast.C.pred.df, year <= max(dat[which(dat$elev.factor == "Coa
stal"),]$median.year+200) & year >= min(dat[which(dat$elev.factor == "Coastal"),]$media
n.year-200))
#Now calculate the central tendency line and the standard errors over observed space. Wh
at this code is doing is finding the mean central tendency, mean standard error, and mea
n standard deviation using all 10,000 GAMs. This allows us to plot a central tendency li
ne and confidence intervals based on the iterated samples without having to plot all 10,
000 lines.
coast.c.obs.mean <- aggregate(coast.c.obs.yrs[,4], list(coast.c.obs.yrs$year), FUN = mea
n)
coast.c.obs.se <- aggregate(coast.c.obs.yrs[,5], list(coast.c.obs.yrs$year), FUN = mean)
coast.c.obs.sd <- aggregate(coast.c.obs.yrs[,4], list(coast.c.obs.yrs$year), FUN = sd)

midel.C.pred.df <- read.csv("Midel_Gams_preds_Carbon_10000.csv")
#Subset the separated output dataframe by the max and min median year +- 200 years. This
allow us to plot predicted lines beginning where we have observations at each elevation
 zone (and in 200 years either earlier or later), rather than predicting beyond where we
have any observed values
midel.c.obs.yrs <- subset(midel.C.pred.df, year <= max(dat[which(dat$elev.factor == "Mid
-elevation"),]$median.year+200) & year >= min(dat[which(dat$elev.factor == "Mid-elevatio
n"),]$median.year-200))
#Now calculate the central tendency line and the standard errors over observed space. Wh
at this code is doing is finding the mean central tendency, mean standard error, and mea
n standard deviation using all 10,000 GAMs. This allows us to plot a central tendency li
ne and confidence intervals based on the iterated samples without having to plot all 10,
000 lines.
midel.c.obs.mean <- aggregate(midel.c.obs.yrs[,4], list(midel.c.obs.yrs$year), FUN = mea
n)
midel.c.obs.se <- aggregate(midel.c.obs.yrs[,5], list(midel.c.obs.yrs$year), FUN = mean)
midel.c.obs.sd <- aggregate(midel.c.obs.yrs[,4], list(midel.c.obs.yrs$year), FUN = sd)

highl.C.pred.df <- read.csv("Highl_Gams_preds_Carbon_10000.csv")
#Subset the separated output dataframe by the max and min median year +- 200 years. This
allow us to plot predicted lines beginning where we have observations at each elevation
 zone (and in 200 years either earlier or later), rather than predicting beyond where we
have any observed values
highl.c.obs.yrs <- subset(highl.C.pred.df, year <= max(dat[which(dat$elev.factor == "Hig
hland"),]$median.year+200) & year >= min(dat[which(dat$elev.factor == "Highland"),]$medi
an.year-200))
#Now calculate the central tendency line and the standard errors over observed space. Wh
at this code is doing is finding the mean central tendency, mean standard error, and mea
n standard deviation using all 10,000 GAMs. This allows us to plot a central tendency li
ne and confidence intervals based on the iterated samples without having to plot all 10,
000 lines.
highl.c.obs.mean <- aggregate(highl.c.obs.yrs[,4], list(highl.c.obs.yrs$year), FUN = mea
```

```
n)
highl.c.obs.se <- aggregate(highl.c.obs.yrs[,5], list(highl.c.obs.yrs$year), FUN = mean)
highl.c.obs.sd <- aggregate(highl.c.obs.yrs[,4], list(highl.c.obs.yrs$year), FUN = sd)
```

Finally, make Figure 3, the GAM fit with 95% confidence intervals created through Monte Carlo style simulated 10,000 GAMs for each elevation zone and isotope.

```
#png(filename = "Figure3_11.22.21_GAM.png", width = 7.3, height = 7.3, units = "in", res
= 300)
par(mfrow = c(2,1))
par(oma = c(1,2,0.5,1),mar = c(4,4.5,0,1))

plot(x = dat[which(dat$elev.factor == "Coastal"),]$median.year, y = dat[which(dat$elev.f
actor == "Coastal"),]$N.b.coll., col = coast.alpha, ylim = c(5, 30), xlim = c(0,7000), x
lab = "", ylab = expression({delta}^15*N~'\u2030'))
points(x = dat[which(dat$elev.factor == "Mid-elevation"),]$median.year, y = dat[which(da
t$elev.factor == "Mid-elevation"),]$N.b.coll., col = midel.alpha)
points(x = dat[which(dat$elev.factor == "Highland"),]$median.year, y = dat[which(dat$ele
v.factor == "Highland"),]$N.b.coll., col = highl.alpha)
polygon(x = c(coast.n.obs.mean$Group.1, rev(coast.n.obs.mean$Group.1)), y = c(coast.n.ob
s.mean$x+(coast.n.obs.se$x+(coast.n.obs.sd$x/sqrt(5))*1.96), rev(coast.n.obs.mean$x-(coa
st.n.obs.se$x+(coast.n.obs.sd$x/sqrt(5))*1.96))), col = coast.alpha, border = NA)
lines(x = coast.n.obs.mean$Group.1, y = coast.n.obs.mean$x, col = "#440154FF", lwd = 2)
polygon(x = c(midel.n.obs.mean$Group.1, rev(midel.n.obs.mean$Group.1)), y = c(midel.n.ob
s.mean$x+(midel.n.obs.se$x+(midel.n.obs.sd$x/sqrt(5))*1.96), rev(midel.n.obs.mean$x-(mid
el.n.obs.se$x+(midel.n.obs.sd$x/sqrt(5))*1.96))), col = midel.alpha, border = NA)
lines(x = midel.n.obs.mean$Group.1, y = midel.n.obs.mean$x, col = "#21908CFF", lwd = 2)
polygon(x = c(highl.n.obs.mean$Group.1, rev(highl.n.obs.mean$Group.1)), y = c(highl.n.ob
s.mean$x+(highl.n.obs.se$x+(highl.n.obs.sd$x/sqrt(5))*1.96), rev(highl.n.obs.mean$x-(hig
hl.n.obs.se$x+(highl.n.obs.sd$x/sqrt(5))*1.96))), col = highl.alpha, border = NA)
lines(x = highl.n.obs.mean$Group.1, y = highl.n.obs.mean$x, col = "#FDE725FF", lwd = 2)
abline(v=3749, lty = 2, col = "grey50", xpd = FALSE)
abline(v=2849, lty = 2, col = "grey50", xpd = FALSE)
abline(v=2449, lty = 2, col = "grey50", xpd = FALSE)
abline(v=1350, lty = 2, col = "grey50", xpd = FALSE)
abline(v=950, lty = 2, col = "grey50", xpd = FALSE)
abline(v=480, lty = 2, col = "grey50", xpd = FALSE)
abline(v=418, lty = 2, col = "grey50", xpd = FALSE)
mtext("LH", side = 1, line = -1, at = 449, cex = 0.75)
mtext("LIP", side = 1, line = -1, at = 715, cex = 0.75)
mtext("MH", side = 1, line = -1, at = 1150, cex = 0.75)
mtext("EIP", side = 1, line = -1, at = 1899.5, cex = 0.75)
mtext("EH", side = 1, line = -1, at = 2649, cex = 0.75)
mtext("IP", side = 1, line = -1, at = 3299, cex = 0.75)
mtext("PC", side = 1, line = -1, at = 4250, cex = 0.75)
mtext("A)", side = 1, line = -1, at = 6800, cex = 0.75)
legend("topright", bty="n", fill=c("#440154FF", "#21908CFF", "#FDE725FF"), legend=c("Coa
stal", "Mid-elevation", "Highland"))

plot(x = dat[which(dat$elev.factor == "Coastal"),]$median.year, y = dat[which(dat$elev.f
actor == "Coastal"),]$C.b.coll., col = coast.alpha, ylim = c(-20, -8), xlim = c(0,7000),
xlab = "yBP", ylab = expression({delta}^13*C~'\u2030'))
points(x = dat[which(dat$elev.factor == "Mid-elevation"),]$median.year, y = dat[which(da
t$elev.factor == "Mid-elevation"),]$C.b.coll., col = midel.alpha)
points(x = dat[which(dat$elev.factor == "Highland"),]$median.year, y = dat[which(dat$ele
v.factor == "Highland"),]$C.b.coll., col = highl.alpha)
polygon(x = c(coast.c.obs.mean$Group.1, rev(coast.c.obs.mean$Group.1)), y = c(coast.c.ob
s.mean$x+(coast.c.obs.se$x+(coast.c.obs.sd$x/sqrt(5))*1.96), rev(coast.c.obs.mean$x-(coa
st.c.obs.se$x+(coast.c.obs.sd$x/sqrt(5))*1.96))), col = coast.alpha, border = NA)
```

```
lines(x = coast.c.obs.mean$Group.1, y = coast.c.obs.mean$x, col = "#440154FF", lwd = 2)
polygon(x = c(midel.c.obs.mean$Group.1, rev(midel.c.obs.mean$Group.1)), y = c(midel.c.ob
s.mean$x+(midel.c.obs.se$x+(midel.c.obs.sd$x/sqrt(5))*1.96), rev(midel.c.obs.mean$x-(mid
el.c.obs.se$x+(midel.c.obs.sd$x/sqrt(5))*1.96))), col = midel.alpha, border = NA)
lines(x = midel.c.obs.mean$Group.1, y = midel.c.obs.mean$x, col = "#21908CFF", lwd = 2)
polygon(x = c(highl.c.obs.mean$Group.1, rev(highl.c.obs.mean$Group.1)), y = c(highl.c.ob
s.mean$x+(highl.c.obs.se$x+(highl.c.obs.sd$x/sqrt(5))*1.96), rev(highl.c.obs.mean$x-(hig
hl.c.obs.se$x+(highl.c.obs.sd$x/sqrt(5))*1.96))), col = highl.alpha, border = NA)
lines(x = highl.c.obs.mean$Group.1, y = highl.c.obs.mean$x, col = "#FDE725FF", lwd = 2)
abline(v=3749, lty = 2, col = "grey50", xpd = FALSE)
abline(v=2849, lty = 2, col = "grey50", xpd = FALSE)
abline(v=2449, lty = 2, col = "grey50", xpd = FALSE)
abline(v=1350, lty = 2, col = "grey50", xpd = FALSE)
abline(v=950, lty = 2, col = "grey50", xpd = FALSE)
abline(v=480, lty = 2, col = "grey50", xpd = FALSE)
abline(v=418, lty = 2, col = "grey50", xpd = FALSE)
mtext("LH", side = 1, line = -1, at = 449, cex = 0.75)
mtext("LIP", side = 1, line = -1, at = 715, cex = 0.75)
mtext("MH", side = 1, line = -1, at = 1150, cex = 0.75)
mtext("EIP", side = 1, line = -1, at = 1899.5, cex = 0.75)
mtext("EH", side = 1, line = -1, at = 2649, cex = 0.75)
mtext("IP", side = 1, line = -1, at = 3299, cex = 0.75)
mtext("PC", side = 1, line = -1, at = 4250, cex = 0.75)
mtext("B)", side = 1, line = -1, at = 6800, cex = 0.75)
legend("topright", bty="n", fill=c("#440154FF", "#21908CFF", "#FDE725FF"), legend=c("Coa
stal", "Mid-elevation", "Highland"))

#ev.off()
```

Fig. 3. δ^{15}N‰ and δ^{13}C‰ Changes over Time by Elevation Zone. Visually, Figure 3 produces results similar to that of the SIBER ellipses plots. Key visual patterns are 1) Coastal individuals are nearly always elevated in δ^{15}N‰ compared to mid-elevation and highland individuals, though this disappears during the Late Horizon. 2) Coastal individuals appear to have a general decrease in δ^{15}N‰ beginning in the EIP and continuing to the LH. 3) Mid-elevation and highland δ^{15}N‰ is virtually indistinguishable. 4) Mid-elevation individuals appear to experience higher δ^{15}N‰ during intermediate periods (EIP and LIP in particular) than during horizons (MH and LH). 5) During the LH, δ^{15}N‰ becomes nearly indistinguishable between elevation zones. 6) Coastal individuals experience a general rise in δ^{13}C‰ from the preceramic to the EIP, with relatively constant δ^{13}C‰ after the EIP. 7) Mid-elevation individuals also have a rise in δ^{13}C‰, though it is a little later than on the coast and is more drastic, rising from nearly -18δ^{13}C‰ during and before the EH to -12δ^{13}C‰ during the EIP and remaining around -12δ^{13}C‰ from then on. 8) Highland individuals appear to maintain a lower δ^{13}C‰ than other elevation zones beginning in the EIP and continuing through the LH. 9) There is a general trend of converging diets over time, with particularly mid-elevation and coastal individual's diets looking nearly indistinguishable by the LH.

While all of these visual patterns are intriguing, they cannot test hypotheses. They do, however, inspire our question of whether climate or demography might be more responsible for driving these changes. This question is evaluated next.

To start this evaluation, create two separate dataframes, one with all individuals with bone collagen δ^{15}N‰ and another for all individuals with bone collagen δ^{13}C‰. These are needed as we analyze the relationship between δ^{15}N‰ and δ^{13}C‰ separately.

```
data.N <- subset(dat, !is.na(dat$N.b.coll.))#leave out individuals with NA for N bone co
ll. 1727 individuals

data.C <- subset(dat, !is.na(dat$C.b.coll.))#leave out individuals with NA for C bone co
ll
```

Check the distributions of the individuals with δ^{15}N‰ in time across each elevational zone (coastal, mid-elevation, and highland).

```
data.N$med.date <- (data.N$Start.BP + data.N$End.BP)/2 #For plotting purposes only, assi
gn the mid-point of each individual's date range as the med.date.
N.coast <- data.N[which(data.N$elev.factor == "Coastal"),] #Make a dataframe with only c
oastal individuals.

N.midel <- data.N[which(data.N$elev.factor == "Mid-elevation"),] #Make a dataframe with
 only mid-elevation individuals.

N.highl <- data.N[which(data.N$elev.factor == "Highland"),] #Make a dataframe with only
 highland individuals.

#Plot histograms of the distributions in time
par(mfrow = c(2,2))
hist(data.N$med.date, xlab = "yBP", ylab = "No. Individuals", main = "", xlim = c(0,7000
))
mtext("All Individuals (n = 1727)", cex = 0.75)
hist(N.coast$med.date, col = "#440154FF", xlab = "yBP", ylab = "No. Individuals", main =
"", xlim = c(0,7000), ylim = c(0,300))
mtext("Coastal Individuals (n = 623)", cex = 0.75)
hist(N.midel$med.date,  col = "#21908CFF", xlab = "yBP", ylab = "No. Individuals", main
 = "", xlim = c(0,7000), ylim = c(0,300))
mtext("Mid-Elevation Individuals (n = 855)", cex = 0.75)
hist(N.highl$med.date, col = "#FDE725FF", xlab = "yBP", ylab = "No. Individuals", main =
"", xlim = c(0,7000), ylim = c(0,300))
mtext("Highland Individuals (n = 249)", cex = 0.75)
```

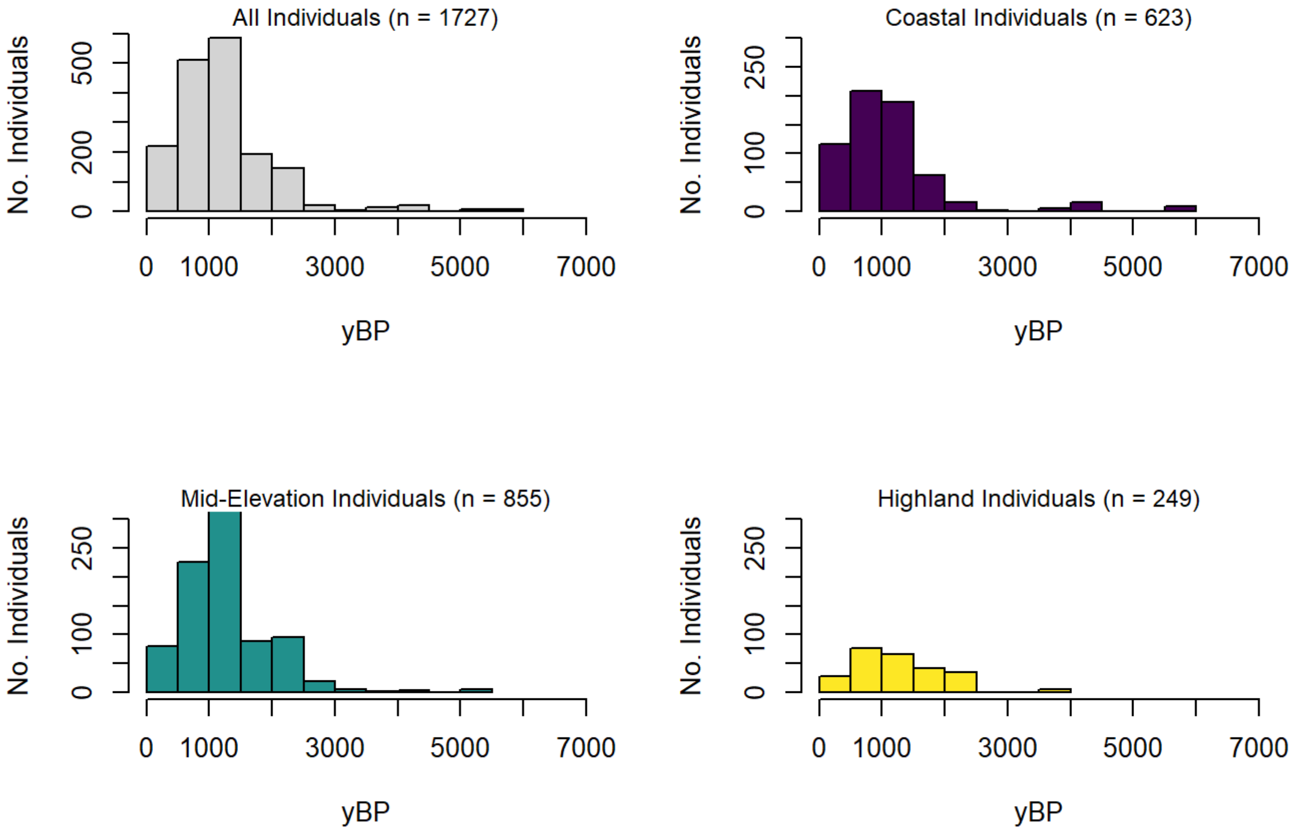

Fig. S2. δ^{15}N‰ Sample Temporal Distributions. We have a large proportion of our individuals representing the past ~2,000 years BP with a small proportion pre-dating that period. Coastal and Mid-elevation zones are more well-represented than the highlands. This suggests we will be more confident in the results relating to the past ~2,000 years and to the coastal and mid-elevation zones than earlier in time or in the highlands.

Now, check the distributions of the individuals with δ^{13}C‰ in time across each elevational zone (coastal, mid-elevation, and highland).

```
data.C$med.date <- (data.C$Start.BP + data.C$End.BP)/2 #these med dates are for plotting only and are not part of the statistical analysis
C.coast <- data.C[which(data.C$elev.factor == "Coastal"),]

C.midel <- data.C[which(data.C$elev.factor == "Mid-elevation"),]

C.highl <- data.C[which(data.C$elev.factor == "Highland"),]

#Plot histograms of the distributions in time
par(mfrow = c(2,2))
hist(data.C$med.date, xlab = "yBP", ylab = "No. Individuals", main = "", xlim = c(0,7000))
mtext("All Individuals (n = 1761)", cex = 0.75)
hist(C.coast$med.date, col = "#440154FF", xlab = "yBP", ylab = "No. Individuals", main = "", xlim = c(0,7000), ylim = c(0,300))
mtext("Coastal Individuals (n = 625)", cex = 0.75)
hist(C.midel$med.date,  col = "#21908CFF", xlab = "yBP", ylab = "No. Individuals", main = "", xlim = c(0,7000), ylim = c(0,300))
mtext("Mid-Elevation Individuals (n = 883)", cex = 0.75)
hist(C.highl$med.date, col = "#FDE725FF", xlab = "yBP", ylab = "No. Individuals", main = "", xlim = c(0,7000), ylim = c(0,300))
mtext("Highland Individuals (n = 253)", cex = 0.75)
```

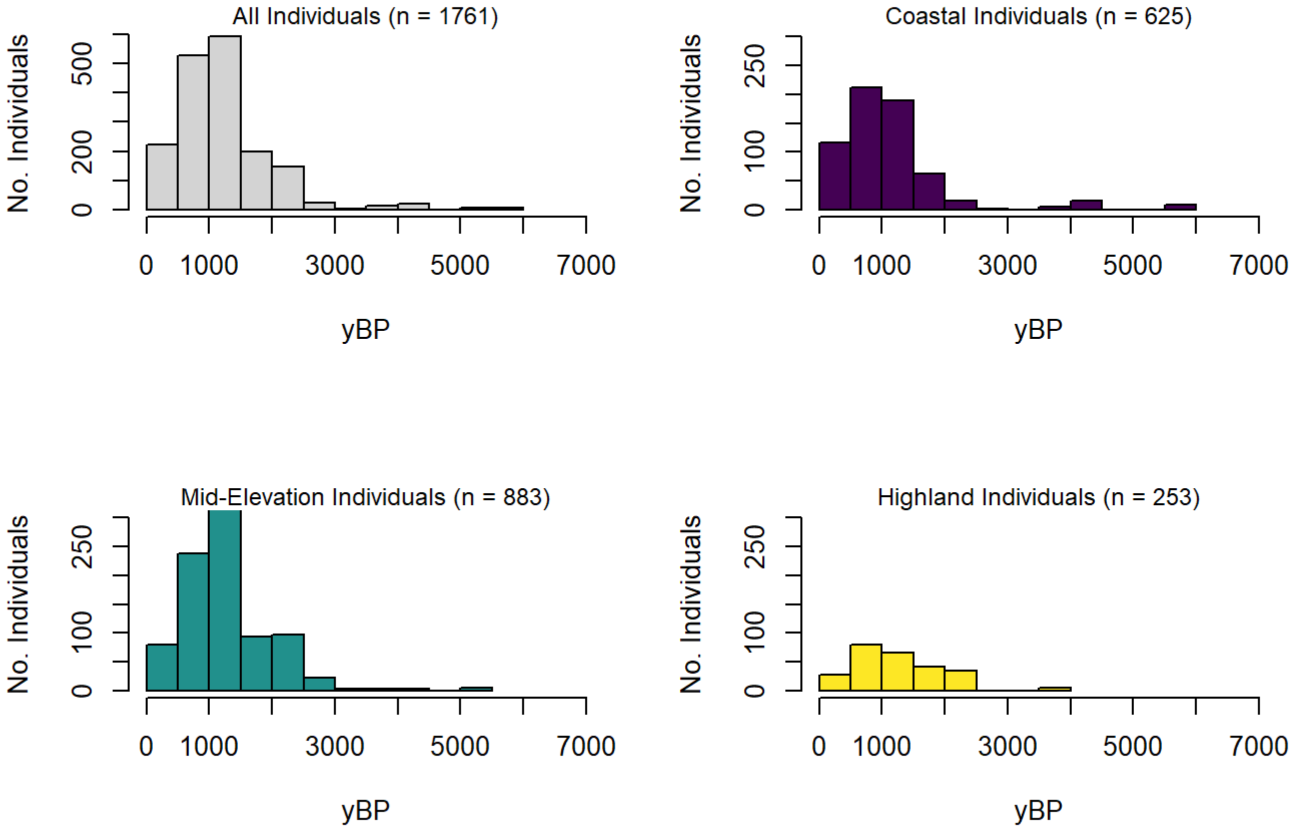

Fig. S3. δ^{13}C‰ Sample Temporal Distributions. These are very similar to the δ^{15}N‰ distributions, with strong representation of the past ~2,000 years BP and of the coasts and mid-elevations.

Next we check for autocorrelation among the variables, beginning with $\delta^{15}N$‰.

```
#Create a function for identifying collinearity among variables
cor.mtest <- function(mat) {
    mat <- as.matrix(mat)
    n <- ncol(mat)
    p.mat<- matrix(NA, n, n)
    diag(p.mat) <- 0
    for (i in 1:(n - 1)) {
        for (j in (i + 1):n) {
            tmp <- cor.test(mat[, i], mat[, j])
            p.mat[i, j] <- p.mat[j, i] <- tmp$p.value
        }
    }
  colnames(p.mat) <- rownames(p.mat) <- colnames(mat)
  p.mat
}


#build a data frame of predictor variables
dat_AllVars_Pred<-with(data.N, #use only individuals with nitrogen
             data.frame(
                    Temp_Mean, #Mean Temperature (c)
                    Precip_Mean, #Mean Precipitation (mm)
                    Temp_Mean_Seasonality, #Mean seasonality of temperature over the yea
r
                    Precip_Mean_Seasonality, #Mean seasonality of precipitation over the
year
                    Demography.kde.14c #mean spd value over timespan
             )
           )

#then test collinearity
cm<- cor(dat_AllVars_Pred,
         method="pearson",
         use="pairwise.complete.obs")


# matrix of the p-value of the correlation
p.mat<-cor.mtest(dat_AllVars_Pred)

#plot the correlation matrix
corrplot(cm,
      method="color",
      type="upper",
      diag=FALSE,
      bg="grey90",
      addCoef.col = "black",
      tl.col="black",
      outline = FALSE,
      addCoefasPercent=TRUE,
      p.mat = p.mat, sig.level = 0.05, insig = "blank"
  )
```

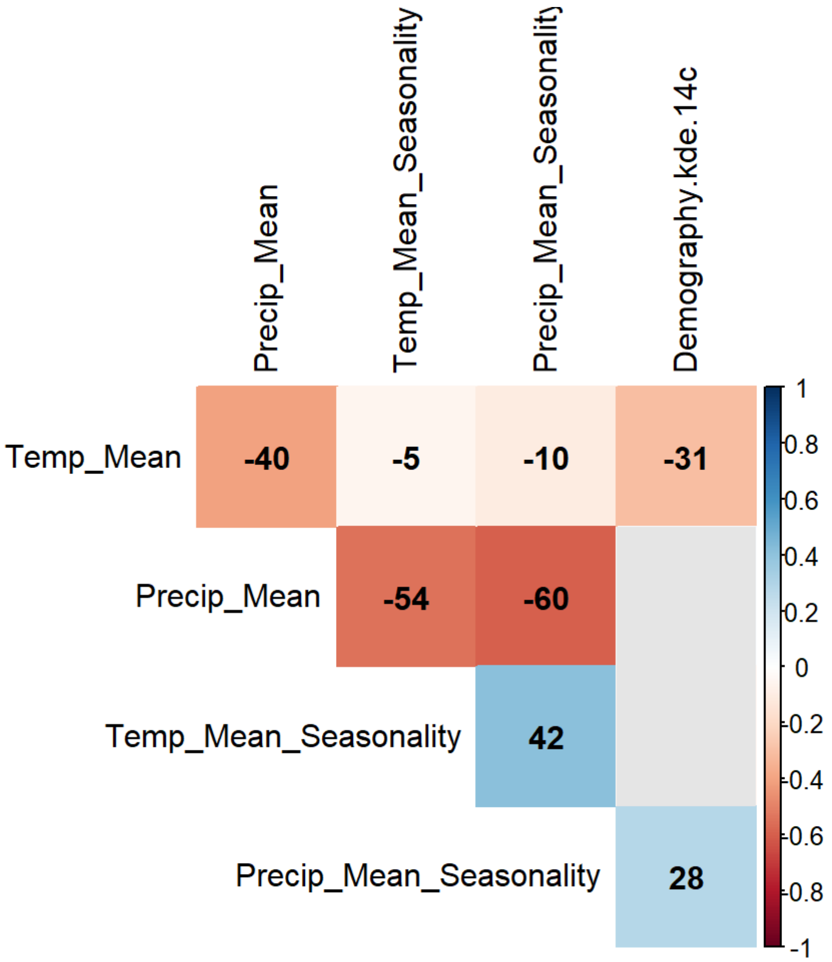

Fig. S4. δ^{15}N‰ Variable Correlation. Variables with correlation strengths less than 0.70 are viable for inclusion in the models (122) and none of our variables are correlated > 0.70. However some correlation does exist between multiple of our variables. While autocorrelation between variables exists, random forest analyses are capable of accounting for high levels of autcorrelation (120) and, therefore, we can confidently leave each variable in the analysis and parse out its individual effect (see below).

Now check autocorrelation among the variables for δ^{13}C‰.

```r
#Create a function for identifying collinearity among variables
cor.mtest <- function(mat) {
    mat <- as.matrix(mat)
    n <- ncol(mat)
    p.mat<- matrix(NA, n, n)
    diag(p.mat) <- 0
    for (i in 1:(n - 1)) {
        for (j in (i + 1):n) {
            tmp <- cor.test(mat[, i], mat[, j])
            p.mat[i, j] <- p.mat[j, i] <- tmp$p.value
        }
    }
  colnames(p.mat) <- rownames(p.mat) <- colnames(mat)
  p.mat
}


#build a data frame of predictor variables
dat_AllVars_Pred<-with(data.C,
             data.frame(
                    Temp_Mean, #Mean Temperature (c)
                    Precip_Mean, #Mean Precipitation (mm)
                    Temp_Mean_Seasonality, #Mean seasonality of temperature over the yea
r
                    Precip_Mean_Seasonality, #Mean seasonality of precipitation over the
year
                    Demography.kde.14c #mean spd across the individual's timespan
             )
           )

#then test collinearity
cm<- cor(dat_AllVars_Pred,
         method="pearson",
         use="pairwise.complete.obs")


# matrix of the p-value of the correlation
p.mat<-cor.mtest(dat_AllVars_Pred)

#plot the correlation matrix
corrplot(cm,
      method="color",
      type="upper",
      diag=FALSE,
      bg="grey90",
      addCoef.col = "black",
      tl.col="black",
      outline = FALSE,
      addCoefasPercent=TRUE,
      p.mat = p.mat, sig.level = 0.05, insig = "blank"
  )
```

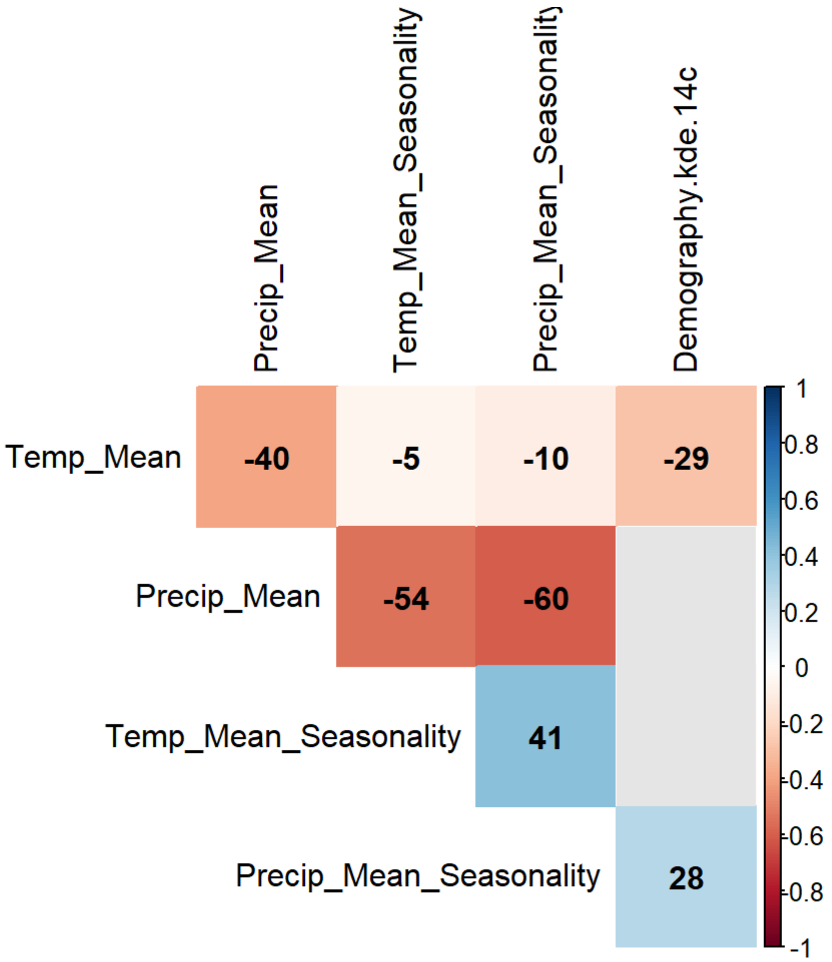

Fig. S5. δ^{13}C‰ Variable Correlation. Autocorrelation among variables is very similar to δ^{15}N‰ and none of the values are problematic for the RF analyses.

Random Forest Regression:

Assess if Observed Dietary Variation Results from Changing Population or Local Climate

Based on theoretical expectations of climate and demography influencing diet, we generate RF regressions that includes each of our variables: four aspects of climate plus elevational zone plus demography. First we analyze δ^{15}N‰.

To begin, subset the dataframe for just the variables included in the RF model.

```
dat.N <- data.N %>%
  dplyr::select(N.b.coll.,
         Temp_Mean,
         Precip_Mean,
         Temp_Mean_Seasonality,
         Precip_Mean_Seasonality,
         elev.factor,
         Demography.kde.14c)
```

Now run RF regression to determine how well our suite of 6 variables predict δ^{15}N‰.

```
set.seed(33)#set a seed for replicability
#examine how bone N values vary as a function of all other variables in the data
RF.N <- ranger(N.b.coll. ~ ., dat.N)#Use RF regression to predict nitrogen values using
 each of the predictor variables

print(RF.N)
```

```
## Ranger result
##
## Call:
##  ranger(N.b.coll. ~ ., dat.N)
##
## Type:                             Regression
## Number of trees:                  500
## Sample size:                      1727
## Number of independent variables:  6
## Mtry:                             2
## Target node size:                 5
## Variable importance mode:         none
## Splitrule:                        variance
## OOB prediction error (MSE):       4.173522
## R squared (OOB):                  0.7996307
```

The RF model appears to do well (r^2 = 79.96) at explaining the variance in bone collagen δ^{15}N‰. For validation purposes, we run multiple model performance evaluators below.

First, to better estimate how accurately our model predicts δ^{15}N‰, we use cross-validation following (149, 150, 151) to obtain the variance explained and root mean square error (RMSE).

```
myvars.N <- c("elev.factor", "Temp_Mean", "Precip_Mean", "Temp_Mean_Seasonality", "Preci
p_Mean_Seasonality", "Demography.kde.14c")#select the key explanatory variables we are u
sing in our analysis

pred.vars.N <- dat.N[myvars.N]#subset the isodat.N dataframe to keep only the predictor
 variables

set.seed(33)
RF.N.cv <- rgcv(pred.vars.N, dat.N$N.b.coll., cv.fold = 10)
RF.N.cv$rmse #root mean square error of cross-validated model
```

```
## [1] 2.049556
```

```
RF.N.cv$vecv #variance explained by the model based on cross-validation
```

```
## [1] 79.821
```

Cross-validated results show that the median permuted percent variance explained is 79.82 meaning we explain 79.82% of the variability in bone collagen δ^{15}N‰ with our predictor variables. Mean RMSE is 2.05.

Next we check the model residuals for normalcy and temporal autcorrelation. To do so we first create a Prediction function for use with the ranger (RF) model as ranger has a non-standard predict function.

```
pred <- function(model, newdata)  {
  results <- as.data.frame(predict(model, newdata)$predictions)
  return(results)
}
```

Plot δ^{15}N‰ model residuals to check for normalcy:

```
pred.N <-  pred(RF.N, dat.N) #Use the pred function to predict the Nitrogen value for ea
ch individual

par(pty="s")

RF.resid.N <- dat.N$N.b.coll. - pred.N #calculate the residuals
colnames(RF.resid.N) <- "Residuals"

#bug <- as.numeric(unlist(RF.resid.N))

hist(RF.resid.N, col="grey", main=NA, xlab="Residuals")
```

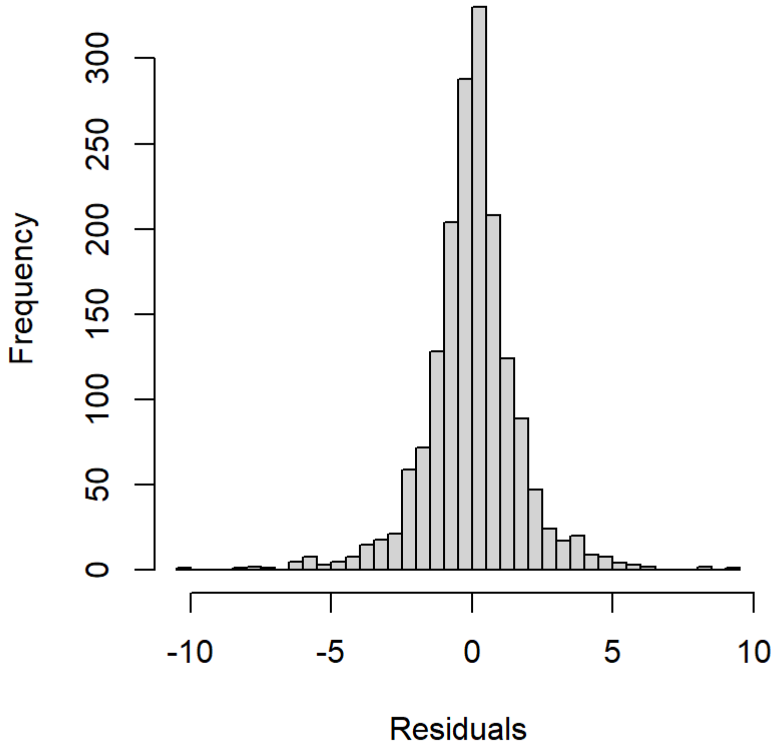

n:1727 m:0

Fig. S6. δ^{15}N‰ RF Model Residuals. Residuals are normally distributed around 0.

Check for temporal autocorrelation in the δ^{15}N‰ residuals, following “Time after time: calculating the autocorrelation function for uneven or grouped time series” by Ariel Muldoon https://aosmith.rbind.io/2018/06/27/uneven-grouped-autocorrelation/ (https://aosmith.rbind.io/2018/06/27/uneven-grouped-autocorrelation/).

```
#Add the median year to the residuals dataframe for use in ordering individuals
RF.resid.N$median.date <- data.N$median.year

#Arrange the individuals by their median date
RF.resid.N.dat <- RF.resid.N %>% arrange(median.date)

#For individuals who have the same median year, aggregate their residuals into one obser
vation by taking the mean of each year with repeated individuals.
RF.resid.N.dat <- aggregate(RF.resid.N.dat, by = list(RF.resid.N.dat$median.date), FUN =
mean)

#Pad the dataset with NAs so each elevation zone has an equal number of rows. We want 1
 row per year from the minimum median date to the maximum median date across the full te
mporal spread. This is 385 to 5941 yBP.
RF.resid.N.dat.expand <- RF.resid.N.dat %>%
  complete(median.date = 385:5941)

corr <- acf(RF.resid.N.dat.expand$Residuals, lag.max=300,type="correlation",plot=TRUE,n
a.action=na.pass, main = "", xlab = "Lag (yrs)")#generate an acf plot of the residuals t
o visually assess temporal autocorrelation
mtext("All Individuals with Nitrogen", side = 3, line = 0.5)
```

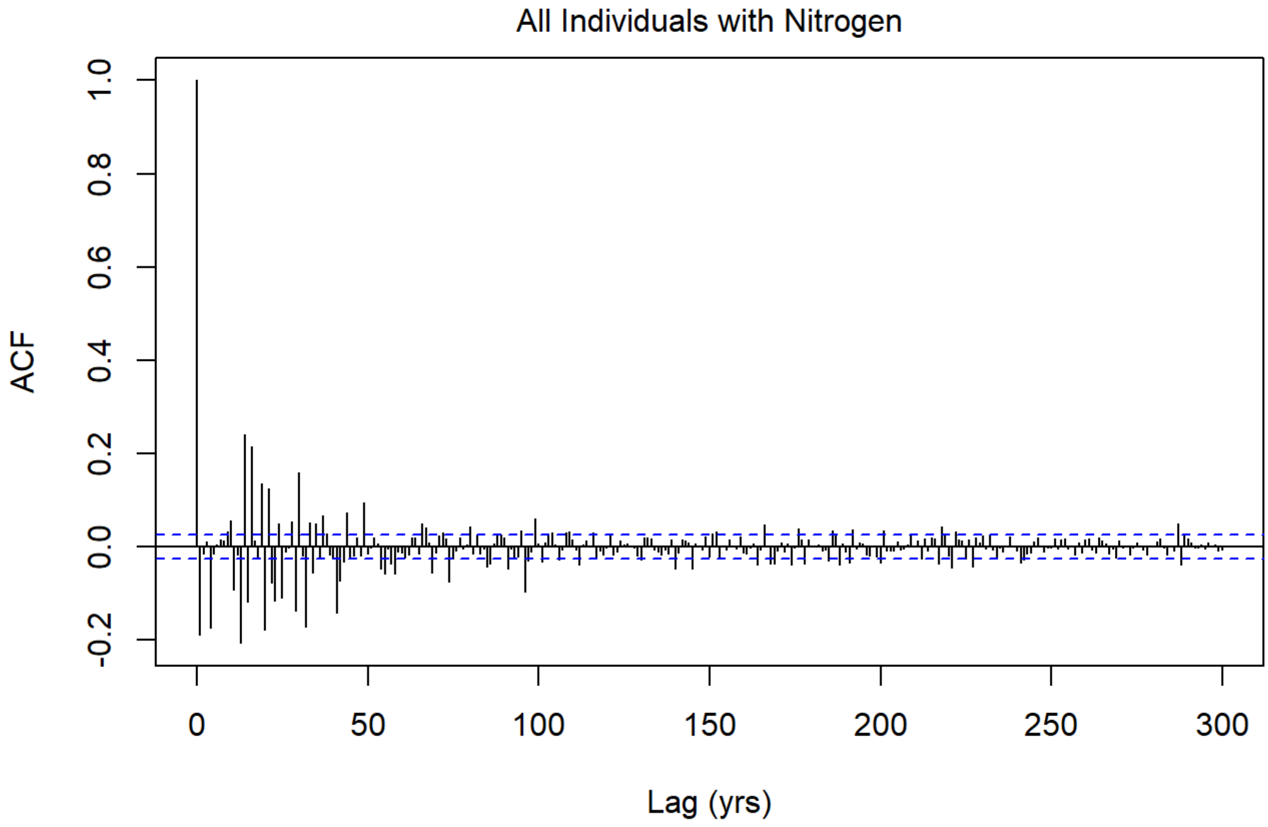

Fig. S7. Temporal Autocorrelation ACF Plot for δ^{15}N‰. ACF plot shows there is some temporal autocorrelation in the residuals up to about 200 years. This is not surprising given the way dates are assigned to individuals. The autocorrelation is not overly patterned but may exert some influence.

Check for Spatial Autocorrelation within the residuals.

```
#make a distance matrix
spat.dist <- as.matrix(dist(cbind(data.N$Latitude, data.N$Longitude)))
spat.dist.inv <- 1/spat.dist #inverse distance matrix
spat.dist.inv[is.infinite(spat.dist.inv)] <- 0 #set any infinite values to zero

#Check for spatial autocorrelation using global Moran's I
data.frame("Coordinates"=round(unlist(c(Moran.I(RF.resid.N$Residuals, spat.dist.inv, na.
rm=T))), 4)
           )
```

```
##          Coordinates
## observed     -0.0027
## expected     -0.0006
## sd            0.0029
## p.value       0.4701
```

The p-value is 0.4701, thus we do not reject the null hypothesis that there is zero spatial autocorrelation in the residuals. This suggests we do not have residual spatial variation structuring residual variation.

We evaluate the influence of each variable within each elevational cateogry by calcualting the effect size as the model sum of squares, discounted by the proportion of the variable's effect that results from its interaction with the other variables.

First make a predictor to use with the IML package:

```
mod <- Predictor$new(RF.N,
                     data = dat.N[-which(names(dat.N) == "N.b.coll.")],
                     y = dat.N$N.b.coll.,
                     predict.function = pred)
```

Calculate Friedman's H-Stat using IML. This is the proportion of the effect of each variable due to its interaction with the other variables for the complete model (not subset by elevational zone).

```
ia <- suppressMessages(Interaction$new(mod))
ia
```

```
## Interpretation method:  Interaction
##
##
## Analysed predictor:
## Prediction task: unknown
##
##
## Analysed data:
## Sampling from data.frame with 1727 rows and 6 columns.
##
## Head of results:
##                   .feature .interaction
## 1:                Temp_Mean    0.2314946
## 2:              Precip_Mean    0.1996338
## 3:   Temp_Mean_Seasonality    0.3145577
## 4: Precip_Mean_Seasonality    0.1550968
## 5:              elev.factor    0.1704729
## 6:       Demography.kde.14c    0.3398536
```

We are interested in how the variables predict δ^{15}N‰ within each of the elevational zones, however. To get the interaction and effect sizes within each elevational category, we take the below steps.

First, grab the data set

```
#This code grabs all of the input data for our RF model. This is the complete set of cas
es from our raw data (in other words, all 1679 individuals with nitrogen on bone collage
n)
data.sample <- ia$.__enclos_env__$private$sampler$get.x()
```

Second, set the batch size for processing (1000)

```
batch.size <- ia$predictor$batch.size
```

Third, set the grid size for calculating Partial Dependencies

```
#this is how many points within the chosen variable's observed range we use to calculate the effect size. Using mean_temp as an example, a grid of 100 will equate to randomly choosing 100 values of mean_temp. This then creates 100 repeated iterations of our complete dataset and assigns 1 of the 100 mean_temp values to the first copy of the dataset followed by assigning the 2nd mean_temp value to the second copy of the data set and so on. This allows us to get the effect size for temp_mean by predicting $\delta$^15^N‰ for every individual at each of the 100 chosen mean_temp values.
grid.size <- ia$grid.size
grid.size <- 100
```

Fourth, set the mean function (required)

```
myq <- ia$.__enclos_env__$private$q
```

Fifth, choose which predictor variable to calculate

```
#Choose the variable of interest. This will need to be repeated for each of our predictor variables (Temp_Mean, Temp_Mean_Seasonality, Precip_Mean, Precip_Mean_Seasonality, and Demography.spd)
feature.name <- c("Temp_Mean")
```

Sixth, set the prediction function

```
predictor <- ia$predictor
```

Seventh, get all the interactions for the chosen variable, calculate the Friedman's H-stat (proportion of the effect due to the variable's interaction with the other variables), calulate the effect size as the model sum of squares, discount the effect size based on the Friedman's H-stat, and calculate the effect size a a percent of the variation explained. For additional information on Friedman's H-stat, see (124). *Note: this code may take a long time (~10+ mins per variable) to run.*

```
#iterate the data sampling for interactions 100 times to increase accuracy of output.

runs <- seq(1, 100, 1)

#Make an object for the sampling output to go into
myia <- list(res = data.table(), qr = data.frame())

#Make a datafame object for the results of each iteration
N.Result.df <- data.frame()

#Make an empty dataframe for the total results to append to
Var.N.Result.df <- data.frame()



for (k in runs) {

  myia <- interaction.run.single(
  dataSample = data.sample,
  feature.name = feature.name[1],
  grid.size = grid.size,
  batch.size = batch.size,
  q = myq,
  predictor = predictor
  )


  partial_dat <- myia$res #make a dataframe with the data generated for the interactions
  partial_dat$.y.hat <- myia$qr #add the predicted values to the dataframe
  partial_dat$.class <- 1 #specify 1 so when we do the prediction R knows this is a regr
ession problem

  #collapse into 'F(x)' values - this will provide the average of all of the predictions
for each unique value of the chosen variable. So average of all predictions per observat
ion on grid and per value of the feature.
  pd.all <- data.table::dcast(partial_dat, .feature + .id + elev.factor + .class ~ .typ
e,
                        value.var = ".y.hat", fun.aggregate = mean
)

#We get a set of 300 predictions with pd.all, but we are missing 200 f and no.j values w
hich is because the chosen predictor value isn't present at 2 of the 3 elevational categ
ories. Therefore we remove the NA values, bringing us back to the 100 predictions we obt
ained. We can then subset the 100 predictions by which elevation factor they represent.
 This will give us a breakdown of the 100 into 3 subsets.
  pd.all.nona <- pd.all[which(!is.na(pd.all$f)),]#drop NA rows, leaving the 100 aggregat
ed predictions
  pd.all.nona.coast <- pd.all.nona[which(pd.all.nona$elev.factor == "Coastal"),]#Grab on
ly coastal cases.
  pd.all.nona.midel <- pd.all.nona[which(pd.all.nona$elev.factor == "Mid-elevation"),]#G
rab only mid-elevation cases.
  pd.all.nona.highl <- pd.all.nona[which(pd.all.nona$elev.factor == "Highland"),]#Grab o
```

```
nly highland cases.

  #Calculate Total Response of Nitrogen
  f.all <- scale(pd.all.nona$f, scale = FALSE)
  f.coast <- scale(pd.all.nona.coast$f, scale = FALSE)
  f.midel <- scale(pd.all.nona.midel$f, scale = FALSE)
  f.highl <- scale(pd.all.nona.highl$f, scale = FALSE)

  #Calculate Partial Dependence of Nitrogen for Chosen Variable
  f.j <- scale(pd.all.nona$j, scale = FALSE)
  f.j.coast <- scale(pd.all.nona.coast$j, scale = FALSE)
  f.j.midel <- scale(pd.all.nona.midel$j, scale = FALSE)
  f.j.highl <- scale(pd.all.nona.highl$j, scale = FALSE)

  #Calculate Combined Partial Dependence of Nitrogen to all Other Variables
  f.no.j <- scale(pd.all.nona$no.j, scale = FALSE)
  f.noj.coast <- scale(pd.all.nona.coast$no.j, scale = FALSE)
  f.noj.midel <- scale(pd.all.nona.midel$no.j, scale = FALSE)
  f.noj.highl <- scale(pd.all.nona.highl$no.j, scale = FALSE)

  #Calculate Friedman's H-Stat to get the proportion of the effect due to interaction wi
th the other variables
  h.all <- sqrt(sum((f.all - (f.j + f.no.j))^2) / sum(f.all^2))
  h.coast <- sqrt(sum((f.coast - (f.j.coast + f.noj.coast))^2) / sum(f.coast^2))
  h.midel <- sqrt(sum((f.midel - (f.j.midel + f.noj.midel))^2) / sum(f.midel^2))
  h.highl <- sqrt(sum((f.highl - (f.j.highl + f.noj.highl))^2) / sum(f.highl^2))

  #Assign the Friedman's H-Stat to a dataframe
  N.Result.df <- data.frame(var = "Temp Mean", H.Stat = h.all, H.Stat.Coast = h.coast,
 H.Stat.Mid = h.midel, H.Stat.High = h.highl)

  #Model Effect Size of the Chosen Variable (Amount of the per mil variance explained by
the chosen variable)
  sqrt(sum((f.j)^2))
  sqrt(sum((f.j.coast)^2))
  sqrt(sum((f.j.midel)^2))
  sqrt(sum((f.j.highl)^2))

  #Add the effect size to the dataframe
  meant.EF <- data.frame(EF = sqrt(sum((f.j)^2)), EF.coast = sqrt(sum((f.j.coast)^2)), E
F.midel = sqrt(sum((f.j.midel)^2)), EF.high = sqrt(sum((f.j.highl)^2)))
  N.Result.df <- cbind(N.Result.df, meant.EF)

  #Percent Effect of the Chosen Variable (Percent of the variance in Nitrogen explained
 by the chosen variable)
  sqrt(sum(f.j^2) / sum(f.all^2))
  sqrt(sum(f.j.coast^2) / sum(f.coast^2))
  sqrt(sum(f.j.midel^2) / sum(f.midel^2))
  sqrt(sum(f.j.highl^2) / sum(f.highl^2))

  #Assign the percent effect size to the dataframe
  meant.EF.per <- data.frame(EF.per = sqrt(sum(f.j^2) / sum(f.all^2)), EF.per.coast = sq
```

```
rt(sum(f.j.coast^2) / sum(f.coast^2)), EF.per.midel = sqrt(sum(f.j.midel^2) / sum(f.mide
l^2)), EF.per.high = sqrt(sum(f.j.highl^2) / sum(f.highl^2)))
  N.Result.df <- cbind(N.Result.df, meant.EF.per)

  #Now use the Friedman's H-Stat to control the effect size and percent to get the amoun
t of the variance explained by the chosen variable without its interaction with other va
riables
  #Effect Size Controlled
  sqrt(sum((f.j*(1-h.all))^2))
  sqrt(sum((f.j.coast*(1-h.coast))^2))
  sqrt(sum((f.j.midel*(1-h.midel))^2))
  sqrt(sum((f.j.highl*(1-h.highl))^2))

  #Assign the controlled effect size to a dataframe
  meant.EF.cont <- data.frame(EF.cont = sqrt(sum((f.j*(1-h.all))^2)), EF.cont.coast = sq
rt(sum((f.j.coast*(1-h.coast))^2)), EF.cont.midel = sqrt(sum((f.j.midel*(1-h.midel))^2
)), EF.cont.highl = sqrt(sum((f.j.highl*(1-h.highl))^2)))
  N.Result.df <- cbind(N.Result.df, meant.EF.cont)

  #Percent Effect of the Chosen Variable Controlled with Friedman's H-Stat
  #Effect Size Percent Controlled
  sqrt(sum((f.j*(1-h.all))^2) / sum(f.all^2))
  sqrt(sum((f.j.coast*(1-h.coast))^2) / sum(f.coast^2))
  sqrt(sum((f.j.midel*(1-h.midel))^2) / sum(f.midel^2))
  sqrt(sum((f.j.highl*(1-h.highl))^2) / sum(f.highl^2))

    #Assign the controlled percent effect size to a dataframe
  meant.EF.per.cont <- data.frame(EF.per.cont = sqrt(sum((f.j*(1-h.all))^2) / sum(f.all^
2)), EF.per.cont.coast = sqrt(sum((f.j.coast*(1-h.coast))^2) / sum(f.coast^2)), EF.per.c
ont.midel = sqrt(sum((f.j.midel*(1-h.midel))^2) / sum(f.midel^2)), EF.per.cont.highl = s
qrt(sum((f.j.highl*(1-h.highl))^2) / sum(f.highl^2)))
  N.Result.df <- cbind(N.Result.df, meant.EF.per.cont)


  if (k == 1) {
    Var.N.Result.df <- N.Result.df
  }
  else {
  Var.N.Result.df <- rbind(Var.N.Result.df, N.Result.df)
  }
}

#You can check to see if the output is correct - there should be 100 rows of 21 columns
#Var.N.Result.df

#Average the 21 columns and assign the values to a new dataframe. This gives us the aver
age effect sizes based on 100 iterations of randomly sampling 100 values for the chosen
 variable, assigning those values to each individual, and predicting their N value.
Total.N.Result.df <- data.frame(row.names = "Temp Mean", t(colMeans(Var.N.Result.df[2:21
])))
```

Eighth, repeat for each of the other predictor variables.

Repeat for Mean Precip Vector of features to calculate

```
#Choose the variable of interest. This will need to be repeated for each of our predicto
r variables (Temp_Mean, Temp_Mean_Seasonality, Precip_Mean, Precip_Mean_Seasonality, and
Demography.spd)
feature.name <- c("Precip_Mean")
```

Prediction function

```
predictor <- ia$predictor
```

Now get all the interactions for the chosen variable, calculate the Friedman's H-stat (proportion of the effect due to the variable's interaction with the other variables), calulate the effect size as the model sum of squares, discount the effect size based on the Friedman's H-stat, and calculate the effect size a a percent of the variation explained.

```
#iterate the data sampling for interactions 100 times.
runs <- seq(1, 100, 1)

#Make an object for the sampling output to go into
myia <- list(res = data.table(), qr = data.frame())

#Make a datafame object for the results of each iteration
N.Result.df <- data.frame()

#Make an empty dataframe for the total results to append to
Var.N.Result.df <- data.frame()



for (k in runs) {

  myia <- interaction.run.single(
  dataSample = data.sample,
  feature.name = feature.name[1],
  grid.size = grid.size,
  batch.size = batch.size,
  q = myq,
  predictor = predictor
  )


  partial_dat <- myia$res #make a dataframe with the data generated for the interactions
  partial_dat$.y.hat <- myia$qr #add the predicted values to the dataframe
  partial_dat$.class <- 1 #specify 1 so when we do the prediction R knows this is a regr
ession problem

  #collapse into 'F(x)' values - this will provide the average of all of the predictions
for each unique value of the chosen variable. So average of all predictions per observat
ion on grid and per value of the feature.
  pd.all <- data.table::dcast(partial_dat, .feature + .id + elev.factor + .class ~ .typ
e,
                        value.var = ".y.hat", fun.aggregate = mean
)

  pd.all.nona <- pd.all[which(!is.na(pd.all$f)),]#drop NA rows, leaving the 100 aggregat
ed predictions
  pd.all.nona.coast <- pd.all.nona[which(pd.all.nona$elev.factor == "Coastal"),]#Grab on
ly coastal cases.
  pd.all.nona.midel <- pd.all.nona[which(pd.all.nona$elev.factor == "Mid-elevation"),]#G
rab only mid-elevation cases.
  pd.all.nona.highl <- pd.all.nona[which(pd.all.nona$elev.factor == "Highland"),]#Grab o
nly highland cases.

  #Calculate Total Response of Nitrogen
  f.all <- scale(pd.all.nona$f, scale = FALSE)
  f.coast <- scale(pd.all.nona.coast$f, scale = FALSE)
  f.midel <- scale(pd.all.nona.midel$f, scale = FALSE)
```

```
  f.highl <- scale(pd.all.nona.highl$f, scale = FALSE)

  #Calculate Partial Dependence of Nitrogen for Chosen Variable
  f.j <- scale(pd.all.nona$j, scale = FALSE)
  f.j.coast <- scale(pd.all.nona.coast$j, scale = FALSE)
  f.j.midel <- scale(pd.all.nona.midel$j, scale = FALSE)
  f.j.highl <- scale(pd.all.nona.highl$j, scale = FALSE)

  #Calculate Combined Partial Dependence of Nitrogen to all Other Variables
  f.no.j <- scale(pd.all.nona$no.j, scale = FALSE)
  f.noj.coast <- scale(pd.all.nona.coast$no.j, scale = FALSE)
  f.noj.midel <- scale(pd.all.nona.midel$no.j, scale = FALSE)
  f.noj.highl <- scale(pd.all.nona.highl$no.j, scale = FALSE)

  #Calculate Friedman's H-Stat to get the proportion of the effect due to interaction wi
th the other variables
  h.all <- sqrt(sum((f.all - (f.j + f.no.j))^2) / sum(f.all^2))
  h.coast <- sqrt(sum((f.coast - (f.j.coast + f.noj.coast))^2) / sum(f.coast^2))
  h.midel <- sqrt(sum((f.midel - (f.j.midel + f.noj.midel))^2) / sum(f.midel^2))
  h.highl <- sqrt(sum((f.highl - (f.j.highl + f.noj.highl))^2) / sum(f.highl^2))

  #Assign the Friedman's H-Stat to a dataframe
  N.Result.df <- data.frame(var = "Precip Mean", H.Stat = h.all, H.Stat.Coast = h.coast,
H.Stat.Mid = h.midel, H.Stat.High = h.highl)

  #Model Effect Size of the Chosen Variable (Amount of the per mil variance explained by
the chosen variable)
  sqrt(sum((f.j)^2))
  sqrt(sum((f.j.coast)^2))
  sqrt(sum((f.j.midel)^2))
  sqrt(sum((f.j.highl)^2))

  #Add the effect size to the dataframe
  meant.EF <- data.frame(EF = sqrt(sum((f.j)^2)), EF.coast = sqrt(sum((f.j.coast)^2)), E
F.midel = sqrt(sum((f.j.midel)^2)), EF.high = sqrt(sum((f.j.highl)^2)))
  N.Result.df <- cbind(N.Result.df, meant.EF)

  #Percent Effect of the Chosen Variable (Percent of the variance in Nitrogen explained
 by the chosen variable)
  sqrt(sum(f.j^2) / sum(f.all^2))
  sqrt(sum(f.j.coast^2) / sum(f.coast^2))
  sqrt(sum(f.j.midel^2) / sum(f.midel^2))
  sqrt(sum(f.j.highl^2) / sum(f.highl^2))

  #Assign the percent effect size to the dataframe
  meant.EF.per <- data.frame(EF.per = sqrt(sum(f.j^2) / sum(f.all^2)), EF.per.coast = sq
rt(sum(f.j.coast^2) / sum(f.coast^2)), EF.per.midel = sqrt(sum(f.j.midel^2) / sum(f.mide
l^2)), EF.per.high = sqrt(sum(f.j.highl^2) / sum(f.highl^2)))
  N.Result.df <- cbind(N.Result.df, meant.EF.per)

  #Now use the Friedman's H-Stat to control the effect size and percent to get the amoun
t of the variance explained by the chosen variable without its interaction with other va
```

```
riables
  #Effect Size Controlled
  sqrt(sum((f.j*(1-h.all))^2))
  sqrt(sum((f.j.coast*(1-h.coast))^2))
  sqrt(sum((f.j.midel*(1-h.midel))^2))
  sqrt(sum((f.j.highl*(1-h.highl))^2))

  #Assign the controlled effect size to a dataframe
  meant.EF.cont <- data.frame(EF.cont = sqrt(sum((f.j*(1-h.all))^2)), EF.cont.coast = sq
rt(sum((f.j.coast*(1-h.coast))^2)), EF.cont.midel = sqrt(sum((f.j.midel*(1-h.midel))^2
)), EF.cont.highl = sqrt(sum((f.j.highl*(1-h.highl))^2)))
  N.Result.df <- cbind(N.Result.df, meant.EF.cont)

  #Percent Effect of the Chosen Variable Controlled with Friedman's H-Stat
  #Effect Size Percent Controlled
  sqrt(sum((f.j*(1-h.all))^2) / sum(f.all^2))
  sqrt(sum((f.j.coast*(1-h.coast))^2) / sum(f.coast^2))
  sqrt(sum((f.j.midel*(1-h.midel))^2) / sum(f.midel^2))
  sqrt(sum((f.j.highl*(1-h.highl))^2) / sum(f.highl^2))

  #Assign the controlled percent effect size to a dataframe
  meant.EF.per.cont <- data.frame(EF.per.cont = sqrt(sum((f.j*(1-h.all))^2) / sum(f.all^
2)), EF.per.cont.coast = sqrt(sum((f.j.coast*(1-h.coast))^2) / sum(f.coast^2)), EF.per.c
ont.midel = sqrt(sum((f.j.midel*(1-h.midel))^2) / sum(f.midel^2)), EF.per.cont.highl = s
qrt(sum((f.j.highl*(1-h.highl))^2) / sum(f.highl^2)))
  N.Result.df <- cbind(N.Result.df, meant.EF.per.cont)



  if (k == 1) {
    Var.N.Result.df <- N.Result.df
  }
  else {
  Var.N.Result.df <- rbind(Var.N.Result.df, N.Result.df)
  }
}

#Grab the column averages
Var.avg <- data.frame(row.names = "Precip Mean", t(colMeans(Var.N.Result.df[2:21])))

#Bind the resul to the Total Effect Size dataframe
Total.N.Result.df <- rbind(Total.N.Result.df, Var.avg)
```

Repeat for Precip Seasonality Vector of features to calculate

```
#Choose the variable of interest. This will need to be repeated for each of our predicto
r variables (Temp_Mean, Temp_Mean_Seasonality, Precip_Mean, Precip_Mean_Seasonality, and
Demography.spd)
feature.name <- c("Precip_Mean_Seasonality")
```

Prediction function

```
predictor <- ia$predictor
```

Now get all the interactions for the chosen variable, calculate the Friedman's H-stat (proportion of the effect due to the variable's interaction with the other variables), calulate the effect size as the model sum of squares, discount the effect size based on the Friedman's H-stat, and calculate the effect size a a percent of the variation explained.

```
#iterate the data sampling for interactions 100 times.

runs <- seq(1, 100, 1)

#Make an object for the sampling output to go into
myia <- list(res = data.table(), qr = data.frame())

#Make a datafame object for the results of each iteration
N.Result.df <- data.frame()

#Make an empty dataframe for the total results to append to
Var.N.Result.df <- data.frame()



for (k in runs) {

  myia <- interaction.run.single(
  dataSample = data.sample,
  feature.name = feature.name[1],
  grid.size = grid.size,
  batch.size = batch.size,
  q = myq,
  predictor = predictor
  )


  partial_dat <- myia$res#make a dataframe with the data generated for the interactions
  partial_dat$.y.hat <- myia$qr#add the predicted values to the dataframe
  partial_dat$.class <- 1#specify 1 so when we do the prediction R knows this is a regre
ssion problem

  #collapse into 'F(x)' values - this will provide the average of all of the predictions
for each unique value of the chosen variable. So average of all predictions per observat
ion on grid and per value of the feature.
  pd.all <- data.table::dcast(partial_dat, .feature + .id + elev.factor + .class ~ .typ
e,
                        value.var = ".y.hat", fun.aggregate = mean
)

  pd.all.nona <- pd.all[which(!is.na(pd.all$f)),]#drop NA rows, leaving the 100 aggregat
ed predictions
  pd.all.nona.coast <- pd.all.nona[which(pd.all.nona$elev.factor == "Coastal"),]#Grab on
ly coastal cases.
  pd.all.nona.midel <- pd.all.nona[which(pd.all.nona$elev.factor == "Mid-elevation"),]#G
rab only mid-elevation cases.
  pd.all.nona.highl <- pd.all.nona[which(pd.all.nona$elev.factor == "Highland"),]#Grab o
nly highland cases.

  #Calculate Total Response of Nitrogen
  f.all <- scale(pd.all.nona$f, scale = FALSE)
  f.coast <- scale(pd.all.nona.coast$f, scale = FALSE)
```

```
  f.midel <- scale(pd.all.nona.midel$f, scale = FALSE)
  f.highl <- scale(pd.all.nona.highl$f, scale = FALSE)

  #Calculate Partial Dependence of Nitrogen for Chosen Variable
  f.j <- scale(pd.all.nona$j, scale = FALSE)
  f.j.coast <- scale(pd.all.nona.coast$j, scale = FALSE)
  f.j.midel <- scale(pd.all.nona.midel$j, scale = FALSE)
  f.j.highl <- scale(pd.all.nona.highl$j, scale = FALSE)

  #Calculate Combined Partial Dependence of Nitrogen to all Other Variables
  f.no.j <- scale(pd.all.nona$no.j, scale = FALSE)
  f.noj.coast <- scale(pd.all.nona.coast$no.j, scale = FALSE)
  f.noj.midel <- scale(pd.all.nona.midel$no.j, scale = FALSE)
  f.noj.highl <- scale(pd.all.nona.highl$no.j, scale = FALSE)

  #Calculate Friedman's H-Stat to get the proportion of the effect due to interaction wi
th the other variables
  h.all <- sqrt(sum((f.all - (f.j + f.no.j))^2) / sum(f.all^2))
  h.coast <- sqrt(sum((f.coast - (f.j.coast + f.noj.coast))^2) / sum(f.coast^2))
  h.midel <- sqrt(sum((f.midel - (f.j.midel + f.noj.midel))^2) / sum(f.midel^2))
  h.highl <- sqrt(sum((f.highl - (f.j.highl + f.noj.highl))^2) / sum(f.highl^2))

  #Assign the Friedman's H-Stat to a dataframe
  N.Result.df <- data.frame(var = "Precip Mean Seasonality", H.Stat = h.all, H.Stat.Coas
t = h.coast, H.Stat.Mid = h.midel, H.Stat.High = h.highl)

  #Model Effect Size of the Chosen Variable (Amount of the per mil variance explained by
the chosen variable)
  sqrt(sum((f.j)^2))
  sqrt(sum((f.j.coast)^2))
  sqrt(sum((f.j.midel)^2))
  sqrt(sum((f.j.highl)^2))

  #Add the effect size to the dataframe
  meant.EF <- data.frame(EF = sqrt(sum((f.j)^2)), EF.coast = sqrt(sum((f.j.coast)^2)), E
F.midel = sqrt(sum((f.j.midel)^2)), EF.high = sqrt(sum((f.j.highl)^2)))
  N.Result.df <- cbind(N.Result.df, meant.EF)

  #Percent Effect of the Chosen Variable (Percent of the variance in Nitrogen explained
 by the chosen variable)
  sqrt(sum(f.j^2) / sum(f.all^2))
  sqrt(sum(f.j.coast^2) / sum(f.coast^2))
  sqrt(sum(f.j.midel^2) / sum(f.midel^2))
  sqrt(sum(f.j.highl^2) / sum(f.highl^2))

  #Assign the percent effect size to the dataframe
  meant.EF.per <- data.frame(EF.per = sqrt(sum(f.j^2) / sum(f.all^2)), EF.per.coast = sq
rt(sum(f.j.coast^2) / sum(f.coast^2)), EF.per.midel = sqrt(sum(f.j.midel^2) / sum(f.mide
l^2)), EF.per.high = sqrt(sum(f.j.highl^2) / sum(f.highl^2)))
  N.Result.df <- cbind(N.Result.df, meant.EF.per)

  #Now use the Friedman's H-Stat to control the effect size and percent to get the amoun
```

```
t of the variance explained by the chosen variable without its interaction with other va
riables
  #Effect Size Controlled
  sqrt(sum((f.j*(1-h.all))^2))
  sqrt(sum((f.j.coast*(1-h.coast))^2))
  sqrt(sum((f.j.midel*(1-h.midel))^2))
  sqrt(sum((f.j.highl*(1-h.highl))^2))

  #Assign the controlled effect size to a dataframe
  meant.EF.cont <- data.frame(EF.cont = sqrt(sum((f.j*(1-h.all))^2)), EF.cont.coast = sq
rt(sum((f.j.coast*(1-h.coast))^2)), EF.cont.midel = sqrt(sum((f.j.midel*(1-h.midel))^2
)), EF.cont.highl = sqrt(sum((f.j.highl*(1-h.highl))^2)))
  N.Result.df <- cbind(N.Result.df, meant.EF.cont)

  #Percent Effect of the Chosen Variable Controlled with Friedman's H-Stat
  #Effect Size Percent Controlled
  sqrt(sum((f.j*(1-h.all))^2) / sum(f.all^2))
  sqrt(sum((f.j.coast*(1-h.coast))^2) / sum(f.coast^2))
  sqrt(sum((f.j.midel*(1-h.midel))^2) / sum(f.midel^2))
  sqrt(sum((f.j.highl*(1-h.highl))^2) / sum(f.highl^2))

  #Assign the controlled percent effect size to a dataframe
  meant.EF.per.cont <- data.frame(EF.per.cont = sqrt(sum((f.j*(1-h.all))^2) / sum(f.all^
2)), EF.per.cont.coast = sqrt(sum((f.j.coast*(1-h.coast))^2) / sum(f.coast^2)), EF.per.c
ont.midel = sqrt(sum((f.j.midel*(1-h.midel))^2) / sum(f.midel^2)), EF.per.cont.highl = s
qrt(sum((f.j.highl*(1-h.highl))^2) / sum(f.highl^2)))
  N.Result.df <- cbind(N.Result.df, meant.EF.per.cont)


  if (k == 1) {
    Var.N.Result.df <- N.Result.df
  }
  else {
  Var.N.Result.df <- rbind(Var.N.Result.df, N.Result.df)
  }
}


#Grab the column averages
Var.avg <- data.frame(row.names = "Precip Mean Seasonality", t(colMeans(Var.N.Result.df[
2:21])))

#Bind the resul to the Total Effect Size dataframe
Total.N.Result.df <- rbind(Total.N.Result.df, Var.avg)
```

Repeat for Temp Seasonality Vector of features to calculate

```
#Choose the variable of interest. This will need to be repeated for each of our predicto
r variables (Temp_Mean, Temp_Mean_Seasonality, Precip_Mean, Precip_Mean_Seasonality, and
Demography.spd)
feature.name <- c("Temp_Mean_Seasonality")
```

Prediction function

```
predictor <- ia$predictor
```

Now get all the interactions for the chosen variable, calculate the Friedman's H-stat (proportion of the effect due to the variable's interaction with the other variables), calulate the effect size as the model sum of squares, discount the effect size based on the Friedman's H-stat, and calculate the effect size a a percent of the variation explained.

```
#iterate the data sampling for interactions 100 times.

runs <- seq(1, 100, 1)

#Make an object for the sampling output to go into
myia <- list(res = data.table(), qr = data.frame())

#Make a datafame object for the results of each iteration
N.Result.df <- data.frame()

#Make an empty dataframe for the total results to append to
Var.N.Result.df <- data.frame()



for (k in runs) {

  myia <- interaction.run.single(
  dataSample = data.sample,
  feature.name = feature.name[1],
  grid.size = grid.size,
  batch.size = batch.size,
  q = myq,
  predictor = predictor
  )


  partial_dat <- myia$res#make a dataframe with the data generated for the interactions
  partial_dat$.y.hat <- myia$qr#add the predicted values to the dataframe
  partial_dat$.class <- 1#specify 1 so when we do the prediction R knows this is a regre
ssion problem

  #collapse into 'F(x)' values - this will provide the average of all of the predictions
for each unique value of the chosen variable. So average of all predictions per observat
ion on grid and per value of the feature.
  pd.all <- data.table::dcast(partial_dat, .feature + .id + elev.factor + .class ~ .typ
e,
                        value.var = ".y.hat", fun.aggregate = mean
)

  pd.all.nona <- pd.all[which(!is.na(pd.all$f)),]#drop NA rows, leaving the 100 aggregat
ed predictions
  pd.all.nona.coast <- pd.all.nona[which(pd.all.nona$elev.factor == "Coastal"),]#Grab on
ly coastal cases.
  pd.all.nona.midel <- pd.all.nona[which(pd.all.nona$elev.factor == "Mid-elevation"),]#G
rab only mid-elevation cases.
  pd.all.nona.highl <- pd.all.nona[which(pd.all.nona$elev.factor == "Highland"),]#Grab o
nly highland cases.

  #Calculate Total Response of Nitrogen
  f.all <- scale(pd.all.nona$f, scale = FALSE)
  f.coast <- scale(pd.all.nona.coast$f, scale = FALSE)
```

```
  f.midel <- scale(pd.all.nona.midel$f, scale = FALSE)
  f.highl <- scale(pd.all.nona.highl$f, scale = FALSE)

  #Calculate Partial Dependence of Nitrogen for Chosen Variable
  f.j <- scale(pd.all.nona$j, scale = FALSE)
  f.j.coast <- scale(pd.all.nona.coast$j, scale = FALSE)
  f.j.midel <- scale(pd.all.nona.midel$j, scale = FALSE)
  f.j.highl <- scale(pd.all.nona.highl$j, scale = FALSE)

  #Calculate Combined Partial Dependence of Nitrogen to all Other Variables
  f.no.j <- scale(pd.all.nona$no.j, scale = FALSE)
  f.noj.coast <- scale(pd.all.nona.coast$no.j, scale = FALSE)
  f.noj.midel <- scale(pd.all.nona.midel$no.j, scale = FALSE)
  f.noj.highl <- scale(pd.all.nona.highl$no.j, scale = FALSE)

  #Calculate Friedman's H-Stat to get the proportion of the effect due to interaction wi
th the other variables
  h.all <- sqrt(sum((f.all - (f.j + f.no.j))^2) / sum(f.all^2))
  h.coast <- sqrt(sum((f.coast - (f.j.coast + f.noj.coast))^2) / sum(f.coast^2))
  h.midel <- sqrt(sum((f.midel - (f.j.midel + f.noj.midel))^2) / sum(f.midel^2))
  h.highl <- sqrt(sum((f.highl - (f.j.highl + f.noj.highl))^2) / sum(f.highl^2))

  #Assign the Friedman's H-Stat to a dataframe
  N.Result.df <- data.frame(var = "Temp Mean Seasonality", H.Stat = h.all, H.Stat.Coast
 = h.coast, H.Stat.Mid = h.midel, H.Stat.High = h.highl)

  #Model Effect Size of the Chosen Variable (Amount of the per mil variance explained by
the chosen variable)
  sqrt(sum((f.j)^2))
  sqrt(sum((f.j.coast)^2))
  sqrt(sum((f.j.midel)^2))
  sqrt(sum((f.j.highl)^2))

  #Add the effect size to the dataframe
  meant.EF <- data.frame(EF = sqrt(sum((f.j)^2)), EF.coast = sqrt(sum((f.j.coast)^2)), E
F.midel = sqrt(sum((f.j.midel)^2)), EF.high = sqrt(sum((f.j.highl)^2)))
  N.Result.df <- cbind(N.Result.df, meant.EF)

  #Percent Effect of the Chosen Variable (Percent of the variance in Nitrogen explained
 by the chosen variable)
  sqrt(sum(f.j^2) / sum(f.all^2))
  sqrt(sum(f.j.coast^2) / sum(f.coast^2))
  sqrt(sum(f.j.midel^2) / sum(f.midel^2))
  sqrt(sum(f.j.highl^2) / sum(f.highl^2))

  #Assign the percent effect size to the dataframe
  meant.EF.per <- data.frame(EF.per = sqrt(sum(f.j^2) / sum(f.all^2)), EF.per.coast = sq
rt(sum(f.j.coast^2) / sum(f.coast^2)), EF.per.midel = sqrt(sum(f.j.midel^2) / sum(f.mide
l^2)), EF.per.high = sqrt(sum(f.j.highl^2) / sum(f.highl^2)))
  N.Result.df <- cbind(N.Result.df, meant.EF.per)

  #Now use the Friedman's H-Stat to control the effect size and percent to get the amoun
```

```
t of the variance explained by the chosen variable without its interaction with other va
riables
  #Effect Size Controlled
  sqrt(sum((f.j*(1-h.all))^2))
  sqrt(sum((f.j.coast*(1-h.coast))^2))
  sqrt(sum((f.j.midel*(1-h.midel))^2))
  sqrt(sum((f.j.highl*(1-h.highl))^2))

  #Assign the controlled effect size to a dataframe
  meant.EF.cont <- data.frame(EF.cont = sqrt(sum((f.j*(1-h.all))^2)), EF.cont.coast = sq
rt(sum((f.j.coast*(1-h.coast))^2)), EF.cont.midel = sqrt(sum((f.j.midel*(1-h.midel))^2
)), EF.cont.highl = sqrt(sum((f.j.highl*(1-h.highl))^2)))
  N.Result.df <- cbind(N.Result.df, meant.EF.cont)

  #Percent Effect of the Chosen Variable Controlled with Friedman's H-Stat
  #Effect Size Percent Controlled
  sqrt(sum((f.j*(1-h.all))^2) / sum(f.all^2))
  sqrt(sum((f.j.coast*(1-h.coast))^2) / sum(f.coast^2))
  sqrt(sum((f.j.midel*(1-h.midel))^2) / sum(f.midel^2))
  sqrt(sum((f.j.highl*(1-h.highl))^2) / sum(f.highl^2))

  #Assign the controlled percent effect size to a dataframe
  meant.EF.per.cont <- data.frame(EF.per.cont = sqrt(sum((f.j*(1-h.all))^2) / sum(f.all^
2)), EF.per.cont.coast = sqrt(sum((f.j.coast*(1-h.coast))^2) / sum(f.coast^2)), EF.per.c
ont.midel = sqrt(sum((f.j.midel*(1-h.midel))^2) / sum(f.midel^2)), EF.per.cont.highl = s
qrt(sum((f.j.highl*(1-h.highl))^2) / sum(f.highl^2)))
  N.Result.df <- cbind(N.Result.df, meant.EF.per.cont)


  if (k == 1) {
    Var.N.Result.df <- N.Result.df
  }
  else {
  Var.N.Result.df <- rbind(Var.N.Result.df, N.Result.df)
  }
}


#Grab the column averages (this will be the 100 runs of 100 different var values average
d together)
Var.avg <- data.frame(row.names = "Temp Mean Seasonality", t(colMeans(Var.N.Result.df[2:
21])))

#Bind the resul to the Total Effect Size dataframe
Total.N.Result.df <- rbind(Total.N.Result.df, Var.avg)

#Generate the Cumulative Climate Effect
Cum.Clim. <- data.frame(row.names = "Cumulative Climate", t(colSums(Total.N.Result.df)))

#Add Cumulative Climate to the Total Effect Size Dataframe
Total.N.Result.df <- rbind(Total.N.Result.df, Cum.Clim.)
```

```
#Pull Out the Key Effect Sizes to store in their own dataframe
Cumulative.EF <- data.frame(Elev.cat = c("Coastal", "Mid-elevation", "Highland"), Cum.Cl
im.EF = c(Total.N.Result.df[5,14], Total.N.Result.df[5,15], Total.N.Result.df[5,16]), Cu
m.Clim.EF.percent = c(Total.N.Result.df[5,18], Total.N.Result.df[5,19], Total.N.Result.d
f[5,20]))
```

Repeat for Demography Vector of features to calculate

```
#Choose the variable of interest. This will need to be repeated for each of our predicto
r variables (Temp_Mean, Temp_Mean_Seasonality, Precip_Mean, Precip_Mean_Seasonality, and
Demography.spd)
feature.name <- c("Demography.kde.14c")
```

Prediction function

```
predictor <- ia$predictor
```

Now get all the interactions for the chosen variable, calculate the Friedman's H-stat (proportion of the effect due to the variable's interaction with the other variables), calulate the effect size as the model sum of squares, discount the effect size based on the Friedman's H-stat, and calculate the effect size a a percent of the variation explained.

```
#iterate the data sampling for interactions 100 times.

runs <- seq(1, 100, 1)

#Make an object for the sampling output to go into
myia <- list(res = data.table(), qr = data.frame())

#Make a datafame object for the results of each iteration
N.Result.df <- data.frame()

#Make an empty dataframe for the total results to append to
Var.N.Result.df <- data.frame()


for (k in runs) {

  myia <- interaction.run.single(
  dataSample = data.sample,
  feature.name = feature.name[1],
  grid.size = grid.size,
  batch.size = batch.size,
  q = myq,
  predictor = predictor
  )



  partial_dat <- myia$res#make a dataframe with the data generated for the interactions
  partial_dat$.y.hat <- myia$qr#add the predicted values to the dataframe
  partial_dat$.class <- 1#specify 1 so when we do the prediction R knows this is a regre
ssion problem

  #collapse into 'F(x)' values - this will provide the average of all of the predictions
for each unique value of the chosen variable. So average of all predictions per observat
ion on grid and per value of the feature.
  pd.all <- data.table::dcast(partial_dat, .feature + .id + elev.factor + .class ~ .typ
e,
                        value.var = ".y.hat", fun.aggregate = mean
)

  pd.all.nona <- pd.all[which(!is.na(pd.all$f)),]#drop NA rows, leaving the 100 aggregat
ed predictions
  pd.all.nona.coast <- pd.all.nona[which(pd.all.nona$elev.factor == "Coastal"),]#Grab on
ly coastal cases.
  pd.all.nona.midel <- pd.all.nona[which(pd.all.nona$elev.factor == "Mid-elevation"),]#G
rab only mid-elevation cases.
  pd.all.nona.highl <- pd.all.nona[which(pd.all.nona$elev.factor == "Highland"),]#Grab o
nly highland cases.

  #Calculate Total Response of Nitrogen
  f.all <- scale(pd.all.nona$f, scale = FALSE)
  f.coast <- scale(pd.all.nona.coast$f, scale = FALSE)
  f.midel <- scale(pd.all.nona.midel$f, scale = FALSE)
```

```
  f.highl <- scale(pd.all.nona.highl$f, scale = FALSE)

  #Calculate Partial Dependence of Nitrogen for Chosen Variable
  f.j <- scale(pd.all.nona$j, scale = FALSE)
  f.j.coast <- scale(pd.all.nona.coast$j, scale = FALSE)
  f.j.midel <- scale(pd.all.nona.midel$j, scale = FALSE)
  f.j.highl <- scale(pd.all.nona.highl$j, scale = FALSE)

  #Calculate Combined Partial Dependence of Nitrogen to all Other Variables
  f.no.j <- scale(pd.all.nona$no.j, scale = FALSE)
  f.noj.coast <- scale(pd.all.nona.coast$no.j, scale = FALSE)
  f.noj.midel <- scale(pd.all.nona.midel$no.j, scale = FALSE)
  f.noj.highl <- scale(pd.all.nona.highl$no.j, scale = FALSE)

  #Calculate Friedman's H-Stat to get the proportion of the effect due to interaction wi
th the other variables
  h.all <- sqrt(sum((f.all - (f.j + f.no.j))^2) / sum(f.all^2))
  h.coast <- sqrt(sum((f.coast - (f.j.coast + f.noj.coast))^2) / sum(f.coast^2))
  h.midel <- sqrt(sum((f.midel - (f.j.midel + f.noj.midel))^2) / sum(f.midel^2))
  h.highl <- sqrt(sum((f.highl - (f.j.highl + f.noj.highl))^2) / sum(f.highl^2))

  #Assign the Friedman's H-Stat to a dataframe
  N.Result.df <- data.frame(var = "Demography", H.Stat = h.all, H.Stat.Coast = h.coast,
 H.Stat.Mid = h.midel, H.Stat.High = h.highl)

  #Model Effect Size of the Chosen Variable (Amount of the per mil variance explained by
the chosen variable)
  sqrt(sum((f.j)^2))
  sqrt(sum((f.j.coast)^2))
  sqrt(sum((f.j.midel)^2))
  sqrt(sum((f.j.highl)^2))

  #Add the effect size to the dataframe
  meant.EF <- data.frame(EF = sqrt(sum((f.j)^2)), EF.coast = sqrt(sum((f.j.coast)^2)), E
F.midel = sqrt(sum((f.j.midel)^2)), EF.high = sqrt(sum((f.j.highl)^2)))
  N.Result.df <- cbind(N.Result.df, meant.EF)

  #Percent Effect of the Chosen Variable (Percent of the variance in Nitrogen explained
 by the chosen variable)
  sqrt(sum(f.j^2) / sum(f.all^2))
  sqrt(sum(f.j.coast^2) / sum(f.coast^2))
  sqrt(sum(f.j.midel^2) / sum(f.midel^2))
  sqrt(sum(f.j.highl^2) / sum(f.highl^2))

  #Assign the percent effect size to the dataframe
  meant.EF.per <- data.frame(EF.per = sqrt(sum(f.j^2) / sum(f.all^2)), EF.per.coast = sq
rt(sum(f.j.coast^2) / sum(f.coast^2)), EF.per.midel = sqrt(sum(f.j.midel^2) / sum(f.mide
l^2)), EF.per.high = sqrt(sum(f.j.highl^2) / sum(f.highl^2)))
  N.Result.df <- cbind(N.Result.df, meant.EF.per)

  #Now use the Friedman's H-Stat to control the effect size and percent to get the amoun
t of the variance explained by the chosen variable without its interaction with other va
```

```
riables
  #Effect Size Controlled
  sqrt(sum((f.j*(1-h.all))^2))
  sqrt(sum((f.j.coast*(1-h.coast))^2))
  sqrt(sum((f.j.midel*(1-h.midel))^2))
  sqrt(sum((f.j.highl*(1-h.highl))^2))

  #Assign the controlled effect size to a dataframe
  meant.EF.cont <- data.frame(EF.cont = sqrt(sum((f.j*(1-h.all))^2)), EF.cont.coast = sq
rt(sum((f.j.coast*(1-h.coast))^2)), EF.cont.midel = sqrt(sum((f.j.midel*(1-h.midel))^2
)), EF.cont.highl = sqrt(sum((f.j.highl*(1-h.highl))^2)))
  N.Result.df <- cbind(N.Result.df, meant.EF.cont)

  #Percent Effect of the Chosen Variable Controlled with Friedman's H-Stat
  #Effect Size Percent Controlled
  sqrt(sum((f.j*(1-h.all))^2) / sum(f.all^2))
  sqrt(sum((f.j.coast*(1-h.coast))^2) / sum(f.coast^2))
  sqrt(sum((f.j.midel*(1-h.midel))^2) / sum(f.midel^2))
  sqrt(sum((f.j.highl*(1-h.highl))^2) / sum(f.highl^2))

  #Assign the controlled percent effect size to a dataframe
  meant.EF.per.cont <- data.frame(EF.per.cont = sqrt(sum((f.j*(1-h.all))^2) / sum(f.all^
2)), EF.per.cont.coast = sqrt(sum((f.j.coast*(1-h.coast))^2) / sum(f.coast^2)), EF.per.c
ont.midel = sqrt(sum((f.j.midel*(1-h.midel))^2) / sum(f.midel^2)), EF.per.cont.highl = s
qrt(sum((f.j.highl*(1-h.highl))^2) / sum(f.highl^2)))
  N.Result.df <- cbind(N.Result.df, meant.EF.per.cont)


  if (k == 1) {
    Var.N.Result.df <- N.Result.df
  }
  else {
  Var.N.Result.df <- rbind(Var.N.Result.df, N.Result.df)
  }
}


#Grab the column averages (this will be the 100 runs of 100 different var values average
d together)
Var.avg <- data.frame(row.names = "Demography", t(colMeans(Var.N.Result.df[2:21])))

#Bind the resul to the Total Effect Size dataframe
Total.N.Result.df <- rbind(Total.N.Result.df, Var.avg)

#Add demography results to the cumulative effect size dataframe
Cumulative.EF$Dem.EF <- c(sqrt(sum((f.j.coast*(1-h.coast))^2)), sqrt(sum((f.j.midel*(1-
h.midel))^2)), sqrt(sum((f.j.highl*(1-h.highl))^2)))
Cumulative.EF$Dem.EF.percent <- c(sqrt(sum((f.j.coast*(1-h.coast))^2) / sum(f.coast^2)),
sqrt(sum((f.j.midel*(1-h.midel))^2) / sum(f.midel^2)), sqrt(sum((f.j.highl*(1-h.highl))^
2) / sum(f.highl^2)))
```

Show the complete set of effect size calculations

```
#show the complete set of effect size calculations - for each variable, this is: H-stat,
Effect Size, Effect Size percent, Effect size controlled by H-stat, Effect size percent
 controlled by H-stat
round(Total.N.Result.df, 2)
```

```
##                           H.Stat H.Stat.Coast H.Stat.Mid H.Stat.High     EF
## Temp Mean                   0.55         0.25       0.31        0.49  23.17
## Precip Mean                 0.44         0.26       0.25        0.37  29.66
## Precip Mean Seasonality     0.72         0.24       0.15        0.22  27.83
## Temp Mean Seasonality       0.52         0.25       0.35        0.46  25.49
## Cumulative Climate          2.23         1.01       1.06        1.53 106.16
## Demography                  0.64         0.21       0.29        0.47  26.18
##                           EF.coast EF.midel EF.high EF.per EF.per.coast
## Temp Mean                     1.90     1.03    0.75   0.57         0.08
## Precip Mean                  13.89     7.03    0.84   0.73         0.54
## Precip Mean Seasonality       1.61     1.11    0.93   0.68         0.06
## Temp Mean Seasonality         8.63     3.10    0.78   0.62         0.34
## Cumulative Climate           26.03    12.26    3.30   2.60         1.03
## Demography                    2.08     5.43    1.02   0.65         0.08
##                           EF.per.midel EF.per.high EF.cont EF.cont.coast
## Temp Mean                         0.06        0.16   10.37          1.42
## Precip Mean                       0.40        0.17   16.55         10.25
## Precip Mean Seasonality           0.06        0.20    7.84          1.22
## Temp Mean Seasonality             0.18        0.17   12.27          6.50
## Cumulative Climate                0.70        0.70   47.03         19.38
## Demography                        0.31        0.21    9.51          1.63
##                           EF.cont.midel EF.cont.highl EF.per.cont
## Temp Mean                          0.71          0.39        0.25
## Precip Mean                        5.27          0.53        0.40
## Precip Mean Seasonality            0.95          0.73        0.19
## Temp Mean Seasonality              2.03          0.43        0.30
## Cumulative Climate                 8.96          2.08        1.14
## Demography                         3.84          0.54        0.23
##                           EF.per.cont.coast EF.per.cont.midel EF.per.cont.highl
## Temp Mean                              0.06              0.04              0.08
## Precip Mean                            0.40              0.30              0.11
## Precip Mean Seasonality                0.05              0.05              0.15
## Temp Mean Seasonality                  0.26              0.12              0.09
## Cumulative Climate                     0.76              0.51              0.44
## Demography                             0.07              0.22              0.11
```

Table S1. Complete Effect Size Calcuations for δ^{15}N‰. For each variable we report the complete model H-stat and effect size as well as the H-stat and effect sizes within each elevational zone.

```
N.Results <- Total.N.Result.df[,c(14:16, 18:20)]
round(N.Results, 4)
```

```
##                           EF.cont.coast EF.cont.midel EF.cont.highl
## Temp Mean                        1.4166        0.7101        0.3855
## Precip Mean                     10.2469        5.2653        0.5339
## Precip Mean Seasonality          1.2184        0.9456        0.7300
## Temp Mean Seasonality            6.4959        2.0348        0.4282
## Cumulative Climate              19.3779        8.9558        2.0776
## Demography                       1.6332        3.8402        0.5444
##                           EF.per.cont.coast EF.per.cont.midel EF.per.cont.highl
## Temp Mean                            0.0562            0.0409            0.0846
## Precip Mean                          0.4004            0.2987            0.1097
## Precip Mean Seasonality              0.0484            0.0549            0.1541
## Temp Mean Seasonality                0.2573            0.1175            0.0927
## Cumulative Climate                   0.7624            0.5120            0.4412
## Demography                           0.0659            0.2206            0.1121
```

Table 1 Part 1. δ^{15}N‰ Effect Sizes. *Note: as these results are generated through many iterations the results will not exactly match those in the main manuscript text. Despite small fluctuations the ranking of variables and effect sizes do not change significantly when re-running the code.*

Now we repeat all of the above for δ^{13}C‰.

To begin, subset the dataframe for just the variables included in the RF model.

```
dat.C <- data.C %>%
  dplyr::select(C.b.coll.,
         Temp_Mean,
         Precip_Mean,
         Temp_Mean_Seasonality,
         Precip_Mean_Seasonality,
         elev.factor,
         Demography.kde.14c)
```

Now run RF regression to determine how well our suite of 6 variables predict δ^{13}C‰.

```
set.seed(33)#set a seed for replicability
#examine how bone C values vary as a function of all other variables in the data
RF.C <- ranger(C.b.coll. ~ ., dat.C)#Use RF regression to predict nitrogen values using
 each of the predictor variables

print(RF.C)
```

```
## Ranger result
##
## Call:
##  ranger(C.b.coll. ~ ., dat.C)
##
## Type:                             Regression
## Number of trees:                  500
## Sample size:                      1761
## Number of independent variables:  6
## Mtry:                             2
## Target node size:                 5
## Variable importance mode:         none
## Splitrule:                        variance
## OOB prediction error (MSE):       2.595212
## R squared (OOB):                  0.6637098
```

The RF model appears to do well at explaining the variance in bone collagen δ^{13}C‰. For validation purposes, we run multiple model performance evaluators below.

First, to better estimate how accurately our model predicts δ^{13}C‰, we use cross-validation following (149, 150, 151) to obtain the variance explained and root mean square error (RMSE).

```
myvars.C <- c("elev.factor", "Temp_Mean", "Precip_Mean", "Temp_Mean_Seasonality", "Precip_Mean_Seasonality", "Demography.kde.14c")#select the key explanatory variables we are using in our analysis

pred.vars.C <- dat.C[myvars.C]#subset the dat.C dataframe to keep only the predictor variables

set.seed(33)
RF.C.cv <- rgcv(pred.vars.C, dat.C$C.b.coll., cv.fold = 10)
RF.C.cv$rmse #root mean square error of cross-validated model
```

```
## [1] 1.619009
```

```
RF.C.cv$vecv #variance explained by the model based on cross-validation
```

```
## [1] 66.01505
```

Results show that the median permuted percent variance explained is 66.02 meaning we explain 66.02% of the variability in bone collagen δ^{13}C‰ with our predictor variables. Mean RMSE is 1.61.

Next we check the model residuals for normalcy, temporal, and spatial autcorrelation. Plot δ^{13}C‰ model residuals to check for normalcy:

```
pred.C <-  pred(RF.C, dat.C) #Use the pred function to predict the Nitrogen value for ea
ch individual

par(pty="s")

RF.resid.C <- dat.C$C.b.coll. - pred.C #calculate the residuals
colnames(RF.resid.C) <- "Residuals"

hist(RF.resid.C, col="grey", main=NA, xlab="Residuals")
```

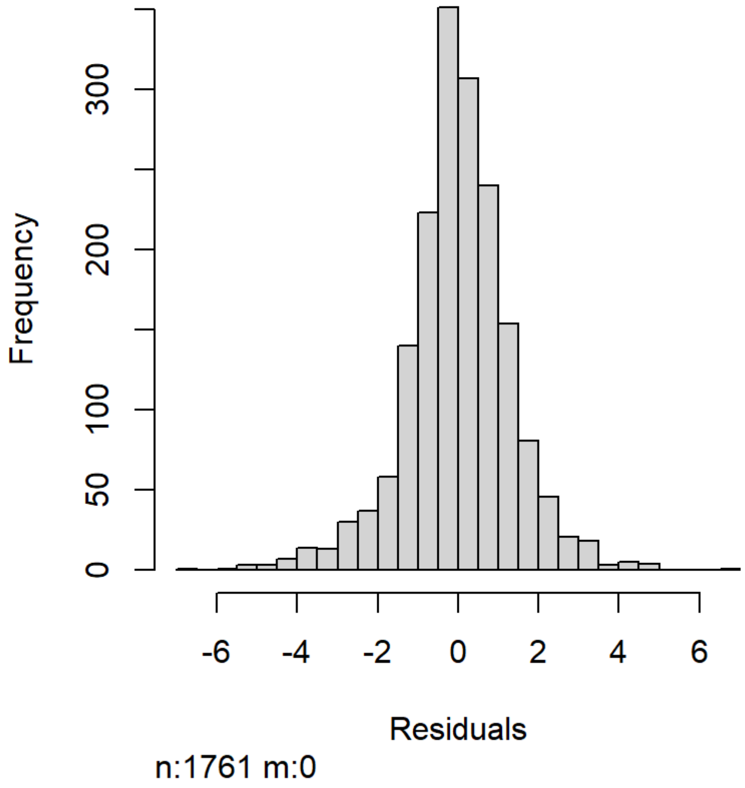

Fig. S8. δ^{13}C‰ RF Model Residuals. Residuals are normally distributed around 0.

Check for temporal autocorrelation in the δ^{13}C‰ residuals, following “Time after time: calculating the autocorrelation function for uneven or grouped time series” by Ariel Muldoon https://aosmith.rbind.io/2018/06/27/uneven-grouped-autocorrelation/ (https://aosmith.rbind.io/2018/06/27/uneven-grouped-autocorrelation/).

```
#Add the median year to the residuals dataframe for use in ordering individuals
RF.resid.C$median.date <- data.C$median.year

#Arrange the individuals by their median date
RF.resid.C.dat <- RF.resid.C %>% arrange(median.date)

#For individuals who have the same median year, aggregate their residuals into one obser
vation by taking the mean of each year with repeated individuals.
RF.resid.C.dat <- aggregate(RF.resid.C.dat, by = list(RF.resid.C.dat$median.date), FUN =
mean)

#Pad the dataset with NAs so each elevation zone has an equal number of rows. We want 1
 row per year from the minimum median date to the maximum median date across the full te
mporal spread. This is 385 to 5941 yBP.
RF.resid.C.dat.expand <- RF.resid.C.dat %>%
  complete(median.date = 385:5941)

corr <- acf(RF.resid.C.dat.expand$Residuals, lag.max=300,type="correlation",plot=TRUE,n
a.action=na.pass, main = "", xlab = "Lag (yrs)")#generate an acf plot of the residuals t
o visually assess temporal autocorrelation
mtext("All Individuals with Carbon", side = 3, line = 0.5)
```

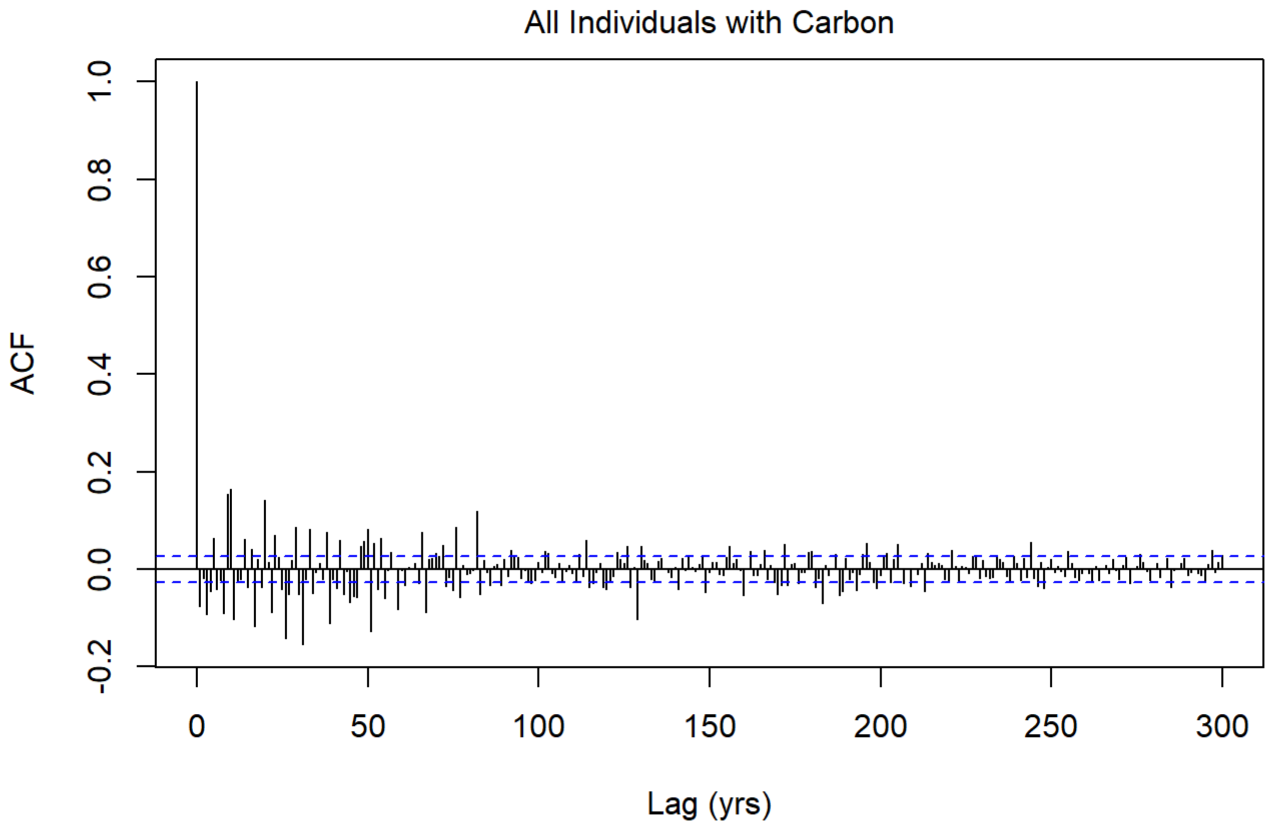

Fig. S9. Temporal Autocorellation ACF Plot. ACF plot shows there is some temporal autocorrelation in the residuals up to about 200 years. This is not surprising given the way dates are assigned to individuals. The autocorrelation is not overly patterned but may exert some influence.

Check for Spatial Autocorrelation within the residuals.

```
#make a distance matrix
spat.dist <- as.matrix(dist(cbind(data.C$Latitude, data.C$Longitude)))
spat.dist.inv <- 1/spat.dist #inverse distance matrix
spat.dist.inv[is.infinite(spat.dist.inv)] <- 0 #set any infinate values to zero

#Check for spatial autocorrelation using global Moran's I
data.frame("Coordinates"=round(unlist(c(Moran.I(RF.resid.C$Residuals, spat.dist.inv, na.
rm=T))), 4)
           )
```

```
##          Coordinates
## observed     -0.0009
## expected     -0.0006
## sd            0.0029
## p.value       0.9063
```

We fail to reject the null hypothesis, suggesting that we do not have residual spatial autocorrelation influencing the results.

We evaluate the influence of each variable within each elevational cateogry by calcualting the effect size as the model sum of squares, discounted by the proportion of the variable's effect that results from its interaction with the other variables - the same as for δ^{15}N‰.

Make a predictor to use with the IML package:

```
mod <- Predictor$new(RF.C,
                     data = dat.C[-which(names(dat.C) == "C.b.coll.")],
                     y = dat.C$C.b.coll.,
                     predict.function = pred)
```

Calculate Friedman's H-Stat using IML. This is the proportion of the effect of each variable due to its interaction with the other variables for the complete model (not subset by elevational zone).

```
#Friedman's H-Stat proportion of the effect of each variable due to its interaction with
the others.
ia <- Interaction$new(mod)
ia
```

```
## Interpretation method:  Interaction
##
##
## Analysed predictor:
## Prediction task: unknown
##
##
## Analysed data:
## Sampling from data.frame with 1761 rows and 6 columns.
##
## Head of results:
##                   .feature .interaction
## 1:               Temp_Mean    0.2303248
## 2:             Precip_Mean    0.2486678
## 3:   Temp_Mean_Seasonality    0.2646568
## 4: Precip_Mean_Seasonality    0.2941704
## 5:             elev.factor    0.2893889
## 6:      Demography.kde.14c    0.2150809
```

Note that this is the Friedman's H-stat for the total model, not within each elevational category. We are interested in how the variables predict δ^{13}C‰ within each of the elevational zones. To get the interaction and effect sizes within each elevational category, we take the below steps.

First, grab the data set

```
#This code grabs all of the input data for our RF model. This is the complete set of cas
es from our raw data (in other words, all 1784 individuals with nitrogen on bone collage
n)
data.sample <- ia$.__enclos_env__$private$sampler$get.x()
```

Second, set the batch size for processing (1000)

```
batch.size <- ia$predictor$batch.size
```

Third, set the grid size for calculating Partial Dependencies

```
grid.size <- ia$grid.size
grid.size <- 100
```

Fourth, set the mean function (required

```
myq <- ia$.__enclos_env__$private$q
```

Fifth, choose which predictor variable to calculate

```
#Choose the variable of interest. This will need to be repeated for each of our predicto
r variables (Temp_Mean, Temp_Mean_Seasonality, Precip_Mean, Precip_Mean_Seasonality, and
Demography.spd)
feature.name <- c("Temp_Mean")
```

Sixth, set the prediction function

```
predictor <- ia$predictor
```

Seventh, get all the interactions for the chosen variable, calculate the Friedman's H-stat (proportion of the effect due to the variable's interaction with the other variables), calulate the effect size as the model sum of squares, discount the effect size based on the Friedman's H-stat, and calculate the effect size a a percent of the variation explained. For additional information on Friedman's H-stat, see (124). *Note: this code may take a long time (~10+ mins per variable) to run.*

```
#iterate the data sampling for interactions 100 times.
runs <- seq(1, 100, 1)

#Make an object for the sampling output to go into
myia <- list(res = data.table(), qr = data.frame())

#Make a datafame object for the results of each iteration
C.Result.df <- data.frame()

#Make an empty dataframe for the total results to append to
Var.C.Result.df <- data.frame()


for (k in runs) {

  myia <- interaction.run.single(
  dataSample = data.sample,
  feature.name = feature.name[1],
  grid.size = grid.size,
  batch.size = batch.size,
  q = myq,
  predictor = predictor
  )


  partial_dat <- myia$res#make a dataframe with the data generated for the interactions
  partial_dat$.y.hat <- myia$qr#add the predicted values to the dataframe
  partial_dat$.class <- 1#specify 1 so when we do the prediction R knows this is a regre
ssion problem

  #collapse into 'F(x)' values - this will provide the average of all of the predictions
for each unique value of the chosen variable. So average of all predictions per observat
ion on grid and per value of the feature.
  pd.all <- data.table::dcast(partial_dat, .feature + .id + elev.factor + .class ~ .typ
e,
                        value.var = ".y.hat", fun.aggregate = mean
)

  pd.all.nona <- pd.all[which(!is.na(pd.all$f)),]#drop NA rows, leaving the 100 aggregat
ed predictions
  pd.all.nona.coast <- pd.all.nona[which(pd.all.nona$elev.factor == "Coastal"),]#Grab on
ly coastal cases.
  pd.all.nona.midel <- pd.all.nona[which(pd.all.nona$elev.factor == "Mid-elevation"),]#G
rab only mid-elevation cases.
  pd.all.nona.highl <- pd.all.nona[which(pd.all.nona$elev.factor == "Highland"),]#Grab o
nly highland cases.

  #Calculate Total Response of Carbon
  f.all <- scale(pd.all.nona$f, scale = FALSE)
  f.coast <- scale(pd.all.nona.coast$f, scale = FALSE)
  f.midel <- scale(pd.all.nona.midel$f, scale = FALSE)
  f.highl <- scale(pd.all.nona.highl$f, scale = FALSE)
```

```
  #Calculate Partial Dependence of Carbon for Chosen Variable
  f.j <- scale(pd.all.nona$j, scale = FALSE)
  f.j.coast <- scale(pd.all.nona.coast$j, scale = FALSE)
  f.j.midel <- scale(pd.all.nona.midel$j, scale = FALSE)
  f.j.highl <- scale(pd.all.nona.highl$j, scale = FALSE)

  #Calculate Combined Partial Dependence of Carbon to all Other Variables
  f.no.j <- scale(pd.all.nona$no.j, scale = FALSE)
  f.noj.coast <- scale(pd.all.nona.coast$no.j, scale = FALSE)
  f.noj.midel <- scale(pd.all.nona.midel$no.j, scale = FALSE)
  f.noj.highl <- scale(pd.all.nona.highl$no.j, scale = FALSE)

  #Calculate Friedman's H-Stat to get the proportion of the effect due to interaction wi
th the other variables
  h.all <- sqrt(sum((f.all - (f.j + f.no.j))^2) / sum(f.all^2))
  h.coast <- sqrt(sum((f.coast - (f.j.coast + f.noj.coast))^2) / sum(f.coast^2))
  h.midel <- sqrt(sum((f.midel - (f.j.midel + f.noj.midel))^2) / sum(f.midel^2))
  h.highl <- sqrt(sum((f.highl - (f.j.highl + f.noj.highl))^2) / sum(f.highl^2))

  #Assign the Friedman's H-Stat to a dataframe
  C.Result.df <- data.frame(var = "Temp Mean", H.Stat = h.all, H.Stat.Coast = h.coast,
 H.Stat.Mid = h.midel, H.Stat.High = h.highl)

  #Model Effect Size of the Chosen Variable (Amount of the per mil variance explained by
the chosen variable)
  sqrt(sum((f.j)^2))
  sqrt(sum((f.j.coast)^2))
  sqrt(sum((f.j.midel)^2))
  sqrt(sum((f.j.highl)^2))

  #Add the effect size to the dataframe
  meant.EF <- data.frame(EF = sqrt(sum((f.j)^2)), EF.coast = sqrt(sum((f.j.coast)^2)), E
F.midel = sqrt(sum((f.j.midel)^2)), EF.high = sqrt(sum((f.j.highl)^2)))
  C.Result.df <- cbind(C.Result.df, meant.EF)

  #Percent Effect of the Chosen Variable (Percent of the variance in Carbon explained by
the chosen variable)
  sqrt(sum(f.j^2) / sum(f.all^2))
  sqrt(sum(f.j.coast^2) / sum(f.coast^2))
  sqrt(sum(f.j.midel^2) / sum(f.midel^2))
  sqrt(sum(f.j.highl^2) / sum(f.highl^2))

  #Assign the percent effect size to the dataframe
  meant.EF.per <- data.frame(EF.per = sqrt(sum(f.j^2) / sum(f.all^2)), EF.per.coast = sq
rt(sum(f.j.coast^2) / sum(f.coast^2)), EF.per.midel = sqrt(sum(f.j.midel^2) / sum(f.mide
l^2)), EF.per.high = sqrt(sum(f.j.highl^2) / sum(f.highl^2)))
  C.Result.df <- cbind(C.Result.df, meant.EF.per)

  #Now use the Friedman's H-Stat to control the effect size and percent to get the amoun
t of the variance explained by the chosen variable without its interaction with other va
riables
```

```
  #Effect Size Controlled
  sqrt(sum((f.j*(1-h.all))^2))
  sqrt(sum((f.j.coast*(1-h.coast))^2))
  sqrt(sum((f.j.midel*(1-h.midel))^2))
  sqrt(sum((f.j.highl*(1-h.highl))^2))

  #Assign the controlled effect size to a dataframe
  meant.EF.cont <- data.frame(EF.cont = sqrt(sum((f.j*(1-h.all))^2)), EF.cont.coast = sq
rt(sum((f.j.coast*(1-h.coast))^2)), EF.cont.midel = sqrt(sum((f.j.midel*(1-h.midel))^2
)), EF.cont.highl = sqrt(sum((f.j.highl*(1-h.highl))^2)))
  C.Result.df <- cbind(C.Result.df, meant.EF.cont)

  #Percent Effect of the Chosen Variable Controlled with Friedman's H-Stat
  #Effect Size Percent Controlled
  sqrt(sum((f.j*(1-h.all))^2) / sum(f.all^2))
  sqrt(sum((f.j.coast*(1-h.coast))^2) / sum(f.coast^2))
  sqrt(sum((f.j.midel*(1-h.midel))^2) / sum(f.midel^2))
  sqrt(sum((f.j.highl*(1-h.highl))^2) / sum(f.highl^2))

  #Assign the controlled percent effect size to a dataframe
  meant.EF.per.cont <- data.frame(EF.per.cont = sqrt(sum((f.j*(1-h.all))^2) / sum(f.all^
2)), EF.per.cont.coast = sqrt(sum((f.j.coast*(1-h.coast))^2) / sum(f.coast^2)), EF.per.c
ont.midel = sqrt(sum((f.j.midel*(1-h.midel))^2) / sum(f.midel^2)), EF.per.cont.highl = s
qrt(sum((f.j.highl*(1-h.highl))^2) / sum(f.highl^2)))
  C.Result.df <- cbind(C.Result.df, meant.EF.per.cont)


  if (k == 1) {
    Var.C.Result.df <- C.Result.df
  }
  else {
  Var.C.Result.df <- rbind(Var.C.Result.df, C.Result.df)
  }
}

#Average the 21 columns and assign the values to a new dataframe. This gives us the aver
age effect sizes based on 100 iterations of randomly sampling 100 values for the chosen
 variable, assigning those values to each individual, and predicting their C value.
Total.C.Result.df <- data.frame(row.names = "Temp Mean", t(colMeans(Var.C.Result.df[2:21
])))
```

Eighth, repeat for each of the other predictor variables.

Repeat for Mean Precip

```
#Choose the variable of interest. This will need to be repeated for each of our predicto
r variables (Temp_Mean, Temp_Mean_Seasonality, Precip_Mean, Precip_Mean_Seasonality, and
Demography.spd)
feature.name <- c("Precip_Mean")
```

Prediction function

```
predictor <- ia$predictor
```

Now get all the interactions for the chosen variable, calculate the Friedman’s H-stat (proportion of the effect due to the variable’s interaction with the other variables), calulate the effect size as the model sum of squares, discount the effect size based on the Friedman’s H-stat, and calculate the effect size a a percent of the variation explained.

```
#iterate the data sampling for interactions 100 times.
runs <- seq(1, 100, 1)

#Make an object for the sampling output to go into
myia <- list(res = data.table(), qr = data.frame())

#Make a datafame object for the results of each iteration
C.Result.df <- data.frame()

#Make an empty dataframe for the total results to append to
Var.C.Result.df <- data.frame()


for (k in runs) {

  myia <- interaction.run.single(
  dataSample = data.sample,
  feature.name = feature.name[1],
  grid.size = grid.size,
  batch.size = batch.size,
  q = myq,
  predictor = predictor
  )


  partial_dat <- myia$res#make a dataframe with the data generated for the interactions
  partial_dat$.y.hat <- myia$qr#add the predicted values to the dataframe
  partial_dat$.class <- 1#specify 1 so when we do the prediction R knows this is a regre
ssion problem

  #collapse into 'F(x)' values - this will provide the average of all of the predictions
for each unique value of the chosen variable. So average of all predictions per observat
ion on grid and per value of the feature.
  pd.all <- data.table::dcast(partial_dat, .feature + .id + elev.factor + .class ~ .typ
e,
                        value.var = ".y.hat", fun.aggregate = mean
)

  pd.all.nona <- pd.all[which(!is.na(pd.all$f)),]#drop NA rows, leaving the 100 aggregat
ed predictions
  pd.all.nona.coast <- pd.all.nona[which(pd.all.nona$elev.factor == "Coastal"),]#Grab on
ly coastal cases.
  pd.all.nona.midel <- pd.all.nona[which(pd.all.nona$elev.factor == "Mid-elevation"),]#G
rab only mid-elevation cases.
  pd.all.nona.highl <- pd.all.nona[which(pd.all.nona$elev.factor == "Highland"),]#Grab o
nly highland cases.

  #Calculate Total Response of Carbon
  f.all <- scale(pd.all.nona$f, scale = FALSE)
  f.coast <- scale(pd.all.nona.coast$f, scale = FALSE)
  f.midel <- scale(pd.all.nona.midel$f, scale = FALSE)
  f.highl <- scale(pd.all.nona.highl$f, scale = FALSE)
```

```
  #Calculate Partial Dependence of Carbon for Chosen Variable
  f.j <- scale(pd.all.nona$j, scale = FALSE)
  f.j.coast <- scale(pd.all.nona.coast$j, scale = FALSE)
  f.j.midel <- scale(pd.all.nona.midel$j, scale = FALSE)
  f.j.highl <- scale(pd.all.nona.highl$j, scale = FALSE)

  #Calculate Combined Partial Dependence of Carbon to all Other Variables
  f.no.j <- scale(pd.all.nona$no.j, scale = FALSE)
  f.noj.coast <- scale(pd.all.nona.coast$no.j, scale = FALSE)
  f.noj.midel <- scale(pd.all.nona.midel$no.j, scale = FALSE)
  f.noj.highl <- scale(pd.all.nona.highl$no.j, scale = FALSE)

  #Calculate Friedman's H-Stat to get the proportion of the effect due to interaction wi
th the other variables
  h.all <- sqrt(sum((f.all - (f.j + f.no.j))^2) / sum(f.all^2))
  h.coast <- sqrt(sum((f.coast - (f.j.coast + f.noj.coast))^2) / sum(f.coast^2))
  h.midel <- sqrt(sum((f.midel - (f.j.midel + f.noj.midel))^2) / sum(f.midel^2))
  h.highl <- sqrt(sum((f.highl - (f.j.highl + f.noj.highl))^2) / sum(f.highl^2))

  #Assign the Friedman's H-Stat to a dataframe
  C.Result.df <- data.frame(var = "Precip Mean", H.Stat = h.all, H.Stat.Coast = h.coast,
H.Stat.Mid = h.midel, H.Stat.High = h.highl)

  #Model Effect Size of the Chosen Variable (Amount of the per mil variance explained by
the chosen variable)
  sqrt(sum((f.j)^2))
  sqrt(sum((f.j.coast)^2))
  sqrt(sum((f.j.midel)^2))
  sqrt(sum((f.j.highl)^2))

  #Add the effect size to the dataframe
  meant.EF <- data.frame(EF = sqrt(sum((f.j)^2)), EF.coast = sqrt(sum((f.j.coast)^2)), E
F.midel = sqrt(sum((f.j.midel)^2)), EF.high = sqrt(sum((f.j.highl)^2)))
  C.Result.df <- cbind(C.Result.df, meant.EF)

  #Percent Effect of the Chosen Variable (Percent of the variance in Carbon explained by
the chosen variable)
  sqrt(sum(f.j^2) / sum(f.all^2))
  sqrt(sum(f.j.coast^2) / sum(f.coast^2))
  sqrt(sum(f.j.midel^2) / sum(f.midel^2))
  sqrt(sum(f.j.highl^2) / sum(f.highl^2))

  #Assign the percent effect size to the dataframe
  meant.EF.per <- data.frame(EF.per = sqrt(sum(f.j^2) / sum(f.all^2)), EF.per.coast = sq
rt(sum(f.j.coast^2) / sum(f.coast^2)), EF.per.midel = sqrt(sum(f.j.midel^2) / sum(f.mide
l^2)), EF.per.high = sqrt(sum(f.j.highl^2) / sum(f.highl^2)))
  C.Result.df <- cbind(C.Result.df, meant.EF.per)

  #Now use the Friedman's H-Stat to control the effect size and percent to get the amoun
t of the variance explained by the chosen variable without its interaction with other va
riables
```

```
  #Effect Size Controlled
  sqrt(sum((f.j*(1-h.all))^2))
  sqrt(sum((f.j.coast*(1-h.coast))^2))
  sqrt(sum((f.j.midel*(1-h.midel))^2))
  sqrt(sum((f.j.highl*(1-h.highl))^2))

  #Assign the controlled effect size to a dataframe
  meant.EF.cont <- data.frame(EF.cont = sqrt(sum((f.j*(1-h.all))^2)), EF.cont.coast = sq
rt(sum((f.j.coast*(1-h.coast))^2)), EF.cont.midel = sqrt(sum((f.j.midel*(1-h.midel))^2
)), EF.cont.highl = sqrt(sum((f.j.highl*(1-h.highl))^2)))
  C.Result.df <- cbind(C.Result.df, meant.EF.cont)

  #Percent Effect of the Chosen Variable Controlled with Friedman's H-Stat
  #Effect Size Percent Controlled
  sqrt(sum((f.j*(1-h.all))^2) / sum(f.all^2))
  sqrt(sum((f.j.coast*(1-h.coast))^2) / sum(f.coast^2))
  sqrt(sum((f.j.midel*(1-h.midel))^2) / sum(f.midel^2))
  sqrt(sum((f.j.highl*(1-h.highl))^2) / sum(f.highl^2))

  #Assign the controlled percent effect size to a dataframe
  meant.EF.per.cont <- data.frame(EF.per.cont = sqrt(sum((f.j*(1-h.all))^2) / sum(f.all^
2)), EF.per.cont.coast = sqrt(sum((f.j.coast*(1-h.coast))^2) / sum(f.coast^2)), EF.per.c
ont.midel = sqrt(sum((f.j.midel*(1-h.midel))^2) / sum(f.midel^2)), EF.per.cont.highl = s
qrt(sum((f.j.highl*(1-h.highl))^2) / sum(f.highl^2)))
  C.Result.df <- cbind(C.Result.df, meant.EF.per.cont)


  if (k == 1) {
    Var.C.Result.df <- C.Result.df
  }
  else {
  Var.C.Result.df <- rbind(Var.C.Result.df, C.Result.df)
  }
}

#Grab the column averages (this will be the 100 runs of 100 different var values average
d together)
Var.avg <- data.frame(row.names = "Precip Mean", t(colMeans(Var.C.Result.df[2:21])))

#Add the new variable values to the Total dataframe
Total.C.Result.df <- rbind(Total.C.Result.df, Var.avg)
```

Repeat for Precip Seasonality

```
#Choose the variable of interest. This will need to be repeated for each of our predicto
r variables (Temp_Mean, Temp_Mean_Seasonality, Precip_Mean, Precip_Mean_Seasonality, and
Demography.spd)
feature.name <- c("Precip_Mean_Seasonality")
```

Prediction function

```
predictor <- ia$predictor
```

Now get all the interactions for the chosen variable, calculate the Friedman's H-stat (proportion of the effect due to the variable's interaction with the other variables), calulate the effect size as the model sum of squares, discount the effect size based on the Friedman's H-stat, and calculate the effect size a a percent of the variation explained.

```
#iterate the data sampling for interactions 100 times.
runs <- seq(1, 100, 1)

#Make an object for the sampling output to go into
myia <- list(res = data.table(), qr = data.frame())

#Make a datafame object for the results of each iteration
C.Result.df <- data.frame()

#Make an empty dataframe for the total results to append to
Var.C.Result.df <- data.frame()


for (k in runs) {

  myia <- interaction.run.single(
  dataSample = data.sample,
  feature.name = feature.name[1],
  grid.size = grid.size,
  batch.size = batch.size,
  q = myq,
  predictor = predictor
  )


  partial_dat <- myia$res#make a dataframe with the data generated for the interactions
  partial_dat$.y.hat <- myia$qr#add the predicted values to the dataframe
  partial_dat$.class <- 1#specify 1 so when we do the prediction R knows this is a regre
ssion problem

  #collapse into 'F(x)' values - this will provide the average of all of the predictions
for each unique value of the chosen variable. So average of all predictions per observat
ion on grid and per value of the feature.
  pd.all <- data.table::dcast(partial_dat, .feature + .id + elev.factor + .class ~ .typ
e,
                        value.var = ".y.hat", fun.aggregate = mean
)

  pd.all.nona <- pd.all[which(!is.na(pd.all$f)),]#drop NA rows, leaving the 100 aggregat
ed predictions
  pd.all.nona.coast <- pd.all.nona[which(pd.all.nona$elev.factor == "Coastal"),]#Grab on
ly coastal cases.
  pd.all.nona.midel <- pd.all.nona[which(pd.all.nona$elev.factor == "Mid-elevation"),]#G
rab only mid-elevation cases.
  pd.all.nona.highl <- pd.all.nona[which(pd.all.nona$elev.factor == "Highland"),]#Grab o
nly highland cases.

  #Calculate Total Response of Carbon
  f.all <- scale(pd.all.nona$f, scale = FALSE)
  f.coast <- scale(pd.all.nona.coast$f, scale = FALSE)
  f.midel <- scale(pd.all.nona.midel$f, scale = FALSE)
  f.highl <- scale(pd.all.nona.highl$f, scale = FALSE)
```

```
  #Calculate Partial Dependence of Carbon for Chosen Variable
  f.j <- scale(pd.all.nona$j, scale = FALSE)
  f.j.coast <- scale(pd.all.nona.coast$j, scale = FALSE)
  f.j.midel <- scale(pd.all.nona.midel$j, scale = FALSE)
  f.j.highl <- scale(pd.all.nona.highl$j, scale = FALSE)

  #Calculate Combined Partial Dependence of Carbon to all Other Variables
  f.no.j <- scale(pd.all.nona$no.j, scale = FALSE)
  f.noj.coast <- scale(pd.all.nona.coast$no.j, scale = FALSE)
  f.noj.midel <- scale(pd.all.nona.midel$no.j, scale = FALSE)
  f.noj.highl <- scale(pd.all.nona.highl$no.j, scale = FALSE)

  #Calculate Friedman's H-Stat to get the proportion of the effect due to interaction wi
th the other variables
  h.all <- sqrt(sum((f.all - (f.j + f.no.j))^2) / sum(f.all^2))
  h.coast <- sqrt(sum((f.coast - (f.j.coast + f.noj.coast))^2) / sum(f.coast^2))
  h.midel <- sqrt(sum((f.midel - (f.j.midel + f.noj.midel))^2) / sum(f.midel^2))
  h.highl <- sqrt(sum((f.highl - (f.j.highl + f.noj.highl))^2) / sum(f.highl^2))

  #Assign the Friedman's H-Stat to a dataframe
  C.Result.df <- data.frame(var = "Precip Mean Seasonality", H.Stat = h.all, H.Stat.Coas
t = h.coast, H.Stat.Mid = h.midel, H.Stat.High = h.highl)

  #Model Effect Size of the Chosen Variable (Amount of the per mil variance explained by
the chosen variable)
  sqrt(sum((f.j)^2))
  sqrt(sum((f.j.coast)^2))
  sqrt(sum((f.j.midel)^2))
  sqrt(sum((f.j.highl)^2))

  #Add the effect size to the dataframe
  meant.EF <- data.frame(EF = sqrt(sum((f.j)^2)), EF.coast = sqrt(sum((f.j.coast)^2)), E
F.midel = sqrt(sum((f.j.midel)^2)), EF.high = sqrt(sum((f.j.highl)^2)))
  C.Result.df <- cbind(C.Result.df, meant.EF)

  #Percent Effect of the Chosen Variable (Percent of the variance in Carbon explained by
the chosen variable)
  sqrt(sum(f.j^2) / sum(f.all^2))
  sqrt(sum(f.j.coast^2) / sum(f.coast^2))
  sqrt(sum(f.j.midel^2) / sum(f.midel^2))
  sqrt(sum(f.j.highl^2) / sum(f.highl^2))

  #Assign the percent effect size to the dataframe
  meant.EF.per <- data.frame(EF.per = sqrt(sum(f.j^2) / sum(f.all^2)), EF.per.coast = sq
rt(sum(f.j.coast^2) / sum(f.coast^2)), EF.per.midel = sqrt(sum(f.j.midel^2) / sum(f.mide
l^2)), EF.per.high = sqrt(sum(f.j.highl^2) / sum(f.highl^2)))
  C.Result.df <- cbind(C.Result.df, meant.EF.per)

  #Now use the Friedman's H-Stat to control the effect size and percent to get the amoun
t of the variance explained by the chosen variable without its interaction with other va
riables
```

```
  #Effect Size Controlled
  sqrt(sum((f.j*(1-h.all))^2))
  sqrt(sum((f.j.coast*(1-h.coast))^2))
  sqrt(sum((f.j.midel*(1-h.midel))^2))
  sqrt(sum((f.j.highl*(1-h.highl))^2))

  #Assign the controlled effect size to a dataframe
  meant.EF.cont <- data.frame(EF.cont = sqrt(sum((f.j*(1-h.all))^2)), EF.cont.coast = sq
rt(sum((f.j.coast*(1-h.coast))^2)), EF.cont.midel = sqrt(sum((f.j.midel*(1-h.midel))^2
)), EF.cont.highl = sqrt(sum((f.j.highl*(1-h.highl))^2)))
  C.Result.df <- cbind(C.Result.df, meant.EF.cont)

  #Percent Effect of the Chosen Variable Controlled with Friedman's H-Stat
  #Effect Size Percent Controlled
  sqrt(sum((f.j*(1-h.all))^2) / sum(f.all^2))
  sqrt(sum((f.j.coast*(1-h.coast))^2) / sum(f.coast^2))
  sqrt(sum((f.j.midel*(1-h.midel))^2) / sum(f.midel^2))
  sqrt(sum((f.j.highl*(1-h.highl))^2) / sum(f.highl^2))

  #Assign the controlled percent effect size to a dataframe
  meant.EF.per.cont <- data.frame(EF.per.cont = sqrt(sum((f.j*(1-h.all))^2) / sum(f.all^
2)), EF.per.cont.coast = sqrt(sum((f.j.coast*(1-h.coast))^2) / sum(f.coast^2)), EF.per.c
ont.midel = sqrt(sum((f.j.midel*(1-h.midel))^2) / sum(f.midel^2)), EF.per.cont.highl = s
qrt(sum((f.j.highl*(1-h.highl))^2) / sum(f.highl^2)))
  C.Result.df <- cbind(C.Result.df, meant.EF.per.cont)


  if (k == 1) {
    Var.C.Result.df <- C.Result.df
  }
  else {
  Var.C.Result.df <- rbind(Var.C.Result.df, C.Result.df)
  }
}

#Grab the column averages (this will be the 100 runs of 100 different var values average
d together)
Var.avg <- data.frame(row.names = "Precip Mean Seasonality", t(colMeans(Var.C.Result.df[
2:21])))

#Add the new variable values to the Total dataframe
Total.C.Result.df <- rbind(Total.C.Result.df, Var.avg)
```

Repeat for Temp Seasonality

```
#Choose the variable of interest. This will need to be repeated for each of our predicto
r variables (Temp_Mean, Temp_Mean_Seasonality, Precip_Mean, Precip_Mean_Seasonality, and
Demography.spd)
feature.name <- c("Temp_Mean_Seasonality")
```

Prediction function

```
predictor <- ia$predictor
```

Now get all the interactions for the chosen variable, calculate the Friedman's H-stat (proportion of the effect due to the variable's interaction with the other variables), calulate the effect size as the model sum of squares, discount the effect size based on the Friedman's H-stat, and calculate the effect size a a percent of the variation explained.

```
#iterate the data sampling for interactions 100 times.
runs <- seq(1, 100, 1)

#Make an object for the sampling output to go into
myia <- list(res = data.table(), qr = data.frame())

#Make a datafame object for the results of each iteration
C.Result.df <- data.frame()

#Make an empty dataframe for the total results to append to
Var.C.Result.df <- data.frame()


for (k in runs) {

  myia <- interaction.run.single(
  dataSample = data.sample,
  feature.name = feature.name[1],
  grid.size = grid.size,
  batch.size = batch.size,
  q = myq,
  predictor = predictor
  )


  partial_dat <- myia$res#make a dataframe with the data generated for the interactions
  partial_dat$.y.hat <- myia$qr#add the predicted values to the dataframe
  partial_dat$.class <- 1#specify 1 so when we do the prediction R knows this is a regre
ssion problem

  #collapse into 'F(x)' values - this will provide the average of all of the predictions
for each unique value of the chosen variable. So average of all predictions per observat
ion on grid and per value of the feature.
  pd.all <- data.table::dcast(partial_dat, .feature + .id + elev.factor + .class ~ .typ
e,
                        value.var = ".y.hat", fun.aggregate = mean
)

  pd.all.nona <- pd.all[which(!is.na(pd.all$f)),]#drop NA rows, leaving the 100 aggregat
ed predictions
  pd.all.nona.coast <- pd.all.nona[which(pd.all.nona$elev.factor == "Coastal"),]#Grab on
ly coastal cases.
  pd.all.nona.midel <- pd.all.nona[which(pd.all.nona$elev.factor == "Mid-elevation"),]#G
rab only mid-elevation cases.
  pd.all.nona.highl <- pd.all.nona[which(pd.all.nona$elev.factor == "Highland"),]#Grab o
nly highland cases.

  #Calculate Total Response of Carbon
  f.all <- scale(pd.all.nona$f, scale = FALSE)
  f.coast <- scale(pd.all.nona.coast$f, scale = FALSE)
  f.midel <- scale(pd.all.nona.midel$f, scale = FALSE)
  f.highl <- scale(pd.all.nona.highl$f, scale = FALSE)
```

```
  #Calculate Partial Dependence of Carbon for Chosen Variable
  f.j <- scale(pd.all.nona$j, scale = FALSE)
  f.j.coast <- scale(pd.all.nona.coast$j, scale = FALSE)
  f.j.midel <- scale(pd.all.nona.midel$j, scale = FALSE)
  f.j.highl <- scale(pd.all.nona.highl$j, scale = FALSE)

  #Calculate Combined Partial Dependence of Carbon to all Other Variables
  f.no.j <- scale(pd.all.nona$no.j, scale = FALSE)
  f.noj.coast <- scale(pd.all.nona.coast$no.j, scale = FALSE)
  f.noj.midel <- scale(pd.all.nona.midel$no.j, scale = FALSE)
  f.noj.highl <- scale(pd.all.nona.highl$no.j, scale = FALSE)

  #Calculate Friedman's H-Stat to get the proportion of the effect due to interaction wi
th the other variables
  h.all <- sqrt(sum((f.all - (f.j + f.no.j))^2) / sum(f.all^2))
  h.coast <- sqrt(sum((f.coast - (f.j.coast + f.noj.coast))^2) / sum(f.coast^2))
  h.midel <- sqrt(sum((f.midel - (f.j.midel + f.noj.midel))^2) / sum(f.midel^2))
  h.highl <- sqrt(sum((f.highl - (f.j.highl + f.noj.highl))^2) / sum(f.highl^2))

  #Assign the Friedman's H-Stat to a dataframe
  C.Result.df <- data.frame(var = "Temp Mean Seasonality", H.Stat = h.all, H.Stat.Coast
 = h.coast, H.Stat.Mid = h.midel, H.Stat.High = h.highl)

  #Model Effect Size of the Chosen Variable (Amount of the per mil variance explained by
the chosen variable)
  sqrt(sum((f.j)^2))
  sqrt(sum((f.j.coast)^2))
  sqrt(sum((f.j.midel)^2))
  sqrt(sum((f.j.highl)^2))

  #Add the effect size to the dataframe
  meant.EF <- data.frame(EF = sqrt(sum((f.j)^2)), EF.coast = sqrt(sum((f.j.coast)^2)), E
F.midel = sqrt(sum((f.j.midel)^2)), EF.high = sqrt(sum((f.j.highl)^2)))
  C.Result.df <- cbind(C.Result.df, meant.EF)

  #Percent Effect of the Chosen Variable (Percent of the variance in Carbon explained by
the chosen variable)
  sqrt(sum(f.j^2) / sum(f.all^2))
  sqrt(sum(f.j.coast^2) / sum(f.coast^2))
  sqrt(sum(f.j.midel^2) / sum(f.midel^2))
  sqrt(sum(f.j.highl^2) / sum(f.highl^2))

  #Assign the percent effect size to the dataframe
  meant.EF.per <- data.frame(EF.per = sqrt(sum(f.j^2) / sum(f.all^2)), EF.per.coast = sq
rt(sum(f.j.coast^2) / sum(f.coast^2)), EF.per.midel = sqrt(sum(f.j.midel^2) / sum(f.mide
l^2)), EF.per.high = sqrt(sum(f.j.highl^2) / sum(f.highl^2)))
  C.Result.df <- cbind(C.Result.df, meant.EF.per)

  #Now use the Friedman's H-Stat to control the effect size and percent to get the amoun
t of the variance explained by the chosen variable without its interaction with other va
riables
```

```
  #Effect Size Controlled
  sqrt(sum((f.j*(1-h.all))^2))
  sqrt(sum((f.j.coast*(1-h.coast))^2))
  sqrt(sum((f.j.midel*(1-h.midel))^2))
  sqrt(sum((f.j.highl*(1-h.highl))^2))

  #Assign the controlled effect size to a dataframe
  meant.EF.cont <- data.frame(EF.cont = sqrt(sum((f.j*(1-h.all))^2)), EF.cont.coast = sq
rt(sum((f.j.coast*(1-h.coast))^2)), EF.cont.midel = sqrt(sum((f.j.midel*(1-h.midel))^2
)), EF.cont.highl = sqrt(sum((f.j.highl*(1-h.highl))^2)))
  C.Result.df <- cbind(C.Result.df, meant.EF.cont)

  #Percent Effect of the Chosen Variable Controlled with Friedman's H-Stat
  #Effect Size Percent Controlled
  sqrt(sum((f.j*(1-h.all))^2) / sum(f.all^2))
  sqrt(sum((f.j.coast*(1-h.coast))^2) / sum(f.coast^2))
  sqrt(sum((f.j.midel*(1-h.midel))^2) / sum(f.midel^2))
  sqrt(sum((f.j.highl*(1-h.highl))^2) / sum(f.highl^2))

  #Assign the controlled percent effect size to a dataframe
  meant.EF.per.cont <- data.frame(EF.per.cont = sqrt(sum((f.j*(1-h.all))^2) / sum(f.all^
2)), EF.per.cont.coast = sqrt(sum((f.j.coast*(1-h.coast))^2) / sum(f.coast^2)), EF.per.c
ont.midel = sqrt(sum((f.j.midel*(1-h.midel))^2) / sum(f.midel^2)), EF.per.cont.highl = s
qrt(sum((f.j.highl*(1-h.highl))^2) / sum(f.highl^2)))
  C.Result.df <- cbind(C.Result.df, meant.EF.per.cont)


  if (k == 1) {
    Var.C.Result.df <- C.Result.df
  }
  else {
  Var.C.Result.df <- rbind(Var.C.Result.df, C.Result.df)
  }
}

#Grab the column averages (this will be the 100 runs of 100 different var values average
d together)
Var.avg <- data.frame(row.names = "Temp Mean Seasonality", t(colMeans(Var.C.Result.df[2:
21])))

#Add the new variable values to the Total dataframe
Total.C.Result.df <- rbind(Total.C.Result.df, Var.avg)

#Generate the Cumulative Climate Effect
Cum.Clim. <- data.frame(row.names = "Cumulative Climate", t(colSums(Total.C.Result.df)))

#Add Cumulative Climate to the Total Effect Size Dataframe
Total.C.Result.df <- rbind(Total.C.Result.df, Cum.Clim.)

#Pull Out the Key Effect Sizes to store in their own dataframe
Cumulative.EF.C <- data.frame(Elev.cat = c("Coastal", "Mid-elevation", "Highland"), Cum.
Clim.EF = c(Total.C.Result.df[5,14], Total.C.Result.df[5,15], Total.C.Result.df[5,16]),
```

```
 Cum.Clim.EF.percent = c(Total.C.Result.df[5,18], Total.C.Result.df[5,19], Total.C.Resul
t.df[5,20]))

Cumulative.EF
```

```
##        Elev.cat Cum.Clim.EF Cum.Clim.EF.percent    Dem.EF Dem.EF.percent
## 1       Coastal   19.377893           0.7623692 1.6416293     0.06868033
## 2 Mid-elevation    8.955762           0.5120138 3.5941650     0.22688625
## 3      Highland    2.077550           0.4412140 0.5714959     0.09175686
```

```
Cumulative.EF.C
```

```
##        Elev.cat Cum.Clim.EF Cum.Clim.EF.percent
## 1       Coastal    4.099650           0.5812959
## 2 Mid-elevation    8.887869           0.5446406
## 3      Highland    4.885757           0.8070801
```

Repeat for Demography

```
#Choose the variable of interest. This will need to be repeated for each of our predicto
r variables (Temp_Mean, Temp_Mean_Seasonality, Precip_Mean, Precip_Mean_Seasonality, and
Demography.spd)
feature.name <- c("Demography.kde.14c")
```

Prediction function

```
predictor <- ia$predictor
```

Now get all the interactions for the chosen variable, calculate the Friedman's H-stat (proportion of the effect due to the variable's interaction with the other variables), calulate the effect size as the model sum of squares, discount the effect size based on the Friedman's H-stat, and calculate the effect size a a percent of the variation explained.

```
#iterate the data sampling for interactions 100 times.
runs <- seq(1, 100, 1)

#Make an object for the sampling output to go into
myia <- list(res = data.table(), qr = data.frame())

#Make a datafame object for the results of each iteration
C.Result.df <- data.frame()

#Make an empty dataframe for the total results to append to
Var.C.Result.df <- data.frame()


for (k in runs) {

  myia <- interaction.run.single(
  dataSample = data.sample,
  feature.name = feature.name[1],
  grid.size = grid.size,
  batch.size = batch.size,
  q = myq,
  predictor = predictor
  )


  partial_dat <- myia$res#make a dataframe with the data generated for the interactions
  partial_dat$.y.hat <- myia$qr#add the predicted values to the dataframe
  partial_dat$.class <- 1#specify 1 so when we do the prediction R knows this is a regre
ssion problem

  #collapse into 'F(x)' values - this will provide the average of all of the predictions
for each unique value of the chosen variable. So average of all predictions per observat
ion on grid and per value of the feature.
  pd.all <- data.table::dcast(partial_dat, .feature + .id + elev.factor + .class ~ .typ
e,
                        value.var = ".y.hat", fun.aggregate = mean
)

  pd.all.nona <- pd.all[which(!is.na(pd.all$f)),]#drop NA rows, leaving the 100 aggregat
ed predictions
  pd.all.nona.coast <- pd.all.nona[which(pd.all.nona$elev.factor == "Coastal"),]#Grab on
ly coastal cases.
  pd.all.nona.midel <- pd.all.nona[which(pd.all.nona$elev.factor == "Mid-elevation"),]#G
rab only mid-elevation cases.
  pd.all.nona.highl <- pd.all.nona[which(pd.all.nona$elev.factor == "Highland"),]#Grab o
nly highland cases.

  #Calculate Total Response of Carbon
  f.all <- scale(pd.all.nona$f, scale = FALSE)
  f.coast <- scale(pd.all.nona.coast$f, scale = FALSE)
  f.midel <- scale(pd.all.nona.midel$f, scale = FALSE)
  f.highl <- scale(pd.all.nona.highl$f, scale = FALSE)
```

```
  #Calculate Partial Dependence of Carbon for Chosen Variable
  f.j <- scale(pd.all.nona$j, scale = FALSE)
  f.j.coast <- scale(pd.all.nona.coast$j, scale = FALSE)
  f.j.midel <- scale(pd.all.nona.midel$j, scale = FALSE)
  f.j.highl <- scale(pd.all.nona.highl$j, scale = FALSE)

  #Calculate Combined Partial Dependence of Carbon to all Other Variables
  f.no.j <- scale(pd.all.nona$no.j, scale = FALSE)
  f.noj.coast <- scale(pd.all.nona.coast$no.j, scale = FALSE)
  f.noj.midel <- scale(pd.all.nona.midel$no.j, scale = FALSE)
  f.noj.highl <- scale(pd.all.nona.highl$no.j, scale = FALSE)

  #Calculate Friedman's H-Stat to get the proportion of the effect due to interaction wi
th the other variables
  h.all <- sqrt(sum((f.all - (f.j + f.no.j))^2) / sum(f.all^2))
  h.coast <- sqrt(sum((f.coast - (f.j.coast + f.noj.coast))^2) / sum(f.coast^2))
  h.midel <- sqrt(sum((f.midel - (f.j.midel + f.noj.midel))^2) / sum(f.midel^2))
  h.highl <- sqrt(sum((f.highl - (f.j.highl + f.noj.highl))^2) / sum(f.highl^2))

  #Assign the Friedman's H-Stat to a dataframe
  C.Result.df <- data.frame(var = "Demography", H.Stat = h.all, H.Stat.Coast = h.coast,
 H.Stat.Mid = h.midel, H.Stat.High = h.highl)

  #Model Effect Size of the Chosen Variable (Amount of the per mil variance explained by
the chosen variable)
  sqrt(sum((f.j)^2))
  sqrt(sum((f.j.coast)^2))
  sqrt(sum((f.j.midel)^2))
  sqrt(sum((f.j.highl)^2))

  #Add the effect size to the dataframe
  meant.EF <- data.frame(EF = sqrt(sum((f.j)^2)), EF.coast = sqrt(sum((f.j.coast)^2)), E
F.midel = sqrt(sum((f.j.midel)^2)), EF.high = sqrt(sum((f.j.highl)^2)))
  C.Result.df <- cbind(C.Result.df, meant.EF)

  #Percent Effect of the Chosen Variable (Percent of the variance in Carbon explained by
the chosen variable)
  sqrt(sum(f.j^2) / sum(f.all^2))
  sqrt(sum(f.j.coast^2) / sum(f.coast^2))
  sqrt(sum(f.j.midel^2) / sum(f.midel^2))
  sqrt(sum(f.j.highl^2) / sum(f.highl^2))

  #Assign the percent effect size to the dataframe
  meant.EF.per <- data.frame(EF.per = sqrt(sum(f.j^2) / sum(f.all^2)), EF.per.coast = sq
rt(sum(f.j.coast^2) / sum(f.coast^2)), EF.per.midel = sqrt(sum(f.j.midel^2) / sum(f.mide
l^2)), EF.per.high = sqrt(sum(f.j.highl^2) / sum(f.highl^2)))
  C.Result.df <- cbind(C.Result.df, meant.EF.per)

  #Now use the Friedman's H-Stat to control the effect size and percent to get the amoun
t of the variance explained by the chosen variable without its interaction with other va
riables
```

```
  #Effect Size Controlled
  sqrt(sum((f.j*(1-h.all))^2))
  sqrt(sum((f.j.coast*(1-h.coast))^2))
  sqrt(sum((f.j.midel*(1-h.midel))^2))
  sqrt(sum((f.j.highl*(1-h.highl))^2))

  #Assign the controlled effect size to a dataframe
  meant.EF.cont <- data.frame(EF.cont = sqrt(sum((f.j*(1-h.all))^2)), EF.cont.coast = sq
rt(sum((f.j.coast*(1-h.coast))^2)), EF.cont.midel = sqrt(sum((f.j.midel*(1-h.midel))^2
)), EF.cont.highl = sqrt(sum((f.j.highl*(1-h.highl))^2)))
  C.Result.df <- cbind(C.Result.df, meant.EF.cont)

  #Percent Effect of the Chosen Variable Controlled with Friedman's H-Stat
  #Effect Size Percent Controlled
  sqrt(sum((f.j*(1-h.all))^2) / sum(f.all^2))
  sqrt(sum((f.j.coast*(1-h.coast))^2) / sum(f.coast^2))
  sqrt(sum((f.j.midel*(1-h.midel))^2) / sum(f.midel^2))
  sqrt(sum((f.j.highl*(1-h.highl))^2) / sum(f.highl^2))

  #Assign the controlled percent effect size to a dataframe
  meant.EF.per.cont <- data.frame(EF.per.cont = sqrt(sum((f.j*(1-h.all))^2) / sum(f.all^
2)), EF.per.cont.coast = sqrt(sum((f.j.coast*(1-h.coast))^2) / sum(f.coast^2)), EF.per.c
ont.midel = sqrt(sum((f.j.midel*(1-h.midel))^2) / sum(f.midel^2)), EF.per.cont.highl = s
qrt(sum((f.j.highl*(1-h.highl))^2) / sum(f.highl^2)))
  C.Result.df <- cbind(C.Result.df, meant.EF.per.cont)


  if (k == 1) {
    Var.C.Result.df <- C.Result.df
  }
  else {
  Var.C.Result.df <- rbind(Var.C.Result.df, C.Result.df)
  }
}

#Grab the column averages (this will be the 100 runs of 100 different var values average
d together)
Var.avg <- data.frame(row.names = "Demography", t(colMeans(Var.C.Result.df[2:21])))

#Add the new variable values to the Total dataframe
Total.C.Result.df <- rbind(Total.C.Result.df, Var.avg)

#Add demography results to the cumulative effect size dataframe
Cumulative.EF.C$Dem.EF <- c(sqrt(sum((f.j.coast*(1-h.coast))^2)), sqrt(sum((f.j.midel*(1
-h.midel))^2)), sqrt(sum((f.j.highl*(1-h.highl))^2)))
Cumulative.EF.C$Dem.EF.percent <- c(sqrt(sum((f.j.coast*(1-h.coast))^2) / sum(f.coast^2
)), sqrt(sum((f.j.midel*(1-h.midel))^2) / sum(f.midel^2)), sqrt(sum((f.j.highl*(1-h.high
l))^2) / sum(f.highl^2)))
```

Show the complete set of effect size calculations.

```
#show the complete set of effect size calculations - for each variable, this is: H-stat, Effect Size, Effect Size percent, Effect size controlled by H-stat, Effect size percent controlled by H-stat
round(Total.C.Result.df, 2)
```

```
##                          H.Stat H.Stat.Coast H.Stat.Mid H.Stat.High    EF
## Temp Mean                  0.36         0.31       0.27        0.36 11.92
## Precip Mean                0.46         0.29       0.30        0.33 11.76
## Precip Mean Seasonality    0.45         0.33       0.27        0.21 12.86
## Temp Mean Seasonality      0.45         0.44       0.36        0.23 11.48
## Cumulative Climate         1.72         1.37       1.19        1.13 48.02
## Demography                 0.55         0.36       0.28        0.23 12.97
##                          EF.coast EF.midel EF.high EF.per EF.per.coast
## Temp Mean                    1.05     1.38    3.40   0.52         0.15
## Precip Mean                  2.08     2.56    1.63   0.51         0.30
## Precip Mean Seasonality      1.79     4.74    1.33   0.56         0.25
## Temp Mean Seasonality        1.28     4.03    0.76   0.49         0.19
## Cumulative Climate           6.20    12.72    7.11   2.08         0.88
## Demography                   1.46     3.76    0.82   0.56         0.21
##                          EF.per.midel EF.per.high EF.cont EF.cont.coast
## Temp Mean                        0.09        0.56    7.59          0.73
## Precip Mean                      0.16        0.28    6.38          1.47
## Precip Mean Seasonality          0.29        0.22    7.06          1.20
## Temp Mean Seasonality            0.25        0.13    6.28          0.71
## Cumulative Climate               0.78        1.18   27.30          4.10
## Demography                       0.23        0.14    5.86          0.93
##                          EF.cont.midel EF.cont.highl EF.per.cont
## Temp Mean                         1.02          2.17        0.33
## Precip Mean                       1.80          1.09        0.28
## Precip Mean Seasonality           3.46          1.06        0.31
## Temp Mean Seasonality             2.61          0.58        0.27
## Cumulative Climate                8.89          4.89        1.18
## Demography                        2.70          0.64        0.25
##                          EF.per.cont.coast EF.per.cont.midel EF.per.cont.highl
## Temp Mean                             0.10              0.06              0.35
## Precip Mean                           0.21              0.11              0.19
## Precip Mean Seasonality               0.17              0.21              0.17
## Temp Mean Seasonality                 0.10              0.16              0.10
## Cumulative Climate                    0.58              0.54              0.81
## Demography                            0.13              0.16              0.10
```

Table S2. Complete Effect Size Calculations for δ^{13}C‰. For each variable we report the complete model H-stat and effect size as well as the H-stat and effect sizes within each elevational zone.

Key Results

```
C.Results <- Total.C.Result.df[,c(14:16, 18:20)]
round(C.Results,4)
```

```
##                           EF.cont.coast EF.cont.midel EF.cont.highl
## Temp Mean                        0.7299        1.0168        2.1655
## Precip Mean                      1.4663        1.7988        1.0855
## Precip Mean Seasonality          1.1973        3.4630        1.0580
## Temp Mean Seasonality            0.7061        2.6092        0.5768
## Cumulative Climate               4.0997        8.8879        4.8858
## Demography                       0.9268        2.7021        0.6354
##                           EF.per.cont.coast EF.per.cont.midel EF.per.cont.highl
## Temp Mean                            0.1030            0.0638            0.3536
## Precip Mean                          0.2096            0.1101            0.1869
## Precip Mean Seasonality              0.1669            0.2123            0.1709
## Temp Mean Seasonality                0.1018            0.1585            0.0957
## Cumulative Climate                   0.5813            0.5446            0.8071
## Demography                           0.1334            0.1644            0.1044
```

Table 1 Part 2. Carbon Results.

Effect Size Results

Make Figure 4

```
#Rely on the Cumulative Results dataframes

n.bar.ef <- Cumulative.EF
c.bar.ef <- Cumulative.EF.C

n.bar.ef$Cum.Clim.EF <- n.bar.ef$Cum.Clim.EF*-1 #Change the sign to negative for the lef
t hand side of the barplot
n.bar.ef$Cum.Clim.EF.percent <- n.bar.ef$Cum.Clim.EF.percent*-1 #Change the sign to nega
tive for the left hand side of the barplot

c.bar.ef$Cum.Clim.EF <- c.bar.ef$Cum.Clim.EF*-1 #Change the sign to negative for the lef
t hand side of the barplot
c.bar.ef$Cum.Clim.EF.percent <- c.bar.ef$Cum.Clim.EF.percent*-1 #Change the sign to nega
tive for the left hand side of the barplot



#png(filename = "Figure4.EFBarplot.png", width = 7.5, height = 7.5, units = "in", res =
 300)

par(mfrow = c(1,2), pty = "s", mar = c(4,6,1,1))
barplot(height = n.bar.ef$Cum.Clim.EF.percent,
        xlim = c(-1,1),
        xaxt = "n",
        col = viridis(3),
        border = "white",
        horiz=T
        )
barplot(height = n.bar.ef$Dem.EF.percent,
        add=T,
        xaxt = "n",
        col = viridis(3),
        border = "white",
        horiz=T
        )

mtext(side = 3, at = -0.5, "Climate", cex = 0.75)
mtext(side = 3, at = 0.5, "Demography", cex = 0.75)
mtext(side = 2, at = 0.7, line = 0, las = 1, adj = 1, "Coastal", cex = 0.75)
mtext(side = 2, at = 1.9, line = 0, las = 1, adj = 1, "Mid-elevation", cex = 0.75)
mtext(side = 2, at = 3.1, line = 0, las = 1, adj = 1, "Highland", cex = 0.75)
mtext(side = 3, at = 0, line = 1, expression({delta}^15*N~'\u2030'), cex = 1)
mtext(side = 1, at = 0.95, line = -1, "A)", cex = 0.75)

axis(side = 1,
     at = seq(-1, 1, by = 0.25),
     c(1, 0.75, 0.5, 0.25, 0, 0.25, 0.5, 0.75, 1)
     )

par(mar=c(4,1,1,6))
barplot(height = c.bar.ef$Cum.Clim.EF.percent,
        xlim = c(-1,1),
```

```
        xaxt = "n",
        #names.arg = n.bar.ef$Elev.cat,
        col = viridis(3),
        border = "white",
        horiz=T
        )
barplot(height = c.bar.ef$Dem.EF.percent,
        add=T,
        xaxt = "n",
        col = viridis(3),
        border = "white",
        horiz=T
        )

mtext(side = 3, at = -0.5, "Climate", cex = 0.75)
mtext(side = 3, at = 0.5, "Demography", cex = 0.75)
mtext(side = 1, line = 3, at = -1.15, "Effect Size (% Variation Explained)", cex = 0.75)
mtext(side = 3, at = 0, line = 1, expression({delta}^13*C~'\u2030'), cex = 1)
mtext(side = 1, line = -1, at = 0.95, "B)", cex = 0.75)

axis(side = 1,
     at = seq(-1, 1, by = 0.25),
     c(1, 0.75, 0.5, 0.25, 0, 0.25, 0.5, 0.75, 1)
     )
```

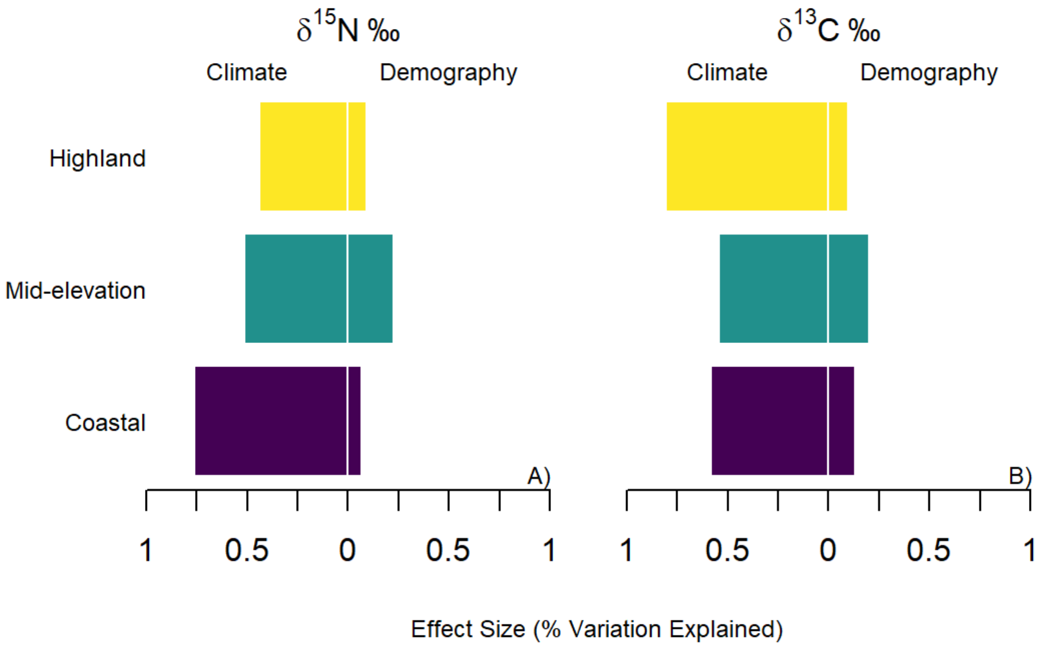

```
#dev.off()
```

Figure 4. Boxplots of the Cumulative effects of Climate and Demography by Elevation Zone

Random Forest Regression with Surovell Corrected KDE:

Assess if Observed Dietary Variation Results from Changing Population or Local Climate

Surovell et al. (2009) demonstrated that taphonomic loss can bias population reconstructions based upon Summed Probability Distributions due to differential loss of older dateable material. In response, they generated a correction equation which may be applied to demographic reconstructions based upon radiocarbon dates. In the Andes, some scholars have advocated for using this correction while others have not. Without a definitive answer as to whether corrected or uncorrected demographic reconstructions may be most appropriate, we here rerun the copmlete analyses above employing the corrected KDEs to evaluate if outcomes vary significantly based upon which demographic proxy employed.

First we analyze δ^{15}N‰.

To begin, subset the dataframe for just the variables included in the RF model.

```
dat.N <- data.N %>%
  dplyr::select(N.b.coll.,
         Temp_Mean,
         Precip_Mean,
         Temp_Mean_Seasonality,
         Precip_Mean_Seasonality,
         elev.factor,
         Demography.kde.corr.14c)#select the corrected KDE value
```

Now run RF regression to determine how well our suite of 6 variables predict δ^{15}N‰.

```
set.seed(33)#set a seed for replicability
#examine how bone N values vary as a function of all other variables in the data
RF.N <- ranger(N.b.coll. ~ ., dat.N)#Use RF regression to predict nitrogen values using
 each of the predictor variables

print(RF.N)
```

```
## Ranger result
##
## Call:
##  ranger(N.b.coll. ~ ., dat.N)
##
## Type:                             Regression
## Number of trees:                  500
## Sample size:                      1727
## Number of independent variables:  6
## Mtry:                             2
## Target node size:                 5
## Variable importance mode:         none
## Splitrule:                        variance
## OOB prediction error (MSE):       4.159674
## R squared (OOB):                  0.8002956
```

The RF model appears to do well at explaining the variance in bone collagen δ^{15}N‰. For validation purposes, we run multiple model performance evaluators below.

First, to better estimate how accurately our model predicts δ^{15}N‰, we use cross-validation following (123, 129, 130) to obtain the variance explained and root mean square error (RMSE).

```
myvars.N <- c("elev.factor", "Temp_Mean", "Precip_Mean", "Temp_Mean_Seasonality", "Preci
p_Mean_Seasonality", "Demography.kde.corr.14c")#select the key explanatory variables we
 are using in our analysis

pred.vars.N <- dat.N[myvars.N]#subset the isodat.N dataframe to keep only the predictor
 variables

set.seed(33)
RF.N.cv <- rgcv(pred.vars.N, dat.N$N.b.coll., cv.fold = 10)
RF.N.cv$rmse #root mean square error of cross-validated model
```

```
## [1] 2.038521
```

```
RF.N.cv$vecv #variance explained by the model based on cross-validation
```

```
## [1] 80.0377
```

Cross-validated results show that the median permuted percent variance explained is 80.04 meaning we explain 80.04% of the variability in bone collagen δ^{15}N‰ with our predictor variables. Mean RMSE is 2.04.

Next we check the model residuals for normalcy and temporal autcorrelation. To do so we first create a Prediction function for use with the ranger (RF) model as ranger has a non-standard predict function.

```
pred <- function(model, newdata)  {
  results <- as.data.frame(predict(model, newdata)$predictions)
  return(results)
}
```

Plot δ^{15}N‰ model residuals to check for normalcy:

```
pred.N <-  pred(RF.N, dat.N) #Use the pred function to predict the Nitrogen value for ea
ch individual

par(pty="s")

RF.resid.N <- dat.N$N.b.coll. - pred.N #calculate the residuals
colnames(RF.resid.N) <- "Residuals"

#bug <- as.numeric(unlist(RF.resid.N))

hist(RF.resid.N, col="grey", main=NA, xlab="Residuals")
```

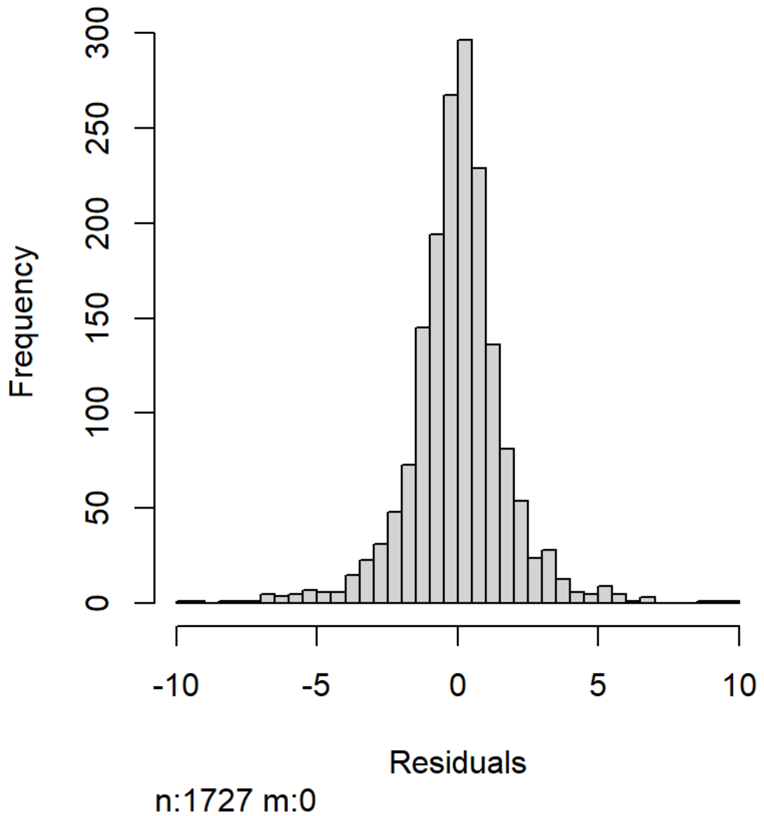

Fig. S10. δ^{15}N‰ RF Model Residuals. Residuals are normally distributed around 0.

Check for temporal autocorrelation in the δ^{15}N‰ residuals, following “Time after time: calculating the autocorrelation function for uneven or grouped time series” by Ariel Muldoon https://aosmith.rbind.io/2018/06/27/uneven-grouped-autocorrelation/ (https://aosmith.rbind.io/2018/06/27/uneven-grouped-autocorrelation/).

```
#Add the median year to the residuals dataframe for use in ordering individuals
RF.resid.N$median.date <- data.N$median.year

#Arrange the individuals by their median date
RF.resid.N.dat <- RF.resid.N %>% arrange(median.date)

#For individuals who have the same median year, aggregate their residuals into one obser
vation by taking the mean of each year with repeated individuals.
RF.resid.N.dat <- aggregate(RF.resid.N.dat, by = list(RF.resid.N.dat$median.date), FUN =
mean)

#Pad the dataset with NAs so each elevation zone has an equal number of rows. We want 1
 row per year from the minimum median date to the maximum median date across the full te
mporal spread. This is 385 to 5941 yBP.
RF.resid.N.dat.expand <- RF.resid.N.dat %>%
  complete(median.date = 385:5941)

corr <- acf(RF.resid.N.dat.expand$Residuals, lag.max=300,type="correlation",plot=TRUE,n
a.action=na.pass, main = "", xlab = "Lag (yrs)")#generate an acf plot of the residuals t
o visually assess temporal autocorrelation
mtext("All Individuals with Nitrogen", side = 3, line = 0.5)
```

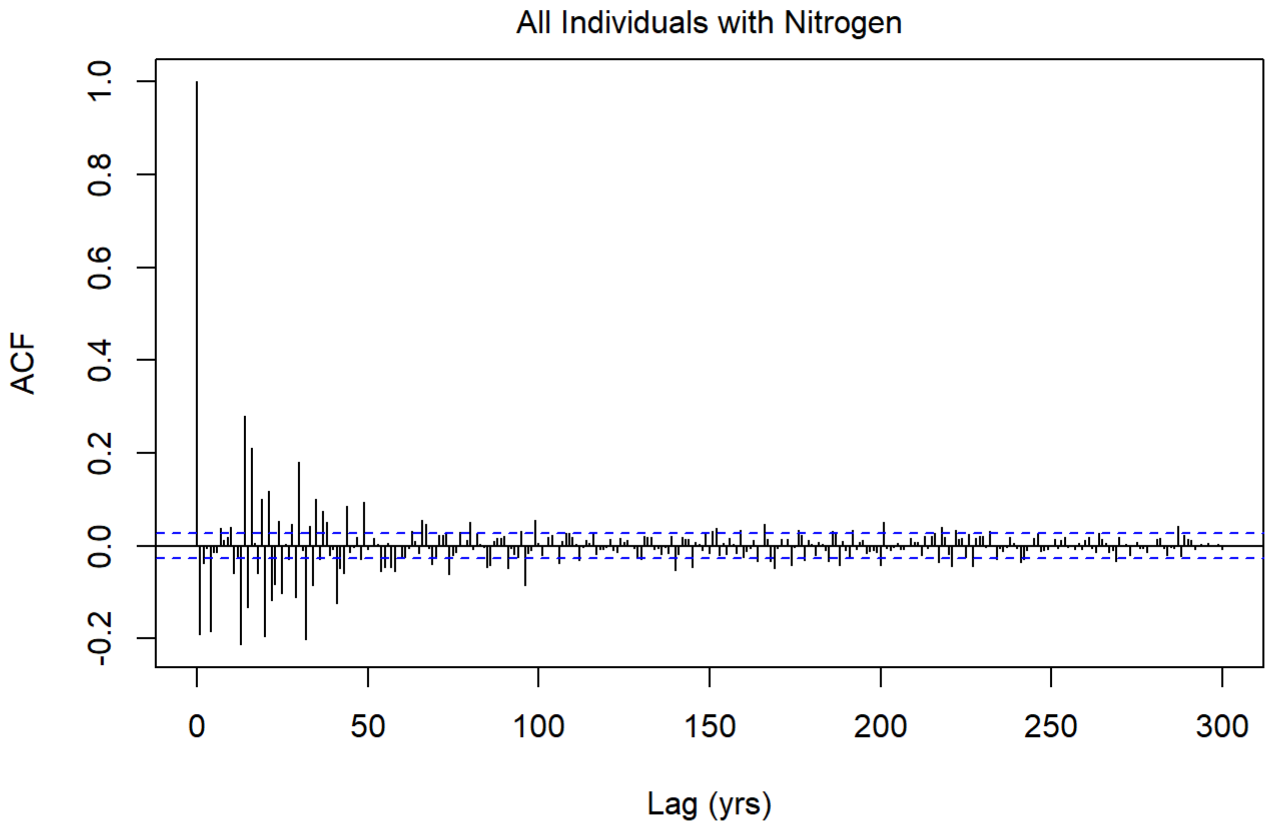

Fig. S11. Temporal Autocorrelation ACF Plot for δ^{15}N‰. ACF plot shows there is some temporal autocorrelation in the residuals up to about 200 years. This is not surprising given the way dates are assigned to individuals. The autocorrelation is not overly patterned but may exert some influence.

Check for Spatial Autocorrelation within the residuals.

```
#make a distance matrix
spat.dist <- as.matrix(dist(cbind(data.N$Latitude, data.N$Longitude)))
spat.dist.inv <- 1/spat.dist #inverse distance matrix
spat.dist.inv[is.infinite(spat.dist.inv)] <- 0 #set any infinate values to zero

#Check for spatial autocorrelation using global Moran's I
data.frame("Coordinates"=round(unlist(c(Moran.I(RF.resid.N$Residuals, spat.dist.inv, na.
rm=T))), 4)
           )
```

```
##          Coordinates
## observed     -0.0028
## expected     -0.0006
## sd            0.0029
## p.value       0.4548
```

We fail to reject the null hypothesis that there is no autocorrelation, this suggests the model adequately accounts for spatial autocorrelation and we do not have residual variation influencing results.

We evaluate the influence of each variable within each elevational cateogry by calcualting the effect size as the model sum of squares, discounted by the proportion of the variable's effect that results from its interaction with the other variables.

First make a predictor to use with the IML package:

```
mod <- Predictor$new(RF.N,
                     data = dat.N[-which(names(dat.N) == "N.b.coll.")],
                     y = dat.N$N.b.coll.,
                     predict.function = pred)
```

Calculate Friedman's H-Stat using IML. This is the proportion of the effect of each variable due to its interaction with the other variables for the complete model (not subset by elevational zone).

```
ia <- suppressMessages(Interaction$new(mod))
ia
```

```
## Interpretation method:  Interaction
##
##
## Analysed predictor:
## Prediction task: unknown
##
##
## Analysed data:
## Sampling from data.frame with 1727 rows and 6 columns.
##
## Head of results:
##                   .feature .interaction
## 1:               Temp_Mean    0.2390007
## 2:             Precip_Mean    0.2134842
## 3:   Temp_Mean_Seasonality    0.3292905
## 4: Precip_Mean_Seasonality    0.1617840
## 5:             elev.factor    0.1992742
## 6: Demography.kde.corr.14c    0.1830654
```

We are interested in how the variables predict δ^{15}N‰ within each of the elevational zones, however. To get the interaction and effect sizes within each elevational category, we take the below steps.

First, grab the data set

```
#This code grabs all of the input data for our RF model. This is the complete set of cas
es from our raw data (in other words, all 1679 individuals with nitrogen on bone collage
n)
data.sample <- ia$.__enclos_env__$private$sampler$get.x()
```

Second, set the batch size for processing (1000)

```
batch.size <- ia$predictor$batch.size
```

Third, set the grid size for calculating Partial Dependencies

```
#this is how many points within the chosen variable's observed range we use to calculate
the effect size. Using mean_temp as an example, a grid of 100 will equate to randomly ch
oosing 100 values of mean_temp. This then creates 100 repeated iterations of our complet
e dataset and assigns 1 of the 100 mean_temp values to the first copy of the dataset fol
lowed by assigning the 2nd mean_temp value to the second copy of the data set and so on.
This allows us to get the effect size for temp_mean by predicting $\delta$^15^N‰ for eve
ry individual at each of the 100 chosen mean_temp values.
grid.size <- ia$grid.size
grid.size <- 100
```

Fourth, set the mean function (required)

```
myq <- ia$.__enclos_env__$private$q
```

Fifth, choose which predictor variable to calculate

```
#Choose the variable of interest. This will need to be repeated for each of our predicto
r variables (Temp_Mean, Temp_Mean_Seasonality, Precip_Mean, Precip_Mean_Seasonality, and
Demography.spd)
feature.name <- c("Temp_Mean")
```

Sixth, set the prediction function

```
predictor <- ia$predictor
```

Seventh, get all the interactions for the chosen variable, calculate the Friedman's H-stat (proportion of the effect due to the variable's interaction with the other variables), calulate the effect size as the model sum of squares, discount the effect size based on the Friedman's H-stat, and calculate the effect size a a percent of the variation explained. For additional information on Friedman's H-stat, see (124). *Note: this code may take a long time (~10+ mins per variable) to run.*

```
#iterate the data sampling for interactions 100 times to increase accuracy of output.

runs <- seq(1, 100, 1)

#Make an object for the sampling output to go into
myia <- list(res = data.table(), qr = data.frame())

#Make a datafame object for the results of each iteration
N.Result.df <- data.frame()

#Make an empty dataframe for the total results to append to
Var.N.Result.df <- data.frame()



for (k in runs) {

  myia <- interaction.run.single(
  dataSample = data.sample,
  feature.name = feature.name[1],
  grid.size = grid.size,
  batch.size = batch.size,
  q = myq,
  predictor = predictor
  )


  partial_dat <- myia$res #make a dataframe with the data generated for the interactions
  partial_dat$.y.hat <- myia$qr #add the predicted values to the dataframe
  partial_dat$.class <- 1 #specify 1 so when we do the prediction R knows this is a regr
ession problem

  #collapse into 'F(x)' values - this will provide the average of all of the predictions
for each unique value of the chosen variable. So average of all predictions per observat
ion on grid and per value of the feature.
  pd.all <- data.table::dcast(partial_dat, .feature + .id + elev.factor + .class ~ .typ
e,
                        value.var = ".y.hat", fun.aggregate = mean
)

#We get a set of 300 predictions with pd.all, but we are missing 200 f and no.j values w
hich is because the chosen predictor value isn't present at 2 of the 3 elevational categ
ories. Therefore we remove the NA values, bringing us back to the 100 predictions we obt
ained. We can then subset the 100 predictions by which elevation factor they represent.
 This will give us a breakdown of the 100 into 3 subsets.
  pd.all.nona <- pd.all[which(!is.na(pd.all$f)),]#drop NA rows, leaving the 100 aggregat
ed predictions
  pd.all.nona.coast <- pd.all.nona[which(pd.all.nona$elev.factor == "Coastal"),]#Grab on
ly coastal cases.
  pd.all.nona.midel <- pd.all.nona[which(pd.all.nona$elev.factor == "Mid-elevation"),]#G
rab only mid-elevation cases.
  pd.all.nona.highl <- pd.all.nona[which(pd.all.nona$elev.factor == "Highland"),]#Grab o
```

```
nly highland cases.

  #Calculate Total Response of Nitrogen
  f.all <- scale(pd.all.nona$f, scale = FALSE)
  f.coast <- scale(pd.all.nona.coast$f, scale = FALSE)
  f.midel <- scale(pd.all.nona.midel$f, scale = FALSE)
  f.highl <- scale(pd.all.nona.highl$f, scale = FALSE)

  #Calculate Partial Dependence of Nitrogen for Chosen Variable
  f.j <- scale(pd.all.nona$j, scale = FALSE)
  f.j.coast <- scale(pd.all.nona.coast$j, scale = FALSE)
  f.j.midel <- scale(pd.all.nona.midel$j, scale = FALSE)
  f.j.highl <- scale(pd.all.nona.highl$j, scale = FALSE)

  #Calculate Combined Partial Dependence of Nitrogen to all Other Variables
  f.no.j <- scale(pd.all.nona$no.j, scale = FALSE)
  f.noj.coast <- scale(pd.all.nona.coast$no.j, scale = FALSE)
  f.noj.midel <- scale(pd.all.nona.midel$no.j, scale = FALSE)
  f.noj.highl <- scale(pd.all.nona.highl$no.j, scale = FALSE)

  #Calculate Friedman's H-Stat to get the proportion of the effect due to interaction wi
th the other variables
  h.all <- sqrt(sum((f.all - (f.j + f.no.j))^2) / sum(f.all^2))
  h.coast <- sqrt(sum((f.coast - (f.j.coast + f.noj.coast))^2) / sum(f.coast^2))
  h.midel <- sqrt(sum((f.midel - (f.j.midel + f.noj.midel))^2) / sum(f.midel^2))
  h.highl <- sqrt(sum((f.highl - (f.j.highl + f.noj.highl))^2) / sum(f.highl^2))

  #Assign the Friedman's H-Stat to a dataframe
  N.Result.df <- data.frame(var = "Temp Mean", H.Stat = h.all, H.Stat.Coast = h.coast,
 H.Stat.Mid = h.midel, H.Stat.High = h.highl)

  #Model Effect Size of the Chosen Variable (Amount of the per mil variance explained by
the chosen variable)
  sqrt(sum((f.j)^2))
  sqrt(sum((f.j.coast)^2))
  sqrt(sum((f.j.midel)^2))
  sqrt(sum((f.j.highl)^2))

  #Add the effect size to the dataframe
  meant.EF <- data.frame(EF = sqrt(sum((f.j)^2)), EF.coast = sqrt(sum((f.j.coast)^2)), E
F.midel = sqrt(sum((f.j.midel)^2)), EF.high = sqrt(sum((f.j.highl)^2)))
  N.Result.df <- cbind(N.Result.df, meant.EF)

  #Percent Effect of the Chosen Variable (Percent of the variance in Nitrogen explained
 by the chosen variable)
  sqrt(sum(f.j^2) / sum(f.all^2))
  sqrt(sum(f.j.coast^2) / sum(f.coast^2))
  sqrt(sum(f.j.midel^2) / sum(f.midel^2))
  sqrt(sum(f.j.highl^2) / sum(f.highl^2))

  #Assign the percent effect size to the dataframe
  meant.EF.per <- data.frame(EF.per = sqrt(sum(f.j^2) / sum(f.all^2)), EF.per.coast = sq
```

```
rt(sum(f.j.coast^2) / sum(f.coast^2)), EF.per.midel = sqrt(sum(f.j.midel^2) / sum(f.mide
l^2)), EF.per.high = sqrt(sum(f.j.highl^2) / sum(f.highl^2)))
  N.Result.df <- cbind(N.Result.df, meant.EF.per)

  #Now use the Friedman's H-Stat to control the effect size and percent to get the amoun
t of the variance explained by the chosen variable without its interaction with other va
riables
  #Effect Size Controlled
  sqrt(sum((f.j*(1-h.all))^2))
  sqrt(sum((f.j.coast*(1-h.coast))^2))
  sqrt(sum((f.j.midel*(1-h.midel))^2))
  sqrt(sum((f.j.highl*(1-h.highl))^2))

  #Assign the controlled effect size to a dataframe
  meant.EF.cont <- data.frame(EF.cont = sqrt(sum((f.j*(1-h.all))^2)), EF.cont.coast = sq
rt(sum((f.j.coast*(1-h.coast))^2)), EF.cont.midel = sqrt(sum((f.j.midel*(1-h.midel))^2
)), EF.cont.highl = sqrt(sum((f.j.highl*(1-h.highl))^2)))
  N.Result.df <- cbind(N.Result.df, meant.EF.cont)

  #Percent Effect of the Chosen Variable Controlled with Friedman's H-Stat
  #Effect Size Percent Controlled
  sqrt(sum((f.j*(1-h.all))^2) / sum(f.all^2))
  sqrt(sum((f.j.coast*(1-h.coast))^2) / sum(f.coast^2))
  sqrt(sum((f.j.midel*(1-h.midel))^2) / sum(f.midel^2))
  sqrt(sum((f.j.highl*(1-h.highl))^2) / sum(f.highl^2))

    #Assign the controlled percent effect size to a dataframe
  meant.EF.per.cont <- data.frame(EF.per.cont = sqrt(sum((f.j*(1-h.all))^2) / sum(f.all^
2)), EF.per.cont.coast = sqrt(sum((f.j.coast*(1-h.coast))^2) / sum(f.coast^2)), EF.per.c
ont.midel = sqrt(sum((f.j.midel*(1-h.midel))^2) / sum(f.midel^2)), EF.per.cont.highl = s
qrt(sum((f.j.highl*(1-h.highl))^2) / sum(f.highl^2)))
  N.Result.df <- cbind(N.Result.df, meant.EF.per.cont)


  if (k == 1) {
    Var.N.Result.df <- N.Result.df
  }
  else {
  Var.N.Result.df <- rbind(Var.N.Result.df, N.Result.df)
  }
}

#You can check to see if the output is correct - there should be 100 rows of 21 columns
#Var.N.Result.df

#Average the 21 columns and assign the values to a new dataframe. This gives us the aver
age effect sizes based on 100 iterations of randomly sampling 100 values for the chosen
 variable, assigning those values to each individual, and predicting their N value.
Total.N.Result.df <- data.frame(row.names = "Temp Mean", t(colMeans(Var.N.Result.df[2:21
])))
```

Eighth, repeat for each of the other predictor variables.

Repeat for Mean Precip Vector of features to calculate

```
#Choose the variable of interest. This will need to be repeated for each of our predicto
r variables (Temp_Mean, Temp_Mean_Seasonality, Precip_Mean, Precip_Mean_Seasonality, and
Demography.kde.corr.14c)
feature.name <- c("Precip_Mean")
```

Prediction function

```
predictor <- ia$predictor
```

Now get all the interactions for the chosen variable, calculate the Friedman's H-stat (proportion of the effect due to the variable's interaction with the other variables), calulate the effect size as the model sum of squares, discount the effect size based on the Friedman's H-stat, and calculate the effect size a a percent of the variation explained.

```
#iterate the data sampling for interactions 100 times.
runs <- seq(1, 100, 1)

#Make an object for the sampling output to go into
myia <- list(res = data.table(), qr = data.frame())

#Make a datafame object for the results of each iteration
N.Result.df <- data.frame()

#Make an empty dataframe for the total results to append to
Var.N.Result.df <- data.frame()



for (k in runs) {

  myia <- interaction.run.single(
  dataSample = data.sample,
  feature.name = feature.name[1],
  grid.size = grid.size,
  batch.size = batch.size,
  q = myq,
  predictor = predictor
  )


  partial_dat <- myia$res #make a dataframe with the data generated for the interactions
  partial_dat$.y.hat <- myia$qr #add the predicted values to the dataframe
  partial_dat$.class <- 1 #specify 1 so when we do the prediction R knows this is a regr
ession problem

  #collapse into 'F(x)' values - this will provide the average of all of the predictions
for each unique value of the chosen variable. So average of all predictions per observat
ion on grid and per value of the feature.
  pd.all <- data.table::dcast(partial_dat, .feature + .id + elev.factor + .class ~ .typ
e,
                        value.var = ".y.hat", fun.aggregate = mean
)

  pd.all.nona <- pd.all[which(!is.na(pd.all$f)),]#drop NA rows, leaving the 100 aggregat
ed predictions
  pd.all.nona.coast <- pd.all.nona[which(pd.all.nona$elev.factor == "Coastal"),]#Grab on
ly coastal cases.
  pd.all.nona.midel <- pd.all.nona[which(pd.all.nona$elev.factor == "Mid-elevation"),]#G
rab only mid-elevation cases.
  pd.all.nona.highl <- pd.all.nona[which(pd.all.nona$elev.factor == "Highland"),]#Grab o
nly highland cases.

  #Calculate Total Response of Nitrogen
  f.all <- scale(pd.all.nona$f, scale = FALSE)
  f.coast <- scale(pd.all.nona.coast$f, scale = FALSE)
  f.midel <- scale(pd.all.nona.midel$f, scale = FALSE)
```

```
  f.highl <- scale(pd.all.nona.highl$f, scale = FALSE)

  #Calculate Partial Dependence of Nitrogen for Chosen Variable
  f.j <- scale(pd.all.nona$j, scale = FALSE)
  f.j.coast <- scale(pd.all.nona.coast$j, scale = FALSE)
  f.j.midel <- scale(pd.all.nona.midel$j, scale = FALSE)
  f.j.highl <- scale(pd.all.nona.highl$j, scale = FALSE)

  #Calculate Combined Partial Dependence of Nitrogen to all Other Variables
  f.no.j <- scale(pd.all.nona$no.j, scale = FALSE)
  f.noj.coast <- scale(pd.all.nona.coast$no.j, scale = FALSE)
  f.noj.midel <- scale(pd.all.nona.midel$no.j, scale = FALSE)
  f.noj.highl <- scale(pd.all.nona.highl$no.j, scale = FALSE)

  #Calculate Friedman's H-Stat to get the proportion of the effect due to interaction wi
th the other variables
  h.all <- sqrt(sum((f.all - (f.j + f.no.j))^2) / sum(f.all^2))
  h.coast <- sqrt(sum((f.coast - (f.j.coast + f.noj.coast))^2) / sum(f.coast^2))
  h.midel <- sqrt(sum((f.midel - (f.j.midel + f.noj.midel))^2) / sum(f.midel^2))
  h.highl <- sqrt(sum((f.highl - (f.j.highl + f.noj.highl))^2) / sum(f.highl^2))

  #Assign the Friedman's H-Stat to a dataframe
  N.Result.df <- data.frame(var = "Precip Mean", H.Stat = h.all, H.Stat.Coast = h.coast,
H.Stat.Mid = h.midel, H.Stat.High = h.highl)

  #Model Effect Size of the Chosen Variable (Amount of the per mil variance explained by
the chosen variable)
  sqrt(sum((f.j)^2))
  sqrt(sum((f.j.coast)^2))
  sqrt(sum((f.j.midel)^2))
  sqrt(sum((f.j.highl)^2))

  #Add the effect size to the dataframe
  meant.EF <- data.frame(EF = sqrt(sum((f.j)^2)), EF.coast = sqrt(sum((f.j.coast)^2)), E
F.midel = sqrt(sum((f.j.midel)^2)), EF.high = sqrt(sum((f.j.highl)^2)))
  N.Result.df <- cbind(N.Result.df, meant.EF)

  #Percent Effect of the Chosen Variable (Percent of the variance in Nitrogen explained
 by the chosen variable)
  sqrt(sum(f.j^2) / sum(f.all^2))
  sqrt(sum(f.j.coast^2) / sum(f.coast^2))
  sqrt(sum(f.j.midel^2) / sum(f.midel^2))
  sqrt(sum(f.j.highl^2) / sum(f.highl^2))

  #Assign the percent effect size to the dataframe
  meant.EF.per <- data.frame(EF.per = sqrt(sum(f.j^2) / sum(f.all^2)), EF.per.coast = sq
rt(sum(f.j.coast^2) / sum(f.coast^2)), EF.per.midel = sqrt(sum(f.j.midel^2) / sum(f.mide
l^2)), EF.per.high = sqrt(sum(f.j.highl^2) / sum(f.highl^2)))
  N.Result.df <- cbind(N.Result.df, meant.EF.per)

  #Now use the Friedman's H-Stat to control the effect size and percent to get the amoun
t of the variance explained by the chosen variable without its interaction with other va
```

```
riables
  #Effect Size Controlled
  sqrt(sum((f.j*(1-h.all))^2))
  sqrt(sum((f.j.coast*(1-h.coast))^2))
  sqrt(sum((f.j.midel*(1-h.midel))^2))
  sqrt(sum((f.j.highl*(1-h.highl))^2))

  #Assign the controlled effect size to a dataframe
  meant.EF.cont <- data.frame(EF.cont = sqrt(sum((f.j*(1-h.all))^2)), EF.cont.coast = sq
rt(sum((f.j.coast*(1-h.coast))^2)), EF.cont.midel = sqrt(sum((f.j.midel*(1-h.midel))^2
)), EF.cont.highl = sqrt(sum((f.j.highl*(1-h.highl))^2)))
  N.Result.df <- cbind(N.Result.df, meant.EF.cont)

  #Percent Effect of the Chosen Variable Controlled with Friedman's H-Stat
  #Effect Size Percent Controlled
  sqrt(sum((f.j*(1-h.all))^2) / sum(f.all^2))
  sqrt(sum((f.j.coast*(1-h.coast))^2) / sum(f.coast^2))
  sqrt(sum((f.j.midel*(1-h.midel))^2) / sum(f.midel^2))
  sqrt(sum((f.j.highl*(1-h.highl))^2) / sum(f.highl^2))

  #Assign the controlled percent effect size to a dataframe
  meant.EF.per.cont <- data.frame(EF.per.cont = sqrt(sum((f.j*(1-h.all))^2) / sum(f.all^
2)), EF.per.cont.coast = sqrt(sum((f.j.coast*(1-h.coast))^2) / sum(f.coast^2)), EF.per.c
ont.midel = sqrt(sum((f.j.midel*(1-h.midel))^2) / sum(f.midel^2)), EF.per.cont.highl = s
qrt(sum((f.j.highl*(1-h.highl))^2) / sum(f.highl^2)))
  N.Result.df <- cbind(N.Result.df, meant.EF.per.cont)


  if (k == 1) {
    Var.N.Result.df <- N.Result.df
  }
  else {
  Var.N.Result.df <- rbind(Var.N.Result.df, N.Result.df)
  }
}

#Grab the column averages
Var.avg <- data.frame(row.names = "Precip Mean", t(colMeans(Var.N.Result.df[2:21])))

#Bind the resul to the Total Effect Size dataframe
Total.N.Result.df <- rbind(Total.N.Result.df, Var.avg)
```

Repeat for Precip Seasonality Vector of features to calculate

```
#Choose the variable of interest. This will need to be repeated for each of our predicto
r variables (Temp_Mean, Temp_Mean_Seasonality, Precip_Mean, Precip_Mean_Seasonality, and
Demography.spd)
feature.name <- c("Precip_Mean_Seasonality")
```

Prediction function

```
predictor <- ia$predictor
```

Now get all the interactions for the chosen variable, calculate the Friedman's H-stat (proportion of the effect due to the variable's interaction with the other variables), calulate the effect size as the model sum of squares, discount the effect size based on the Friedman's H-stat, and calculate the effect size a a percent of the variation explained.

```
#iterate the data sampling for interactions 100 times.

runs <- seq(1, 100, 1)

#Make an object for the sampling output to go into
myia <- list(res = data.table(), qr = data.frame())

#Make a datafame object for the results of each iteration
N.Result.df <- data.frame()

#Make an empty dataframe for the total results to append to
Var.N.Result.df <- data.frame()



for (k in runs) {

  myia <- interaction.run.single(
  dataSample = data.sample,
  feature.name = feature.name[1],
  grid.size = grid.size,
  batch.size = batch.size,
  q = myq,
  predictor = predictor
  )


  partial_dat <- myia$res#make a dataframe with the data generated for the interactions
  partial_dat$.y.hat <- myia$qr#add the predicted values to the dataframe
  partial_dat$.class <- 1#specify 1 so when we do the prediction R knows this is a regre
ssion problem

  #collapse into 'F(x)' values - this will provide the average of all of the predictions
for each unique value of the chosen variable. So average of all predictions per observat
ion on grid and per value of the feature.
  pd.all <- data.table::dcast(partial_dat, .feature + .id + elev.factor + .class ~ .typ
e,
                        value.var = ".y.hat", fun.aggregate = mean
)

  pd.all.nona <- pd.all[which(!is.na(pd.all$f)),]#drop NA rows, leaving the 100 aggregat
ed predictions
  pd.all.nona.coast <- pd.all.nona[which(pd.all.nona$elev.factor == "Coastal"),]#Grab on
ly coastal cases.
  pd.all.nona.midel <- pd.all.nona[which(pd.all.nona$elev.factor == "Mid-elevation"),]#G
rab only mid-elevation cases.
  pd.all.nona.highl <- pd.all.nona[which(pd.all.nona$elev.factor == "Highland"),]#Grab o
nly highland cases.

  #Calculate Total Response of Nitrogen
  f.all <- scale(pd.all.nona$f, scale = FALSE)
  f.coast <- scale(pd.all.nona.coast$f, scale = FALSE)
```

```
  f.midel <- scale(pd.all.nona.midel$f, scale = FALSE)
  f.highl <- scale(pd.all.nona.highl$f, scale = FALSE)

  #Calculate Partial Dependence of Nitrogen for Chosen Variable
  f.j <- scale(pd.all.nona$j, scale = FALSE)
  f.j.coast <- scale(pd.all.nona.coast$j, scale = FALSE)
  f.j.midel <- scale(pd.all.nona.midel$j, scale = FALSE)
  f.j.highl <- scale(pd.all.nona.highl$j, scale = FALSE)

  #Calculate Combined Partial Dependence of Nitrogen to all Other Variables
  f.no.j <- scale(pd.all.nona$no.j, scale = FALSE)
  f.noj.coast <- scale(pd.all.nona.coast$no.j, scale = FALSE)
  f.noj.midel <- scale(pd.all.nona.midel$no.j, scale = FALSE)
  f.noj.highl <- scale(pd.all.nona.highl$no.j, scale = FALSE)

  #Calculate Friedman's H-Stat to get the proportion of the effect due to interaction wi
th the other variables
  h.all <- sqrt(sum((f.all - (f.j + f.no.j))^2) / sum(f.all^2))
  h.coast <- sqrt(sum((f.coast - (f.j.coast + f.noj.coast))^2) / sum(f.coast^2))
  h.midel <- sqrt(sum((f.midel - (f.j.midel + f.noj.midel))^2) / sum(f.midel^2))
  h.highl <- sqrt(sum((f.highl - (f.j.highl + f.noj.highl))^2) / sum(f.highl^2))

  #Assign the Friedman's H-Stat to a dataframe
  N.Result.df <- data.frame(var = "Precip Mean Seasonality", H.Stat = h.all, H.Stat.Coas
t = h.coast, H.Stat.Mid = h.midel, H.Stat.High = h.highl)

  #Model Effect Size of the Chosen Variable (Amount of the per mil variance explained by
the chosen variable)
  sqrt(sum((f.j)^2))
  sqrt(sum((f.j.coast)^2))
  sqrt(sum((f.j.midel)^2))
  sqrt(sum((f.j.highl)^2))

  #Add the effect size to the dataframe
  meant.EF <- data.frame(EF = sqrt(sum((f.j)^2)), EF.coast = sqrt(sum((f.j.coast)^2)), E
F.midel = sqrt(sum((f.j.midel)^2)), EF.high = sqrt(sum((f.j.highl)^2)))
  N.Result.df <- cbind(N.Result.df, meant.EF)

  #Percent Effect of the Chosen Variable (Percent of the variance in Nitrogen explained
 by the chosen variable)
  sqrt(sum(f.j^2) / sum(f.all^2))
  sqrt(sum(f.j.coast^2) / sum(f.coast^2))
  sqrt(sum(f.j.midel^2) / sum(f.midel^2))
  sqrt(sum(f.j.highl^2) / sum(f.highl^2))

  #Assign the percent effect size to the dataframe
  meant.EF.per <- data.frame(EF.per = sqrt(sum(f.j^2) / sum(f.all^2)), EF.per.coast = sq
rt(sum(f.j.coast^2) / sum(f.coast^2)), EF.per.midel = sqrt(sum(f.j.midel^2) / sum(f.mide
l^2)), EF.per.high = sqrt(sum(f.j.highl^2) / sum(f.highl^2)))
  N.Result.df <- cbind(N.Result.df, meant.EF.per)

  #Now use the Friedman's H-Stat to control the effect size and percent to get the amoun
```

```
t of the variance explained by the chosen variable without its interaction with other va
riables
  #Effect Size Controlled
  sqrt(sum((f.j*(1-h.all))^2))
  sqrt(sum((f.j.coast*(1-h.coast))^2))
  sqrt(sum((f.j.midel*(1-h.midel))^2))
  sqrt(sum((f.j.highl*(1-h.highl))^2))

  #Assign the controlled effect size to a dataframe
  meant.EF.cont <- data.frame(EF.cont = sqrt(sum((f.j*(1-h.all))^2)), EF.cont.coast = sq
rt(sum((f.j.coast*(1-h.coast))^2)), EF.cont.midel = sqrt(sum((f.j.midel*(1-h.midel))^2
)), EF.cont.highl = sqrt(sum((f.j.highl*(1-h.highl))^2)))
  N.Result.df <- cbind(N.Result.df, meant.EF.cont)

  #Percent Effect of the Chosen Variable Controlled with Friedman's H-Stat
  #Effect Size Percent Controlled
  sqrt(sum((f.j*(1-h.all))^2) / sum(f.all^2))
  sqrt(sum((f.j.coast*(1-h.coast))^2) / sum(f.coast^2))
  sqrt(sum((f.j.midel*(1-h.midel))^2) / sum(f.midel^2))
  sqrt(sum((f.j.highl*(1-h.highl))^2) / sum(f.highl^2))

  #Assign the controlled percent effect size to a dataframe
  meant.EF.per.cont <- data.frame(EF.per.cont = sqrt(sum((f.j*(1-h.all))^2) / sum(f.all^
2)), EF.per.cont.coast = sqrt(sum((f.j.coast*(1-h.coast))^2) / sum(f.coast^2)), EF.per.c
ont.midel = sqrt(sum((f.j.midel*(1-h.midel))^2) / sum(f.midel^2)), EF.per.cont.highl = s
qrt(sum((f.j.highl*(1-h.highl))^2) / sum(f.highl^2)))
  N.Result.df <- cbind(N.Result.df, meant.EF.per.cont)


  if (k == 1) {
    Var.N.Result.df <- N.Result.df
  }
  else {
  Var.N.Result.df <- rbind(Var.N.Result.df, N.Result.df)
  }
}


#Grab the column averages
Var.avg <- data.frame(row.names = "Precip Mean Seasonality", t(colMeans(Var.N.Result.df[
2:21])))

#Bind the resul to the Total Effect Size dataframe
Total.N.Result.df <- rbind(Total.N.Result.df, Var.avg)
```

Repeat for Temp Seasonality Vector of features to calculate

```
#Choose the variable of interest. This will need to be repeated for each of our predicto
r variables (Temp_Mean, Temp_Mean_Seasonality, Precip_Mean, Precip_Mean_Seasonality, and
Demography.spd)
feature.name <- c("Temp_Mean_Seasonality")
```

Prediction function

```
predictor <- ia$predictor
```

Now get all the interactions for the chosen variable, calculate the Friedman's H-stat (proportion of the effect due to the variable's interaction with the other variables), calulate the effect size as the model sum of squares, discount the effect size based on the Friedman's H-stat, and calculate the effect size a a percent of the variation explained.

```
#iterate the data sampling for interactions 100 times.

runs <- seq(1, 100, 1)

#Make an object for the sampling output to go into
myia <- list(res = data.table(), qr = data.frame())

#Make a datafame object for the results of each iteration
N.Result.df <- data.frame()

#Make an empty dataframe for the total results to append to
Var.N.Result.df <- data.frame()



for (k in runs) {

  myia <- interaction.run.single(
  dataSample = data.sample,
  feature.name = feature.name[1],
  grid.size = grid.size,
  batch.size = batch.size,
  q = myq,
  predictor = predictor
  )


  partial_dat <- myia$res#make a dataframe with the data generated for the interactions
  partial_dat$.y.hat <- myia$qr#add the predicted values to the dataframe
  partial_dat$.class <- 1#specify 1 so when we do the prediction R knows this is a regre
ssion problem

  #collapse into 'F(x)' values - this will provide the average of all of the predictions
for each unique value of the chosen variable. So average of all predictions per observat
ion on grid and per value of the feature.
  pd.all <- data.table::dcast(partial_dat, .feature + .id + elev.factor + .class ~ .typ
e,
                        value.var = ".y.hat", fun.aggregate = mean
)

  pd.all.nona <- pd.all[which(!is.na(pd.all$f)),]#drop NA rows, leaving the 100 aggregat
ed predictions
  pd.all.nona.coast <- pd.all.nona[which(pd.all.nona$elev.factor == "Coastal"),]#Grab on
ly coastal cases.
  pd.all.nona.midel <- pd.all.nona[which(pd.all.nona$elev.factor == "Mid-elevation"),]#G
rab only mid-elevation cases.
  pd.all.nona.highl <- pd.all.nona[which(pd.all.nona$elev.factor == "Highland"),]#Grab o
nly highland cases.

  #Calculate Total Response of Nitrogen
  f.all <- scale(pd.all.nona$f, scale = FALSE)
  f.coast <- scale(pd.all.nona.coast$f, scale = FALSE)
```

```
  f.midel <- scale(pd.all.nona.midel$f, scale = FALSE)
  f.highl <- scale(pd.all.nona.highl$f, scale = FALSE)

  #Calculate Partial Dependence of Nitrogen for Chosen Variable
  f.j <- scale(pd.all.nona$j, scale = FALSE)
  f.j.coast <- scale(pd.all.nona.coast$j, scale = FALSE)
  f.j.midel <- scale(pd.all.nona.midel$j, scale = FALSE)
  f.j.highl <- scale(pd.all.nona.highl$j, scale = FALSE)

  #Calculate Combined Partial Dependence of Nitrogen to all Other Variables
  f.no.j <- scale(pd.all.nona$no.j, scale = FALSE)
  f.noj.coast <- scale(pd.all.nona.coast$no.j, scale = FALSE)
  f.noj.midel <- scale(pd.all.nona.midel$no.j, scale = FALSE)
  f.noj.highl <- scale(pd.all.nona.highl$no.j, scale = FALSE)

  #Calculate Friedman's H-Stat to get the proportion of the effect due to interaction wi
th the other variables
  h.all <- sqrt(sum((f.all - (f.j + f.no.j))^2) / sum(f.all^2))
  h.coast <- sqrt(sum((f.coast - (f.j.coast + f.noj.coast))^2) / sum(f.coast^2))
  h.midel <- sqrt(sum((f.midel - (f.j.midel + f.noj.midel))^2) / sum(f.midel^2))
  h.highl <- sqrt(sum((f.highl - (f.j.highl + f.noj.highl))^2) / sum(f.highl^2))

  #Assign the Friedman's H-Stat to a dataframe
  N.Result.df <- data.frame(var = "Temp Mean Seasonality", H.Stat = h.all, H.Stat.Coast
 = h.coast, H.Stat.Mid = h.midel, H.Stat.High = h.highl)

  #Model Effect Size of the Chosen Variable (Amount of the per mil variance explained by
the chosen variable)
  sqrt(sum((f.j)^2))
  sqrt(sum((f.j.coast)^2))
  sqrt(sum((f.j.midel)^2))
  sqrt(sum((f.j.highl)^2))

  #Add the effect size to the dataframe
  meant.EF <- data.frame(EF = sqrt(sum((f.j)^2)), EF.coast = sqrt(sum((f.j.coast)^2)), E
F.midel = sqrt(sum((f.j.midel)^2)), EF.high = sqrt(sum((f.j.highl)^2)))
  N.Result.df <- cbind(N.Result.df, meant.EF)

  #Percent Effect of the Chosen Variable (Percent of the variance in Nitrogen explained
 by the chosen variable)
  sqrt(sum(f.j^2) / sum(f.all^2))
  sqrt(sum(f.j.coast^2) / sum(f.coast^2))
  sqrt(sum(f.j.midel^2) / sum(f.midel^2))
  sqrt(sum(f.j.highl^2) / sum(f.highl^2))

  #Assign the percent effect size to the dataframe
  meant.EF.per <- data.frame(EF.per = sqrt(sum(f.j^2) / sum(f.all^2)), EF.per.coast = sq
rt(sum(f.j.coast^2) / sum(f.coast^2)), EF.per.midel = sqrt(sum(f.j.midel^2) / sum(f.mide
l^2)), EF.per.high = sqrt(sum(f.j.highl^2) / sum(f.highl^2)))
  N.Result.df <- cbind(N.Result.df, meant.EF.per)

  #Now use the Friedman's H-Stat to control the effect size and percent to get the amoun
```

```
t of the variance explained by the chosen variable without its interaction with other va
riables
  #Effect Size Controlled
  sqrt(sum((f.j*(1-h.all))^2))
  sqrt(sum((f.j.coast*(1-h.coast))^2))
  sqrt(sum((f.j.midel*(1-h.midel))^2))
  sqrt(sum((f.j.highl*(1-h.highl))^2))

  #Assign the controlled effect size to a dataframe
  meant.EF.cont <- data.frame(EF.cont = sqrt(sum((f.j*(1-h.all))^2)), EF.cont.coast = sq
rt(sum((f.j.coast*(1-h.coast))^2)), EF.cont.midel = sqrt(sum((f.j.midel*(1-h.midel))^2
)), EF.cont.highl = sqrt(sum((f.j.highl*(1-h.highl))^2)))
  N.Result.df <- cbind(N.Result.df, meant.EF.cont)

  #Percent Effect of the Chosen Variable Controlled with Friedman's H-Stat
  #Effect Size Percent Controlled
  sqrt(sum((f.j*(1-h.all))^2) / sum(f.all^2))
  sqrt(sum((f.j.coast*(1-h.coast))^2) / sum(f.coast^2))
  sqrt(sum((f.j.midel*(1-h.midel))^2) / sum(f.midel^2))
  sqrt(sum((f.j.highl*(1-h.highl))^2) / sum(f.highl^2))

  #Assign the controlled percent effect size to a dataframe
  meant.EF.per.cont <- data.frame(EF.per.cont = sqrt(sum((f.j*(1-h.all))^2) / sum(f.all^
2)), EF.per.cont.coast = sqrt(sum((f.j.coast*(1-h.coast))^2) / sum(f.coast^2)), EF.per.c
ont.midel = sqrt(sum((f.j.midel*(1-h.midel))^2) / sum(f.midel^2)), EF.per.cont.highl = s
qrt(sum((f.j.highl*(1-h.highl))^2) / sum(f.highl^2)))
  N.Result.df <- cbind(N.Result.df, meant.EF.per.cont)


  if (k == 1) {
    Var.N.Result.df <- N.Result.df
  }
  else {
  Var.N.Result.df <- rbind(Var.N.Result.df, N.Result.df)
  }
}


#Grab the column averages (this will be the 100 runs of 100 different var values average
d together)
Var.avg <- data.frame(row.names = "Temp Mean Seasonality", t(colMeans(Var.N.Result.df[2:
21])))

#Bind the resul to the Total Effect Size dataframe
Total.N.Result.df <- rbind(Total.N.Result.df, Var.avg)

#Generate the Cumulative Climate Effect
Cum.Clim. <- data.frame(row.names = "Cumulative Climate", t(colSums(Total.N.Result.df)))

#Add Cumulative Climate to the Total Effect Size Dataframe
Total.N.Result.df <- rbind(Total.N.Result.df, Cum.Clim.)
```

```
#Pull Out the Key Effect Sizes to store in their own dataframe
Cumulative.EF <- data.frame(Elev.cat = c("Coastal", "Mid-elevation", "Highland"), Cum.Cl
im.EF = c(Total.N.Result.df[5,14], Total.N.Result.df[5,15], Total.N.Result.df[5,16]), Cu
m.Clim.EF.percent = c(Total.N.Result.df[5,18], Total.N.Result.df[5,19], Total.N.Result.d
f[5,20]))
```

Repeat for Demography Vector of features to calculate

```
#Choose the variable of interest. This will need to be repeated for each of our predicto
r variables (Temp_Mean, Temp_Mean_Seasonality, Precip_Mean, Precip_Mean_Seasonality, and
Demography.spd)
feature.name <- c("Demography.kde.corr.14c")
```

Prediction function

```
predictor <- ia$predictor
```

Now get all the interactions for the chosen variable, calculate the Friedman’s H-stat (proportion of the effect due to the variable’s interaction with the other variables), calulate the effect size as the model sum of squares, discount the effect size based on the Friedman’s H-stat, and calculate the effect size a a percent of the variation explained.

```
#iterate the data sampling for interactions 100 times.

runs <- seq(1, 100, 1)

#Make an object for the sampling output to go into
myia <- list(res = data.table(), qr = data.frame())

#Make a datafame object for the results of each iteration
N.Result.df <- data.frame()

#Make an empty dataframe for the total results to append to
Var.N.Result.df <- data.frame()


for (k in runs) {

  myia <- interaction.run.single(
  dataSample = data.sample,
  feature.name = feature.name[1],
  grid.size = grid.size,
  batch.size = batch.size,
  q = myq,
  predictor = predictor
  )


  partial_dat <- myia$res#make a dataframe with the data generated for the interactions
  partial_dat$.y.hat <- myia$qr#add the predicted values to the dataframe
  partial_dat$.class <- 1#specify 1 so when we do the prediction R knows this is a regre
ssion problem

  #collapse into 'F(x)' values - this will provide the average of all of the predictions
for each unique value of the chosen variable. So average of all predictions per observat
ion on grid and per value of the feature.
  pd.all <- data.table::dcast(partial_dat, .feature + .id + elev.factor + .class ~ .typ
e,
                        value.var = ".y.hat", fun.aggregate = mean
)

  pd.all.nona <- pd.all[which(!is.na(pd.all$f)),]#drop NA rows, leaving the 100 aggregat
ed predictions
  pd.all.nona.coast <- pd.all.nona[which(pd.all.nona$elev.factor == "Coastal"),]#Grab on
ly coastal cases.
  pd.all.nona.midel <- pd.all.nona[which(pd.all.nona$elev.factor == "Mid-elevation"),]#G
rab only mid-elevation cases.
  pd.all.nona.highl <- pd.all.nona[which(pd.all.nona$elev.factor == "Highland"),]#Grab o
nly highland cases.

  #Calculate Total Response of Nitrogen
  f.all <- scale(pd.all.nona$f, scale = FALSE)
  f.coast <- scale(pd.all.nona.coast$f, scale = FALSE)
  f.midel <- scale(pd.all.nona.midel$f, scale = FALSE)
```

```
  f.highl <- scale(pd.all.nona.highl$f, scale = FALSE)

  #Calculate Partial Dependence of Nitrogen for Chosen Variable
  f.j <- scale(pd.all.nona$j, scale = FALSE)
  f.j.coast <- scale(pd.all.nona.coast$j, scale = FALSE)
  f.j.midel <- scale(pd.all.nona.midel$j, scale = FALSE)
  f.j.highl <- scale(pd.all.nona.highl$j, scale = FALSE)

  #Calculate Combined Partial Dependence of Nitrogen to all Other Variables
  f.no.j <- scale(pd.all.nona$no.j, scale = FALSE)
  f.noj.coast <- scale(pd.all.nona.coast$no.j, scale = FALSE)
  f.noj.midel <- scale(pd.all.nona.midel$no.j, scale = FALSE)
  f.noj.highl <- scale(pd.all.nona.highl$no.j, scale = FALSE)

  #Calculate Friedman's H-Stat to get the proportion of the effect due to interaction wi
th the other variables
  h.all <- sqrt(sum((f.all - (f.j + f.no.j))^2) / sum(f.all^2))
  h.coast <- sqrt(sum((f.coast - (f.j.coast + f.noj.coast))^2) / sum(f.coast^2))
  h.midel <- sqrt(sum((f.midel - (f.j.midel + f.noj.midel))^2) / sum(f.midel^2))
  h.highl <- sqrt(sum((f.highl - (f.j.highl + f.noj.highl))^2) / sum(f.highl^2))

  #Assign the Friedman's H-Stat to a dataframe
  N.Result.df <- data.frame(var = "Demography", H.Stat = h.all, H.Stat.Coast = h.coast,
 H.Stat.Mid = h.midel, H.Stat.High = h.highl)

  #Model Effect Size of the Chosen Variable (Amount of the per mil variance explained by
the chosen variable)
  sqrt(sum((f.j)^2))
  sqrt(sum((f.j.coast)^2))
  sqrt(sum((f.j.midel)^2))
  sqrt(sum((f.j.highl)^2))

  #Add the effect size to the dataframe
  meant.EF <- data.frame(EF = sqrt(sum((f.j)^2)), EF.coast = sqrt(sum((f.j.coast)^2)), E
F.midel = sqrt(sum((f.j.midel)^2)), EF.high = sqrt(sum((f.j.highl)^2)))
  N.Result.df <- cbind(N.Result.df, meant.EF)

  #Percent Effect of the Chosen Variable (Percent of the variance in Nitrogen explained
 by the chosen variable)
  sqrt(sum(f.j^2) / sum(f.all^2))
  sqrt(sum(f.j.coast^2) / sum(f.coast^2))
  sqrt(sum(f.j.midel^2) / sum(f.midel^2))
  sqrt(sum(f.j.highl^2) / sum(f.highl^2))

  #Assign the percent effect size to the dataframe
  meant.EF.per <- data.frame(EF.per = sqrt(sum(f.j^2) / sum(f.all^2)), EF.per.coast = sq
rt(sum(f.j.coast^2) / sum(f.coast^2)), EF.per.midel = sqrt(sum(f.j.midel^2) / sum(f.mide
l^2)), EF.per.high = sqrt(sum(f.j.highl^2) / sum(f.highl^2)))
  N.Result.df <- cbind(N.Result.df, meant.EF.per)

  #Now use the Friedman's H-Stat to control the effect size and percent to get the amoun
t of the variance explained by the chosen variable without its interaction with other va
```

```
riables
  #Effect Size Controlled
  sqrt(sum((f.j*(1-h.all))^2))
  sqrt(sum((f.j.coast*(1-h.coast))^2))
  sqrt(sum((f.j.midel*(1-h.midel))^2))
  sqrt(sum((f.j.highl*(1-h.highl))^2))

  #Assign the controlled effect size to a dataframe
  meant.EF.cont <- data.frame(EF.cont = sqrt(sum((f.j*(1-h.all))^2)), EF.cont.coast = sq
rt(sum((f.j.coast*(1-h.coast))^2)), EF.cont.midel = sqrt(sum((f.j.midel*(1-h.midel))^2
)), EF.cont.highl = sqrt(sum((f.j.highl*(1-h.highl))^2)))
  N.Result.df <- cbind(N.Result.df, meant.EF.cont)

  #Percent Effect of the Chosen Variable Controlled with Friedman's H-Stat
  #Effect Size Percent Controlled
  sqrt(sum((f.j*(1-h.all))^2) / sum(f.all^2))
  sqrt(sum((f.j.coast*(1-h.coast))^2) / sum(f.coast^2))
  sqrt(sum((f.j.midel*(1-h.midel))^2) / sum(f.midel^2))
  sqrt(sum((f.j.highl*(1-h.highl))^2) / sum(f.highl^2))

  #Assign the controlled percent effect size to a dataframe
  meant.EF.per.cont <- data.frame(EF.per.cont = sqrt(sum((f.j*(1-h.all))^2) / sum(f.all^
2)), EF.per.cont.coast = sqrt(sum((f.j.coast*(1-h.coast))^2) / sum(f.coast^2)), EF.per.c
ont.midel = sqrt(sum((f.j.midel*(1-h.midel))^2) / sum(f.midel^2)), EF.per.cont.highl = s
qrt(sum((f.j.highl*(1-h.highl))^2) / sum(f.highl^2)))
  N.Result.df <- cbind(N.Result.df, meant.EF.per.cont)


  if (k == 1) {
    Var.N.Result.df <- N.Result.df
  }
  else {
  Var.N.Result.df <- rbind(Var.N.Result.df, N.Result.df)
  }
}


#Grab the column averages (this will be the 100 runs of 100 different var values average
d together)
Var.avg <- data.frame(row.names = "Demography", t(colMeans(Var.N.Result.df[2:21])))

#Bind the resul to the Total Effect Size dataframe
Total.N.Result.df <- rbind(Total.N.Result.df, Var.avg)

#Add demography results to the cumulative effect size dataframe
Cumulative.EF$Dem.EF <- c(sqrt(sum((f.j.coast*(1-h.coast))^2)), sqrt(sum((f.j.midel*(1-
h.midel))^2)), sqrt(sum((f.j.highl*(1-h.highl))^2)))
Cumulative.EF$Dem.EF.percent <- c(sqrt(sum((f.j.coast*(1-h.coast))^2) / sum(f.coast^2)),
sqrt(sum((f.j.midel*(1-h.midel))^2) / sum(f.midel^2)), sqrt(sum((f.j.highl*(1-h.highl))^
2) / sum(f.highl^2)))
```

Show the complete set of effect size calculations

```
#show the complete set of effect size calculations - for each variable, this is: H-stat, Effect Size, Effect Size percent, Effect size controlled by H-stat, Effect size percent controlled by H-stat
round(Total.N.Result.df,2)
```

```
##                          H.Stat H.Stat.Coast H.Stat.Mid H.Stat.High     EF
## Temp Mean                  0.57         0.25       0.33        0.52  23.23
## Precip Mean                0.43         0.25       0.28        0.40  29.09
## Precip Mean Seasonality    0.70         0.22       0.24        0.23  27.35
## Temp Mean Seasonality      0.51         0.25       0.37        0.42  25.20
## Cumulative Climate         2.20         0.96       1.21        1.58 104.88
## Demography                 0.59         0.17       0.30        0.29  24.56
##                          EF.coast EF.midel EF.high EF.per EF.per.coast
## Temp Mean                    2.25     1.79    1.04   0.57         0.09
## Precip Mean                 13.33     6.94    0.74   0.71         0.52
## Precip Mean Seasonality      1.62     1.97    0.87   0.67         0.06
## Temp Mean Seasonality        8.81     3.12    1.00   0.61         0.35
## Cumulative Climate          26.00    13.82    3.65   2.57         1.03
## Demography                   1.72     1.84    0.80   0.61         0.07
##                          EF.per.midel EF.per.high EF.cont EF.cont.coast
## Temp Mean                        0.10        0.23   10.01          1.68
## Precip Mean                      0.40        0.16   16.60         10.07
## Precip Mean Seasonality          0.12        0.18    8.28          1.26
## Temp Mean Seasonality            0.18        0.22   12.48          6.65
## Cumulative Climate               0.80        0.78   47.37         19.66
## Demography                       0.11        0.17    9.99          1.42
##                          EF.cont.midel EF.cont.highl EF.per.cont
## Temp Mean                         1.20          0.50        0.24
## Precip Mean                       5.04          0.45        0.41
## Precip Mean Seasonality           1.50          0.67        0.20
## Temp Mean Seasonality             1.97          0.58        0.30
## Cumulative Climate                9.71          2.20        1.15
## Demography                        1.28          0.56        0.25
##                          EF.per.cont.coast EF.per.cont.midel EF.per.cont.highl
## Temp Mean                             0.07              0.07              0.11
## Precip Mean                           0.39              0.29              0.09
## Precip Mean Seasonality               0.05              0.09              0.14
## Temp Mean Seasonality                 0.26              0.11              0.13
## Cumulative Climate                    0.77              0.56              0.47
## Demography                            0.06              0.07              0.12
```

Table S3. Complete Effect Size Calcuations for δ^{15}N‰. For each variable we report the complete model H-stat and effect size as well as the H-stat and effect sizes within each elevational zone.

```
N.Results <- Total.N.Result.df[,c(14:16, 18:20)]
round(N.Results, 4)
```

```
##                           EF.cont.coast EF.cont.midel EF.cont.highl
## Temp Mean                        1.6780        1.2028        0.4973
## Precip Mean                     10.0677        5.0416        0.4479
## Precip Mean Seasonality          1.2606        1.5029        0.6727
## Temp Mean Seasonality            6.6533        1.9654        0.5782
## Cumulative Climate              19.6596        9.7127        2.1961
## Demography                       1.4174        1.2821        0.5594
##                           EF.per.cont.coast EF.per.cont.midel EF.per.cont.highl
## Temp Mean                            0.0667            0.0693            0.1083
## Precip Mean                          0.3936            0.2869            0.0938
## Precip Mean Seasonality              0.0500            0.0879            0.1422
## Temp Mean Seasonality                0.2639            0.1149            0.1253
## Cumulative Climate                   0.7742            0.5590            0.4696
## Demography                           0.0569            0.0742            0.1163
```

Table 2 Part 1. δ^{15}N‰ Effect Sizes. *Note: as these results are generated through many iterations the results will not exactly match those in the main manuscript text. Despite small fluctuations the ranking of variables and effect sizes do not change significantly when re-running the code.*

Now we repeat all of the above for δ^{13}C‰.

To begin, subset the dataframe for just the variables included in the RF model.

```
dat.C <- data.C %>%
  dplyr::select(C.b.coll.,
         Temp_Mean,
         Precip_Mean,
         Temp_Mean_Seasonality,
         Precip_Mean_Seasonality,
         elev.factor,
         Demography.kde.corr.14c)
```

Now run RF regression to determine how well our suite of 6 variables predict δ^{13}C‰.

```
set.seed(33)#set a seed for replicability
#examine how bone C values vary as a function of all other variables in the data
RF.C <- ranger(C.b.coll. ~ ., dat.C)#Use RF regression to predict nitrogen values using
 each of the predictor variables

print(RF.C)
```

```
## Ranger result
##
## Call:
##  ranger(C.b.coll. ~ ., dat.C)
##
## Type:                             Regression
## Number of trees:                  500
## Sample size:                      1761
## Number of independent variables:  6
## Mtry:                             2
## Target node size:                 5
## Variable importance mode:         none
## Splitrule:                        variance
## OOB prediction error (MSE):       2.55251
## R squared (OOB):                  0.6692432
```

The RF model appears to do well at explaining the variance in bone collagen δ^{13}C‰. For validation purposes, we run multiple model performance evaluators below.

First, to better estimate how accurately our model predicts δ^{13}C‰, we use cross-validation following (123, 129, 130) to obtain the variance explained and root mean square error (RMSE).

```
myvars.C <- c("elev.factor", "Temp_Mean", "Precip_Mean", "Temp_Mean_Seasonality", "Preci
p_Mean_Seasonality", "Demography.kde.corr.14c")#select the key explanatory variables we
 are using in our analysis

pred.vars.C <- dat.C[myvars.C]#subset the dat.C dataframe to keep only the predictor var
iables

set.seed(33)
RF.C.cv <- rgcv(pred.vars.C, dat.C$C.b.coll., cv.fold = 10)
RF.C.cv$rmse #root mean square error of cross-validated model
```

```
## [1] 1.600937
```

```
RF.C.cv$vecv #variance explained by the model based on cross-validation
```

```
## [1] 66.7695
```

Results show that the median permuted percent variance explained is 66.77 meaning we explain 66.77% of the variability in bone collagen δ^{13}C‰ with our predictor variables. Mean RMSE is 1.60.

Next we check the model residuals for normalcy and temporal autcorrelation. Plot δ^{13}C‰ model residuals to check for normalcy:

```
pred.C <-  pred(RF.C, dat.C) #Use the pred function to predict the Nitrogen value for ea
ch individual

par(pty="s")

RF.resid.C <- dat.C$C.b.coll. - pred.C #calculate the residuals
colnames(RF.resid.C) <- "Residuals"

hist(RF.resid.C, col="grey", main=NA, xlab="Residuals")
```

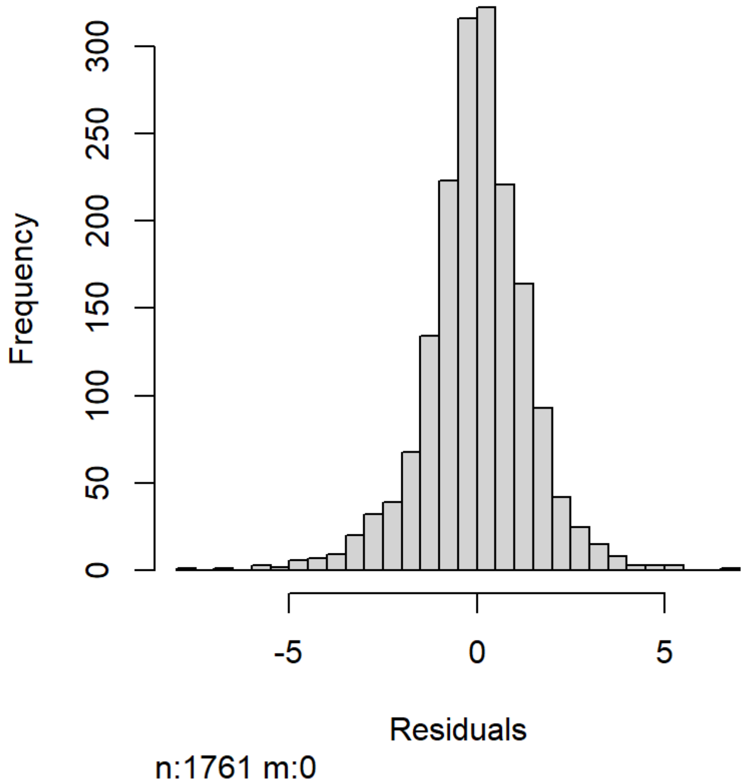

Fig. S12. δ^{13}C‰ RF Model Residuals. Residuals are normally distributed around 0.

Check for temporal autocorrelation in the δ^{13}C‰ residuals, following “Time after time: calculating the autocorrelation function for uneven or grouped time series” by Ariel Muldoon https://aosmith.rbind.io/2018/06/27/uneven-grouped-autocorrelation/ (https://aosmith.rbind.io/2018/06/27/uneven-grouped-autocorrelation/).

```
#Add the median year to the residuals dataframe for use in ordering individuals
RF.resid.C$median.date <- data.C$median.year

#Arrange the individuals by their median date
RF.resid.C.dat <- RF.resid.C %>% arrange(median.date)

#For individuals who have the same median year, aggregate their residuals into one obser
vation by taking the mean of each year with repeated individuals.
RF.resid.C.dat <- aggregate(RF.resid.C.dat, by = list(RF.resid.C.dat$median.date), FUN =
mean)

#Pad the dataset with NAs so each elevation zone has an equal number of rows. We want 1
 row per year from the minimum median date to the maximum median date across the full te
mporal spread. This is 385 to 5941 yBP.
RF.resid.C.dat.expand <- RF.resid.C.dat %>%
  complete(median.date = 385:5941)

corr <- acf(RF.resid.C.dat.expand$Residuals, lag.max=300,type="correlation",plot=TRUE,n
a.action=na.pass, main = "", xlab = "Lag (yrs)")#generate an acf plot of the residuals t
o visually assess temporal autocorrelation
mtext("All Individuals with Carbon", side = 3, line = 0.5)
```

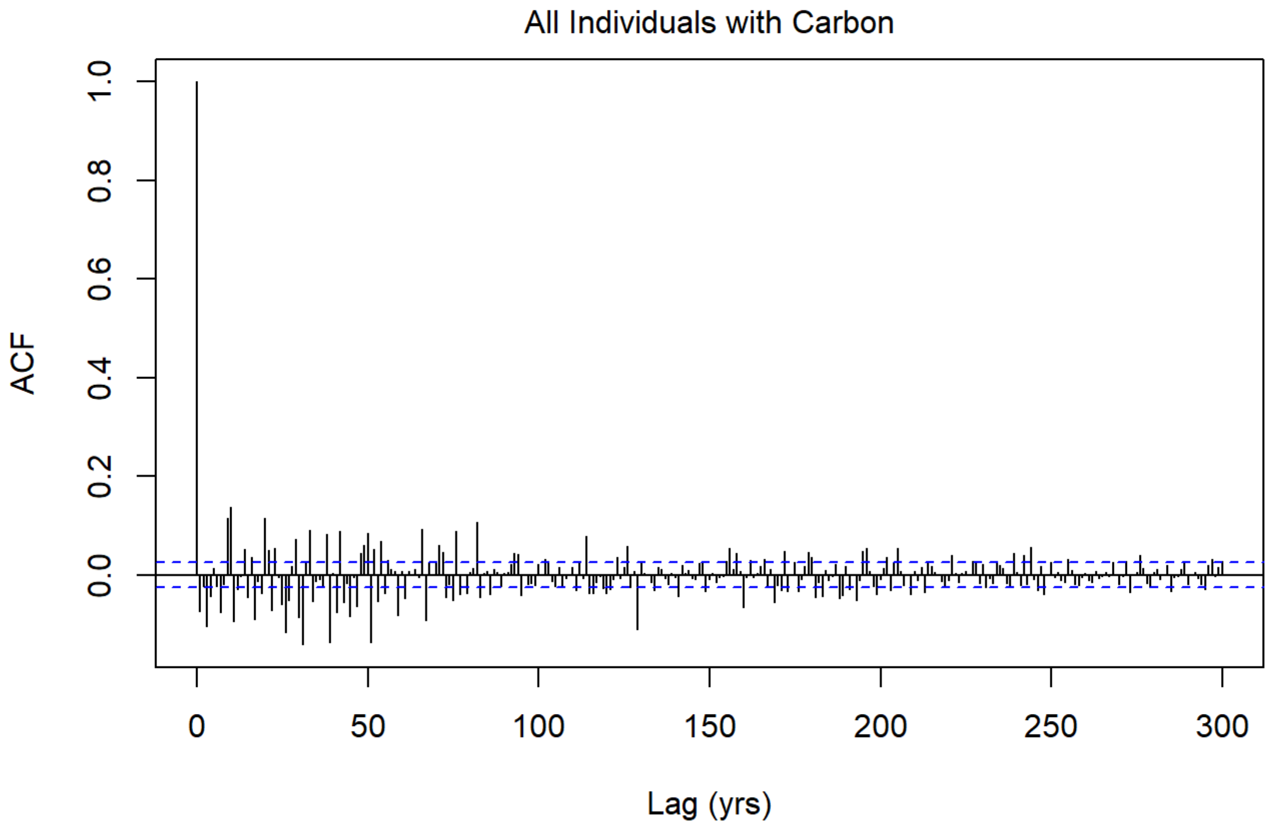

Fig. S13. Temporal Autocorellation ACF Plot. ACF plot shows there is some temporal autocorrelation in the residuals up to about 200 years. This is not surprising given the way dates are assigned to individuals. The autocorrelation is not overly patterned but may exert some influence.

Check for Spatial Autocorrelation within the residuals.

```
#make a distance matrix
spat.dist <- as.matrix(dist(cbind(data.C$Latitude, data.C$Longitude)))
spat.dist.inv <- 1/spat.dist #inverse distance matrix
spat.dist.inv[is.infinite(spat.dist.inv)] <- 0 #set any infinate values to zero

#Check for spatial autocorrelation using global Moran's I
data.frame("Coordinates"=round(unlist(c(Moran.I(RF.resid.C$Residuals, spat.dist.inv, na.
rm=T))), 4)
           )
```

```
##          Coordinates
## observed     -0.0009
## expected     -0.0006
## sd            0.0029
## p.value       0.8960
```

We find no evidence of spatial autocorrelation in the residuals.

We evaluate the influence of each variable within each elevational cateogry by calcualting the effect size as the model sum of squares, discounted by the proportion of the variable's effect that results from its interaction with the other variables - the same as for δ^{15}N‰.

Make a predictor to use with the IML package:

```
mod <- Predictor$new(RF.C,
                     data = dat.C[-which(names(dat.C) == "C.b.coll.")],
                     y = dat.C$C.b.coll.,
                     predict.function = pred)
```

Calculate Friedman's H-Stat using IML. This is the proportion of the effect of each variable due to its interaction with the other variables for the complete model (not subset by elevational zone).

```
#Friedman's H-Stat proportion of the effect of each variable due to its interaction with
the others.
ia <- Interaction$new(mod)
ia
```

```
## Interpretation method:  Interaction
##
##
## Analysed predictor:
## Prediction task: unknown
##
##
## Analysed data:
## Sampling from data.frame with 1761 rows and 6 columns.
##
## Head of results:
##                    .feature .interaction
## 1:                Temp_Mean    0.2665168
## 2:              Precip_Mean    0.2716398
## 3:   Temp_Mean_Seasonality    0.2648589
## 4: Precip_Mean_Seasonality    0.3139748
## 5:              elev.factor    0.3489818
## 6: Demography.kde.corr.14c    0.2407896
```

Note that this is the Friedman's H-stat for the total model, not within each elevational category. We are interested in how the variables predict δ^{13}C‰ within each of the elevational zones. To get the interaction and effect sizes within each elevational category, we take the below steps.

First, grab the data set

```
#This code grabs all of the input data for our RF model. This is the complete set of cas
es from our raw data (in other words, all 1784 individuals with nitrogen on bone collage
n)
data.sample <- ia$.__enclos_env__$private$sampler$get.x()
```

Second, set the batch size for processing (1000)

```
batch.size <- ia$predictor$batch.size
```

Third, set the grid size for calculating Partial Dependencies

```
grid.size <- ia$grid.size
grid.size <- 100
```

Fourth, set the mean function (required

```
myq <- ia$.__enclos_env__$private$q
```

Fifth, choose which predictor variable to calculate

```
#Choose the variable of interest. This will need to be repeated for each of our predicto
r variables (Temp_Mean, Temp_Mean_Seasonality, Precip_Mean, Precip_Mean_Seasonality, and
Demography.spd)
feature.name <- c("Temp_Mean")
```

Sixth, set the prediction function

```
predictor <- ia$predictor
```

Seventh, get all the interactions for the chosen variable, calculate the Friedman’s H-stat (proportion of the effect due to the variable’s interaction with the other variables), calulate the effect size as the model sum of squares, discount the effect size based on the Friedman’s H-stat, and calculate the effect size a a percent of the variation explained. For additional information on Friedman’s H-stat, see (124). *Note: this code may take a long time (~10+ mins per variable) to run.*

```
#iterate the data sampling for interactions 100 times.
runs <- seq(1, 100, 1)

#Make an object for the sampling output to go into
myia <- list(res = data.table(), qr = data.frame())

#Make a datafame object for the results of each iteration
C.Result.df <- data.frame()

#Make an empty dataframe for the total results to append to
Var.C.Result.df <- data.frame()


for (k in runs) {

  myia <- interaction.run.single(
  dataSample = data.sample,
  feature.name = feature.name[1],
  grid.size = grid.size,
  batch.size = batch.size,
  q = myq,
  predictor = predictor
  )


  partial_dat <- myia$res#make a dataframe with the data generated for the interactions
  partial_dat$.y.hat <- myia$qr#add the predicted values to the dataframe
  partial_dat$.class <- 1#specify 1 so when we do the prediction R knows this is a regre
ssion problem

  #collapse into 'F(x)' values - this will provide the average of all of the predictions
for each unique value of the chosen variable. So average of all predictions per observat
ion on grid and per value of the feature.
  pd.all <- data.table::dcast(partial_dat, .feature + .id + elev.factor + .class ~ .typ
e,
                        value.var = ".y.hat", fun.aggregate = mean
)

  pd.all.nona <- pd.all[which(!is.na(pd.all$f)),]#drop NA rows, leaving the 100 aggregat
ed predictions
  pd.all.nona.coast <- pd.all.nona[which(pd.all.nona$elev.factor == "Coastal"),]#Grab on
ly coastal cases.
  pd.all.nona.midel <- pd.all.nona[which(pd.all.nona$elev.factor == "Mid-elevation"),]#G
rab only mid-elevation cases.
  pd.all.nona.highl <- pd.all.nona[which(pd.all.nona$elev.factor == "Highland"),]#Grab o
nly highland cases.

  #Calculate Total Response of Carbon
  f.all <- scale(pd.all.nona$f, scale = FALSE)
  f.coast <- scale(pd.all.nona.coast$f, scale = FALSE)
  f.midel <- scale(pd.all.nona.midel$f, scale = FALSE)
  f.highl <- scale(pd.all.nona.highl$f, scale = FALSE)
```

```
  #Calculate Partial Dependence of Carbon for Chosen Variable
  f.j <- scale(pd.all.nona$j, scale = FALSE)
  f.j.coast <- scale(pd.all.nona.coast$j, scale = FALSE)
  f.j.midel <- scale(pd.all.nona.midel$j, scale = FALSE)
  f.j.highl <- scale(pd.all.nona.highl$j, scale = FALSE)

  #Calculate Combined Partial Dependence of Carbon to all Other Variables
  f.no.j <- scale(pd.all.nona$no.j, scale = FALSE)
  f.noj.coast <- scale(pd.all.nona.coast$no.j, scale = FALSE)
  f.noj.midel <- scale(pd.all.nona.midel$no.j, scale = FALSE)
  f.noj.highl <- scale(pd.all.nona.highl$no.j, scale = FALSE)

  #Calculate Friedman's H-Stat to get the proportion of the effect due to interaction wi
th the other variables
  h.all <- sqrt(sum((f.all - (f.j + f.no.j))^2) / sum(f.all^2))
  h.coast <- sqrt(sum((f.coast - (f.j.coast + f.noj.coast))^2) / sum(f.coast^2))
  h.midel <- sqrt(sum((f.midel - (f.j.midel + f.noj.midel))^2) / sum(f.midel^2))
  h.highl <- sqrt(sum((f.highl - (f.j.highl + f.noj.highl))^2) / sum(f.highl^2))

  #Assign the Friedman's H-Stat to a dataframe
  C.Result.df <- data.frame(var = "Temp Mean", H.Stat = h.all, H.Stat.Coast = h.coast,
 H.Stat.Mid = h.midel, H.Stat.High = h.highl)

  #Model Effect Size of the Chosen Variable (Amount of the per mil variance explained by
the chosen variable)
  sqrt(sum((f.j)^2))
  sqrt(sum((f.j.coast)^2))
  sqrt(sum((f.j.midel)^2))
  sqrt(sum((f.j.highl)^2))

  #Add the effect size to the dataframe
  meant.EF <- data.frame(EF = sqrt(sum((f.j)^2)), EF.coast = sqrt(sum((f.j.coast)^2)), E
F.midel = sqrt(sum((f.j.midel)^2)), EF.high = sqrt(sum((f.j.highl)^2)))
  C.Result.df <- cbind(C.Result.df, meant.EF)

  #Percent Effect of the Chosen Variable (Percent of the variance in Carbon explained by
the chosen variable)
  sqrt(sum(f.j^2) / sum(f.all^2))
  sqrt(sum(f.j.coast^2) / sum(f.coast^2))
  sqrt(sum(f.j.midel^2) / sum(f.midel^2))
  sqrt(sum(f.j.highl^2) / sum(f.highl^2))

  #Assign the percent effect size to the dataframe
  meant.EF.per <- data.frame(EF.per = sqrt(sum(f.j^2) / sum(f.all^2)), EF.per.coast = sq
rt(sum(f.j.coast^2) / sum(f.coast^2)), EF.per.midel = sqrt(sum(f.j.midel^2) / sum(f.mide
l^2)), EF.per.high = sqrt(sum(f.j.highl^2) / sum(f.highl^2)))
  C.Result.df <- cbind(C.Result.df, meant.EF.per)

  #Now use the Friedman's H-Stat to control the effect size and percent to get the amoun
t of the variance explained by the chosen variable without its interaction with other va
riables
```

```
  #Effect Size Controlled
  sqrt(sum((f.j*(1-h.all))^2))
  sqrt(sum((f.j.coast*(1-h.coast))^2))
  sqrt(sum((f.j.midel*(1-h.midel))^2))
  sqrt(sum((f.j.highl*(1-h.highl))^2))

  #Assign the controlled effect size to a dataframe
  meant.EF.cont <- data.frame(EF.cont = sqrt(sum((f.j*(1-h.all))^2)), EF.cont.coast = sq
rt(sum((f.j.coast*(1-h.coast))^2)), EF.cont.midel = sqrt(sum((f.j.midel*(1-h.midel))^2
)), EF.cont.highl = sqrt(sum((f.j.highl*(1-h.highl))^2)))
  C.Result.df <- cbind(C.Result.df, meant.EF.cont)

  #Percent Effect of the Chosen Variable Controlled with Friedman's H-Stat
  #Effect Size Percent Controlled
  sqrt(sum((f.j*(1-h.all))^2) / sum(f.all^2))
  sqrt(sum((f.j.coast*(1-h.coast))^2) / sum(f.coast^2))
  sqrt(sum((f.j.midel*(1-h.midel))^2) / sum(f.midel^2))
  sqrt(sum((f.j.highl*(1-h.highl))^2) / sum(f.highl^2))

  #Assign the controlled percent effect size to a dataframe
  meant.EF.per.cont <- data.frame(EF.per.cont = sqrt(sum((f.j*(1-h.all))^2) / sum(f.all^
2)), EF.per.cont.coast = sqrt(sum((f.j.coast*(1-h.coast))^2) / sum(f.coast^2)), EF.per.c
ont.midel = sqrt(sum((f.j.midel*(1-h.midel))^2) / sum(f.midel^2)), EF.per.cont.highl = s
qrt(sum((f.j.highl*(1-h.highl))^2) / sum(f.highl^2)))
  C.Result.df <- cbind(C.Result.df, meant.EF.per.cont)


  if (k == 1) {
    Var.C.Result.df <- C.Result.df
  }
  else {
  Var.C.Result.df <- rbind(Var.C.Result.df, C.Result.df)
  }
}

#Average the 21 columns and assign the values to a new dataframe. This gives us the aver
age effect sizes based on 100 iterations of randomly sampling 100 values for the chosen
 variable, assigning those values to each individual, and predicting their C value.
Total.C.Result.df <- data.frame(row.names = "Temp Mean", t(colMeans(Var.C.Result.df[2:21
])))
```

Eighth, repeat for each of the other predictor variables.

Repeat for Mean Precip

```
#Choose the variable of interest. This will need to be repeated for each of our predicto
r variables (Temp_Mean, Temp_Mean_Seasonality, Precip_Mean, Precip_Mean_Seasonality, and
Demography.spd)
feature.name <- c("Precip_Mean")
```

Prediction function

```
predictor <- ia$predictor
```

Now get all the interactions for the chosen variable, calculate the Friedman's H-stat (proportion of the effect due to the variable's interaction with the other variables), calulate the effect size as the model sum of squares, discount the effect size based on the Friedman's H-stat, and calculate the effect size a a percent of the variation explained.

```
#iterate the data sampling for interactions 100 times.
runs <- seq(1, 100, 1)

#Make an object for the sampling output to go into
myia <- list(res = data.table(), qr = data.frame())

#Make a datafame object for the results of each iteration
C.Result.df <- data.frame()

#Make an empty dataframe for the total results to append to
Var.C.Result.df <- data.frame()


for (k in runs) {

  myia <- interaction.run.single(
  dataSample = data.sample,
  feature.name = feature.name[1],
  grid.size = grid.size,
  batch.size = batch.size,
  q = myq,
  predictor = predictor
  )


  partial_dat <- myia$res#make a dataframe with the data generated for the interactions
  partial_dat$.y.hat <- myia$qr#add the predicted values to the dataframe
  partial_dat$.class <- 1#specify 1 so when we do the prediction R knows this is a regre
ssion problem

  #collapse into 'F(x)' values - this will provide the average of all of the predictions
for each unique value of the chosen variable. So average of all predictions per observat
ion on grid and per value of the feature.
  pd.all <- data.table::dcast(partial_dat, .feature + .id + elev.factor + .class ~ .typ
e,
                        value.var = ".y.hat", fun.aggregate = mean
)

  pd.all.nona <- pd.all[which(!is.na(pd.all$f)),]#drop NA rows, leaving the 100 aggregat
ed predictions
  pd.all.nona.coast <- pd.all.nona[which(pd.all.nona$elev.factor == "Coastal"),]#Grab on
ly coastal cases.
  pd.all.nona.midel <- pd.all.nona[which(pd.all.nona$elev.factor == "Mid-elevation"),]#G
rab only mid-elevation cases.
  pd.all.nona.highl <- pd.all.nona[which(pd.all.nona$elev.factor == "Highland"),]#Grab o
nly highland cases.

  #Calculate Total Response of Carbon
  f.all <- scale(pd.all.nona$f, scale = FALSE)
  f.coast <- scale(pd.all.nona.coast$f, scale = FALSE)
  f.midel <- scale(pd.all.nona.midel$f, scale = FALSE)
  f.highl <- scale(pd.all.nona.highl$f, scale = FALSE)
```

```
  #Calculate Partial Dependence of Carbon for Chosen Variable
  f.j <- scale(pd.all.nona$j, scale = FALSE)
  f.j.coast <- scale(pd.all.nona.coast$j, scale = FALSE)
  f.j.midel <- scale(pd.all.nona.midel$j, scale = FALSE)
  f.j.highl <- scale(pd.all.nona.highl$j, scale = FALSE)

  #Calculate Combined Partial Dependence of Carbon to all Other Variables
  f.no.j <- scale(pd.all.nona$no.j, scale = FALSE)
  f.noj.coast <- scale(pd.all.nona.coast$no.j, scale = FALSE)
  f.noj.midel <- scale(pd.all.nona.midel$no.j, scale = FALSE)
  f.noj.highl <- scale(pd.all.nona.highl$no.j, scale = FALSE)

  #Calculate Friedman's H-Stat to get the proportion of the effect due to interaction wi
th the other variables
  h.all <- sqrt(sum((f.all - (f.j + f.no.j))^2) / sum(f.all^2))
  h.coast <- sqrt(sum((f.coast - (f.j.coast + f.noj.coast))^2) / sum(f.coast^2))
  h.midel <- sqrt(sum((f.midel - (f.j.midel + f.noj.midel))^2) / sum(f.midel^2))
  h.highl <- sqrt(sum((f.highl - (f.j.highl + f.noj.highl))^2) / sum(f.highl^2))

  #Assign the Friedman's H-Stat to a dataframe
  C.Result.df <- data.frame(var = "Precip Mean", H.Stat = h.all, H.Stat.Coast = h.coast,
H.Stat.Mid = h.midel, H.Stat.High = h.highl)

  #Model Effect Size of the Chosen Variable (Amount of the per mil variance explained by
the chosen variable)
  sqrt(sum((f.j)^2))
  sqrt(sum((f.j.coast)^2))
  sqrt(sum((f.j.midel)^2))
  sqrt(sum((f.j.highl)^2))

  #Add the effect size to the dataframe
  meant.EF <- data.frame(EF = sqrt(sum((f.j)^2)), EF.coast = sqrt(sum((f.j.coast)^2)), E
F.midel = sqrt(sum((f.j.midel)^2)), EF.high = sqrt(sum((f.j.highl)^2)))
  C.Result.df <- cbind(C.Result.df, meant.EF)

  #Percent Effect of the Chosen Variable (Percent of the variance in Carbon explained by
the chosen variable)
  sqrt(sum(f.j^2) / sum(f.all^2))
  sqrt(sum(f.j.coast^2) / sum(f.coast^2))
  sqrt(sum(f.j.midel^2) / sum(f.midel^2))
  sqrt(sum(f.j.highl^2) / sum(f.highl^2))

  #Assign the percent effect size to the dataframe
  meant.EF.per <- data.frame(EF.per = sqrt(sum(f.j^2) / sum(f.all^2)), EF.per.coast = sq
rt(sum(f.j.coast^2) / sum(f.coast^2)), EF.per.midel = sqrt(sum(f.j.midel^2) / sum(f.mide
l^2)), EF.per.high = sqrt(sum(f.j.highl^2) / sum(f.highl^2)))
  C.Result.df <- cbind(C.Result.df, meant.EF.per)

  #Now use the Friedman's H-Stat to control the effect size and percent to get the amoun
t of the variance explained by the chosen variable without its interaction with other va
riables
```

```
  #Effect Size Controlled
  sqrt(sum((f.j*(1-h.all))^2))
  sqrt(sum((f.j.coast*(1-h.coast))^2))
  sqrt(sum((f.j.midel*(1-h.midel))^2))
  sqrt(sum((f.j.highl*(1-h.highl))^2))

  #Assign the controlled effect size to a dataframe
  meant.EF.cont <- data.frame(EF.cont = sqrt(sum((f.j*(1-h.all))^2)), EF.cont.coast = sq
rt(sum((f.j.coast*(1-h.coast))^2)), EF.cont.midel = sqrt(sum((f.j.midel*(1-h.midel))^2
)), EF.cont.highl = sqrt(sum((f.j.highl*(1-h.highl))^2)))
  C.Result.df <- cbind(C.Result.df, meant.EF.cont)

  #Percent Effect of the Chosen Variable Controlled with Friedman's H-Stat
  #Effect Size Percent Controlled
  sqrt(sum((f.j*(1-h.all))^2) / sum(f.all^2))
  sqrt(sum((f.j.coast*(1-h.coast))^2) / sum(f.coast^2))
  sqrt(sum((f.j.midel*(1-h.midel))^2) / sum(f.midel^2))
  sqrt(sum((f.j.highl*(1-h.highl))^2) / sum(f.highl^2))

  #Assign the controlled percent effect size to a dataframe
  meant.EF.per.cont <- data.frame(EF.per.cont = sqrt(sum((f.j*(1-h.all))^2) / sum(f.all^
2)), EF.per.cont.coast = sqrt(sum((f.j.coast*(1-h.coast))^2) / sum(f.coast^2)), EF.per.c
ont.midel = sqrt(sum((f.j.midel*(1-h.midel))^2) / sum(f.midel^2)), EF.per.cont.highl = s
qrt(sum((f.j.highl*(1-h.highl))^2) / sum(f.highl^2)))
  C.Result.df <- cbind(C.Result.df, meant.EF.per.cont)


  if (k == 1) {
    Var.C.Result.df <- C.Result.df
  }
  else {
  Var.C.Result.df <- rbind(Var.C.Result.df, C.Result.df)
  }
}

#Grab the column averages (this will be the 100 runs of 100 different var values average
d together)
Var.avg <- data.frame(row.names = "Precip Mean", t(colMeans(Var.C.Result.df[2:21])))

#Add the new variable values to the Total dataframe
Total.C.Result.df <- rbind(Total.C.Result.df, Var.avg)
```

Repeat for Precip Seasonality

```
#Choose the variable of interest. This will need to be repeated for each of our predicto
r variables (Temp_Mean, Temp_Mean_Seasonality, Precip_Mean, Precip_Mean_Seasonality, and
Demography.spd)
feature.name <- c("Precip_Mean_Seasonality")
```

Prediction function

```
predictor <- ia$predictor
```

Now get all the interactions for the chosen variable, calculate the Friedman's H-stat (proportion of the effect due to the variable's interaction with the other variables), calulate the effect size as the model sum of squares, discount the effect size based on the Friedman's H-stat, and calculate the effect size a a percent of the variation explained.

```
#iterate the data sampling for interactions 100 times.
runs <- seq(1, 100, 1)

#Make an object for the sampling output to go into
myia <- list(res = data.table(), qr = data.frame())

#Make a datafame object for the results of each iteration
C.Result.df <- data.frame()

#Make an empty dataframe for the total results to append to
Var.C.Result.df <- data.frame()


for (k in runs) {

  myia <- interaction.run.single(
  dataSample = data.sample,
  feature.name = feature.name[1],
  grid.size = grid.size,
  batch.size = batch.size,
  q = myq,
  predictor = predictor
  )


  partial_dat <- myia$res#make a dataframe with the data generated for the interactions
  partial_dat$.y.hat <- myia$qr#add the predicted values to the dataframe
  partial_dat$.class <- 1#specify 1 so when we do the prediction R knows this is a regre
ssion problem

  #collapse into 'F(x)' values - this will provide the average of all of the predictions
for each unique value of the chosen variable. So average of all predictions per observat
ion on grid and per value of the feature.
  pd.all <- data.table::dcast(partial_dat, .feature + .id + elev.factor + .class ~ .typ
e,
                        value.var = ".y.hat", fun.aggregate = mean
)

  pd.all.nona <- pd.all[which(!is.na(pd.all$f)),]#drop NA rows, leaving the 100 aggregat
ed predictions
  pd.all.nona.coast <- pd.all.nona[which(pd.all.nona$elev.factor == "Coastal"),]#Grab on
ly coastal cases.
  pd.all.nona.midel <- pd.all.nona[which(pd.all.nona$elev.factor == "Mid-elevation"),]#G
rab only mid-elevation cases.
  pd.all.nona.highl <- pd.all.nona[which(pd.all.nona$elev.factor == "Highland"),]#Grab o
nly highland cases.

  #Calculate Total Response of Carbon
  f.all <- scale(pd.all.nona$f, scale = FALSE)
  f.coast <- scale(pd.all.nona.coast$f, scale = FALSE)
  f.midel <- scale(pd.all.nona.midel$f, scale = FALSE)
  f.highl <- scale(pd.all.nona.highl$f, scale = FALSE)
```

```
  #Calculate Partial Dependence of Carbon for Chosen Variable
  f.j <- scale(pd.all.nona$j, scale = FALSE)
  f.j.coast <- scale(pd.all.nona.coast$j, scale = FALSE)
  f.j.midel <- scale(pd.all.nona.midel$j, scale = FALSE)
  f.j.highl <- scale(pd.all.nona.highl$j, scale = FALSE)

  #Calculate Combined Partial Dependence of Carbon to all Other Variables
  f.no.j <- scale(pd.all.nona$no.j, scale = FALSE)
  f.noj.coast <- scale(pd.all.nona.coast$no.j, scale = FALSE)
  f.noj.midel <- scale(pd.all.nona.midel$no.j, scale = FALSE)
  f.noj.highl <- scale(pd.all.nona.highl$no.j, scale = FALSE)

  #Calculate Friedman's H-Stat to get the proportion of the effect due to interaction wi
th the other variables
  h.all <- sqrt(sum((f.all - (f.j + f.no.j))^2) / sum(f.all^2))
  h.coast <- sqrt(sum((f.coast - (f.j.coast + f.noj.coast))^2) / sum(f.coast^2))
  h.midel <- sqrt(sum((f.midel - (f.j.midel + f.noj.midel))^2) / sum(f.midel^2))
  h.highl <- sqrt(sum((f.highl - (f.j.highl + f.noj.highl))^2) / sum(f.highl^2))

  #Assign the Friedman's H-Stat to a dataframe
  C.Result.df <- data.frame(var = "Precip Mean Seasonality", H.Stat = h.all, H.Stat.Coas
t = h.coast, H.Stat.Mid = h.midel, H.Stat.High = h.highl)

  #Model Effect Size of the Chosen Variable (Amount of the per mil variance explained by
the chosen variable)
  sqrt(sum((f.j)^2))
  sqrt(sum((f.j.coast)^2))
  sqrt(sum((f.j.midel)^2))
  sqrt(sum((f.j.highl)^2))

  #Add the effect size to the dataframe
  meant.EF <- data.frame(EF = sqrt(sum((f.j)^2)), EF.coast = sqrt(sum((f.j.coast)^2)), E
F.midel = sqrt(sum((f.j.midel)^2)), EF.high = sqrt(sum((f.j.highl)^2)))
  C.Result.df <- cbind(C.Result.df, meant.EF)

  #Percent Effect of the Chosen Variable (Percent of the variance in Carbon explained by
the chosen variable)
  sqrt(sum(f.j^2) / sum(f.all^2))
  sqrt(sum(f.j.coast^2) / sum(f.coast^2))
  sqrt(sum(f.j.midel^2) / sum(f.midel^2))
  sqrt(sum(f.j.highl^2) / sum(f.highl^2))

  #Assign the percent effect size to the dataframe
  meant.EF.per <- data.frame(EF.per = sqrt(sum(f.j^2) / sum(f.all^2)), EF.per.coast = sq
rt(sum(f.j.coast^2) / sum(f.coast^2)), EF.per.midel = sqrt(sum(f.j.midel^2) / sum(f.mide
l^2)), EF.per.high = sqrt(sum(f.j.highl^2) / sum(f.highl^2)))
  C.Result.df <- cbind(C.Result.df, meant.EF.per)

  #Now use the Friedman's H-Stat to control the effect size and percent to get the amoun
t of the variance explained by the chosen variable without its interaction with other va
riables
```

```
  #Effect Size Controlled
  sqrt(sum((f.j*(1-h.all))^2))
  sqrt(sum((f.j.coast*(1-h.coast))^2))
  sqrt(sum((f.j.midel*(1-h.midel))^2))
  sqrt(sum((f.j.highl*(1-h.highl))^2))

  #Assign the controlled effect size to a dataframe
  meant.EF.cont <- data.frame(EF.cont = sqrt(sum((f.j*(1-h.all))^2)), EF.cont.coast = sq
rt(sum((f.j.coast*(1-h.coast))^2)), EF.cont.midel = sqrt(sum((f.j.midel*(1-h.midel))^2
)), EF.cont.highl = sqrt(sum((f.j.highl*(1-h.highl))^2)))
  C.Result.df <- cbind(C.Result.df, meant.EF.cont)

  #Percent Effect of the Chosen Variable Controlled with Friedman's H-Stat
  #Effect Size Percent Controlled
  sqrt(sum((f.j*(1-h.all))^2) / sum(f.all^2))
  sqrt(sum((f.j.coast*(1-h.coast))^2) / sum(f.coast^2))
  sqrt(sum((f.j.midel*(1-h.midel))^2) / sum(f.midel^2))
  sqrt(sum((f.j.highl*(1-h.highl))^2) / sum(f.highl^2))

  #Assign the controlled percent effect size to a dataframe
  meant.EF.per.cont <- data.frame(EF.per.cont = sqrt(sum((f.j*(1-h.all))^2) / sum(f.all^
2)), EF.per.cont.coast = sqrt(sum((f.j.coast*(1-h.coast))^2) / sum(f.coast^2)), EF.per.c
ont.midel = sqrt(sum((f.j.midel*(1-h.midel))^2) / sum(f.midel^2)), EF.per.cont.highl = s
qrt(sum((f.j.highl*(1-h.highl))^2) / sum(f.highl^2)))
  C.Result.df <- cbind(C.Result.df, meant.EF.per.cont)


  if (k == 1) {
    Var.C.Result.df <- C.Result.df
  }
  else {
  Var.C.Result.df <- rbind(Var.C.Result.df, C.Result.df)
  }
}

#Grab the column averages (this will be the 100 runs of 100 different var values average
d together)
Var.avg <- data.frame(row.names = "Precip Mean Seasonality", t(colMeans(Var.C.Result.df[
2:21])))

#Add the new variable values to the Total dataframe
Total.C.Result.df <- rbind(Total.C.Result.df, Var.avg)
```

Repeat for Temp Seasonality

```
#Choose the variable of interest. This will need to be repeated for each of our predicto
r variables (Temp_Mean, Temp_Mean_Seasonality, Precip_Mean, Precip_Mean_Seasonality, and
Demography.spd)
feature.name <- c("Temp_Mean_Seasonality")
```

Prediction function

```
predictor <- ia$predictor
```

Now get all the interactions for the chosen variable, calculate the Friedman's H-stat (proportion of the effect due to the variable's interaction with the other variables), calulate the effect size as the model sum of squares, discount the effect size based on the Friedman's H-stat, and calculate the effect size a a percent of the variation explained.

```
#iterate the data sampling for interactions 100 times.
runs <- seq(1, 100, 1)

#Make an object for the sampling output to go into
myia <- list(res = data.table(), qr = data.frame())

#Make a datafame object for the results of each iteration
C.Result.df <- data.frame()

#Make an empty dataframe for the total results to append to
Var.C.Result.df <- data.frame()


for (k in runs) {

  myia <- interaction.run.single(
  dataSample = data.sample,
  feature.name = feature.name[1],
  grid.size = grid.size,
  batch.size = batch.size,
  q = myq,
  predictor = predictor
  )


  partial_dat <- myia$res#make a dataframe with the data generated for the interactions
  partial_dat$.y.hat <- myia$qr#add the predicted values to the dataframe
  partial_dat$.class <- 1#specify 1 so when we do the prediction R knows this is a regre
ssion problem

  #collapse into 'F(x)' values - this will provide the average of all of the predictions
for each unique value of the chosen variable. So average of all predictions per observat
ion on grid and per value of the feature.
  pd.all <- data.table::dcast(partial_dat, .feature + .id + elev.factor + .class ~ .typ
e,
                        value.var = ".y.hat", fun.aggregate = mean
)

  pd.all.nona <- pd.all[which(!is.na(pd.all$f)),]#drop NA rows, leaving the 100 aggregat
ed predictions
  pd.all.nona.coast <- pd.all.nona[which(pd.all.nona$elev.factor == "Coastal"),]#Grab on
ly coastal cases.
  pd.all.nona.midel <- pd.all.nona[which(pd.all.nona$elev.factor == "Mid-elevation"),]#G
rab only mid-elevation cases.
  pd.all.nona.highl <- pd.all.nona[which(pd.all.nona$elev.factor == "Highland"),]#Grab o
nly highland cases.

  #Calculate Total Response of Carbon
  f.all <- scale(pd.all.nona$f, scale = FALSE)
  f.coast <- scale(pd.all.nona.coast$f, scale = FALSE)
  f.midel <- scale(pd.all.nona.midel$f, scale = FALSE)
  f.highl <- scale(pd.all.nona.highl$f, scale = FALSE)
```

```
  #Calculate Partial Dependence of Carbon for Chosen Variable
  f.j <- scale(pd.all.nona$j, scale = FALSE)
  f.j.coast <- scale(pd.all.nona.coast$j, scale = FALSE)
  f.j.midel <- scale(pd.all.nona.midel$j, scale = FALSE)
  f.j.highl <- scale(pd.all.nona.highl$j, scale = FALSE)

  #Calculate Combined Partial Dependence of Carbon to all Other Variables
  f.no.j <- scale(pd.all.nona$no.j, scale = FALSE)
  f.noj.coast <- scale(pd.all.nona.coast$no.j, scale = FALSE)
  f.noj.midel <- scale(pd.all.nona.midel$no.j, scale = FALSE)
  f.noj.highl <- scale(pd.all.nona.highl$no.j, scale = FALSE)

  #Calculate Friedman's H-Stat to get the proportion of the effect due to interaction wi
th the other variables
  h.all <- sqrt(sum((f.all - (f.j + f.no.j))^2) / sum(f.all^2))
  h.coast <- sqrt(sum((f.coast - (f.j.coast + f.noj.coast))^2) / sum(f.coast^2))
  h.midel <- sqrt(sum((f.midel - (f.j.midel + f.noj.midel))^2) / sum(f.midel^2))
  h.highl <- sqrt(sum((f.highl - (f.j.highl + f.noj.highl))^2) / sum(f.highl^2))

  #Assign the Friedman's H-Stat to a dataframe
  C.Result.df <- data.frame(var = "Temp Mean Seasonality", H.Stat = h.all, H.Stat.Coast
 = h.coast, H.Stat.Mid = h.midel, H.Stat.High = h.highl)

  #Model Effect Size of the Chosen Variable (Amount of the per mil variance explained by
the chosen variable)
  sqrt(sum((f.j)^2))
  sqrt(sum((f.j.coast)^2))
  sqrt(sum((f.j.midel)^2))
  sqrt(sum((f.j.highl)^2))

  #Add the effect size to the dataframe
  meant.EF <- data.frame(EF = sqrt(sum((f.j)^2)), EF.coast = sqrt(sum((f.j.coast)^2)), E
F.midel = sqrt(sum((f.j.midel)^2)), EF.high = sqrt(sum((f.j.highl)^2)))
  C.Result.df <- cbind(C.Result.df, meant.EF)

  #Percent Effect of the Chosen Variable (Percent of the variance in Carbon explained by
the chosen variable)
  sqrt(sum(f.j^2) / sum(f.all^2))
  sqrt(sum(f.j.coast^2) / sum(f.coast^2))
  sqrt(sum(f.j.midel^2) / sum(f.midel^2))
  sqrt(sum(f.j.highl^2) / sum(f.highl^2))

  #Assign the percent effect size to the dataframe
  meant.EF.per <- data.frame(EF.per = sqrt(sum(f.j^2) / sum(f.all^2)), EF.per.coast = sq
rt(sum(f.j.coast^2) / sum(f.coast^2)), EF.per.midel = sqrt(sum(f.j.midel^2) / sum(f.mide
l^2)), EF.per.high = sqrt(sum(f.j.highl^2) / sum(f.highl^2)))
  C.Result.df <- cbind(C.Result.df, meant.EF.per)

  #Now use the Friedman's H-Stat to control the effect size and percent to get the amoun
t of the variance explained by the chosen variable without its interaction with other va
riables
```

```
  #Effect Size Controlled
  sqrt(sum((f.j*(1-h.all))^2))
  sqrt(sum((f.j.coast*(1-h.coast))^2))
  sqrt(sum((f.j.midel*(1-h.midel))^2))
  sqrt(sum((f.j.highl*(1-h.highl))^2))

  #Assign the controlled effect size to a dataframe
  meant.EF.cont <- data.frame(EF.cont = sqrt(sum((f.j*(1-h.all))^2)), EF.cont.coast = sq
rt(sum((f.j.coast*(1-h.coast))^2)), EF.cont.midel = sqrt(sum((f.j.midel*(1-h.midel))^2
)), EF.cont.highl = sqrt(sum((f.j.highl*(1-h.highl))^2)))
  C.Result.df <- cbind(C.Result.df, meant.EF.cont)

  #Percent Effect of the Chosen Variable Controlled with Friedman's H-Stat
  #Effect Size Percent Controlled
  sqrt(sum((f.j*(1-h.all))^2) / sum(f.all^2))
  sqrt(sum((f.j.coast*(1-h.coast))^2) / sum(f.coast^2))
  sqrt(sum((f.j.midel*(1-h.midel))^2) / sum(f.midel^2))
  sqrt(sum((f.j.highl*(1-h.highl))^2) / sum(f.highl^2))

  #Assign the controlled percent effect size to a dataframe
  meant.EF.per.cont <- data.frame(EF.per.cont = sqrt(sum((f.j*(1-h.all))^2) / sum(f.all^
2)), EF.per.cont.coast = sqrt(sum((f.j.coast*(1-h.coast))^2) / sum(f.coast^2)), EF.per.c
ont.midel = sqrt(sum((f.j.midel*(1-h.midel))^2) / sum(f.midel^2)), EF.per.cont.highl = s
qrt(sum((f.j.highl*(1-h.highl))^2) / sum(f.highl^2)))
  C.Result.df <- cbind(C.Result.df, meant.EF.per.cont)


  if (k == 1) {
    Var.C.Result.df <- C.Result.df
  }
  else {
  Var.C.Result.df <- rbind(Var.C.Result.df, C.Result.df)
  }
}

#Grab the column averages (this will be the 100 runs of 100 different var values average
d together)
Var.avg <- data.frame(row.names = "Temp Mean Seasonality", t(colMeans(Var.C.Result.df[2:
21])))

#Add the new variable values to the Total dataframe
Total.C.Result.df <- rbind(Total.C.Result.df, Var.avg)

#Generate the Cumulative Climate Effect
Cum.Clim. <- data.frame(row.names = "Cumulative Climate", t(colSums(Total.C.Result.df)))

#Add Cumulative Climate to the Total Effect Size Dataframe
Total.C.Result.df <- rbind(Total.C.Result.df, Cum.Clim.)

#Pull Out the Key Effect Sizes to store in their own dataframe
Cumulative.EF.C <- data.frame(Elev.cat = c("Coastal", "Mid-elevation", "Highland"), Cum.
Clim.EF = c(Total.C.Result.df[5,14], Total.C.Result.df[5,15], Total.C.Result.df[5,16]),
```

```
 Cum.Clim.EF.percent = c(Total.C.Result.df[5,18], Total.C.Result.df[5,19], Total.C.Resul
t.df[5,20]))

Cumulative.EF
```

```
##          Elev.cat Cum.Clim.EF Cum.Clim.EF.percent    Dem.EF Dem.EF.percent
## 1         Coastal   19.659594           0.7742414 1.3080191     0.05484111
## 2 Mid-elevation    9.712703           0.5590055 1.1027920     0.07113437
## 3        Highland    2.196065           0.4695958 0.7135108     0.11511053
```

```
Cumulative.EF.C
```

```
##          Elev.cat Cum.Clim.EF Cum.Clim.EF.percent
## 1         Coastal    3.856206           0.5511095
## 2 Mid-elevation    8.577675           0.5272065
## 3        Highland    4.952236           0.8269320
```

Repeat for Demography

```
#Choose the variable of interest. This will need to be repeated for each of our predicto
r variables (Temp_Mean, Temp_Mean_Seasonality, Precip_Mean, Precip_Mean_Seasonality, and
Demography.spd)
feature.name <- c("Demography.kde.corr.14c")
```

Prediction function

```
predictor <- ia$predictor
```

Now get all the interactions for the chosen variable, calculate the Friedman's H-stat (proportion of the effect due to the variable's interaction with the other variables), calulate the effect size as the model sum of squares, discount the effect size based on the Friedman's H-stat, and calculate the effect size a a percent of the variation explained.

```
#iterate the data sampling for interactions 100 times.
runs <- seq(1, 100, 1)

#Make an object for the sampling output to go into
myia <- list(res = data.table(), qr = data.frame())

#Make a datafame object for the results of each iteration
C.Result.df <- data.frame()

#Make an empty dataframe for the total results to append to
Var.C.Result.df <- data.frame()


for (k in runs) {

  myia <- interaction.run.single(
  dataSample = data.sample,
  feature.name = feature.name[1],
  grid.size = grid.size,
  batch.size = batch.size,
  q = myq,
  predictor = predictor
  )


  partial_dat <- myia$res#make a dataframe with the data generated for the interactions
  partial_dat$.y.hat <- myia$qr#add the predicted values to the dataframe
  partial_dat$.class <- 1#specify 1 so when we do the prediction R knows this is a regre
ssion problem

  #collapse into 'F(x)' values - this will provide the average of all of the predictions
for each unique value of the chosen variable. So average of all predictions per observat
ion on grid and per value of the feature.
  pd.all <- data.table::dcast(partial_dat, .feature + .id + elev.factor + .class ~ .typ
e,
                        value.var = ".y.hat", fun.aggregate = mean
)

  pd.all.nona <- pd.all[which(!is.na(pd.all$f)),]#drop NA rows, leaving the 100 aggregat
ed predictions
  pd.all.nona.coast <- pd.all.nona[which(pd.all.nona$elev.factor == "Coastal"),]#Grab on
ly coastal cases.
  pd.all.nona.midel <- pd.all.nona[which(pd.all.nona$elev.factor == "Mid-elevation"),]#G
rab only mid-elevation cases.
  pd.all.nona.highl <- pd.all.nona[which(pd.all.nona$elev.factor == "Highland"),]#Grab o
nly highland cases.

  #Calculate Total Response of Carbon
  f.all <- scale(pd.all.nona$f, scale = FALSE)
  f.coast <- scale(pd.all.nona.coast$f, scale = FALSE)
  f.midel <- scale(pd.all.nona.midel$f, scale = FALSE)
  f.highl <- scale(pd.all.nona.highl$f, scale = FALSE)
```

```
  #Calculate Partial Dependence of Carbon for Chosen Variable
  f.j <- scale(pd.all.nona$j, scale = FALSE)
  f.j.coast <- scale(pd.all.nona.coast$j, scale = FALSE)
  f.j.midel <- scale(pd.all.nona.midel$j, scale = FALSE)
  f.j.highl <- scale(pd.all.nona.highl$j, scale = FALSE)

  #Calculate Combined Partial Dependence of Carbon to all Other Variables
  f.no.j <- scale(pd.all.nona$no.j, scale = FALSE)
  f.noj.coast <- scale(pd.all.nona.coast$no.j, scale = FALSE)
  f.noj.midel <- scale(pd.all.nona.midel$no.j, scale = FALSE)
  f.noj.highl <- scale(pd.all.nona.highl$no.j, scale = FALSE)

  #Calculate Friedman's H-Stat to get the proportion of the effect due to interaction wi
th the other variables
  h.all <- sqrt(sum((f.all - (f.j + f.no.j))^2) / sum(f.all^2))
  h.coast <- sqrt(sum((f.coast - (f.j.coast + f.noj.coast))^2) / sum(f.coast^2))
  h.midel <- sqrt(sum((f.midel - (f.j.midel + f.noj.midel))^2) / sum(f.midel^2))
  h.highl <- sqrt(sum((f.highl - (f.j.highl + f.noj.highl))^2) / sum(f.highl^2))

  #Assign the Friedman's H-Stat to a dataframe
  C.Result.df <- data.frame(var = "Demography", H.Stat = h.all, H.Stat.Coast = h.coast,
 H.Stat.Mid = h.midel, H.Stat.High = h.highl)

  #Model Effect Size of the Chosen Variable (Amount of the per mil variance explained by
the chosen variable)
  sqrt(sum((f.j)^2))
  sqrt(sum((f.j.coast)^2))
  sqrt(sum((f.j.midel)^2))
  sqrt(sum((f.j.highl)^2))

  #Add the effect size to the dataframe
  meant.EF <- data.frame(EF = sqrt(sum((f.j)^2)), EF.coast = sqrt(sum((f.j.coast)^2)), E
F.midel = sqrt(sum((f.j.midel)^2)), EF.high = sqrt(sum((f.j.highl)^2)))
  C.Result.df <- cbind(C.Result.df, meant.EF)

  #Percent Effect of the Chosen Variable (Percent of the variance in Carbon explained by
the chosen variable)
  sqrt(sum(f.j^2) / sum(f.all^2))
  sqrt(sum(f.j.coast^2) / sum(f.coast^2))
  sqrt(sum(f.j.midel^2) / sum(f.midel^2))
  sqrt(sum(f.j.highl^2) / sum(f.highl^2))

  #Assign the percent effect size to the dataframe
  meant.EF.per <- data.frame(EF.per = sqrt(sum(f.j^2) / sum(f.all^2)), EF.per.coast = sq
rt(sum(f.j.coast^2) / sum(f.coast^2)), EF.per.midel = sqrt(sum(f.j.midel^2) / sum(f.mide
l^2)), EF.per.high = sqrt(sum(f.j.highl^2) / sum(f.highl^2)))
  C.Result.df <- cbind(C.Result.df, meant.EF.per)

  #Now use the Friedman's H-Stat to control the effect size and percent to get the amoun
t of the variance explained by the chosen variable without its interaction with other va
riables
```

```
  #Effect Size Controlled
  sqrt(sum((f.j*(1-h.all))^2))
  sqrt(sum((f.j.coast*(1-h.coast))^2))
  sqrt(sum((f.j.midel*(1-h.midel))^2))
  sqrt(sum((f.j.highl*(1-h.highl))^2))

  #Assign the controlled effect size to a dataframe
  meant.EF.cont <- data.frame(EF.cont = sqrt(sum((f.j*(1-h.all))^2)), EF.cont.coast = sq
rt(sum((f.j.coast*(1-h.coast))^2)), EF.cont.midel = sqrt(sum((f.j.midel*(1-h.midel))^2
)), EF.cont.highl = sqrt(sum((f.j.highl*(1-h.highl))^2)))
  C.Result.df <- cbind(C.Result.df, meant.EF.cont)

  #Percent Effect of the Chosen Variable Controlled with Friedman's H-Stat
  #Effect Size Percent Controlled
  sqrt(sum((f.j*(1-h.all))^2) / sum(f.all^2))
  sqrt(sum((f.j.coast*(1-h.coast))^2) / sum(f.coast^2))
  sqrt(sum((f.j.midel*(1-h.midel))^2) / sum(f.midel^2))
  sqrt(sum((f.j.highl*(1-h.highl))^2) / sum(f.highl^2))

  #Assign the controlled percent effect size to a dataframe
  meant.EF.per.cont <- data.frame(EF.per.cont = sqrt(sum((f.j*(1-h.all))^2) / sum(f.all^
2)), EF.per.cont.coast = sqrt(sum((f.j.coast*(1-h.coast))^2) / sum(f.coast^2)), EF.per.c
ont.midel = sqrt(sum((f.j.midel*(1-h.midel))^2) / sum(f.midel^2)), EF.per.cont.highl = s
qrt(sum((f.j.highl*(1-h.highl))^2) / sum(f.highl^2)))
  C.Result.df <- cbind(C.Result.df, meant.EF.per.cont)


  if (k == 1) {
    Var.C.Result.df <- C.Result.df
  }
  else {
  Var.C.Result.df <- rbind(Var.C.Result.df, C.Result.df)
  }
}

#Grab the column averages (this will be the 100 runs of 100 different var values average
d together)
Var.avg <- data.frame(row.names = "Demography", t(colMeans(Var.C.Result.df[2:21])))

#Add the new variable values to the Total dataframe
Total.C.Result.df <- rbind(Total.C.Result.df, Var.avg)

#Add demography results to the cumulative effect size dataframe
Cumulative.EF.C$Dem.EF <- c(sqrt(sum((f.j.coast*(1-h.coast))^2)), sqrt(sum((f.j.midel*(1
-h.midel))^2)), sqrt(sum((f.j.highl*(1-h.highl))^2)))
Cumulative.EF.C$Dem.EF.percent <- c(sqrt(sum((f.j.coast*(1-h.coast))^2) / sum(f.coast^2
)), sqrt(sum((f.j.midel*(1-h.midel))^2) / sum(f.midel^2)), sqrt(sum((f.j.highl*(1-h.high
l))^2) / sum(f.highl^2)))
```

Show the complete set of effect size calculations.

```
#show the complete set of effect size calculations - for each variable, this is: H-stat,
Effect Size, Effect Size percent, Effect size controlled by H-stat, Effect size percent
 controlled by H-stat
round(Total.C.Result.df, 2)
```

```
##                          H.Stat H.Stat.Coast H.Stat.Mid H.Stat.High    EF
## Temp Mean                  0.40         0.32       0.30        0.35 12.00
## Precip Mean                0.47         0.31       0.32        0.34 11.68
## Precip Mean Seasonality    0.48         0.34       0.31        0.21 12.92
## Temp Mean Seasonality      0.48         0.46       0.39        0.24 11.89
## Cumulative Climate         1.83         1.42       1.31        1.14 48.49
## Demography                 0.58         0.34       0.33        0.18 12.94
##                          EF.coast EF.midel EF.high EF.per EF.per.coast
## Temp Mean                    1.12     1.48    3.23   0.52         0.16
## Precip Mean                  1.77     2.15    1.64   0.51         0.25
## Precip Mean Seasonality      1.72     4.72    1.42   0.56         0.24
## Temp Mean Seasonality        1.37     4.54    0.86   0.51         0.20
## Cumulative Climate           5.98    12.88    7.14   2.11         0.85
## Demography                   1.05     1.42    0.74   0.56         0.15
##                          EF.per.midel EF.per.high EF.cont EF.cont.coast
## Temp Mean                        0.09        0.53    7.24          0.77
## Precip Mean                      0.13        0.28    6.17          1.22
## Precip Mean Seasonality          0.29        0.23    6.66          1.13
## Temp Mean Seasonality            0.28        0.14    6.19          0.73
## Cumulative Climate               0.79        1.19   26.26          3.86
## Demography                       0.09        0.13    5.37          0.70
##                          EF.cont.midel EF.cont.highl EF.per.cont
## Temp Mean                         1.04          2.11        0.31
## Precip Mean                       1.47          1.07        0.27
## Precip Mean Seasonality           3.30          1.12        0.29
## Temp Mean Seasonality             2.78          0.65        0.27
## Cumulative Climate                8.58          4.95        1.14
## Demography                        0.95          0.61        0.23
##                          EF.per.cont.coast EF.per.cont.midel EF.per.cont.highl
## Temp Mean                             0.11              0.06              0.35
## Precip Mean                           0.18              0.09              0.19
## Precip Mean Seasonality               0.16              0.20              0.18
## Temp Mean Seasonality                 0.11              0.17              0.11
## Cumulative Climate                    0.55              0.53              0.83
## Demography                            0.10              0.06              0.10
```

Table S4. Complete Effect Size Calcuations for δ^{13}C‰. For each variable we report the complete model H-stat and effect size as well as the H-stat and effect sizes within each elevational zone.

Key Results

```
C.Results <- Total.C.Result.df[,c(14:16, 18:20)]
round(C.Results, 4)
```

```
##                          EF.cont.coast EF.cont.midel EF.cont.highl
## Temp Mean                       0.7670        1.0384        2.1104
## Precip Mean                     1.2247        1.4652        1.0744
## Precip Mean Seasonality         1.1325        3.2957        1.1202
## Temp Mean Seasonality           0.7321        2.7784        0.6471
## Cumulative Climate              3.8562        8.5777        4.9522
## Demography                      0.6972        0.9546        0.6087
##                          EF.per.cont.coast EF.per.cont.midel EF.per.cont.highl
## Temp Mean                           0.1087            0.0650            0.3491
## Precip Mean                         0.1767            0.0897            0.1876
## Precip Mean Seasonality             0.1593            0.2029            0.1841
## Temp Mean Seasonality               0.1063            0.1696            0.1061
## Cumulative Climate                  0.5511            0.5272            0.8269
## Demography                          0.1016            0.0586            0.1039
```

Table 2 Part 2. Carbon Results.

Effect Size Results

Make Figure 4 with the output from the models using both the uncorrected and corrected KDE estimates

```
#Lets make Figure 4 a 4 panel figure, using both the uncorr and corr KDE results

#Put the effect size data into a dataframe for plotting
n.bar.ef.kde <- data.frame("elev.factor" = c("Coastal", "Mid-elevation", "Highland"), "C
lim.Exp" = c(0.76, 0.51, 0.44), "Dem.Exp" = c(0.07, 0.22, 0.11))
c.bar.ef.kde <- data.frame("elev.factor" = c("Coastal", "Mid-elevation", "Highland"), "C
lim.Exp" = c(0.58, 0.55, 0.81), "Dem.Exp" = c(0.13, 0.16, 0.10))

n.bar.ef.kde$Clim.Exp<- n.bar.ef.kde$Clim.Exp*-1 #Change the sign to negative for the le
ft hand side of the barplot

c.bar.ef.kde$Clim.Exp <- c.bar.ef.kde$Clim.Exp*-1 #Change the sign to negative for the l
eft hand side of the barplot

#Also make the effect size data from the uncorrected KDE model runs
n.bar.ef.kde.corr <- data.frame("elev.factor" = c("Coastal", "Mid-elevation", "Highland"
), "Clim.Exp" = c(0.77, 0.56, 0.47), "Dem.Exp" = c(0.06, 0.07, 0.12))
c.bar.ef.kde.corr <- data.frame("elev.factor" = c("Coastal", "Mid-elevation", "Highland"
), "Clim.Exp" = c(0.55, 0.53, 0.83), "Dem.Exp" = c(0.10, 0.06, 0.10))

n.bar.ef.kde.corr$Clim.Exp<- n.bar.ef.kde.corr$Clim.Exp*-1 #Change the sign to negative
 for the left hand side of the barplot

c.bar.ef.kde.corr$Clim.Exp <- c.bar.ef.kde.corr$Clim.Exp*-1 #Change the sign to negative
for the left hand side of the barplot



#png(filename = "Figure4_allKDE.11.22.21.png", width = 5, height = 5, units = "in", res
 = 300)
par(oma = c(4,4,2,1),mar = c(0,2,1,1), mfrow = c(2,2), pty = "s", xpd = NA)
#par(mfrow = c(2,2), pty = "s", mar = c(4,6,1,1))
par(xpd = FALSE)
barplot(height = n.bar.ef.kde$Clim.Exp,
        xlim = c(-1,1),
        xaxt = "n",
        col = viridis(3),
        border = "white",
        horiz=T
        )
barplot(height = n.bar.ef.kde$Dem.Exp,
        add=T,
        xaxt = "n",
        col = viridis(3),
        border = "white",
        horiz=T
        )

mtext(side = 3, at = -0.5, "Climate", cex = 0.75)
mtext(side = 3, at = 0.5, "Demography", cex = 0.75)
mtext(side = 2, at = 0.7, line = 0, las = 1, adj = 1, "Coastal", cex = 0.75)
mtext(side = 2, at = 1.9, line = 0, las = 1, adj = 1, "Mid-elevation", cex = 0.75)
mtext(side = 2, at = 3.1, line = 0, las = 1, adj = 1, "Highland", cex = 0.75)
```

```
mtext(side = 3, at = 0, line = 1, expression({delta}^15*N~'\u2030'), cex = 1)
mtext(side = 1, at = 0.95, line = -1, "a)", cex = 0.75)
axis(side = 1,
     at = seq(-1, 1, by = 0.25),
     c("", "", "", "", "", "", "", "", "")
     )

par(xpd = FALSE)
barplot(height = c.bar.ef.kde$Clim.Exp,
        xlim = c(-1,1),
        xaxt = "n",
        #names.arg = n.bar.ef$Elev.cat,
        col = viridis(3),
        border = "white",
       horiz=T
        )
barplot(height = c.bar.ef.kde$Dem.Exp,
        add=T,
        xaxt = "n",
        col = viridis(3),
        border = "white",
        horiz=T
        )

mtext(side = 3, at = -0.5, "Climate", cex = 0.75)
mtext(side = 3, at = 0.5, "Demography", cex = 0.75)
#mtext(side = 1, line = 3, at = -1.15, "Effect Size (% Variation Explained)", cex = 0.7
5)
mtext(side = 3, at = 0, line = 1, expression({delta}^13*C~'\u2030'), cex = 1)
mtext(side = 1, line = -1, at = 0.95, "b)", cex = 0.75)
axis(side = 1,
     at = seq(-1, 1, by = 0.25),
     c("", "", "", "", "", "", "", "", "")
     )

par(xpd = FALSE)
barplot(height = n.bar.ef.kde.corr$Clim.Exp,
        xlim = c(-1,1),
        xaxt = "n",
        col = viridis(3),
        border = "white",
        horiz=T
        )
barplot(height = n.bar.ef.kde.corr$Dem.Exp,
        add=T,
        xaxt = "n",
        col = viridis(3),
        border = "white",
        horiz=T
        )

#mtext(side = 3, at = -0.5, "Climate", cex = 0.75)
```

```
#mtext(side = 3, at = 0.5, "Demography", cex = 0.75)
mtext(side = 2, at = 0.7, line = 0, las = 1, adj = 1, "Coastal", cex = 0.75)
mtext(side = 2, at = 1.9, line = 0, las = 1, adj = 1, "Mid-elevation", cex = 0.75)
mtext(side = 2, at = 3.1, line = 0, las = 1, adj = 1, "Highland", cex = 0.75)
#mtext(side = 3, at = 0, line = 1, expression({delta}^15*N~'\u2030'), cex = 1)
mtext(side = 1, at = 0.95, line = -1, "c)", cex = 0.75)
axis(side = 1,
     at = seq(-1, 1, by = 0.25),
     c(1, 0.75, 0.5, 0.25, 0, 0.25, 0.5, 0.75, 1)
     )

par(xpd = FALSE)
barplot(height = c.bar.ef.kde.corr$Clim.Exp,
        xlim = c(-1,1),
        xaxt = "n",
        #names.arg = n.bar.ef$Elev.cat,
        col = viridis(3),
        border = "white",
       horiz=T
        )
barplot(height = c.bar.ef.kde.corr$Dem.Exp,
        add=T,
        xaxt = "n",
        col = viridis(3),
        border = "white",
        horiz=T
        )

#mtext(side = 3, at = -0.5, "Climate", cex = 0.75)
#mtext(side = 3, at = 0.5, "Demography", cex = 0.75)
mtext(side = 1, line = 3, at = -1.30, "Effect Size (% Variation Explained)", cex = 0.75)
#mtext(side = 3, at = 0, line = 1, expression({delta}^13*C~'\u2030'), cex = 1)
mtext(side = 1, line = -1, at = 0.95, "d)", cex = 0.75)


axis(side = 1,
     at = seq(-1, 1, by = 0.25),
     c(1, 0.75, 0.5, 0.25, 0, 0.25, 0.5, 0.75, 1)
     )
```

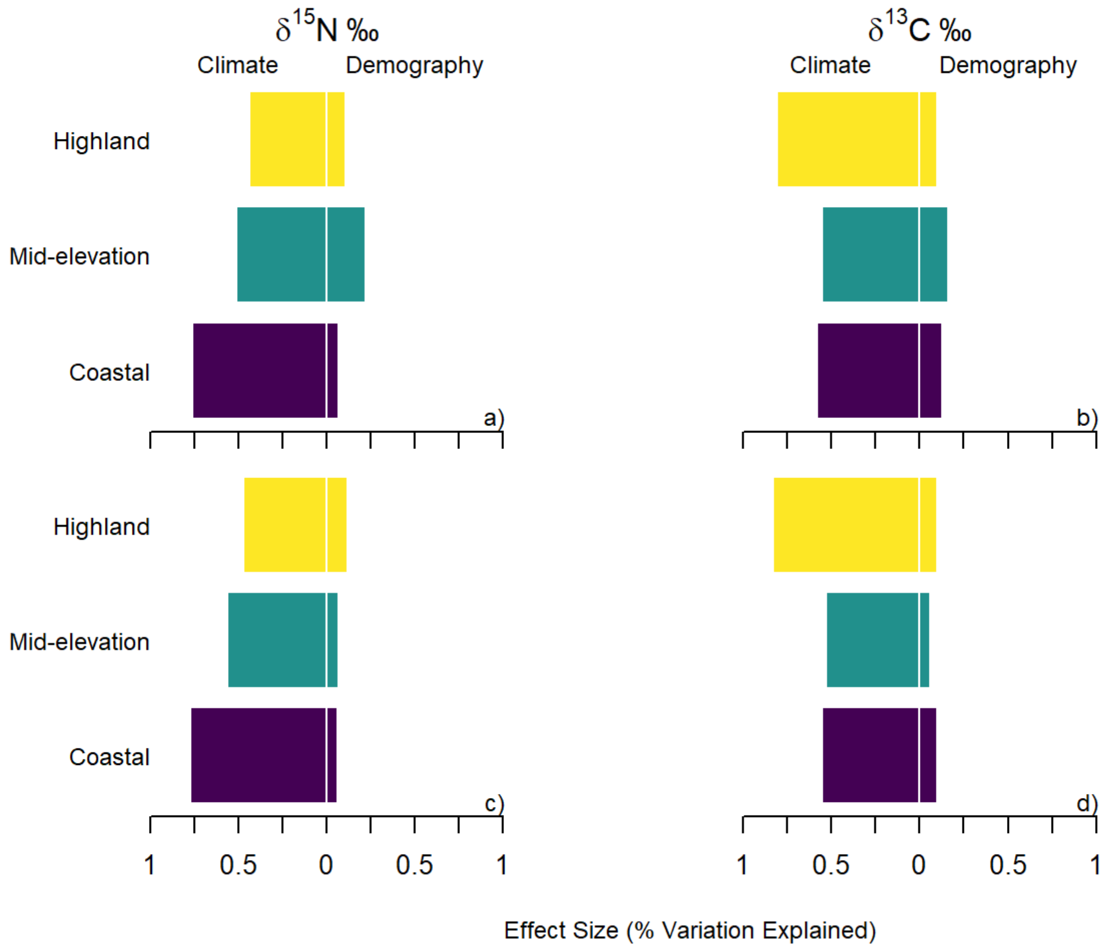


```
#dev.off()
```

References

27. J. Pulgar Vidal, *Geografía del Perú: Las ocho regiones naturales del Perú*. (Editorial Universo, Lima, Peru, ed. 8th, 1981).

149. G. Claeskens, N. L. Hjort, *Model selection and model averaging*. (Cambridge University Press, Cambridge, UK, 2008).

140. L. Breiman, Random forests. Machine learning 45, 5-32 (2001).

142. C. F. Dormann et al., Collinearity: a review of methods to deal with it and a simulation study evaluating their performance. Ecography 36, 27-46 (2013).

143. J. Li, spm: Spatial Predictive Modeling. R packager version 1.2.0. https://CRAN.R-project.org/ (https://CRAN.R-project.org/) package=spm, (2019).

144. J. H. Friedman, B. E. Popescu, Predictive learning via rule ensembles. Annals of Applied Statistics 2, 916-954 (2008).

136. T. A. Surovell, et al., Correcting temporal frequency distributions for taphonomic bias. Journal of Archaeological Science 36, 1715-1724 (2009).

150. J.S. Evans, M.A. Murphy, rfUtilities. R package version 2.1-3. https://cran.r-project.org/package=rfUtilities (https://cran.r-project.org/package=rfUtilities),(2018).

151. J.S. Evans, M.A. Murphy, Z.A. Holden, S.A. Cushman, "Modeling species distribution and change using Random Forests" in *Predictive Species and Habitat Modeling in Landscape Ecology: Concepts and Applications,* C.A. Drew, Y.F. Wiersma, F. Huettmann, Eds (Springer, New York, 2011)

Below is the source code for running the Friedman's *H* statistic calculations in the methods. **This code should be saved as its own R file in the same file location as the main R markdown.** This code will need to be read in for the effect size calculations to work.

```
# For a grid (grid.dat) of features (param features) creates a blown up dataset
# with the marginals of features not in 'features'. The samples (n.sample.dist
# number of samples) for the marginals are drawn from dist.dat. If n.sample.dist
# is not set, the whole cartesian product between grid.dat and dist.dat is built
# grid.dat only needs to contain the columns which are fixed. Decide here which
# grid points should be used.
# dist.dat needs to contain all columns
library(R6)
MarginalGenerator <- R6Class(
  public = list(
    initialize = function(grid.dat, dist.dat, features, n.sample.dist = NULL,
                          y = NULL, id.dist = FALSE, cartesian = FALSE) {
      assert_data_table(grid.dat)
      assert_data_table(dist.dat)
      if (!is.null(n.sample.dist) && n.sample.dist > nrow(dist.dat)) {
        message("Number of repetitions larger than number of unique permutations per row.
          Switching to cartesian = TRUE")
        cartesian <- TRUE
      }
      if (cartesian) {
        n.sample.dist <- nrow(dist.dat)
      }
      stopifnot(cartesian | !is.null(n.sample.dist))
      assert_true(all(features %in% colnames(grid.dat)))
      assert_true(all(colnames(grid.dat) %in% colnames(dist.dat)))
      assert_character(features, unique = TRUE)
      assert_data_frame(y, null.ok = TRUE, nrows = nrow(dist.dat))

      private$grid.dat <- grid.dat
      private$dist.dat <- dist.dat
      private$features <- features
      private$features.rest <- setdiff(colnames(dist.dat), features)
      private$n.sample.dist <- n.sample.dist
      private$id.dist <- id.dist
      private$y <- y
      self$n_total <- n.sample.dist * nrow(grid.dat)

      private$grid.index <- rep(1:nrow(grid.dat), each = n.sample.dist)

      if (!cartesian && (nrow(dist.dat) == nrow(grid.dat)) &&
```

```
      (n.sample.dist == 1)) {
    # special case which amounts to shuffling
    private$dist.index <- sample(1:nrow(dist.dat), size = nrow(dist.dat))
  } else {
    private$dist.index <- unlist(lapply(
      1:nrow(grid.dat),
      function(x) sample(1:nrow(dist.dat), size = n.sample.dist)
    ))
  }
},

finished = FALSE,
n_total = NULL,

# Return the next n samples
next.batch = function(n, y = FALSE) {
  if (!self$finished) {
    pointer <- private$counter
    if (n > (length(private$grid.index) - pointer)) self$finished <- TRUE

    n.left <- length(private$grid.index) - pointer
    step <- min(n - 1, n.left)
    batch.index <- pointer:(pointer + step)

    partial_j1 <- private$grid.dat[private$grid.index[batch.index],
                                   private$features,
                                   with = FALSE
    ]

    partial_j2 <- private$dist.dat[private$dist.index[batch.index],
                                   private$features.rest,
                                   with = FALSE
    ]

    partial_j <- cbind(partial_j1, partial_j2)

    partial_j$.id <- private$grid.index[batch.index]
    if (private$id.dist) {
      partial_j$.id.dist <- private$dist.index[batch.index]
    }
    private$counter <- private$counter + n

    if (y) partial_j <- cbind(partial_j, private$y[partial_j$.id.dist, ])
    partial_j
```

```
      }
    },

    all = function() {
      private$counter <- 1
      self$next.batch(length(private$grid.index))
    }
  ),

  private = list(
    counter = 1,
    grid.dat = NULL,
    dist.dat = NULL,
    features = NULL,
    features.rest = NULL,
    n.sample.dist = NULL,
    id.dist = NULL,
    grid.index = NULL,
    dist.index = NULL,
    y = NULL
  )
)

interaction.run.single = function(batch.size, dataSample, feature.name,
                                  grid.size, predictor, q) {

  assert_data_table(dataSample)
  assert_character(feature.name,
                   min.len = 1, max.len = 2,
                   any.missing = FALSE
  )
  assert_number(grid.size)

  grid.dat <- dataSample[sample(1:nrow(dataSample), size = grid.size), ]
  dist.dat <- dataSample

  res.intermediate <- data.table()
  if (length(feature.name) == 1) {
    mg_j <- MarginalGenerator$new(grid.dat, dist.dat, feature.name, cartesian = TRUE)
    batch.size.split <- floor(batch.size) / 2
    mg_noj <- MarginalGenerator$new(grid.dat, dist.dat,
                                    setdiff(colnames(dataSample), feature.name),
                                    cartesian = TRUE
    )
```

```
    while (!mg_j$finished) {
      partial_j <- mg_j$next.batch(batch.size.split)
      partial_j$.type <- "j"
      partial_noj <- mg_noj$next.batch(batch.size.split)
      partial_noj$.type <- "no.j"
      grid.dat$.type <- "f"
      grid.dat$.id <- 1:nrow(grid.dat)
      res.intermediate <- rbind(res.intermediate, partial_j, partial_noj,
                                grid.dat,
                                use.names = TRUE
      )
    }
  } else if (length(feature.name) == 2) {
    batch.size.split <- floor(batch.size / 3)
    mg_jk <- MarginalGenerator$new(grid.dat, dist.dat, feature.name,
                                   cartesian = TRUE
    )
    mg_j <- MarginalGenerator$new(grid.dat, dist.dat, feature.name[1],
                                  cartesian = TRUE
    )
    mg_k <- MarginalGenerator$new(grid.dat, dist.dat, feature.name[2],
                                  cartesian = TRUE
    )

    while (!mg_j$finished) {
      partial_jk <- mg_jk$next.batch(batch.size.split)
      partial_jk$.type <- "jk"
      partial_j <- mg_j$next.batch(batch.size.split)
      partial_j$.type <- "j"
      partial_k <- mg_k$next.batch(batch.size.split)
      partial_k$.type <- "k"
      res.intermediate <- rbind(res.intermediate, partial_jk, partial_j,
                                partial_k,
                                use.names = TRUE
      )
    }
  }
  predictResults <- predictor$predict(data.frame(res.intermediate))
  qResults <- q(predictResults)
  res.intermediate$.feature <- paste(feature.name, collapse = ":")
  list(res = res.intermediate, qr = qResults)
  #aggregate.interaction(res.intermediate, qResults, feature.name)
}
```

```
h.test <- function(f.all, f.j, f.no.j) {
  assert_numeric(f.all, any.missing = FALSE)
  assert_numeric(f.j, any.missing = FALSE)
  assert_numeric(f.no.j, any.missing = FALSE)
  # center
  f.all <- my.scale(f.all)
  f.j <- my.scale(f.j)
  f.no.j <- my.scale(f.no.j)
  # statistics
  sqrt(sum((f.all - (f.j + f.no.j))^2) / sum(f.all^2))
}

my.scale <- function(x) {
  x.scaled <- scale(x, center = TRUE, scale = FALSE)
  x.scaled[is.na(x.scaled)] <- 0
  x.scaled
}
```
