# Supplementary figures and images for: Climate and demography drive 7000 years of dietary change in the Central Andes
Source: Sci Rep. 2022 Feb 7;12:2026. doi: 10.1038/s41598-022-05774-y (PMC8821598; doi:10.1038/s41598-022-05774-y)

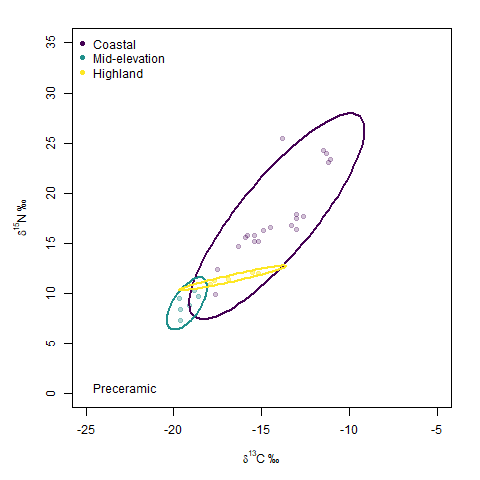

Supplement: Supplementary file 4 — Supplementary Information 4. [file 41598_2022_5774_MOESM4_ESM.gif]
