# Supplementary material for: Climate and demography drive 7000 years of dietary change in the Central Andes
Source: Sci Rep. 2022 Feb 7;12:2026. doi: 10.1038/s41598-022-05774-y (PMC8821598; doi:10.1038/s41598-022-05774-y)
Supplement: Supplementary file 5 — Supplementary Legends. [file 41598_2022_5774_MOESM5_ESM.docx]

Captions for Supplementary Materials

Supplementary Material 1: Complete R code to rerun the analysis performed in this manuscript.

Supplementary Material 2: Animated gif of 95% confidence interval plots of δ^15^N‰ and δ^13^C‰, showing the general changes over time. Plots produced using the **SIBER**^140^ and **animate^153^** packages in the R statistical environment.

Supplementary Data File 1: Complete set of individuals with δ^15^N‰ and δ^13^C‰ compiled for this work, including all individuals who are not included in the analysis due to either C:N ratio, age, or time period.

Supplementary Data File 2: Data file for rerunning the analysis. This file includes all individuals incorporated in the analysis as well as their respective climate, demography, elevation, and elevation zone data. This file may be read into the R code from Supplementary Material 1 to replicate the analysis.
